# Cancer Cell Acid Adaptation Gene Expression Response Is Correlated to Tumor-Specific Tissue Expression Profiles and Patient Survival

**DOI:** 10.3390/cancers12082183

**Published:** 2020-08-05

**Authors:** Jiayi Yao, Dominika Czaplinska, Renata Ialchina, Julie Schnipper, Bin Liu, Albin Sandelin, Stine Falsig Pedersen

**Affiliations:** 1 The Bioinformatics Centre, Department of Biology, University of Copenhagen, DK2200 Copenhagen, Denmark; jiayi.yao@bio.ku.dk; 2 Biotech Research and Innovation Centre, University of Copenhagen, DK2200 Copenhagen, Denmark; 3 Section for Cell Biology and Physiology, Department of Biology, University of Copenhagen, DK2100 Copenhagen, Denmark; dominika.czaplinska@bio.ku.dk (D.C.); renata.ialchina@bio.ku.dk (R.I.); julie_schnipper@hotmail.com (J.S.); 4 Cell Death and Metabolism, Center for Autophagy, Recycling and Disease, Danish Cancer Society Research Center, DK2100 Copenhagen, Denmark; liu@cancer.dk

**Keywords:** RNA sequencing, tumor microenvironment, chronic acidosis, survival analysis, acid adaptation, medical transcriptomics

## Abstract

The acidic pH of the tumor microenvironment plays a critical role in driving cancer development toward a more aggressive phenotype, but the underlying mechanisms are unclear. To this end, phenotypic and genotypic changes induced by adaptation of cancer cells to chronic acidosis have been studied. However, the generality of acid adaptation patterns across cell models and their correlation to the molecular phenotypes and aggressiveness of human cancers are essentially unknown. Here, we define an acid adaptation expression response shared across three cancer cell models, dominated by metabolic rewiring, extracellular matrix remodeling, and altered cell cycle regulation and DNA damage response. We find that many genes which are upregulated by acid adaptation are significantly correlated to patient survival, and more generally, that there are clear correlations between acid adaptation expression response and gene expression change between normal and tumor tissues, for a large subset of cancer patients. Our data support the notion that tumor microenvironment acidity is one of the key factors driving the selection of aggressive cancer cells in human patient tumors, yet it also induces a growth-limiting genotype that likely limits cancer cell growth until the cells are released from acidosis, for instance during invasion.

## 1. Introduction

Invasive cancers, regardless of their origin, acquire characteristic phenotypic traits during their development, including self-sufficiency with respect to growth signals, apoptosis evasion, profound metabolic changes, chemotherapy resistance, and the ability to invade and spread to secondary niches [1]. These changes are the result of a clonal evolution process, in which the combination of somatic mutations and evolutionary selection pressure leads to the preferential expansion of clones of cells that are particularly fit for survival and expansion in the harsh tumor microenvironment [2,3,4].

The microenvironmental conditions in solid tumors are a result of rapid cancer cell proliferation and growth which, in combination with insufficient vascularization, leads to hypoxia, glucose depletion, accumulation of lactate and other metabolites, and profound acidosis. While serum pH is around 7.4, corresponding to a H^+^ concentration ([H^+^]) of ~40 nM, the extracellular pH (pH_e_) in tumors can reach 6.2–6.8, i.e., [H^+^] ~630–160 nM [5,6,7,8,9]. A plethora of studies have investigated the impact of hypoxia on cancer development and the therapeutic potential of targeting hypoxia-inducible factors (reviewed in, e.g., [10]). In contrast, the role of extracellular acidosis in this process remains incompletely understood. Recent studies, mostly employing the acid adaptation of cultured cancer cells, have supported the hypothesis that extracellular acidosis is a driver of cancer aggressiveness. Thus, adaptation to acidosis was shown to favor epithelial-to-mesenchymal transition (EMT) [11,12], invasiveness and metastasis in cell culture and xenograft models of melanoma, cervical cancer, and breast and colon cancer [13,14,15,16]. Accordingly, studies of mouse breast tumors in vivo showed that acidosis (detected by pH (low) insertion peptide (pHLIP) labeling), carbonic anhydrase (CA9) expression and plasma membrane-localized LAMP2 correlated with invasive features such as the absence of laminin and the expression of matrix metalloproteinase (MMP)-9 and -14 [17]. Other phenotypic changes induced by the adaptation of cancer cells to acidosis include a shift from glycolytic metabolism toward increased glutaminolysis, fatty acids uptake and β-oxidation [9,15,18,19,20]. Cancer cell proliferation and growth are, however, generally reduced, or unaltered, as long as acidic pH_e_ is maintained [12,13].

The molecular mechanisms through which growth under chronic extracellular acidosis leads to this profound rewiring of cancer cells are poorly understood. While several studies have examined global changes in gene expression upon acidosis or lactic acidosis [15,21,22], the overlap between the changes induced by acidosis and those found in patient tumors, and the correlation of acidosis-responsive gene expression to overall patient survival, have to our knowledge never been comprehensively examined. Because acidic growth seems to drive at least some phenotypes akin to those found in highly aggressive cancers, an important question is whether there is a generic response to acid adaptation that is shared between cancer cells, and whether this response reflects the expression changes observed between tumors and normal tissues in patient samples. Such correlations may indicate that an acidic microenvironment can increase cancer aggressiveness, which in turn should be correlated to patient survival.

In the present study, by applying RNA-seq to three acid-adapted cancer cell lines we identify a shared acid adaptation response gene signature, which we compare with rich expression data from human tumors and normal tissue across many cancers, and overall survival data from the same patients. Our results reveal significant overlaps between gene signatures of acid-adapted cells and tumor tissues for subsets of patients, and we identify sets of shared genes from these signatures that correlate significantly—positively as well as negatively—with patient survival.

## 2. Results

### 2.1. Gene Expression Changes Induced by Chronic Adaptation of Cancer Cells to Acidosis

**Figure 1:**
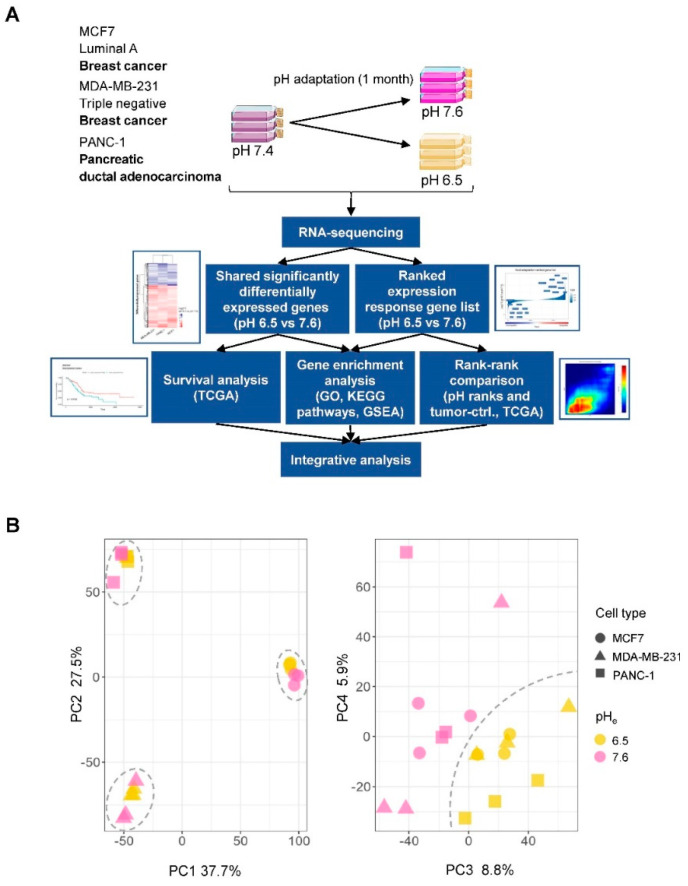
Overview of study procedures and analyses. (**A**) Overview of the experimental design, and individual and integrative analyses performed. Three cancer cell lines in triplicates (MDA-MB-231, MCF7 and PANC-1) were acid-adapted to pH 6.5 and 7.6 over a period of 1 month and subjected to RNA-sequencing analysis (RNA-seq). Below this, an overview of computational analyses performed is shown. Briefly, we used two strategies to define a shared acid adaptation gene expression profile: by defining sets of statistically significant genes, or by ranking genes by their expression fold change (pH 6.5 vs. 7.6; Figure 2). These lists were then subject to over-representation analyses (Figure 2), survival analysis in multiple cancers (Figure 3), and comparison to tumor-control tissue (ctrl.) RNA-seq experiments from patient samples (Figure 4 and Figure 5), to identify a final list of genes that are up-/downregulated in acid adaptation as well as in tumor vs. ctrl. experiments, and whose in vivo expression is associated with overall patient survival (Figure 6). (**B**) Principle component analysis (PCA) based on gene expression. The x- and y-axes show principle components (PCs) 1 and 2 in left panel, 3 and 4 in right panel. Percentage explained variance is shown at each axis. Each dot corresponds to one RNA-seq library. Symbol shapes correspond to cell lines and color corresponds to pH treatment. In the left panel, dashed circles show three groups corresponding to cell lines. In the right panel, the dashed line divides samples by pH treatment.

To understand the changes in gene expression profile induced by changes in environmental pH (pH_e_) across multiple cancer cell types, rather than in a single cancer cell type, we adapted three different cancer cell lines to growth at either pH 7.6 or 6.5 by adjusting the HCO_3_^−^ concentration of the medium [23], followed by culturing at the respective pH for at least 1 month at 5% CO_2_. Cells were lysed, and RNA was isolated and subjected to global RNA-seq in triplicates (Figure 1A, top). Principal component (PC) analysis of gene expression estimates from RNA-seq libraries showed that PC1 and PC2 separated the three cell lines, while PC3 and PC4 separated acid-adapted cells from control cells, indicating a shared acid adaptation response across the three cell lines (Figure 1B). Given this, we set out to analyze the shared acid adaptation response, and compared it to tumor-specific expression profiles and patient survival using a strategy outlined in Figure 1A.

### 2.2. Identification of a Shared Acid Adaptation Gene Response Profile

**Figure 2:**
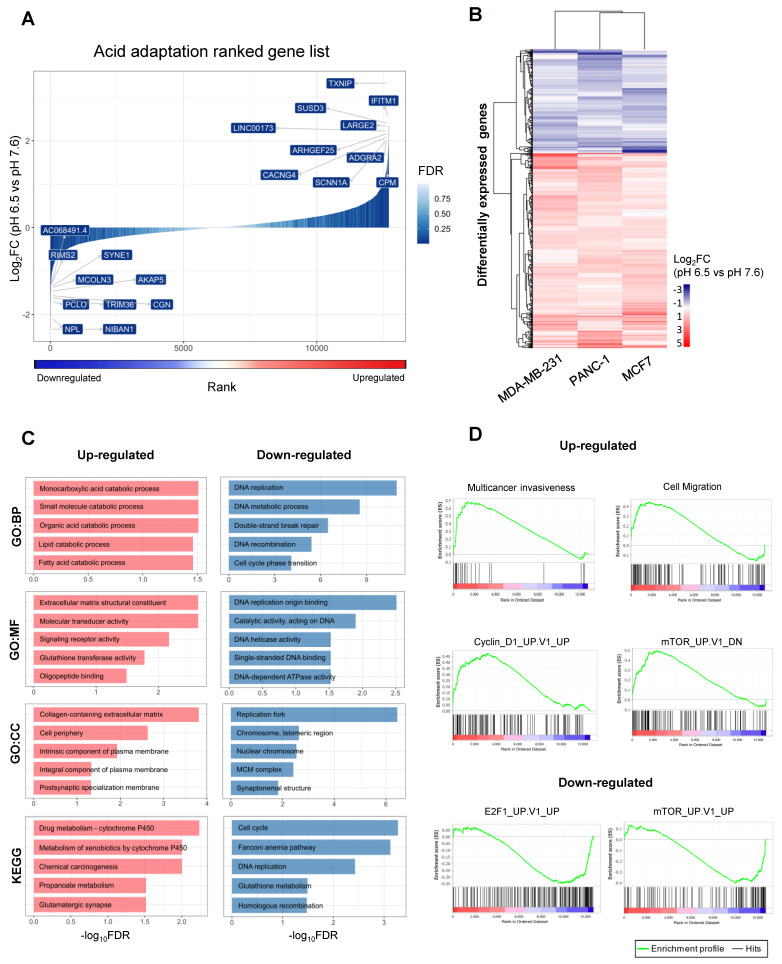
Identification of a shared acid adaptation expression response. (**A**) Fold change-based ranking of all genes differentially expressed in chronically acid-adapted cancer cells. The *x*-axis corresponds to the rank assigned to each gene from 1 to N based on gene expression fold change (pH 6.5 vs. pH 7.6) in log_2_ scale (*y*-axis), also illustrated by blue–red gradient below. Bar color intensity indicates differential expression significance, expressed as false discovery rate (FDR). Genes with the largest absolute fold change are labelled on the plot. (**B**) Heatmap visualization of differential expression change in chronically acid-adapted cells. Rows correspond to significantly differentially expressed genes (480 upregulated and 256 downregulated), and columns correspond to three cancer cell lines (MDA-MB-231, PANC-1 and MCF7). Color indicates average log_2_ fold change of gene expression (pH 6.5 vs. pH 7.6) across three replicates per condition. (**C**) Gene Ontology (GO) term analysis of differentially expressed genes in chronically acid-adapted cells. The *x*-axis shows GO term enrichment FDR values in −log_10_ scale for differentially expressed genes defined above. The *y*-axis shows the top 5 GO terms from three categories (BP—Biological process, MF—Molecular function, CC—Cellular Component) and pathways from the KEGG (Kyoto Encyclopedia of Genes and Genomes) database, ordered by FDR. Bar colors correspond to up- or downregulated gene sets as in (**B**,**D**) Gene set enrichment analyses (GSEA) based on acid adaptation-ranked gene list from A to gene lists in the SigDB database. A subset of comparisons is shown as GSEA plots: the SigDB gene list identifier is shown on top of each plot. The *y*-axis in subplot shows the enrichment score, reflecting how much the chosen gene set is over-represented at the top or bottom of the ranked acid adaptation gene list. In the *x*-axis, red indicates upregulation at pH 6.5, and blue downregulation, as in Figure 2A. Vertical lines in the lower part of the plot show where in the ranked acid adaptation gene list the genes in the selected SigDB gene list occurred.

The Limma method was used to identify genes with a significant and shared expression response to acid adaptation using a linear model (See Materials and Methods). Out of the 12,750 expressed genes, 478 were significantly upregulated, and 255 were downregulated across all three cell lines (abs (log_2_ fold change, log_2_FC) > 0.5, False Discovery Rate (FDR) < 0.05). Figure 2A shows a shared log_2_FC-ranked list of all expressed genes, colored by significance, while Figure 2B shows the mean log_2_FC in each cell line across triplicates for differentially expressed genes. Tables S1 and S2 list all significantly up- and downregulated genes, respectively. The response to pH was overall highly similar between the three cancer cell lines, although the magnitude of change in the expressions of specific genes differed between cell lines (Figure 2B). Among the top 10 upregulated genes was thioredoxin-interacting protein (*TXNIP*, also known as Vitamin D3-upregulated protein 1, *VDUP*1), previously reported to be upregulated in response to extracellular acidosis [22,24] (Figure 2A). The TXNIP protein has several important physiological roles: it is a potent negative regulator of glycolysis, and involved in redox homeostasis, differentiation and growth. *TXNIP* is generally downregulated in cancers, at least in part because it is negatively regulated by oncogenes such as Myc and ErbB2, and low *TXNIP* expression is associated with poor prognosis [25,26,27]. Confirming the relevance of this pathway, the *TXNIP* paralogs *ARRDC2* and *ARRDC4* were also among the significantly upregulated genes (Table S1).

Interestingly, several cation channels previously implicated in cancer development were significantly upregulated. For example, the α subunit of the epithelial sodium channel ENaC (*SCNN1A*) and the γ4 subunit of the voltage-dependent calcium channel (*CACNG4*) (Figure 2A) were among the 10 most upregulated genes. The genes encoding the acid-stimulated ion channel-1 (*ASIC1*), and the β1 subunit of the voltage-gated sodium channel (*SCN1B*), were also significantly upregulated in acid-adapted cells (Table S1). In accordance with our findings, *ASIC1* and *ASIC2* are upregulated in colorectal cancer cells subjected to acidosis [28]. ASICs and ENaC belong to the same channel family, and both ASIC1 [29] and ENaC [30] are acutely activated by acidic pH, and both channels are implicated in cancer development [11,31]. Similarly, both *CACNG4* [32] and *SCN1B* [33] are upregulated in cancer tissue and their gene products have been assigned a role in cancer progression.

Other highly upregulated genes included interferon-induced transmembrane protein 1 (*IFITM1*), which, together with other interferon-regulated genes, are highly upregulated in several cancers and assigned key roles in driving aggressiveness and chemotherapy resistance [34,35]. In addition, the genes encoding Sushi-containing Domain-3 (SUSD3), an estrogen-regulated membrane-localized protein previously found to be upregulated by chronic acidosis [15] and to play a key role in breast cancer cell migration [36], and *LARGE2*, a bifunctional glycosyltransferase involved in proteoglycan modification and hence in cell–extracellular matrix (ECM) interaction [37], were among the 10 most upregulated genes (Figure 2A).

The 10 most downregulated genes across all three acid-adapted cell lines included, firstly, the gene encoding the multidomain scaffold protein A-kinase anchoring protein-5 (AKAP5, a.k.a. AKAP79/150). AKAP5 is a key regulator of cellular cAMP and Ca^2+^ signaling and, downstream from this, a plethora of physiological processes, including glucose metabolism [38]. AKAP5 is not widely linked to cancer in the literature, yet low AKAP5 expression was correlated with poor prognosis in some stomach adenocarcinomas [39]. *NIBAN1*, a.k.a. *FAM129A*, which was also downregulated by acidic growth, is frequently upregulated in cancers, favoring cell motility yet decreasing autophagy [40,41]. Furthermore, mucolipin-3 (*MCOLN3*) was strongly downregulated upon acidosis-induced selection. Interestingly, *MCOLN3* codes for a predominantly endosomal Ca^2+^ channel of the TRP channel family, which is inhibited by acidic pH and plays important roles in endosomal Ca^2+^ and pH homeostasis [42]. Other genes strongly downregulated across all three cell lines included those coding for the tight junction protein cingulin (*CGN*), the E3 ubiquitin ligase *TRIM36*, and *SYNE1* (a.k.a. Nesprin-1), a nuclear envelope protein.

Gene Ontology (GO) terms associated to ECM composition and remodeling, and lipid and carboxylic acid metabolism, were over-represented in the upregulated genes, while GO terms associated with cell proliferation, replication fork function and DNA repair were over-represented in the downregulated genes (Figure 2C). Furthermore, the upregulation of cation channels (Figure 2A and above) was reflected in GO term analysis (Table S3, while Table S4 shows the GO terms for downregulated genes). KEGG pathway analysis showed an over-representation of cytochrome P450-drug and xenobiotic metabolism, chemical carcinogenesis, propanoate metabolism and glutaminergic synapse pathways in the upregulated genes, while downregulated genes were over-represented in the cell cycle, DNA replication, homologous recombination, glutathione metabolism and Fanconi anemia pathways. Notably, a downregulated glutathione metabolism was previously reported in acid-adapted cells, and shown to reflect a shift from glutathione production towards utilization of glutamine as a metabolic fuel [18]. The ranked acid adaptation gene set from Figure 2A was used to complement the above GO analysis with gene set enrichment analysis (GSEA) using the SigDb database of gene sets (Figure 2D). This revealed a clear gene rank enrichment of several oncological gene sets: acid adaptation-upregulated genes were enriched for gene sets associated with increased migration and invasiveness, gene sets upregulated after expression of CyclinD1 (CCND1) (a key regulator of G1-S phase transition), and genes downregulated after mTOR inhibition (Figure 2D). The link to mTOR signaling is notable, given the key role of mTOR in metabolic control. The pH sensitivity of mTOR signaling has previously been assigned a role in the impact of acidosis on metabolism, albeit in a short-term study (i.e., not long enough for acid-induced selection) where cytoplasmic acidosis was found to inhibit mTOR signaling [43,44]. Acid adaptation-downregulated genes were, correspondingly, enriched in gene sets upregulated by mTOR inhibition, or by overexpression of the transcription factor E2F1, a key player in the control of cell cycle progression.

Taken together, these analyses show that across multiple cancer cell types, chronic acidosis is associated with gene expression changes expected to reflect a profound metabolic shift, including the downregulation of fermentative glycolysis and upregulation of glutamine- and lipid-based metabolism, and the downregulation of cell division and DNA repair, as well as changes in ECM remodeling and ion channel activity.

### 2.3. In Vivo Expression of Genes Reacting to Chronic Acidosis Is Predictive of Cancer Patient Survival

The above analysis indicated that there was an overlap between cancer-regulated and acid adaptation-regulated gene sets, motivating a global investigation into whether the expression of acid adaptation-induced genes in patient tumors is correlated to patient overall survival (OS). To do this, RNA-seq data for multiple cancer types from the TCGA database were used. For each gene differentially expressed in all cell lines in the acid adaptation experiment above, patients were classified based on their mRNA expression of the specific genes into a high and low expression group, respectively, using the median expression of genes as the cut-off value.

**Figure 3:**
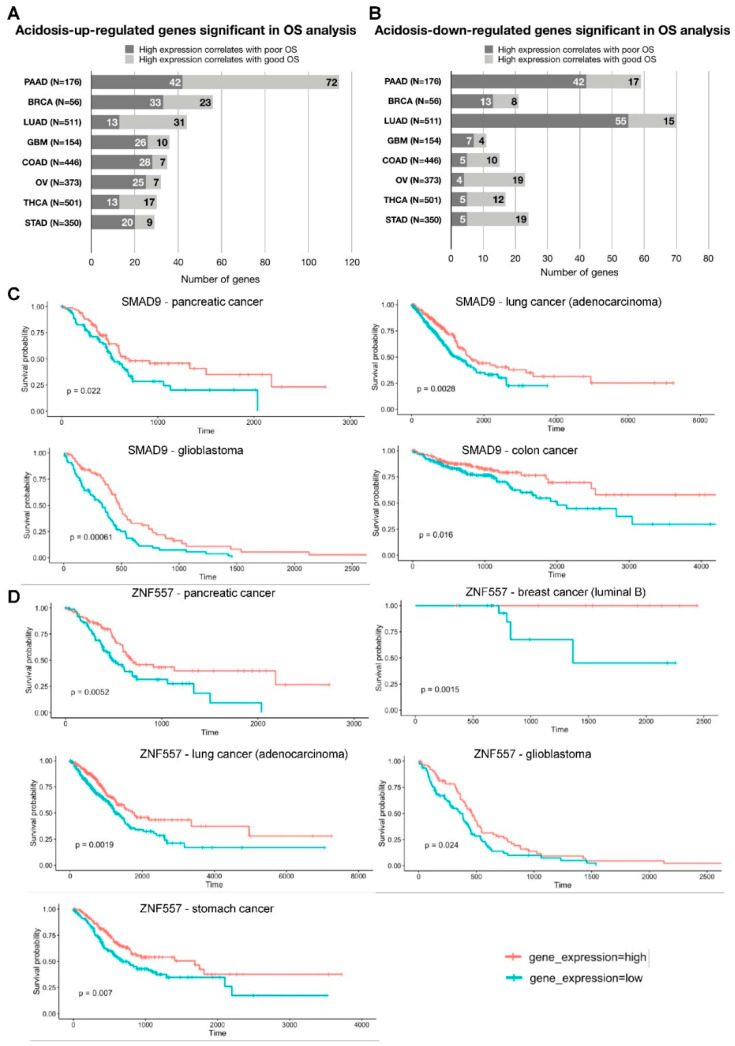
Correlation of genes differentially regulated in chronically acid adaptation cancer cells with patients’ overall survival. (**A**) Summary of patient overall survival (OS) analysis based on acid adaptation-upregulated genes. Bars show the number of genes significantly associated with overall survival (*x*-axis), stratified by whether high patient gene expression correlates with poorer or better OS (indicated by bar color). The *y*-axis shows cancer type, where N is the number of patients analyzed. PAAD—pancreatic cancer, BRCA—breast cancer (luminal B), LUAD—lung cancer (adenocarcinoma), GBM—glioblastoma, COAD—colon cancer, OV—ovarian cancer, THCA—thyroid cancer, STAD—stomach cancer. (**B**) Summary of patient OS analysis based on acid adaptation-downregulated genes. Arranged as in (**A**,**C**) Survival analysis based on SMAD9 expression levels. Kaplan–Meier overall survival analysis in pancreatic cancer, lung adenocarcinoma, glioblastoma and colon cancer patients, respectively, based on SMAD9 expression levels (GDC TCGA datasets). The *y*-axis shows survival probability, the *x*-axis shows time in days. Red line: patient group with high expression of the analyzed gene. Blue line: patient group with low expression of the analyzed gene. Survival analysis *p*-value is shown. (**D**) Survival analysis based on ZNF557 expression levels. Kaplan–Meier overall survival analysis in pancreatic cancer, breast cancer (luminal B), lung adenocarcinoma, glioblastoma and stomach cancer patients respectively, based on ZNF557 expression levels, arranged as in (**C**).

Kaplan–Meier analysis showed that 267 genes upregulated by chronic acidosis and 138 genes downregulated by chronic acidosis were significantly associated with OS. Specifically, expression of 114 genes upregulated by chronic acidosis in all cell lines was significantly correlated with OS of pancreatic cancer patients. In luminal B breast cancer, 56 acid adaptation-upregulated genes correlated significantly with OS, followed by lung cancer (adenocarcinoma), glioblastoma, colon cancer, ovarian cancer, thyroid cancer and stomach cancer (44, 36, 35, 32, 30 and 29 genes, respectively; Figure 3A). In pancreatic, lung and thyroid cancer, a high expression of the majority of acid adaptation-upregulated genes correlated with better OS. Breast, glioblastoma, colon, ovarian and stomach cancer cohorts shared the reverse pattern: for the majority of acid adaptation-upregulated genes, high expression correlated with poor OS outcome (Figure 3A). Additionally, we found 70 acid adaptation-downregulated genes whose expression correlated with OS of lung adenocarcinoma patients. The counts in descending order for other types of cancer were: Pancreatic cancer—59, stomach cancer—24, ovarian cancer—23, luminal B breast cancer—21, thyroid cancer—17, colon cancer—15 and glioblastoma—11 (Figure 3B). High expressions of the majority of acid adaptation-downregulated genes in lung adenocarcinoma, pancreatic cancer, breast cancer and glioblastoma correlated with poor OS. On the contrary, high expressions of the majority of acid adaptation-downregulated genes in stomach, ovarian, thyroid and colon cancers were associated with better OS (Figure 3B).

The data in Figure 3A,B are collectively consistent with the hypothesis that in stomach, ovarian and colon cancers, acidosis-related changes in gene expression may be more likely to be linked to worse OS, whereas in pancreatic and lung cancer, they are more likely to be linked to better OS. A necessary caveat is that these analyses are only correlative (see also Discussion), and the statistical power of the OS analysis is limited by the number of patient datasets available.

Two genes stood out in terms of their high correlation with OS in multiple cancers. Among the acid adaptation-upregulated genes, high expression of the gene encoding the transcription regulator SMAD9 (a.k.a. SMAD8) showed correlation with good OS in four types of cancer (pancreatic, lung, colon and glioblastoma; Figure 3C, Table 1). SMAD9, a receptor-SMAD activated by Bone Morphogenetic Protein (BMP) receptors, was also upregulated by acidosis in colon cancer cells [45]. Although the link of high expression to better OS makes SMAD9 upregulation an unlikely driver of acidosis-induced pro-tumorigenic effects, activating SMAD9 mutations were linked to some gastric cancers [46]. We also identified 5 genes (*LGR4*, *RARG*, *PNISR*, *PCOLCE2*, *RALGDS*) that shared the same patterns across three types of cancer, and 39 genes with two overlaps across eight cancer types (Table 1). As high expression of *LGR4, RARG* and *PCOLCE2* correlated with poor OS, these are interesting candidates for genes selected for in acidosis and linked to poor OS. The most common gene classes (based on the UniProt database) among genes with significant OS analysis results in at least two types of cancer were transcription regulators (9 genes), followed by genes involved in transport processes (8 genes), differentiation/morphogenesis (5 genes) and metabolism (4 genes) (Table 1).

Another gene with multiple cancer OS associations was the transcription factor *ZNF557*, which was downregulated by acid adaptation. Its low expression was associated with a worse OS outcome in five types of cancer (pancreatic, breast luminal B, lung adenocarcinoma, stomach and glioblastoma) (Figure 3D, Table 2). This supports that upregulation of *ZNF557*, an essentially unstudied member of the KRAB-ZNF family of zinc finger transcription factors and a transcriptional target of STAT3 [47], may be particularly relevant to the pro-tumorigenic effects of the acidic microenvironment, a possibility to be addressed in future studies. We furthermore discovered 5 genes (*MDGA1*, *VEGFC*, *MCM10*, *CTBP1-DT*, *CHMP4A*) with three OS correlations, and 22 genes with two OS correlations, across eight cancer types (Table 2). Of these, low expressions of *CTBP1-DT* and *CHMP4A* correlated with poor OS, making them possible candidates for acidosis-stimulated cancer aggressiveness. Among the acid adaptation-downregulated genes significant in OS analysis in at least two types of cancer, we found 17 genes associated with DNA replication/repair/cell cycle, 5 involved in transcription regulation and 4 playing key roles in differentiation and morphogenesis (Table 2).

All 182 acid adaptation-upregulated, and 84 acid adaptation-downregulated, genes that were significant in OS analysis in only one type of cancer are listed in Tables S5 and S6, respectively. Genes that showed contradictory OS results across cancer types (i.e., high and low expression was significantly associated with poor OS in different cancers) are listed in Table S7 (acid adaptation-upregulated) and Table S8 (acid adaptation-downregulated). Figures S1–S16 show plots for all genes with statistically significant OS results.

Taken together, these results show that a large fraction of acid adaptation-responsive genes correlate with patient survival across multiple cancers. Importantly, these genes fall into four major groups: acid adaptation-upregulated genes correlating with either good or poor OS, and acid adaptation-downregulated genes correlating with either better or worse OS across several types of cancers.

### 2.4. RRHO Analysis Identifies Substantial Overlap between Genes Upregulated in Acid Adapted Cells and Patient Tumor Tissue

**Figure 4:**
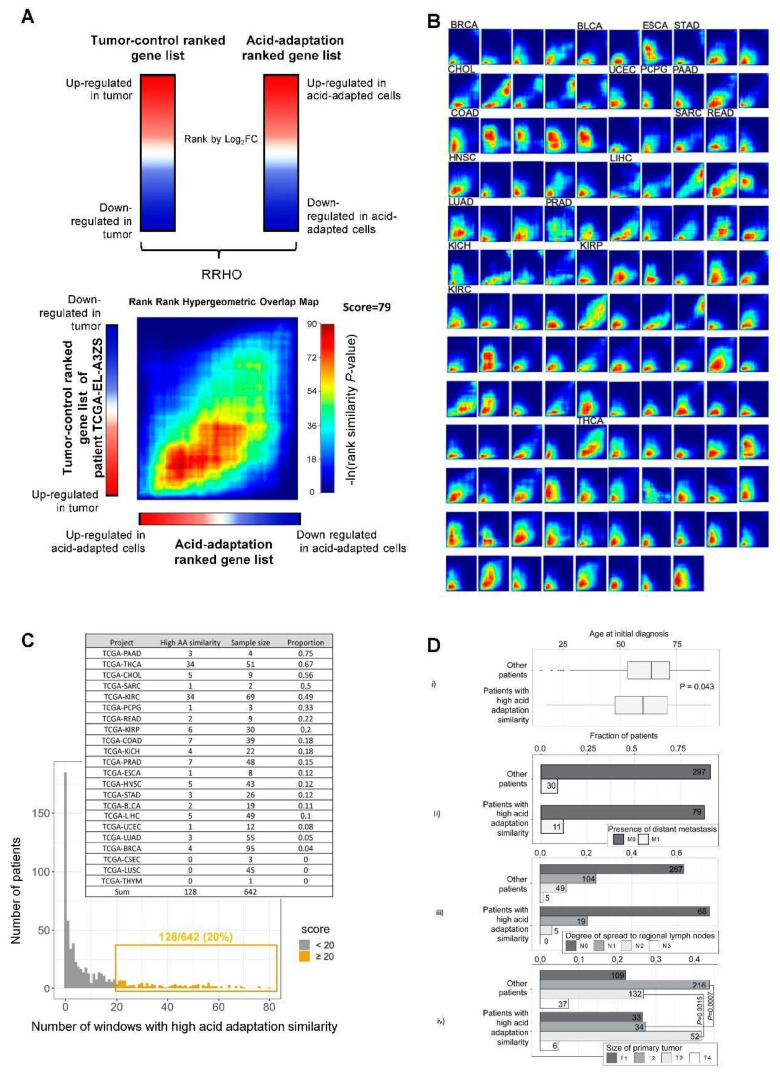
Rank–rank analysis of acid adaptation and patient tumor–ctrl. ranked gene lists. (**A**) Conceptual overview of rank–rank hypergeometric overlap (RRHO) analysis. Genes are ranked by their log2 fold expression change based on either tumor–ctrl. comparison for a given patient (left) or by acid adaptation response (pH 6.5 vs. 7.6). These ranked gene lists are compared in the rank–rank heat map below (exemplified by the comparison of data from the TCGA patient TCGA-EL-A3ZS and the acid adaptation gene list), identifying windows of genes with correlated ranks in respective gene lists and the significance of such correlated ranks, as indicated by color (based on ln(hypergeometric *p*-values) as shown). (**B**) Heat maps resulting from patient tumor–ctrl. vs. acid adaption gene list RRHO analyses showing high gene rank similarity. Each heat map corresponds to patient tumor–ctrl. vs. acid adaptation gene list analysis as in (**A**), where the acid adaptation gene list is always at the *x*-axis and the color scale is as in (**A**). Cancer types are indicated on top of heat maps: consecutive heat maps to the right have the same cancer type unless otherwise indicated (BRCA—breast cancer (luminal B), BLCA—bladder urothelial carcinoma, ESCA—esophageal carcinoma, STAD—stomach cancer, CHOL—cholangiocarcinoma, UCEC—uterine corpus endometrial carcinoma, PCPG—pheochromocytoma and paraganglioma, PAAD—pancreatic cancer, COAD—colon cancer, SARC—sarcoma, READ—renal adenocarcinoma, HNSC—head and neck squamous cell carcinoma, LIHC—liver hepatocellular carcinoma, LUAD—lung cancer (adenocarcinoma), PRAD—prostate adenocarcinoma, KICH—kidney chromophobe, KIRP—kidney renal papillary cell carcinoma, KIRC—kidney renal clear cell carcinoma, THCA—thyroid cancer). (**C**) Distribution of RRHO similarity scores across TCGA patients. The *y*-axis shows the number of patients with a given RRHO similarity score (*x*-axis) calculated as the fraction of windows with a *p* < 0.05 in rank–rank heat maps (out of a total of 100 windows). Analyses resulting in >20 windows with *p* < 0.05 (highlighted in yellow) were considered “high acid adaptation similarity”. The number of analyzed patients and the number of analyses resulting in high acid adaptation similarity scores are shown as an insert table. (**D**) Relation between high acid adaptation similarity score and patient metadata. Subpanel i shows the distribution of age of diagnosis split by whether patients are in the “high acid adaptation similarity” group, visualized as box plots. *p*-value is from two-sided *t* test. Subpanel ii–iv shows fraction of patients classified by TNM staging system. T: size of the primary tumor. T1, T2, T3, T4—size and/or extension of the primary tumor. N: degree of spread to regional lymph nodes. N0—no regional lymph nodes metastasis; N1—regional lymph node metastasis present (at some sites, tumor spread to closest or small number of regional lymph nodes); N2 —tumor spreads to an extent between N1 and N3; N3—tumor spread to more distant or numerous regional lymph nodes. M: presence of distant metastasis. M0—no distant metastasis; M1—metastasis to distant organs (beyond regional lymph nodes). Bars are split by whether patients are in the “high acid adaptation similarity” group. *p*-values from Fisher exact tests are shown, testing the decrease in T1 and increase in T2 in the “high acid adaptation similarity” group vs. remaining patients. Numbers on bars show patient counts.

Given that the expression level in vivo of a substantial number of acidosis-induced genes was correlated with overall patient survival, we reasoned that it would be informative to directly compare the acid adaptation gene expression response and the gene expression change between tumor and ctrl. tissue. This requires paired normal and tumor tissue data from the same patient, and we therefore downloaded TCGA RNA-seq data for 642 patients for which such paired data was available. For each patient, we ranked genes according to the tumor vs. ctrl. tissue RNA-seq log_2_FC. We will refer to such lists as “tumor–ctrl. ranked gene lists”. Each such ranked list was compared to a ranked gene list based on the pH 6.5 vs. 7.6, log_2_FC, as shown in Figure 2A, referred to as the “acid adaptation ranked gene list”. Using the rank–rank hypergeometric test method (RRHO) (Figure 4A), we sought to identify patients whose tumor–ctrl. ranked gene lists had a significantly similar gene order to that of the acid adaptation ranked gene list, and the set of genes that had similar orders in many such pairwise comparisons. In brief, RRHO analysis identifies windows of the two rank gene lists which have higher rank similarities than expected, and assigns *p*-values for each such window based on hypergeometric tests, which can be visualized as a heat map. For example, the heat map in Figure 4A shows an RRHO comparison between the patient TCGA-EL-A3ZS tumor–ctrl. ranked gene list (*y*-axis) vs. the acid adaptation ranked gene list (*x*-axis), where color intensity in the heat map indicates the gene rank similarity significance in a given window across respective ranked lists. In this example, we observed a high overlap in the upregulated genes between the tumor–ctrl. ranked gene list of the TCGA-EL-A3ZS patient and the acid adaptation ranked gene list (red areas), and a less strong but still substantial rank similarity between modestly downregulated genes in both sets (yellow–green areas). To identify clear cases of substantial rank similarity between patient tumor–ctrl. ranked lists and the acid adaptation ranked lists, we counted the fraction of windows in the heat map matrix which had a *p*-value < 0.05, and defined this number as the “acid adaptation similarity”. If >20% of windows had *p* < 0.05, we considered the patient tumor–ctrl. rank gene list to be highly similar to the acid adaptation ranked gene list. The group of 128/642 (~20%) tumor–ctrl. ranked gene lists satisfying this criterion were defined as the ‘high acid adaptation similarity group” (Figure 4C).

Within this group, in most cases, the genes with highest rank similarity were upregulated in pH 6.5 vs. 7.4 and upregulated in tumor vs. ctrl. Figure 4B shows all heat maps from patients in the high acid adaptation similarity group, while Figure 4C shows the distribution of patient ranked gene lists having a given fraction of windows with *p* < 0.05. Interestingly, some cancer types had particularly high proportions of patients within the high acid adaptation similarity group; for example, 34/51 (~63%) of thyroid cancer patients (TCGA-THCA), 34/69 (~49%) of kidney renal clear cell carcinoma (TCGA-KIRC) patients, and 3/4 (75%) of pancreatic adenocarcinoma (TCGA-PAAD) patients exhibited this pattern. The insert in Figure 4C shows the numbers of patients in each cancer type in the high acid adaptation similarity group. Notably, for some cancer types only small paired tumor–ctrl. sample sets were available; hence, for these cancers, the calculated fractions are less certain.

Next, we investigated if patients in the high acid similarity group had specific clinical characteristics. We observed that patients in the high acid adaptation similarity group had a weakly significant average lower age than remaining patients (two-sided *t*-test, *p* = 0.043, means 58.99 and 62.15) (Figure 4D(*i*)). To investigate metastasis status, we obtained tumor–normal–metastasis (TNM) staging system data from the TCGA database and filtered for M0–M1 stages (presence or not of distant metastasis, 417 patients), N0–N3 stages (degree of spread to regional lymph nodes, 517 patients), and T1–T4 stages (size of the primary tumor, 619 patients) (Figure 4D(ii–iv)). We found no significant relation between acid adaptation similarity groups and metastasis or node status, but patients in the high acid adaptation similarity group were less likely to be classified as T2 (two-sided Fisher’s exact test, *p* = 0.0007, odds ratio = 0.48), and more likely to be classified as T3 (two-sided Fisher’s exact test, *p* = 0.0015, odds ratio = 1.95). This is in congruence with the notion that an acidic gene signature is more likely to correlate with a more advanced primary tumor stage than with earlier stages.

**Figure 5:**
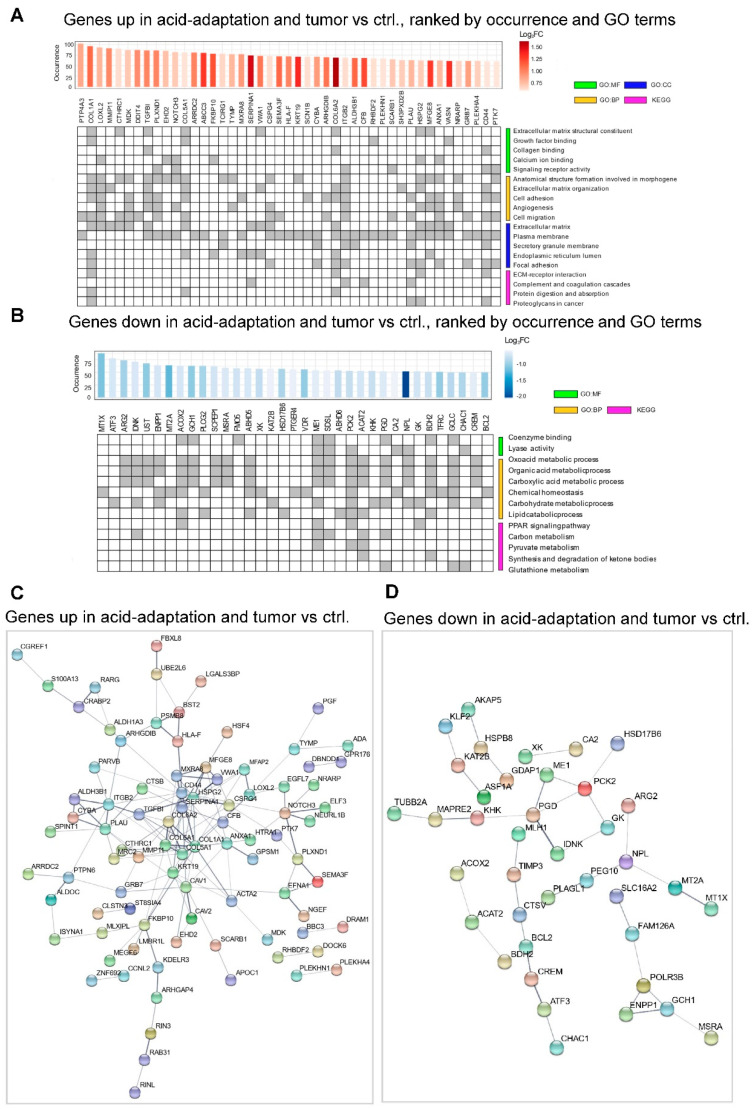
Analysis of overlapping genes between pH ranks and tumor–ctrl. ranks with high similarity. (**A**) Gene ontology (GO) and KEGG pathway analysis of genes upregulated in acid adaptation and correlated in gene expression rank in tumor–ctrl. comparisons in ≥50 patients. The upper bar plot shows the number of tumor–ctrl. vs. acid adaptation RRHO analyses in which the gene was identified as significant. Bar color indicates log_2_ expression FC in the acid adaptation experiment (pH 6.5 vs. 7.6). Gene identifiers are shown below. Rows in the bottom panel correspond to selected GO terms with high significance from three sources (BP—Biological process, MF—Molecular function, CC—Cellular Component) and pathways from the KEGG database. Columns are genes as indicated above. Grey cells indicate that a given gene is labelled with a given GO term or pathway. (**B**) Gene ontology (GO) analysis of genes downregulated in acid adaptation correlated in gene expression rank in tumor–ctrl. comparisons in ≥50 patients, arranged as in (**A**). (**C**) STRING database analysis of genes from panel (**A**). Nodes are genes. Connections indicate STRING database evidence of protein interaction. Fully unconnected genes are not shown. (**D**) STRING database analysis of genes from panel (**B**), arranged as in (**C**).

Overall, the RRHO analysis strongly indicates high similarity in acid adaptation response and tumor–ctrl. expression profiles for a subset of patients spread across many cancers, in particular for the upregulated genes. To investigate the overlap on a gene, rather than patient, basis for each tumor–ctrl. and acid adaptation RRHO analysis, we extracted the set of genes that had significant rank similarities. We then sorted these genes via how many times they were identified in the overlap sets among all 128 comparisons. Figure 5A,B show significant GO term and KEGG pathway annotation of such genes occurring in ≥50 comparisons. STRING [49] protein–protein interaction plots for the up- and downregulated genes with occurrence ≥50 are shown in Figure 5D,E. The GO/KEGG analyses show that the upregulated overlapping genes were significantly associated with ECM remodeling and cell migration and invasion, whereas the downregulated overlapping genes were generally associated with metabolic processes. STRING analysis confirmed this pattern, with upregulated overlapping genes showing a predominant clustering of genes related to ECM components and remodeling (Figure 5C). Downregulated overlapping genes were less clearly clustered, but a cluster of metabolism-related genes including *ME1*, *PCK2*, *PGD*, *GK*, *IDNK* and *KHK* was evident (Figure 5D).

Collectively, these analyses show that there is a substantial overlap between gene sets up/downregulated by acid adaptation and genes up/downregulated in tumor vs. matched normal tissue, respectively. Furthermore, they indicate that the upregulated overlapping gene sets are strongly dominated by genes involved in cell motility, invasiveness and ECM remodeling, consistent with the notion that these are key processes through which microenvironmental acidosis favors cancer development.

### 2.5. Integration of Survival Data and Rank–Rank Analysis

**Figure 6:**
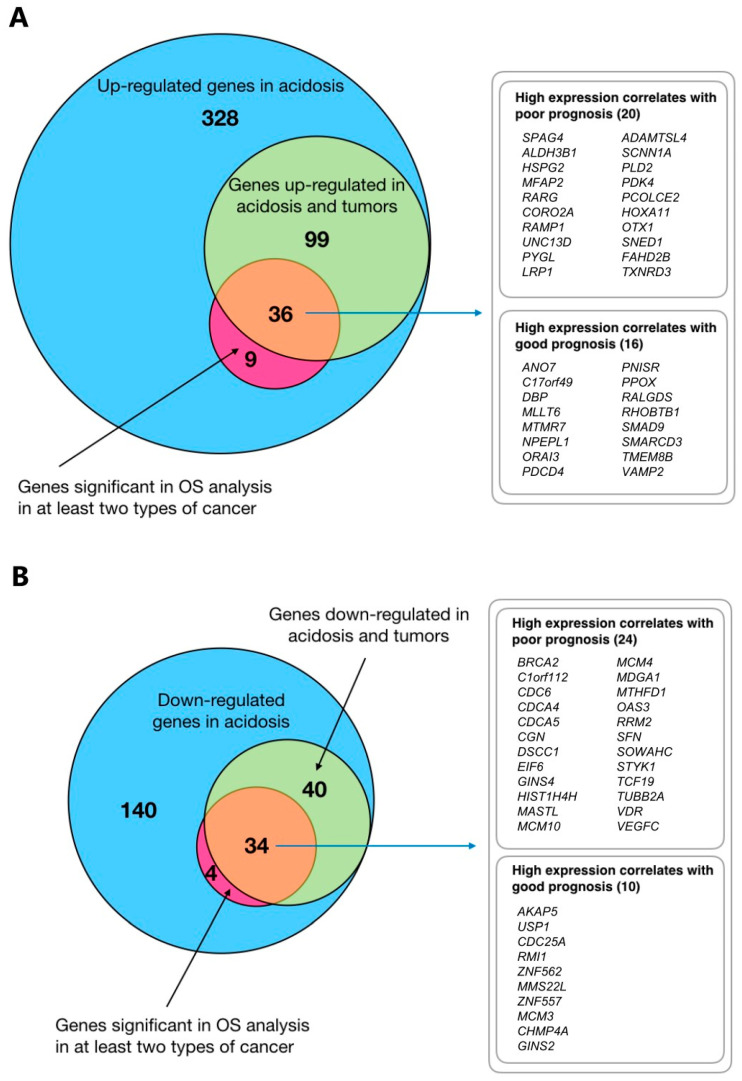
Integrative analysis. (**A**) Overlaps between upregulated genes, OS and RRHO analyses. Venn diagrams show overlaps between acid adaptation-upregulated genes (from Figure 2B), of genes significant in overall survival (OS) analysis in at least two types of cancer (based on TCGA data, Figure 4), and genes identified as having significant rank correlations between acid adaptation and tumor-control gene rank lists (RRHO analysis, Figure 4 and Figure 5). (**B**) Overlaps between downregulated genes, OS and RRHO analyses Venn diagrams arranged as in (**A**) but focused on acid adaptation-downregulated genes.

Above, we have shown that (i) the in vivo expression of many genes selected for during acid adaptation is correlated to overall survival, and (ii) for around 20% of cancer patients investigated, there is a clear correlation in ranked gene expression (tumor–ctrl. vs. acid adaptation response). Seeking to identify a set of genes that both correlated in ranked gene expression between adapted cells and tumors, and was linked to survival, we plotted the overlap of acid adaptation-up- and downregulated genes identified in the respective analyses as Venn diagrams (Figure 6). For survival-related genes, we chose a conservative approach where only genes that were significantly related to survival in at least two cancer types were included. Remarkably, the large majority of such genes overlapped with genes identified by the RRHO analysis, resulting in two consensus gene lists of 36 and 34 genes, respectively (Figure 6A,B), which could be further split based on whether their in vivo expression correlated with higher or lower patient survival.

As expected, the acid adaptation and tumor-upregulated genes that were associated with poor prognosis (Figure 6A, top list) included multiple genes related to ECM and cell motility in various cancers, such as those encoding sperm-associated antigen-4 (SPAG4) [50], microfibril-associated protein-2 (MFAP2) [51], the ADAM protease ADAMTSL4 [52], and ECM components heparan-sulfate proteoglycan-2 (HSPG2) and Procollagen C-Endopeptidase Enhancer 2 (PCOLCE2). Thioredoxin reductase-3 (TXNRD3), likely important for alleviating oxidative stress, the transcription factors HOXA11 and OTX1, as well as the ENaC subunit SCNN1A, discussed above, were also found in this gene set. The upregulated genes for which high expression correlated with good prognosis in the cancers studied (Figure 6A, lower list) included *SMAD9*, discussed above, as well as genes coding for two transport proteins, the Ca^2+^ channel ORAI3 and the TMEM16 family member ANO7, which is a lipid transporter and/or ion channel. It should, however, be noted that although *ANO7* upregulation is associated with good prognosis in breast and colon cancer (Table 1), it is in fact associated with poor prognosis in prostate cancer [53].

The acid adaptation-downregulated genes overlapping with genes downregulated in patient cancers, and for which high expression was associated with good prognosis (i.e., potential candidates for pro-tumorigenic effects of acid adaptation, Figure 6B, lower list) included *AKAP5* and *ZNF557*, described above, as well as several DNA replication- and repair-related genes, including the genes encoding the DNA repair protein MMS22L, the DNA replication licensing factor MCM3, and the replication fork complex protein GINS2. Conversely, the downregulated genes for which high expression correlates with poor prognosis (Figure 6B, top list) were dominated by other genes involved in DNA replication, repair and cell cycle control, such as *BRCA2*, *CDC6*, *CDCA4*, *CDCA5* and *EIF6*.

Collectively, these results reveal that genes regulated similarly in acid-adapted cells and in tumor tissue can be linked to both poor and good prognosis, consistent with the notion that tumor acidosis may be both a driver and a repressor of cancer progression. This indicates that while acidosis is likely to favor cancer progression through the upregulation of ECM/motility related genes, it limits it through the downregulation of DNA replication pathways.

## 3. Discussion

Acidosis is one of the key characteristics of the microenvironment of solid tumors [3,9,20,54]. Agreeing with the idea that cancer is largely an evolutionary disease driven by selection pressure, it has been proposed that chronic acidosis selects for changes in cancer cell genotype and phenotype that increase their “fitness”, and hence aggressiveness [3,55,56]. Such adaptations would potentially render the cells resistant not only to sustained acidosis, but also to other stress stimuli, such as nutrient limitations, hypoxia and chemotherapy, as well as to anti-tumor immunity [9,57,58]. Although individual genes and pathways have previously been compared between acid-adapted cells and patient tumors (e.g., [15,24,59]), a comprehensive integrative analysis comparing genes upregulated by acidosis, genes upregulated in patient tumors and their relation to patient survival is lacking. This was the aim of the work presented here.

Our analyses show that hundreds of genes are similarly regulated by chronic acidosis, in three cancer cell lines from human breast and pancreatic cancers, allowing for the identification of a characteristic gene set shared between cancer cells selected for growth in an acidic microenvironment. Both the up- and downregulated genes show significant overlaps with genes up- and downregulated in patient tumor tissue, compared to normal tissue, for subsets of patients across multiple cancers. Finally, numerous acid-regulated genes correlate with overall patient survival in at least one cancer type, and many in two or more cancers, pointing to a possible functional role. Importantly, survival correlations were both positive and negative, i.e., some genes selected for by acidosis and in patient tissue correlate with increased, and some with decreased, overall survival; furthermore, the effect on survival is often cancer-specific.

It is important to note that although the overlap between acid adaptation and tumor-specific expression was strong, the overall causality is unclear. Extracellular acidosis within tumors may causally lead to cancer aggression in vivo or potentiate an existing aggressive tumor behavior, or the correlations we observe may be caused by an unobserved factor. To detangle these questions and understand their true relevance to tumor growth and metastasis, it will be necessary to measure and manipulate extracellular acidity in tumors in vivo or in advanced in vitro models, that can mimic not only acidosis but other physicochemical, as well as cellular, components of the tumor microenvironment.

A limitation of this work is that a given gene may favor the development of one cancer type and counteract another (Tables S7 and S8), and hence correlations demonstrated here will not apply to all cancers. This notwithstanding, clear patterns were observed.

A number of specific observations deserve special attention with respect to their relation to cancer biology. Firstly, our findings are fully in line with the existing studies proposing that tumor acidosis can promote cancer invasiveness. This includes the prominent pattern of upregulation by acidosis of genes involved in ECM remodeling and invasiveness, as well as the striking extent of upregulation of ASIC1, the ENAC α subunit, and other cation channel genes, many of which have been assigned important roles in cell–cell adhesion and invasion [60,61]. Second, our analyses also support several existing studies that clearly show that adaptation to acidosis is not associated with increased growth of the cancer cells [13,15]. The strong upregulation of *TXNIP* and its paralogs *ARRD2* and *ARRD4* by chronic acidosis is consistent with previous reports of the effect of acidosis [18,24] and lactic acidosis [22] on this family of proteins, which are key negative regulators of glycolytic metabolism. In previous work, TXNIP upregulation in acidosis has been shown to involve MondoA [22,24]. The metabolic signature induced by chronic acidosis in this and previous reports is characterized by the downregulation of fermentative glycolysis and the upregulation of glutamine- and lipid-based metabolism [18,62]. In contrast to this, cancer cells are often highly glycolytic, and accordingly, frequently exhibit TXNIP downregulation [25,26,27]. Interestingly, combined inhibition of mTOR and histone deacetylases strongly upregulated TXNIP, triggering oxidative stress-induced cell death in KRAS-driven tumors [63]. This makes it tempting to speculate that TXNIP could be responsible not only for the metabolic shift away from fermentative glycolysis, but also for oxidative stress and growth restriction in acid-adapted cells.

Other mechanisms from those discussed in this work are likely to contribute to the effect of microenvironmental acidosis on cancer aggressiveness. For instance, extracellular acidosis may impact genomic instability and cause epigenetic changes in cancer cells, in line with the substantial downregulation of DNA damage response mechanisms observed in this work [9,17]. Numerous studies have established the strong inhibitory effect of acidosis on cell cycle progression [64]. After several weeks to a few months of culture in acidic medium, cancer cells attain a growth rate similar to that of normal cells grown at pH 7.4, yet not higher [13,15]. This is in itself remarkable, given the acidosis-induced downregulation of pathways associated with cell cycle progression and DNA replication observed in this study. It is, however, consistent with the notion that the acidic TME is likely to act as a brake on proliferation, and may be seen as a niche favoring cells with limited growth but high growth potential [56,65], such as cancer stem cells. Such cells would be growth-limited in an acidic environment, but could have the capacity for extremely rapid growth when encountering regions of normal pH_e_ [9]. In this regard, therapeutic strategies targeting pathways selected for by chronic acidosis, yet functionally limited by these conditions, may be particularly interesting. Similarly, otherwise highly successful strategies of limiting tumor growth by counteracting acidosis [66,67] could carry the risk of exacerbating aggressiveness if applied at a stage when pH normalization might unleash aggressive growth.

## 4. Materials and Methods

### 4.1. Cell Culture and Acid Adaptation

Human breast cancer cell lines MDA-MB-231, MCF7 and human pancreatic adenocarcinoma cell line PANC-1 were cultured in RPMI-1640 Medium (Sigma Aldrich, Catalog No. R1383, Saint Louis, MO, USA) supplemented with 10% Fetal Bovine Serum (Sigma Aldrich, Cat. No. F9665, Saint Louis, MO, USA) and 1% Penicillin/Streptomycin (Sigma Aldrich, Cat. No. P0781, Saint Louis, MO, USA). Medium pH was adapted by adjusting the HCO_3_^−^ concentration by adding the appropriate amount of NaHCO_3_, ensuring equal osmolarity by adjusting [NaCl]. Cells were adapted to pH 7.6 and 6.5 by adding the appropriately pH-adjusted medium to freshly split cells, and maintaining cells in this medium, splitting or adding fresh medium twice per week for 1 month, until approximately equal growth rates were observed.

### 4.2. RNA Isolation and RNA Sequencing

Cells were grown to 70–90% confluence in 10 cm^2^ Petri dishes. RNA isolation was performed using a Nucleospin RNA purification kit (Macherey-Nagel, Cat. # NC0707522, Düren, Germany) and following the manufacturer’s instructions. RNA library preparation and sequencing were done as a paid service by BGI (Hongkong), producing 100 bp paired-end non-stranded libraries with ~20 M reads per library, using the BGI-seq platform. Three replicate libraries were made for each cell type and condition (in total 18 libraries).

### 4.3. Analyses of RNA-Seq Data

RNA-seq reads were pseudoaligned to Gencode transcriptome release 34 and quantified using Salmon v1.1.0 with flags–validateMappings for selective alignment and −l A for automatic library type inference [68]; Trimmed Mean of M values (TMM) normalization [69] and pre-filtering for low counts were performed using calcNormFactors and filterByExpr function in edgeR package [70,71]. Voom transformation [29] was then applied with a design matrix that used pH treatment as coefficient. To perform differential expression analyses in shared response to pH, inter-subject correlation within three cell lines was performed by duplicateCorrelation function, and was put into the linear model fit using Limma R package [72]. Differential expression, given the above settings, was defined as Benjamini–Hochberg FDR < 0.05 and an absolute log_2_ FC > 0.5. Validation of key up/downregulated genes by qPCR and immunoblotting is provided in a manuscript in preparation and confirms the RNA-seq results.

### 4.4. GO Enrichment Analysis and Gene Set Enrichment Analysis (GSEA)

Enrichment tests for GO-terms were performed on differentially expressed genes sets as defined above using gProfiler [73]. The background gene set was set to all expressed genes in the experiment. Significance threshold was set to Benjamini–Hochberg FDR < 0.05. The Gene Set Enrichment Analysis (GSEA) pre-ranked function was performed on the fold change ranked acid adaptation gene list using the Broad Institute GSEA java tool version 4.0.3. C6: Oncogenic signatures database v7.1, gene sets WU_CELL_MIGRATION (M2001) and ANASTASSIOU_MULTICANCER_INVASIVENESS_SIGNATURE (M2572) were used and the number of permutations was set to 1000 [74].

### 4.5. RRHO Analysis

First, using Limma and Voom as above, we ranked all expressed genes from the RNA-seq experiments by their shared acid adaptation response across cells (log_2_ fold change pH 6.5 vs. pH 7.4). We will refer to this ranked gene list as the ‘acid adaptation ranked gene list’. To be able to compare this to human data, we extracted processed patient RNA-seq data from Cancer Genome Atlas (TCGA) [75] using the University of California Santa Cruz Xena portal [76]. For every patient and cancer where expression data from paired tumor and control tissue were available, and for each such case, we ranked genes according to their log2 fold change (tumor vs. control (ctrl.) tissue). Next, we compared the acid adaptation ranked list to each ranked patient gene lists in a pairwise fashion, using the Rank–Rank Hypergeometric Overlap (RRHO) method [77], as implemented in R [78]. For each comparison, we obtained heat maps showing rank–rank correlations, colored by log-transformed hypergeometric *P*-value of local rank overlaps between sets, and the identifiers of genes in each overlap.

### 4.6. Patient Survival Analysis

TCGA cancer patient data was downloaded from the University of California Santa Cruz Xena portal [76]. The following datasets containing gene expression RNAseq counts, survival data and phenotype information were analyzed: GDC TCGA Pancreatic Cancer (PAAD), GDC TCGA Breast Cancer (BRCA), GDC TCGA Lung Adenocarcinoma (LUAD), GDC TCGA Glioblastoma (GBM), GDC TCGA Colon Cancer (COAD), GDC TCGA Ovarian Cancer (OV), GDC TCGA Thyroid Cancer (THCA) and GDC TCGA Stomach Cancer (STAD).

Kaplan–Meier analysis was performed to estimate the overall survival of patients. Patients were categorized into high and low gene expression groups according to the cut-off value determined by median gene expression. *p*-values for significance of difference between the two groups were calculated using the log-rank test. Statistical analyses were performed using R’s “survival” and “survminer” packages. *p*-values < 0.05 were considered statistically significant.

### 4.7. Data Availability

RNA-sequencing data from this study has been deposited in GEO database under accession number GSE152345.

## 5. Conclusions

We have defined a shared acid adaptation expression response across three cancer cell models, dominated by ECM remodeling, metabolic rewiring and altered cell cycle regulation. Many genes which are upregulated by acid adaption are significantly correlated to patient survival, and there are clear correlations between acid adaptation expression response and expression change between normal and tumor tissues for a large subset of cancer patients. Importantly, our analyses reveal that genes whose expressions are changed by acid adaptation are linked both to increased and decreased overall patient survival. Thus, in conclusion, our data support the notion that adaptation to microenvironmental acidosis can drive the selection of highly invasive cancer cells and ECM remodeling in human patient tumors. However, consistent with existing experimental data, they also show that acid adaptation exerts inherently growth-limiting effects, which could be important for cancer stem cell maintenance but may per se limit full-blown cancer development, until selected against or otherwise eliminated. Future studies should address what dictates the balance between these effects, and hence the net effects of acid adaptation on cancer development in vivo.

## Figures and Tables

**Table 1:** Acid adaptation-upregulated genes significant in OS analysis in at least two types of cancers.

				Types of Cancer								
Gene Name	Log_2_FC	FDR	Correlation with Poor OS	PAAD	BRCA	LUAD	GBM	COAD	OV	THCA	STAD	Molecular Function/Biological Process (UniProt)
4 OVERLAPS												
*SMAD9*	1.1723	0.0091	Low expression									DNA-binding, transcription regulation
3 OVERLAPS												
*LGR4*	0.7849	0.0127	High expression									Differentiation, immunity
*RARG*	0.6840	0.0117	High expression									DNA-binding, transcription regulation
*PNISR*	0.5890	0.0258	Low expression									RNA-binding
*PCOLCE2*	0.5538	0.0408	High expression									Collagen biosynthesis
*RALGDS*	0.5128	0.0249	Low expression									GTPase regulator, signal transduction
2 OVERLAPS												
*SCNN1A*	2.0490	0.0100	High expression									Ion transport
*PLA2R1*	1.6361	0.0249	High expression									Endocytosis
*PDK4*	1.6088	0.0351	High expression									Ser/Thr protein kinase, metabolism
*SNED1*	1.5574	0.0302	High expression									Cell-matrix adhesion
*ADAMTSL4*	1.5083	0.0176	High expression									Peptidase, apoptosis
*RAMP1*	1.4724	0.0169	High expression									Transport, angiogenesis
*MFAP2*	1.3770	0.0154	High expression									Embryonic morphogenesis
*HSPG2*	1.2833	0.0073	High expression									Angiogenesis, morphogenesis,
*GNG7*	1.2239	0.0142	Low expression									GTPase activity
*HOXA11*	1.1569	0.0346	High expression									DNA-binding, transcription regulation
*ALDH3B1*	1.1061	0.0081	High expression									Oxidoreductase, metabolism
*CORO2A*	1.0708	0.0321	High expression									Actin-binding, signal transduction
*LRP1*	1.0403	0.0380	High expression									Endocytosis
*TMEM8B*	0.9897	0.0045	Low expression									Cell adhesion, growth regulation
*ZNF710-AS1*	0.9413	0.0330	Low expression									lncRNA
*PYGL*	0.8856	0.0251	High expression									Transferase, metabolism
*TMED7-TICAM2*	0.8634	0.0325	High expression									Golgi organization, protein transport
*NPEPL1*	0.8764	0.0117	Low expression									Aminopeptidase
*SMARCD3*	0.8743	0.0058	Low expression									Chromatin regulator, neurogenesis
*RHOBTB1*	0.8684	0.0234	Low expression									GTPase activity, actin organization
*UNC13D*	0.8587	0.0368	High expression									Exocytosis
*DBP*	0.8034	0.0249	Low expression									DNA-binding, transcription regulation
*PDCD4*	0.7330	0.0077	Low expression									RNA-binding, apoptosis
*MTMR7*	0.7273	0.0329	Low expression									Phosphatase
*OTX1*	0.6797	0.0091	High expression									DNA-binding, morphogenesis
*TXNRD3*	0.6584	0.0125	High expression									Differentiation, electron transport
*PPOX*	0.6399	0.0279	Low expression									Oxidoreductase, heme biosynthesis
*SPAG4*	0.6392	0.0404	High expression									Differentiation, spermatogenesis
*ANO7*	0.6260	0.0329	Low expression									Lipid transport
*FAHD2B*	0.6164	0.0069	High expression									Hydrolase
*ORAI3*	0.6063	0.0224	Low expression									Calcium channel
*NOP53*	0.5938	0.0156	High expression									Host-virus interaction, ribosome biogenesis
*C17orf49*	0.5910	0.0081	Low expression									Chromatin regulator, DNA-binding
*VAMP2*	0.5901	0.0073	Low expression									Exocytosis, protein transport
*ZNF487*	0.5872	0.0377	Low expression									DNA-binding, transcription regulation
*TDRD3*	0.5832	0.0058	Low expression									Chromatin regulator, RNA-binding
*FRG1HP*	0.5302	0.0376	Low expression									Pseudogene
*PLD2*	0.5226	0.0306	High expression									Hydrolase, lipid metabolism, motility
*MLLT6*	0.5118	0.0038	Low expression									Histone-binding, metal ion-binding

PAAD—pancreatic cancer (dark blue), BRCA—breast cancer–luminal B (pink), LUAD—lung cancer–adenocarcinoma (turquoise), GBM—glioblastoma (orange), COAD—colon cancer (red), OV—ovarian cancer (purple), THCA—thyroid cancer (grey), STAD—stomach cancer (green). Molecular function and/or biological processes according to UniProt database [48].

**Table 2:** Acid adaptation-upregulated genes significant in OS analysis in at least two types of cancers.

				Types of Cancer								
Gene Name	Log_2_FC	FDR	Correlation with Poor OS	PAAD	BRCA	LUAD	GBM	COAD	OV	THCA	STAD	Molecular Function/Biological Process (UniProt)
5 OVERLAPS												
*ZNF557*	−0.5999	0.0069	Low expression									DNA-binding, transcription factor, metal on binding
3 OVERLAPS												
*MDGA1*	−1.2003	0.0153	High expression									Differentiation, neurogenesis
*VEGFC*	−0.8963	0.0345	High expression									Growth factor, angiogenesis, differentiation
*MCM10*	−0.8486	0.0249	High expression									DNA-binding, DNA replication, DNA damage
*CTBP1-DT*	−0.5490	0.0098	Low expression									Oxidoreductase, differentiation, transcription
*CHMP4A*	−0.5182	0.0247	Low expression									Protein transport
2 OVERLAPS												
*CGN*	−1.5661	0.0112	High expression									Tight junction regulation
*AKAP5*	−1.4671	0.0408	Low expression									Calmodulin-binding
*HIST1H4H*	−1.3143	0.0276	High expression									DNA-binding
*STYK1*	−1.0844	0.0290	High expression									Protein tyrosine kinase
*CDC25A*	−1.0023	0.0182	Low expression									Protein phosphatase, cell cycle, cell division
*RRM2*	−0.9925	0.0325	High expression									Oxidoreductase, DNA replication
*VDR*	−0.9901	0.0131	High expression									Vitamin D3 receptor, DNA-binding, transcription
*CDC6*	−0.8805	0.0110	High expression									Cell cycle, cell division, DNA replication
*GINS4*	−0.8574	0.0376	High expression									DNA replication
*AC019069.1*	−0.8118	0.0467	High expression									lncRNA
*TCF19*	−0.7835	0.0296	High expression									DNA-binding, transcription factor
*GINS2*	−0.7784	0.0182	Low expression									DNA replication
*SOWAHC*	−0.7653	0.0201	High expression									Ankyrin repeat domain-containing protein
*BRCA2*	−0.7332	0.0375	High expression									DNA-binding, DNA recombination, DNA damage
*AC092279.1*	−0.6853	0.0203	Low expression									lncRNA
*CDCA5*	−0.6652	0.0425	High expression									Cell cycle, cell division
*TUBB2A*	−0.6559	0.0360	High expression									GTP-binding, microtubule cytoskeleton organization
*OAS3*	−0.6518	0.0117	High expression									RNA-binding, antiviral defense, immunity
*SFN*	−0.6382	0.0158	High expression									DNA damage response
*MTHFD1*	−0.6333	0.0107	High expression									Embryonic morphogenesis
*ZNF562*	−0.6041	0.0069	Low expression									DNA-binding, transcription factor, metal ion binding
*MASTL*	−0.6008	0.0482	High expression									Ser/Thr protein kinase, cell division
*CDCA4*	−0.5907	0.0236	High expression									Cell division
*MMS22L*	−0.5725	0.0418	Low expression									DNA repair
*RMI1*	−0.5661	0.0443	Low expression									DNA replication
*C1orf112*	−0.5581	0.0415	High expression									Uncharacterized protein C1orf112
*MIR4435-2HG*	−0.5537	0.0105	High expression									lncRNA
*MCM4*	−0.5328	0.0482	High expression									DNA-binding, cell cycle, DNA replication
*DSCC1*	−0.5268	0.0492	High expression									DNA-binding, cell cycle, DNA replication
*USP1*	−0.5192	0.0330	Low expression									Protease, DNA repair
*MCM3*	−0.5129	0.0471	Low expression									Transferase, mRNA transport, immunity
*EIF6*	−0.5008	0.0100	High expression									Ribosome biogenesis, protein synthesis

PAAD—pancreatic cancer (dark blue), BRCA—breast cancer–luminal B (pink), LUAD—lung cancer–adenocarcinoma (turquoise), GBM—glioblastoma (orange), COAD—colon cancer (red), OV—ovarian cancer (purple), THCA—thyroid cancer (grey), STAD—stomach cancer (green). Molecular function and/or biological processes according to UniProt database [48].

## Supplementary Materials

The following are available online at https://www.mdpi.com/2072-6694/12/8/2183/s1, Figures S1–S16: Kaplan–Meier overall survival analysis of cancer patients stratified by acidosis up- and downregulated gene expression levels, Table S1: Upregulated genes in pH 6.5 vs. pH 7.6, Table S2: Downregulated genes in pH 6.5 vs. pH 7.6, Table S3: GO and KEGG analysis of upregulated genes in pH 6.5 vs. pH 7.6, Table S4: GO and KEGG analysis of downregulated genes in pH 6.5 vs. pH 7.6, Table S5: Acidosis-upregulated genes significant in overall survival analysis in one type of cancer, Table S6: Acidosis-downregulated genes significant in overall survival analysis in one type of cancer; Table S7: Acidosis-upregulated genes, which showed contradictory survival analysis results across cancer types, Table S8: Acid adaptation-downregulated genes, which showed contradictory survival analysis results across cancer types.
