# Supplementary material for: Cancer Cell Acid Adaptation Gene Expression Response Is Correlated to Tumor-Specific Tissue Expression Profiles and Patient Survival
Source: Cancers (Basel). 2020 Aug 5;12(8):2183. doi: 10.3390/cancers12082183 (PMC7463722; doi:10.3390/cancers12082183)
Supplement: Supplementary file 1 [file cancers-12-02183-s001.zip › cancers-855025-SUPPLE-XML/cancers-855025-supple-proof/cancers-855025-suppl-proof-corrected.docx]

Supplementary Materials: Cancer Cell Acid Adaptation Gene Expression Response Is Correlated to Tumor-Specific Tissue Expression Profiles and Patient Survival

Jiayi Yao, Dominika Czaplinska, Renata Ialchina, Julie Schnipper, Bin Liu, Albin Sandelin and Stine Falsig Pedersen

**Figures S1-S16: Note that only plots with p < 0.05 are shown**

**Figure S1.** Kaplan-Meier overall survival analysis of pancreatic cancer patients stratified by acidosis up-regulated gene expression levels. Patients were categorized into high and low gene expression groups according to the cut-off value determined by median gene expression. OS and gene expression data obtained from the Cancer Genome Atlas (TCGA) database. P-values for significance of difference between high and low expression were calculated using the log-rank test. Time unit: day.

**Figure S2.** Kaplan-Meier overall survival analysis of pancreatic cancer patients stratified by acidosis down-regulated gene expression levels. Patients were categorized into high and low gene expression groups according to the cut-off value determined by median gene expression. OS and gene expression data obtained from the Cancer Genome Atlas (TCGA) database. P-values for significance of difference between high and low expression were calculated using the log-rank test. Time unit: day.

**Figure S3.** Kaplan-Meier overall survival analysis of breast cancer (luminal B) patients stratified by acidosis up-regulated gene expression levels. Patients were categorized into high and low gene expression groups according to the cut-off value determined by median gene expression. OS and gene expression data obtained from the Cancer Genome Atlas (TCGA) database. P-values for significance of difference between high and low expression were calculated using the log-rank test. Time unit: day.

**Figure S4.** Kaplan-Meier overall survival analysis of breast cancer (luminal B) patients stratified by acidosis down-regulated gene expression levels. Patients were categorized into high and low gene expression groups according to the cut-off value determined by median gene expression. OS and gene expression data obtained from the Cancer Genome Atlas (TCGA) database. P-values for significance of difference between high and low expression were calculated using the log-rank test. Time unit: day.

**Figure S5.** Kaplan-Meier overall survival analysis of lung cancer (adenocarcinoma) patients stratified by acidosis up-regulated gene expression levels. Patients were categorized into high and low gene expression groups according to the cut-off value determined by median gene expression. OS and gene expression data obtained from the Cancer Genome Atlas (TCGA) database. P-values for significance of difference between high and low expression were calculated using the log-rank test. Time unit: day.

**Figure S6.** Kaplan-Meier overall survival analysis of lung cancer (adenocarcinoma) patients stratified by acidosis down-regulated gene expression levels. Patients were categorized into high and low gene expression groups according to the cut-off value determined by median gene expression. OS and gene expression data obtained from the Cancer Genome Atlas (TCGA) database. P-values for significance of difference between high and low expression were calculated using the log-rank test. Time unit: day.

**Figure S7.** Kaplan-Meier overall survival analysis of glioblastoma patients stratified by acidosis up-regulated gene expression levels. Patients were categorized into high and low gene expression groups according to the cut-off value determined by median gene expression. OS and gene expression data obtained from the Cancer Genome Atlas (TCGA) database. P-values for significance of difference between high and low expression were calculated using the log-rank test. Time unit: day.

**Figure S8.** Kaplan-Meier overall survival analysis of glioblastoma patients stratified by acidosis down-regulated gene expression levels. Patients were categorized into high and low gene expression groups according to the cut-off value determined by median gene expression. OS and gene expression data obtained from the Cancer Genome Atlas (TCGA) database. P-values for significance of difference between high and low expression were calculated using the log-rank test. Time unit: day.

**Figure S9.** Kaplan-Meier overall survival analysis of colon cancer patients stratified by acidosis up-regulated gene expression levels. Patients were categorized into high and low gene expression groups according to the cut-off value determined by median gene expression. OS and gene expression data obtained from the Cancer Genome Atlas (TCGA) database. P-values for significance of difference between high and low expression were calculated using the log-rank test. Time unit: day.

**Figure S10.** Kaplan-Meier overall survival analysis of colon cancer patients stratified by acidosis down-regulated gene expression levels. Patients were categorized into high and low gene expression groups according to the cut-off value determined by median gene expression. OS and gene expression data obtained from the Cancer Genome Atlas (TCGA) database. P-values for significance of difference between high and low expression were calculated using the log-rank test. Time unit: day.

**Figure S11.** Kaplan-Meier overall survival analysis of ovarian cancer patients stratified by acidosis up-regulated gene expression levels. Patients were categorized into high and low gene expression groups according to the cut-off value determined by median gene expression. OS and gene expression data obtained from the Cancer Genome Atlas (TCGA) database. P-values for significance of difference between high and low expression were calculated using the log-rank test. Time unit: day.

**Figure S12.** Kaplan-Meier overall survival analysis of ovarian cancer patients stratified by acidosis down-regulated gene expression levels. Patients were categorized into high and low gene expression groups according to the cut-off value determined by median gene expression. OS and gene expression data obtained from the Cancer Genome Atlas (TCGA) database. P-values for significance of difference between high and low expression were calculated using the log-rank test. Time unit: day.

**Figure S13.** Kaplan-Meier overall survival analysis of thyroid cancer patients stratified by acidosis up-regulated gene expression levels. Patients were categorized into high and low gene expression groups according to the cut-off value determined by median gene expression. OS and gene expression data obtained from the Cancer Genome Atlas (TCGA) database. P-values for significance of difference between high and low expression were calculated using the log-rank test. Time unit: day.

**Figure S14.** Kaplan-Meier overall survival analysis of thyroid cancer patients stratified by acidosis down-regulated gene expression levels. Patients were categorized into high and low gene expression groups according to the cut-off value determined by median gene expression. OS and gene expression data obtained from the Cancer Genome Atlas (TCGA) database. P-values for significance of difference between high and low expression were calculated using the log-rank test. Time unit: day.

**Figure S15.** Kaplan-Meier overall survival analysis of stomach cancer patients stratified by acidosis up-regulated gene expression levels. Patients were categorized into high and low gene expression groups according to the cut-off value determined by median gene expression. OS and gene expression data obtained from the Cancer Genome Atlas (TCGA) database. P-values for significance of difference between high and low expression were calculated using the log-rank test. Time unit: day.

**Figure S16.** Kaplan-Meier overall survival analysis of stomach cancer patients stratified by acidosis down-regulated gene expression levels. Patients were categorized into high and low gene expression groups according to the cut-off value determined by median gene expression. OS and gene expression data obtained from the Cancer Genome Atlas (TCGA) database. P-values for significance of difference between high and low expression were calculated using the log-rank test. Time unit: day.

**Table S1.** Up-regulated genes in pH 6.5 vs. pH 7.6. Up regulation is defined as false discovery rate (FDR) < 0.05 and log2fc > 0.5. Genes are ordered by FDR.

**Table S2.** Down-regulated genes in pH 6.5 vs pH 7.6. Down regulation is defined as false discovery rate (FDR) < 0.05 and log2fc < −0.5. Genes are ordered by FDR.

**Table S3.** GO and KEGG analysis of up-regulated genes in pH 6.5 vs. pH 7.6. GO terms are from three categories (BP: Biological process, MF: Molecular function, CC: Cellular Component) and pathways from the KEGG (Kyoto Encyclopedia of Genes and Genomes) database, ordered by FDR.

**Table S4.** GO and KEGG analysis of down-regulated genes in pH 6.5 vs. pH 7.6. GO terms are from three categories (BP: Biological process, MF: Molecular function, CC: Cellular Component) and pathways from the KEGG (Kyoto Encyclopedia of Genes and Genomes) database, ordered by FDR.

**Table S5.** Acidosis up-regulated genes significant in overall survival analysis in one type of cancer. Evaluation of the association of acidosis up-regulated genes with overall survival (OS). OS data obtained from the Cancer Genome Atlas (TCGA) database. PAAD: pancreatic cancer, BRCA: breast cancer (luminal B), LUAD: lung cancer (adenocarcinoma), GBM: glioblastoma, COAD: colon cancer, OV: ovarian cancer, THCA: thyroid cancer, STAD: stomach cancer.

**Table S6.** Acidosis down-regulated genes significant in overall survival analysis in one type of cancer. Evaluation of the association of acidosis down-regulated genes with overall survival (OS). OS data obtained from the Cancer Genome Atlas (TCGA) database. PAAD: pancreatic cancer, BRCA-breast cancer (luminal B), LUAD: lung cancer (adenocarcinoma), GBM: glioblastoma, COAD: colon cancer, OV: ovarian cancer, THCA: thyroid cancer, STAD: stomach cancer.

**Table S7.** Acidosis up-regulated genes, which showed contradictory survival analysis results across cancer types. Evaluation of the association of acidosis up-regulated genes with overall survival (OS). OS data obtained from the Cancer Genome Atlas (TCGA) database. PAAD: pancreatic cancer, BRCA: breast cancer (luminal B), LUAD: lung cancer (adenocarcinoma), GBM: glioblastoma, COAD: colon cancer, OV: ovarian cancer, THCA: thyroid cancer, STAD: stomach cancer.

**Table S8.** Acidosis down-regulated genes, which showed contradictory survival analysis results across cancer types. Evaluation of the association of acidosis down-regulated genes with overall survival (OS). OS data obtained from the Cancer Genome Atlas (TCGA) database. PAAD: pancreatic cancer, BRCA: breast cancer (luminal B), LUAD: lung cancer (adenocarcinoma), GBM: glioblastoma, COAD: colon cancer, OV: ovarian cancer, THCA: thyroid cancer, STAD: stomach cancer.

| 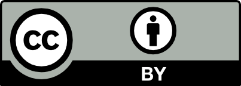 | © 2020 by the authors. Licensee MDPI, Basel, Switzerland. This article is an open access article distributed under the terms and conditions of the Creative Commons Attribution (CC BY) license (http://creativecommons.org/licenses/by/4.0/). |
| --- | --- |
