# Supplementary material for: Cancer Cell Acid Adaptation Gene Expression Response Is Correlated to Tumor-Specific Tissue Expression Profiles and Patient Survival
Source: Cancers (Basel). 2020 Aug 5;12(8):2183. doi: 10.3390/cancers12082183 (PMC7463722; doi:10.3390/cancers12082183)

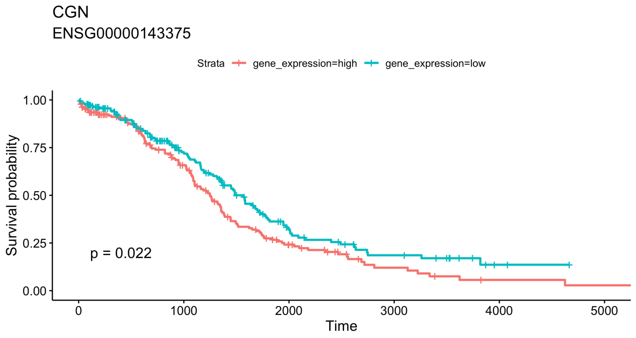
CGN
ENSG00000143375
Strata gene_expression=high gene_expression=low
Survival probability
p = 0.022
Time

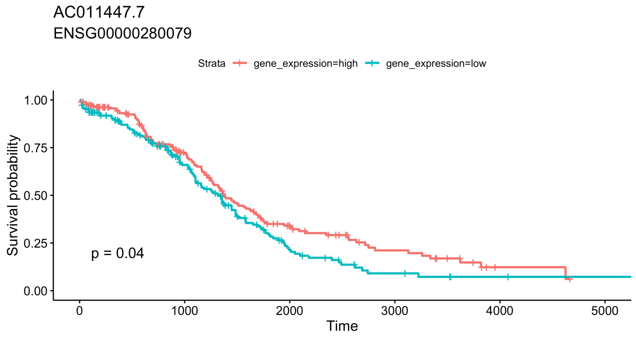
AC011447.7
ENSG00000280079
Strata gene_expression=high gene_expression=low
Survival probability
p = 0.04
Time

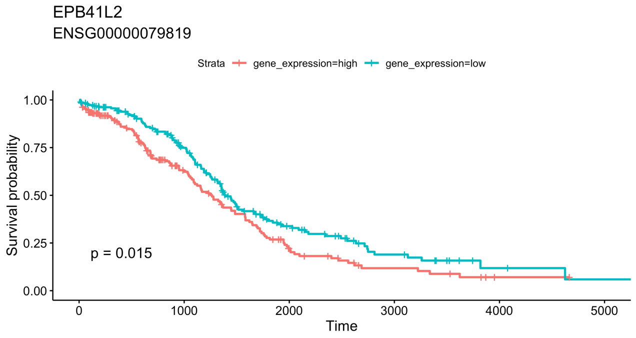
EPB41L2
ENSG00000079819
Strata gene_expression=high gene_expression=low
Survival probability
p = 0.015
Time

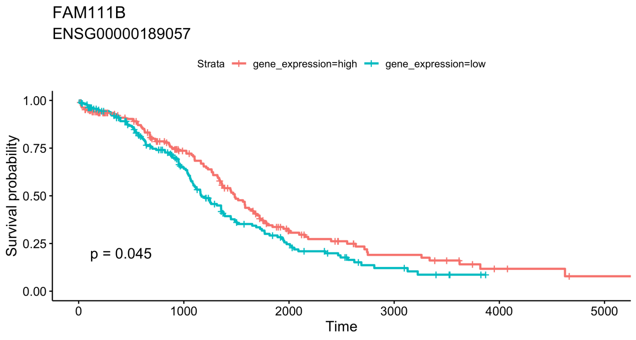
FAM111B
ENSG00000189057
Strata gene_expression=high gene_expression=low
Survival probability
p = 0.045
Time

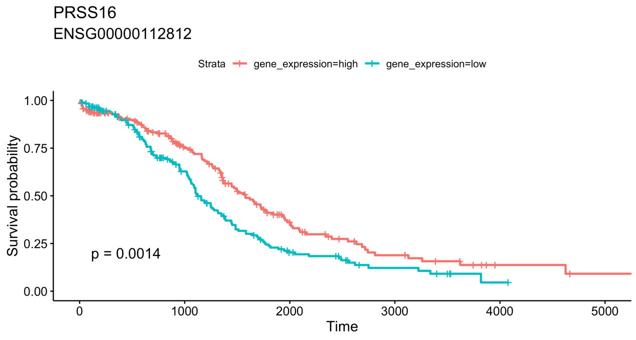
PRSS16
ENSG00000112812
Strata gene_expression=high gene_expression=low
Survival probability
p = 0.0014
Time

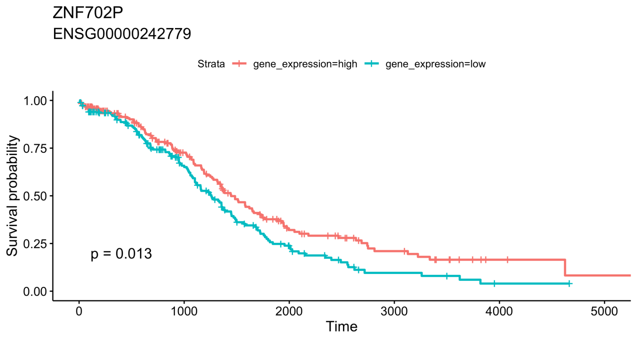
ZNF702P
ENSG00000242779
Strata gene_expression=high gene_expression=low
Survival probability
p = 0.013
Time

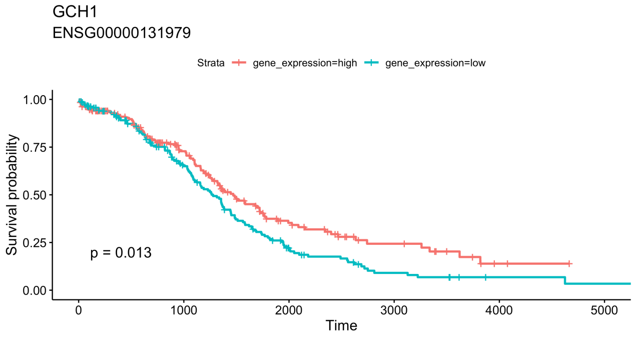
GCH1
ENSG00000131979
Strata gene_expression=high gene_expression=low
Survival probability
p = 0.013
Time

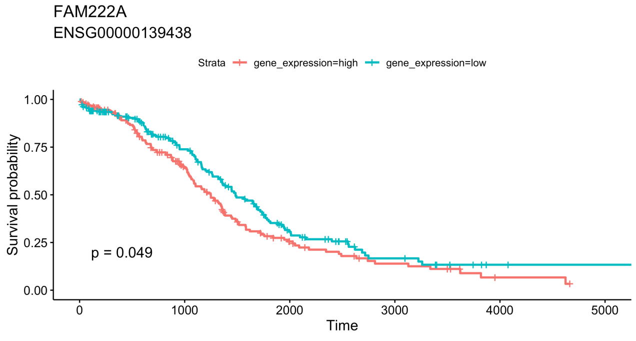
FAM222A
ENSG00000139438
Strata gene_expression=high gene_expression=low
Survival probability
p = 0.049
Time

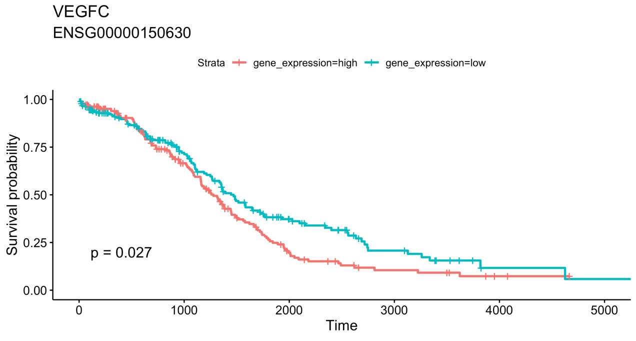
VEGFC
ENSG00000150630
Strata gene_expression=high gene_expression=low
Survival probability
p = 0.027
Time

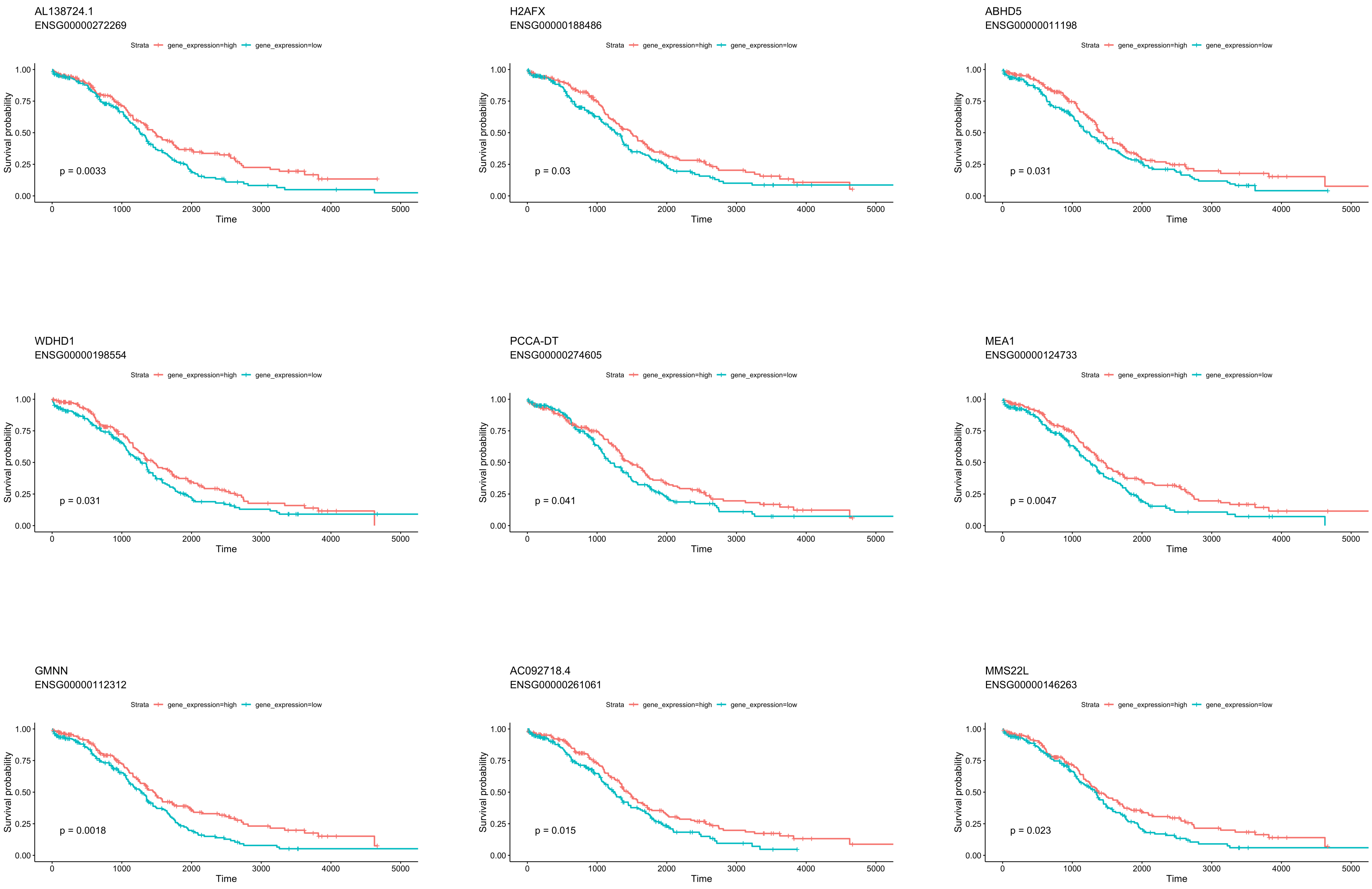
AL138724.1
ENSG00000272269
Strata gene_expression=high gene_expression=low
Survival probability
p = 0.0033
Time
H2AFX
ENSG00000188486
Strata gene_expression=high gene_expression=low
Survival probability
p = 0.03
Time
ABHD5
ENSG00000011198
Strata gene_expression=high gene_expression=low
Survival probability
p = 0.031
Time
WDHD1
ENSG00000198554
Strata gene_expression=high gene_expression=low
Survival probability
p = 0.031
Time
PCCA-DT
ENSG00000274605
Strata gene_expression=high gene_expression=low
Survival probability
p = 0.041
Time
MEA1
ENSG00000124733
Strata gene_expression=high gene_expression=low
Survival probability
p = 0.0047
Time
GMNN
ENSG00000112312
Strata gene_expression=high gene_expression=low
Survival probability
p = 0.0018
Time
AC092718.4
ENSG00000261061
Strata gene_expression=high gene_expression=low
Survival probability
p = 0.015
Time
MMS22L
ENSG00000146263
Strata gene_expression=high gene_expression=low
Survival probability
p = 0.023
Time

ORC6
ENSG00000091651

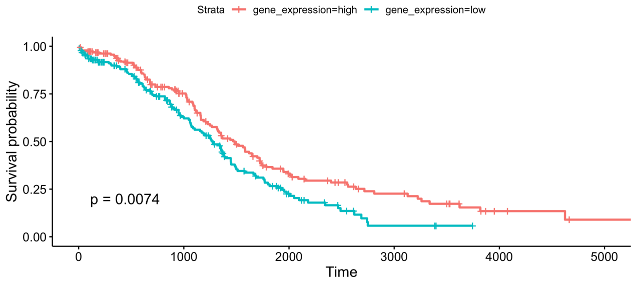

CTBP1-DT
ENSG00000196810

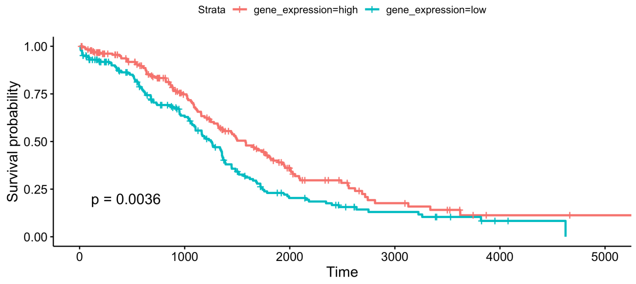

ZNF239
ENSG00000196793

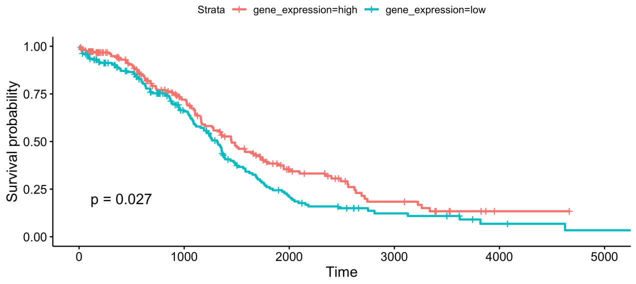

AP1S2
ENSG00000182287

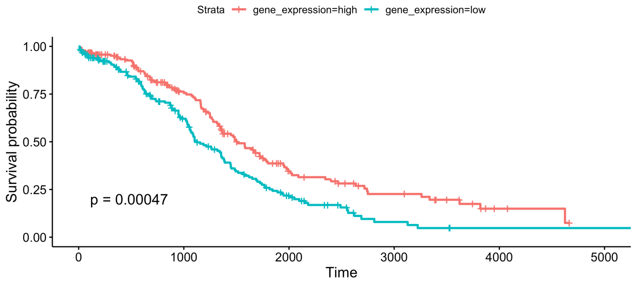

MCM3
ENSG00000112118

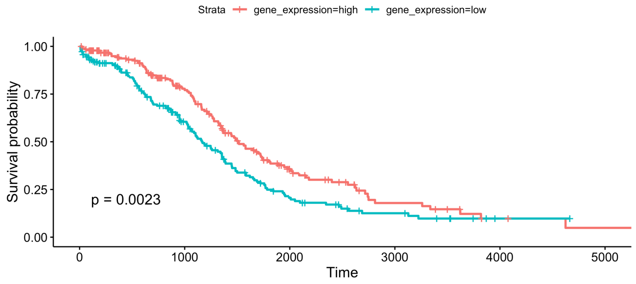

Supplement: Supplementary file 1 [file cancers-12-02183-s001.zip › cancers-855025-SUPPLE-XML/cancers-855025-supple-proof/Suppl_Fig12_ovarian_DOWNregulated_genes.pdf]
