# Supplementary figures and images for: Cancer Cell Acid Adaptation Gene Expression Response Is Correlated to Tumor-Specific Tissue Expression Profiles and Patient Survival
Source: Cancers (Basel). 2020 Aug 5;12(8):2183. doi: 10.3390/cancers12082183 (PMC7463722; doi:10.3390/cancers12082183)

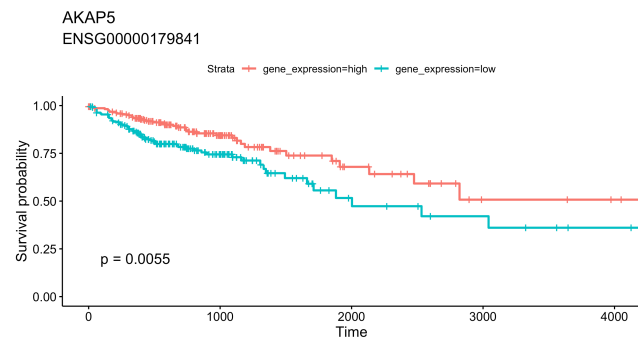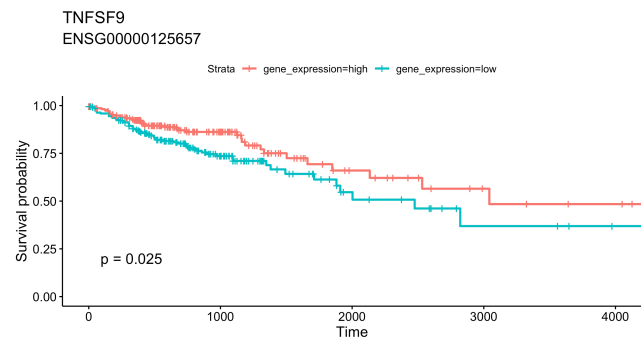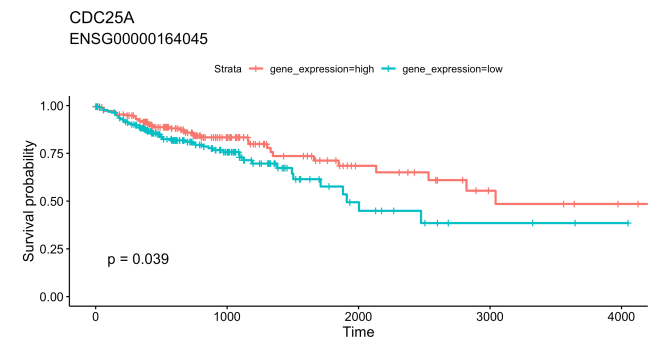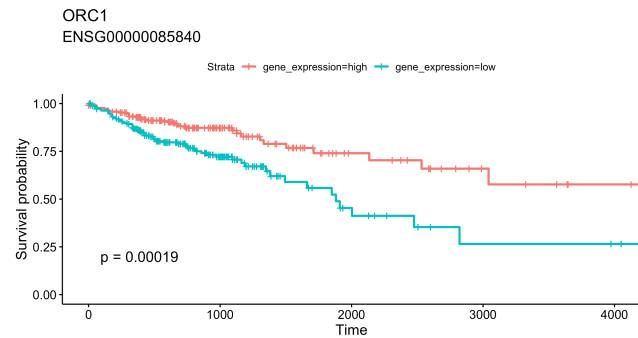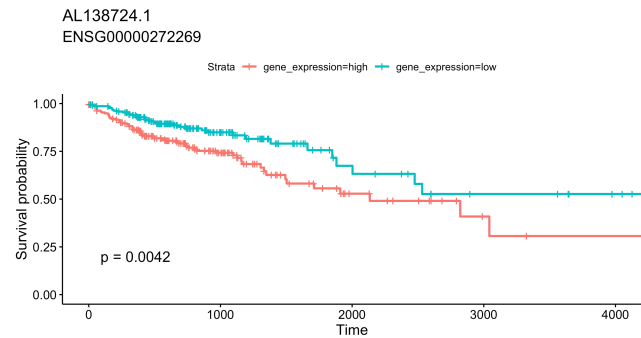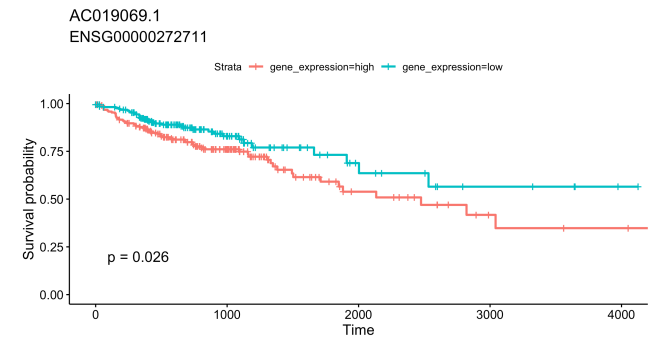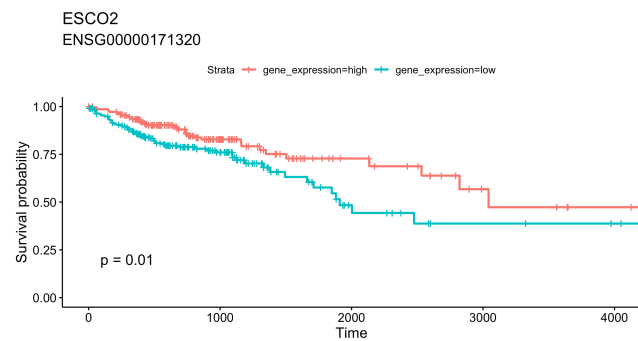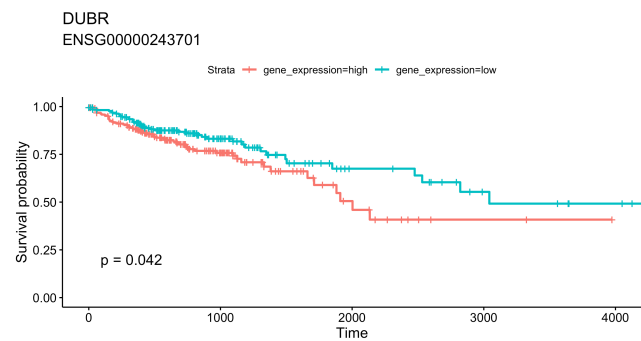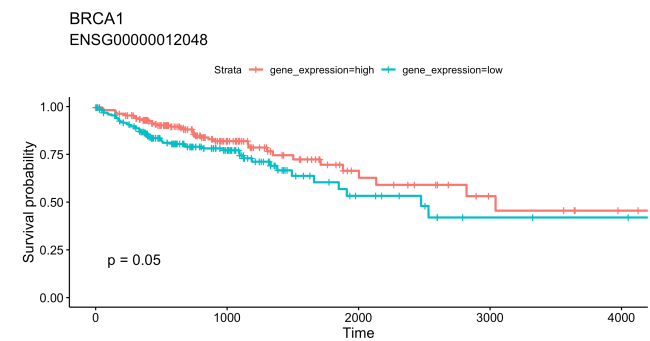

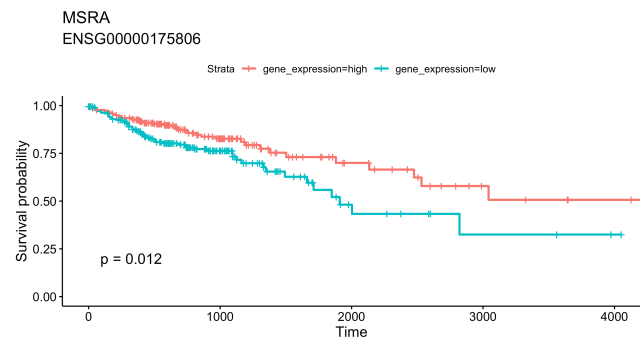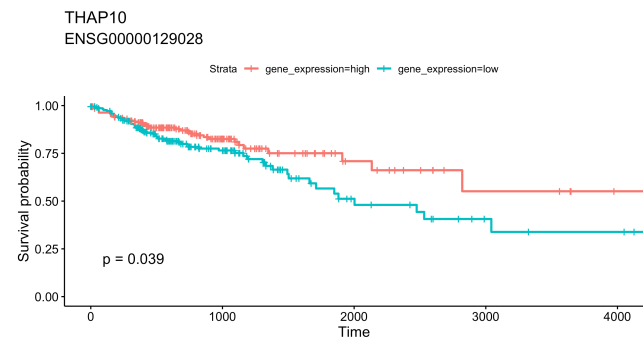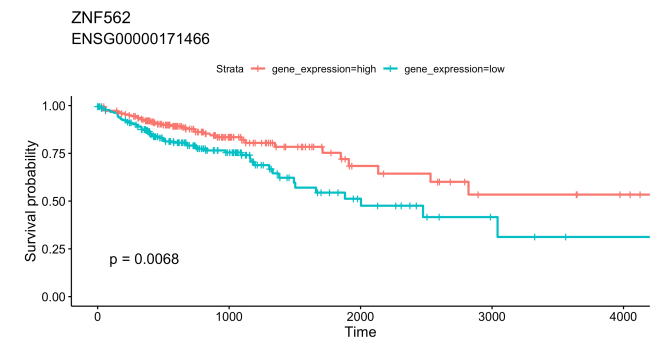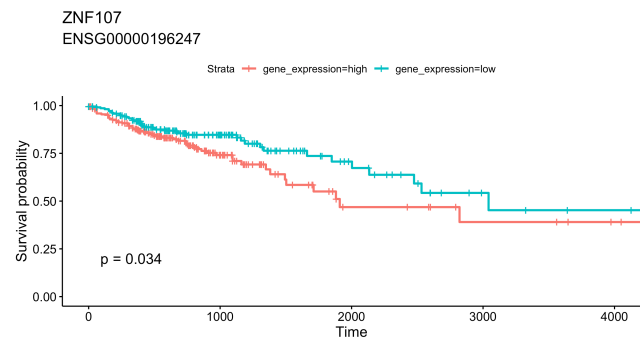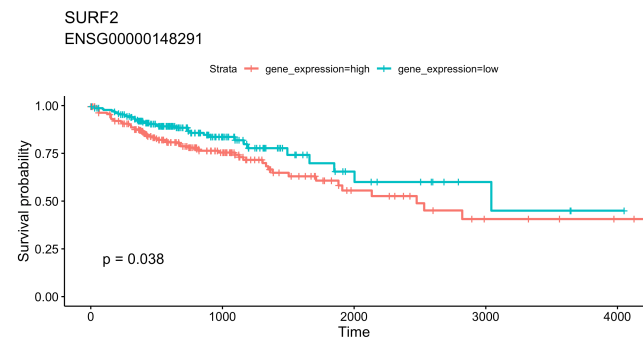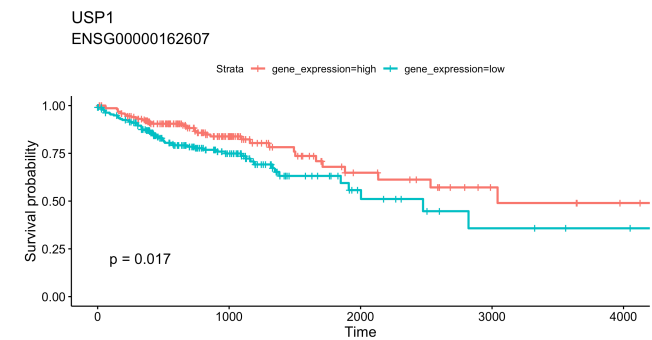

Supplement: Supplementary file 1 [file cancers-12-02183-s001.zip › cancers-855025-SUPPLE-XML/cancers-855025-supple-proof/Suppl_Fig10_colon_DOWNregulated_genes.pdf]

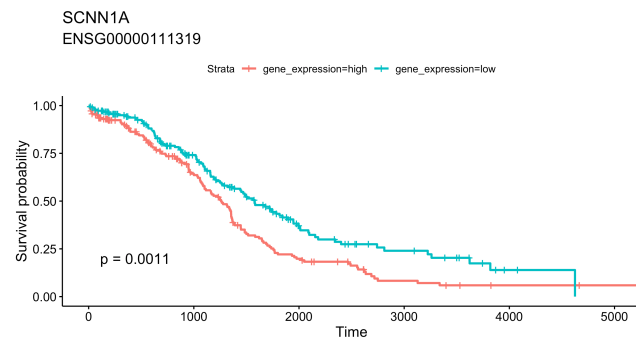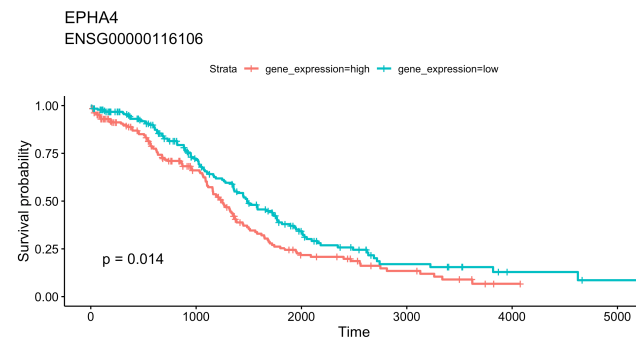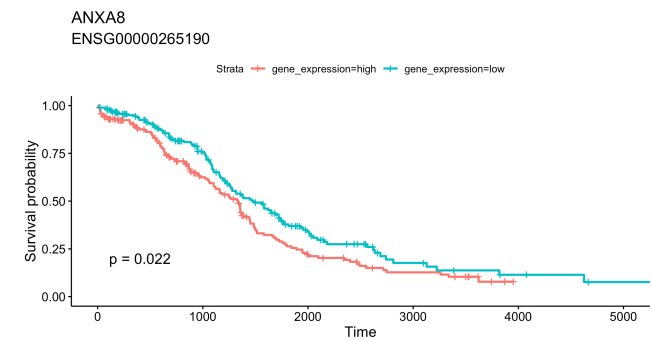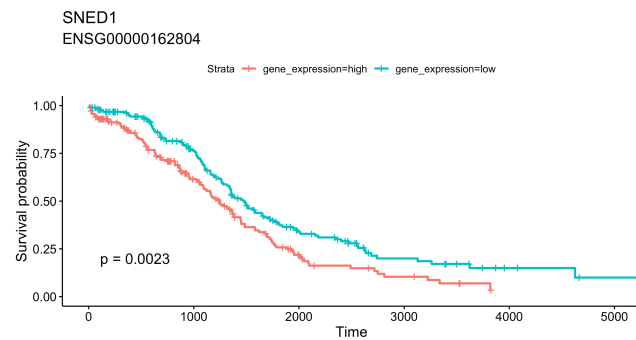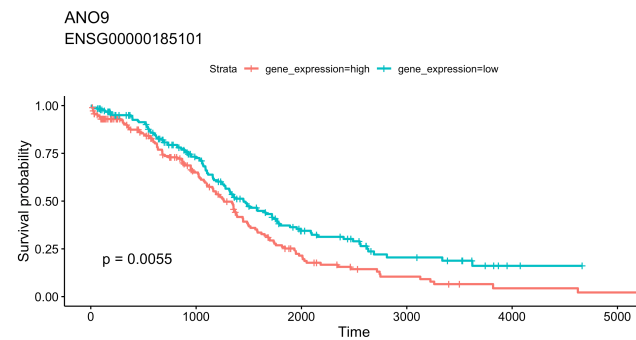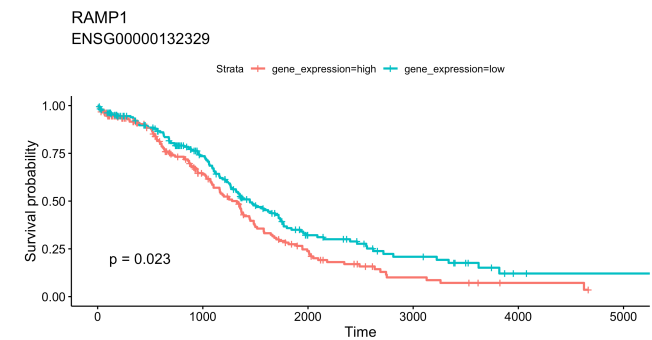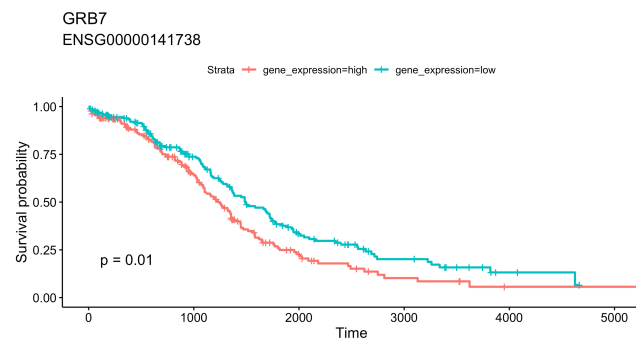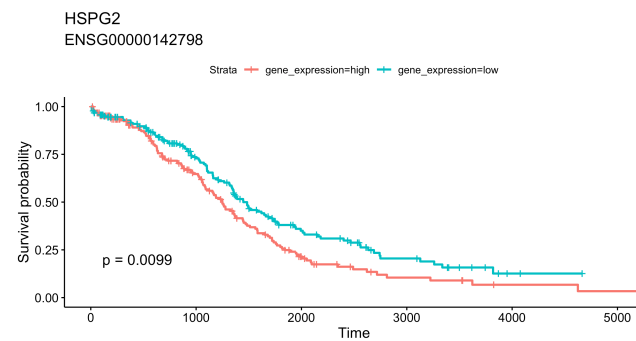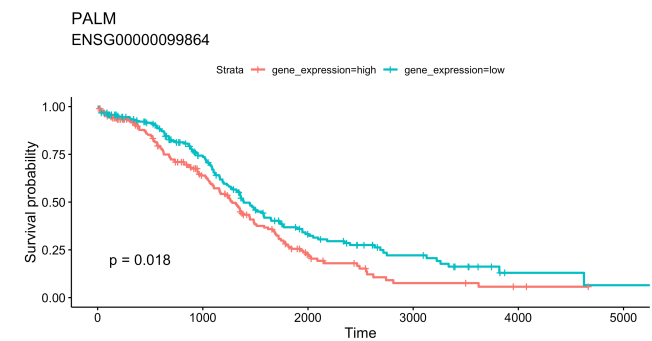

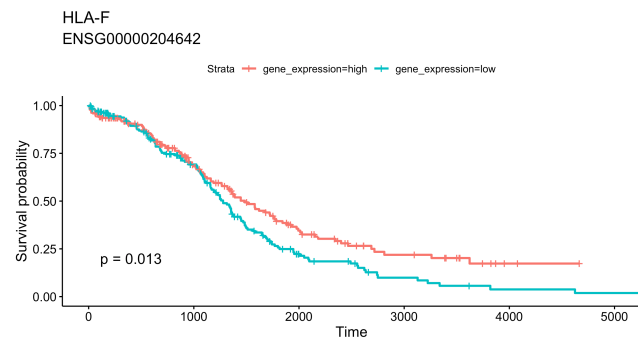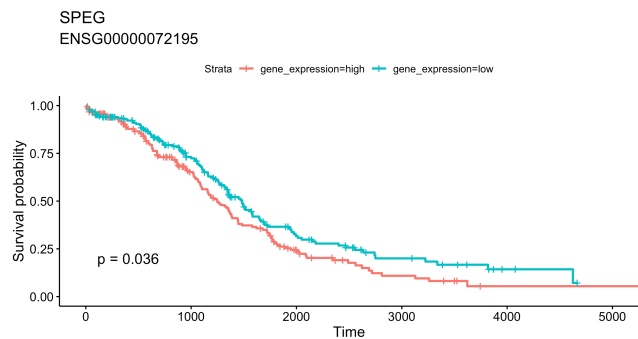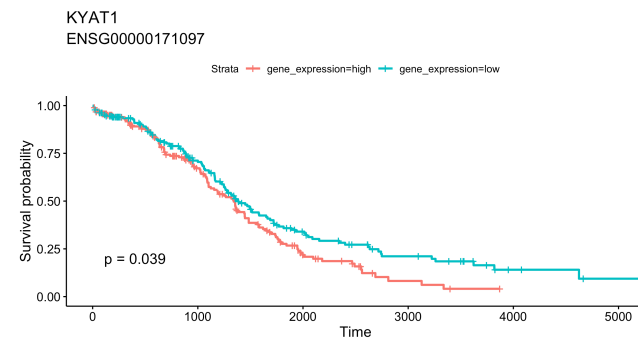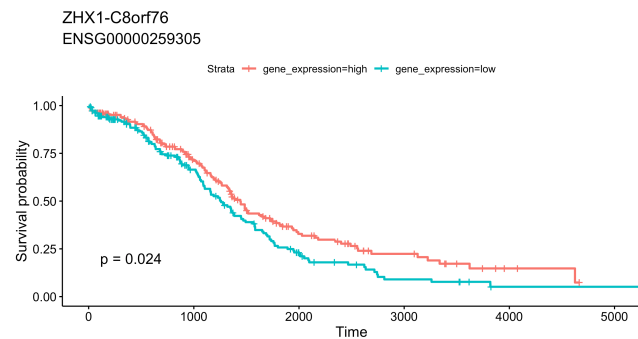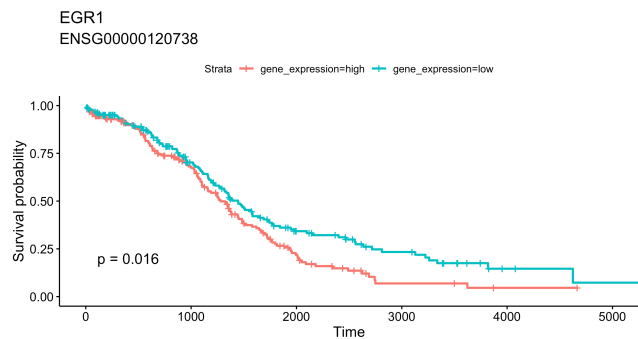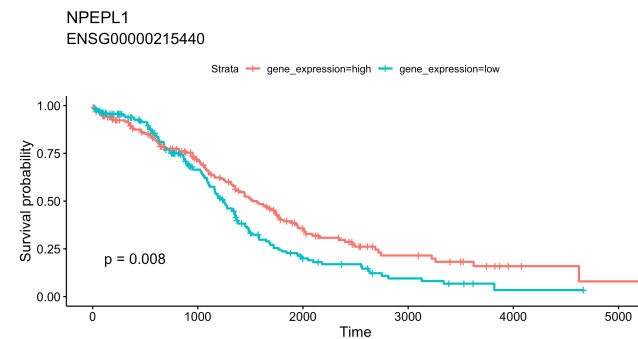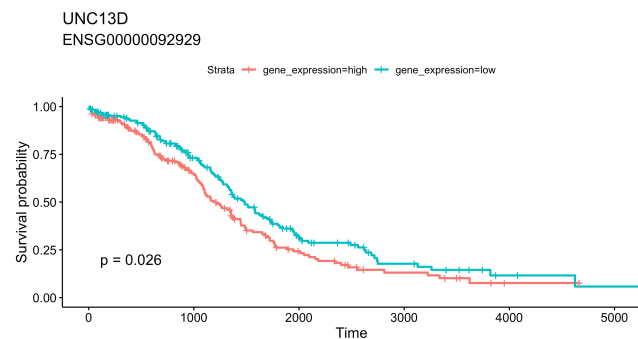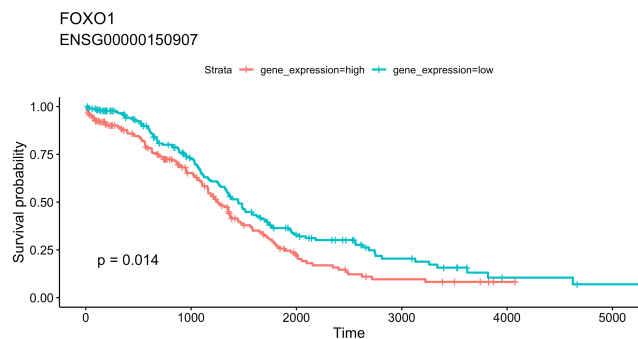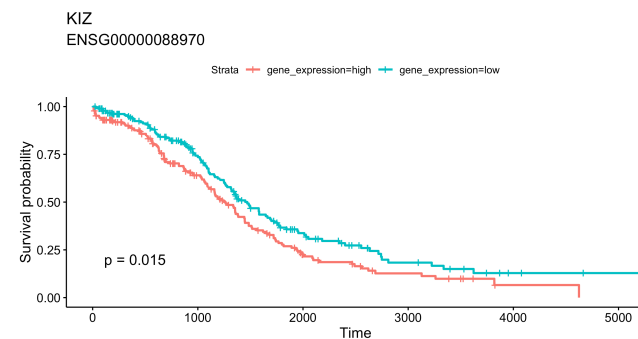

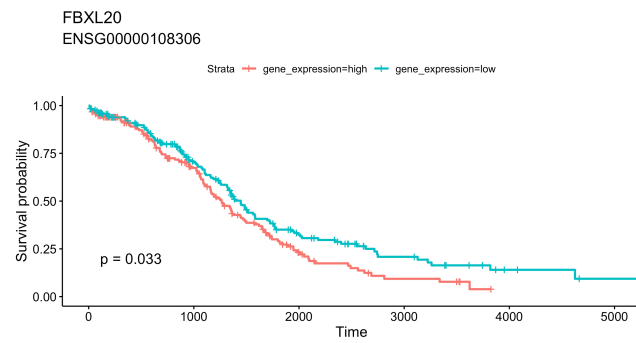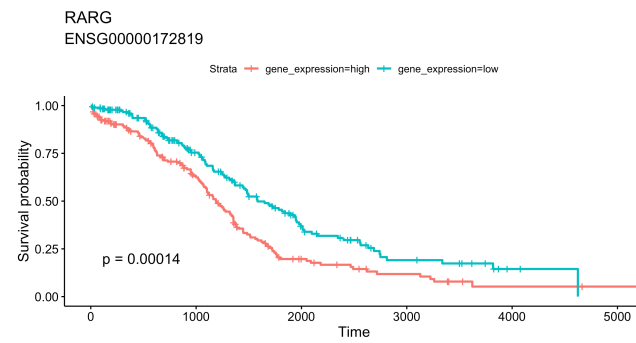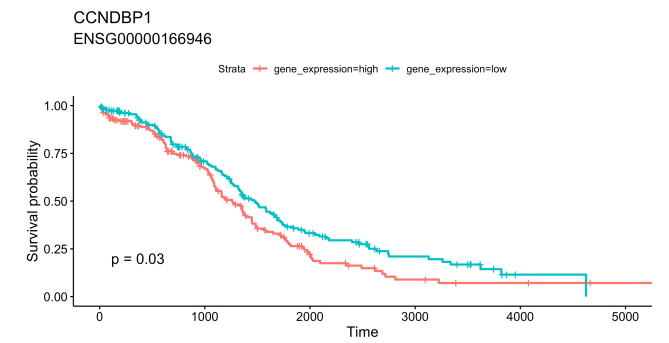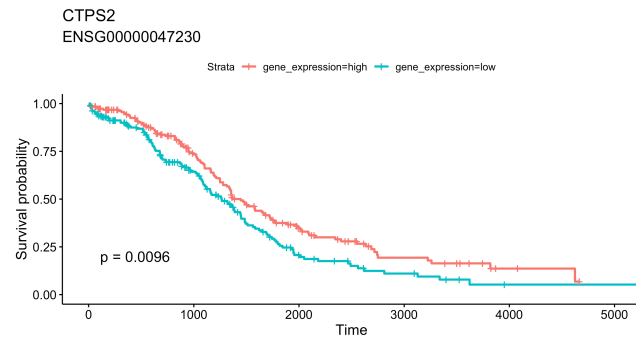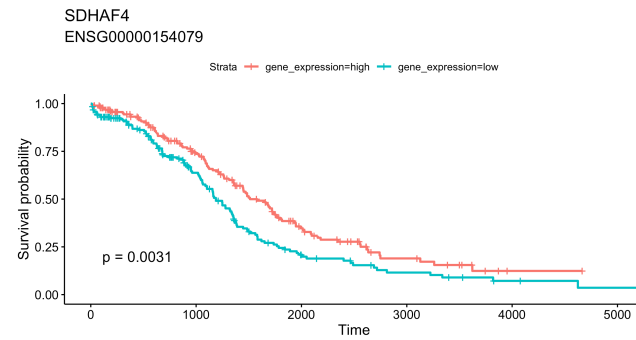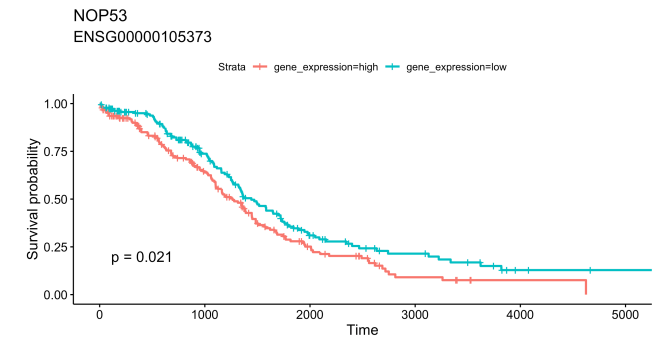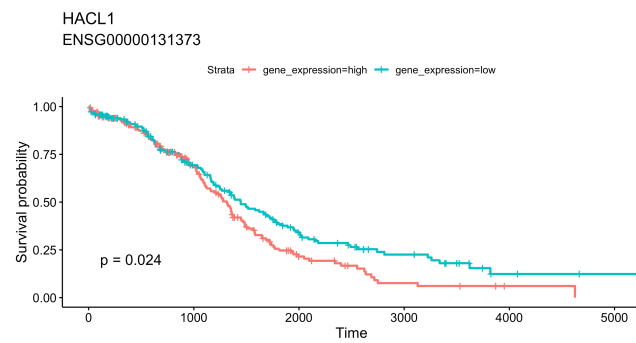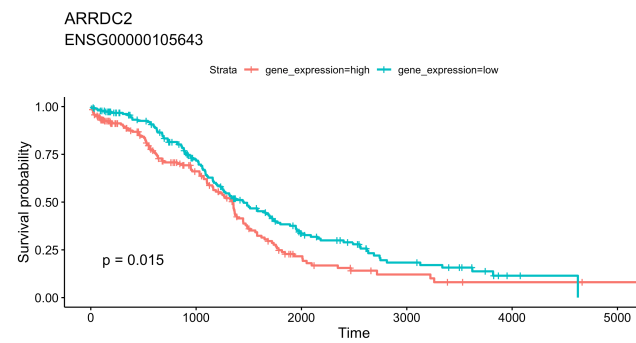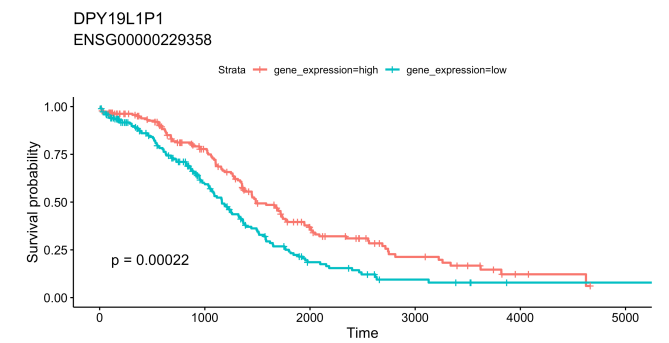

TOM1L2  
ENSG00000175662

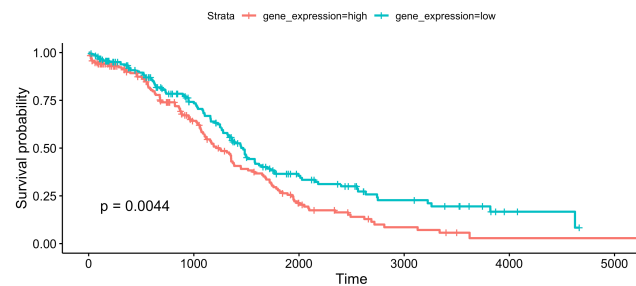

DNPH1  
ENSG00000112667

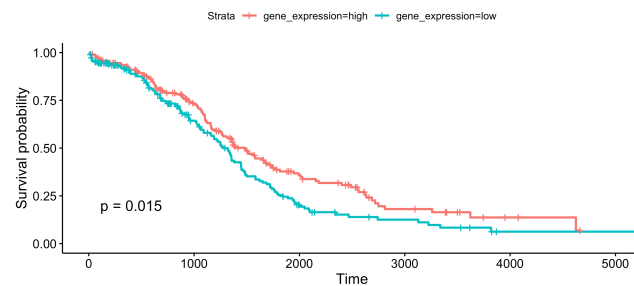

LPIN3  
ENSG00000132793

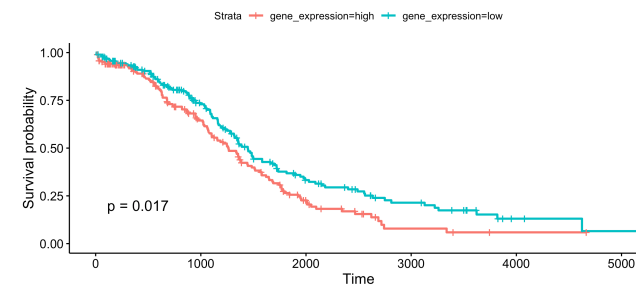

PLD2  
ENSG00000129219

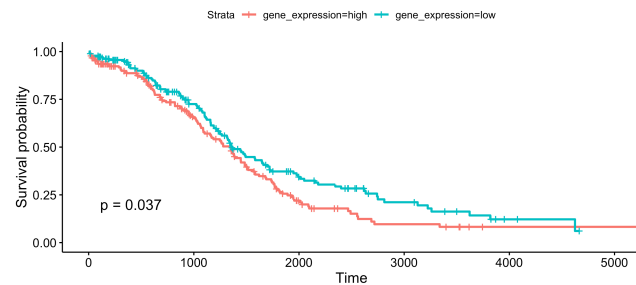

PLEKHH3  
ENSG00000068137

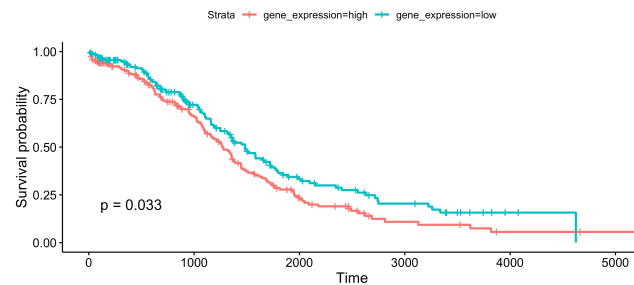

Supplement: Supplementary file 1 [file cancers-12-02183-s001.zip › cancers-855025-SUPPLE-XML/cancers-855025-supple-proof/Suppl_Fig11_ovarian_UPregulated_genes.pdf]

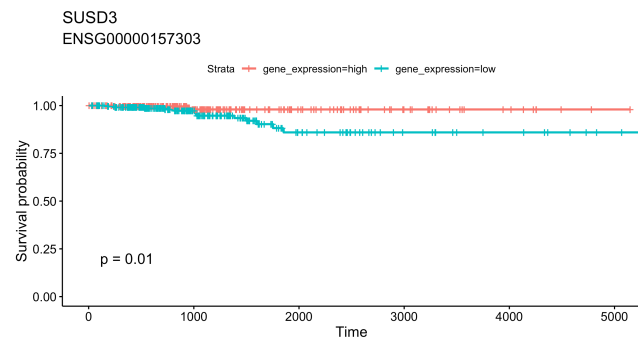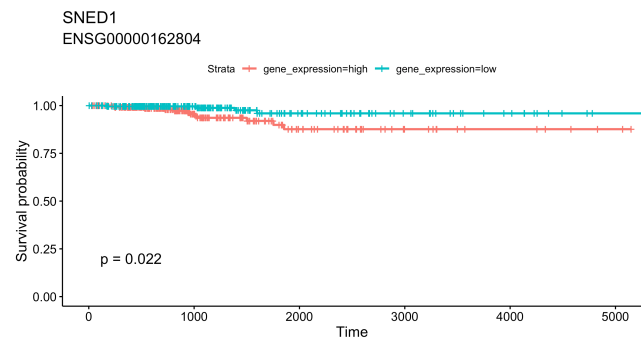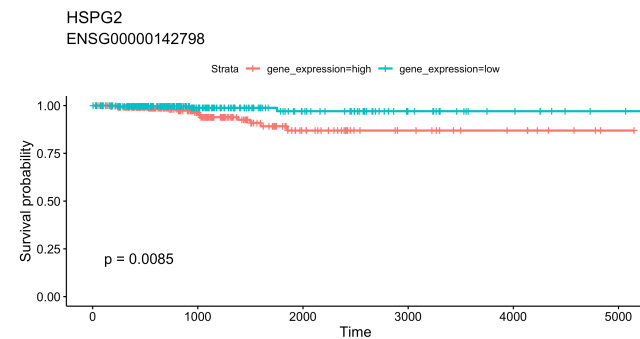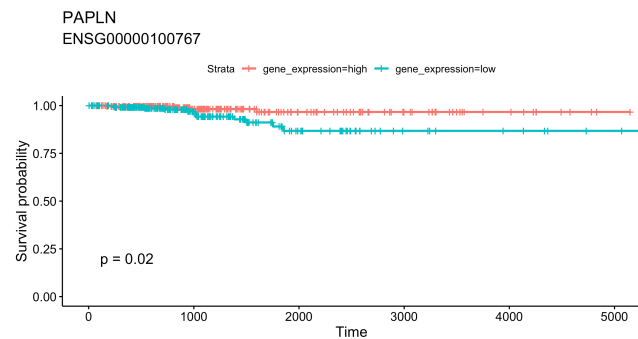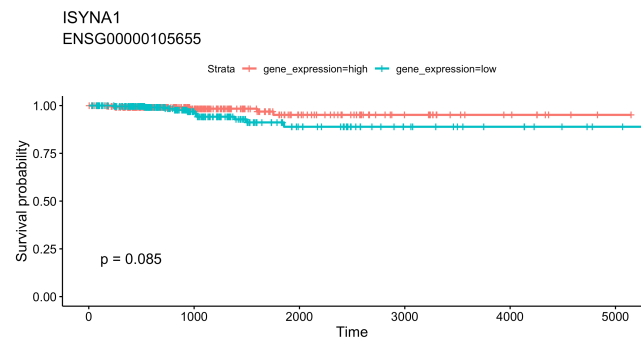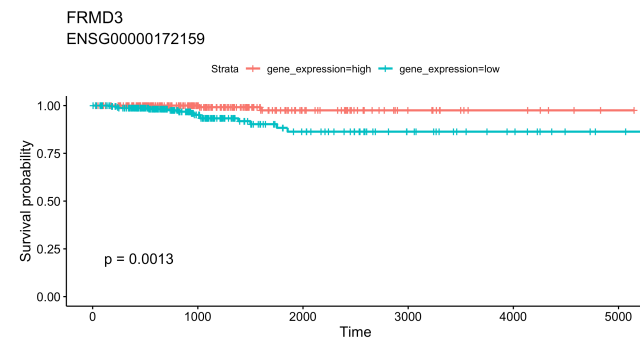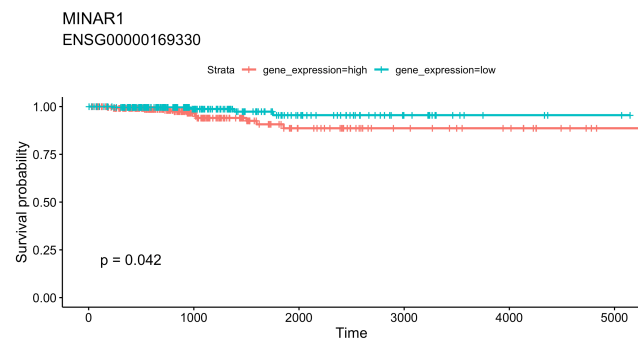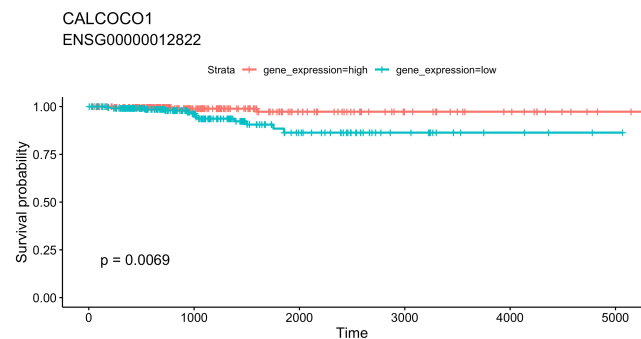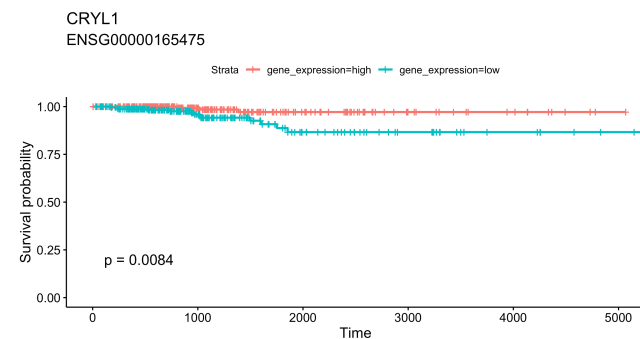

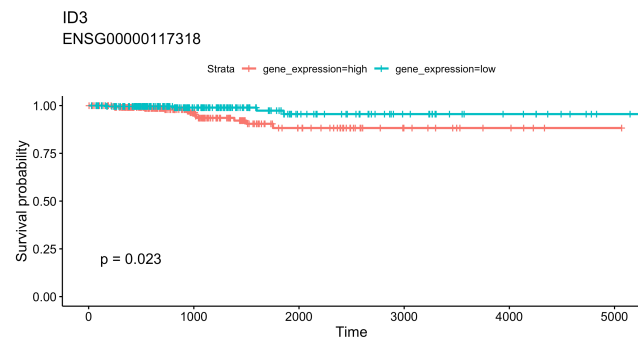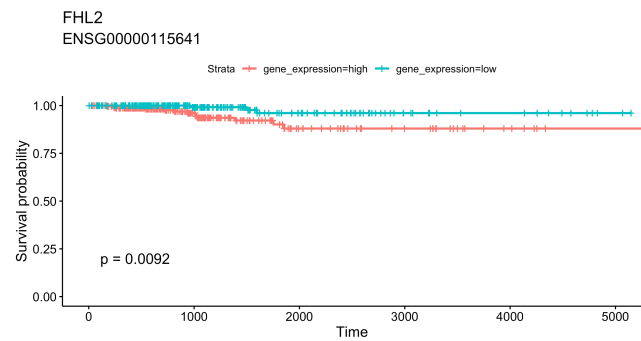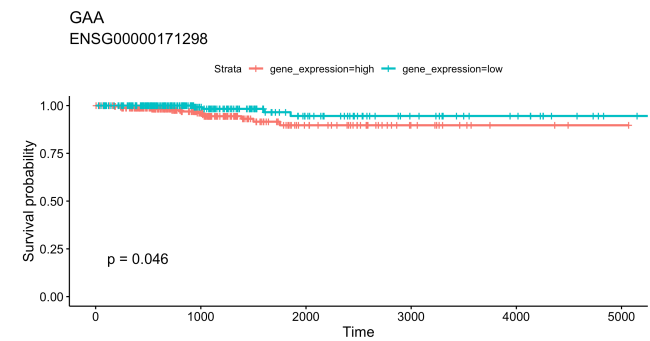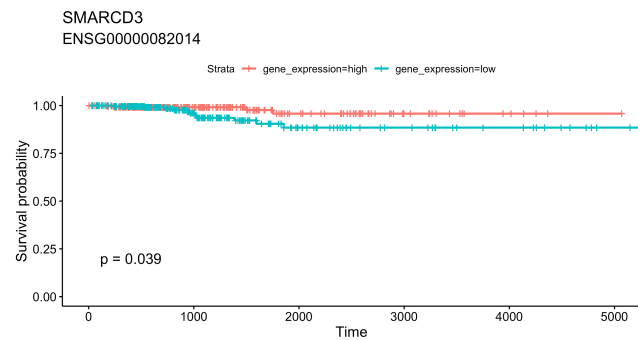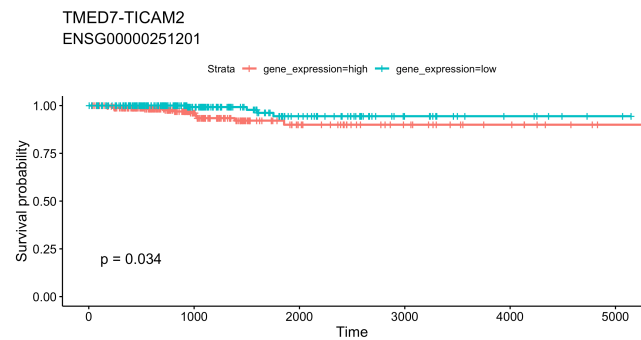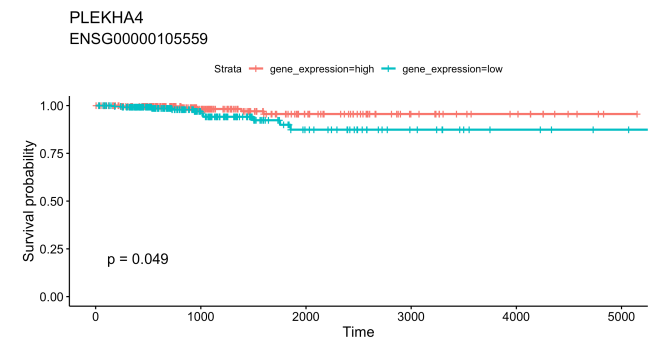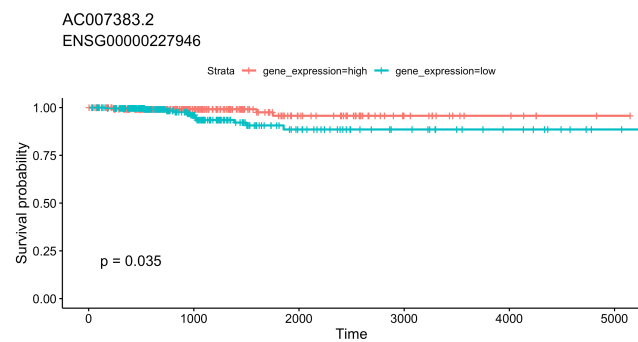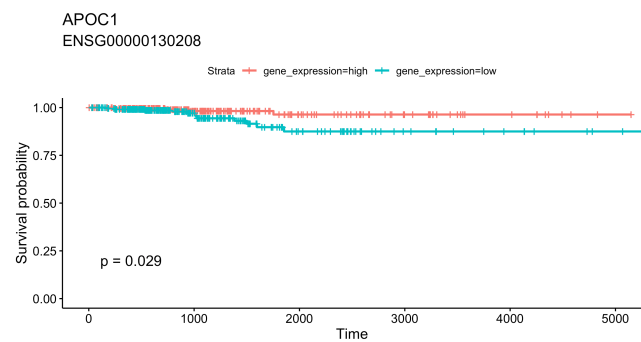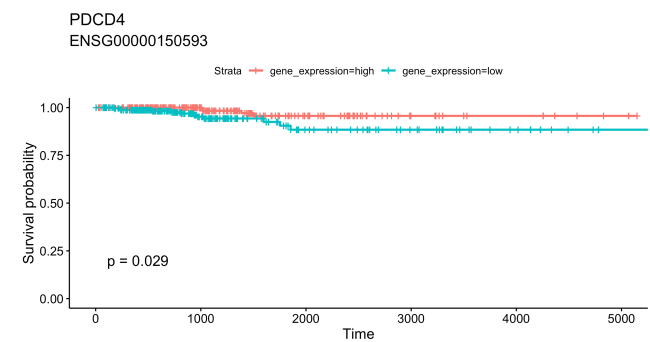

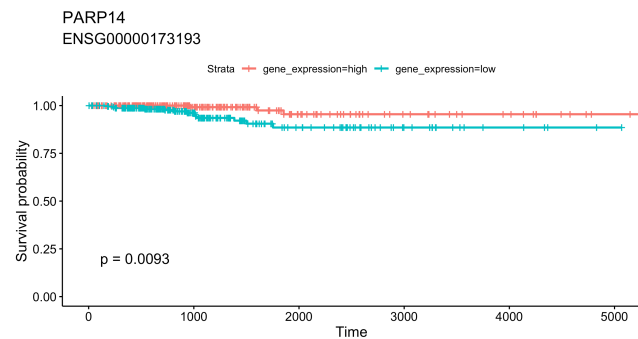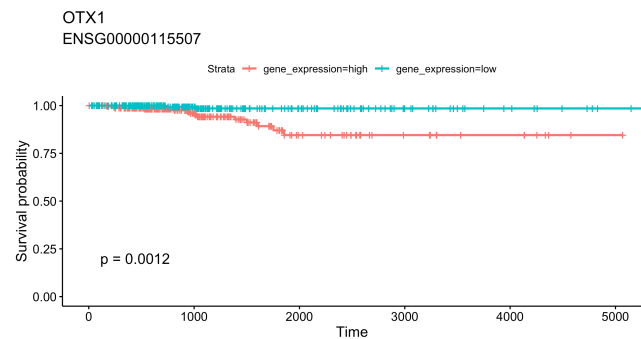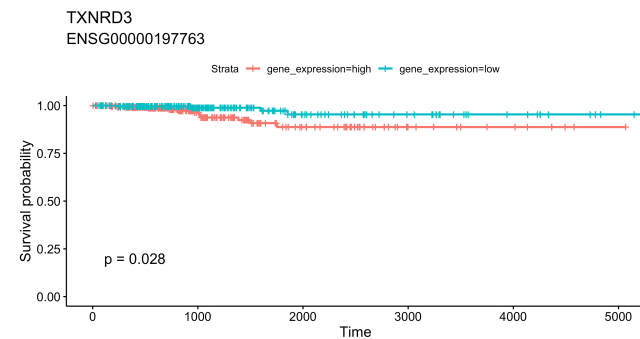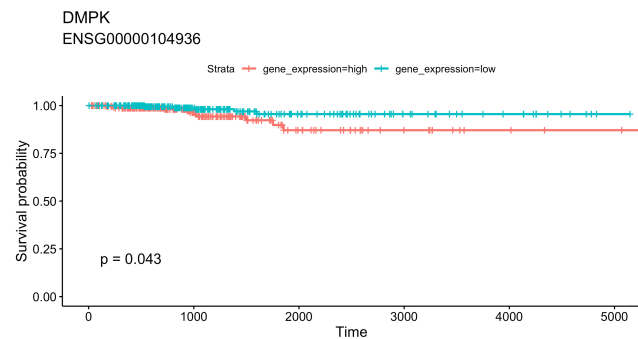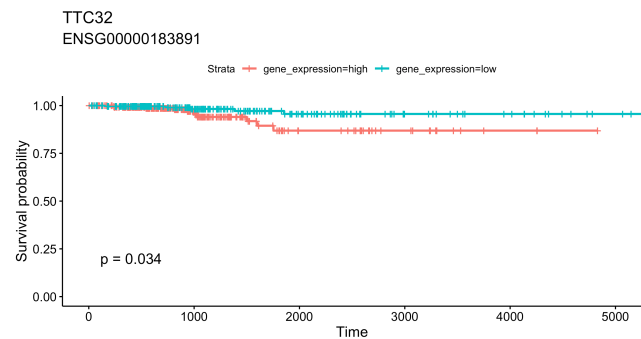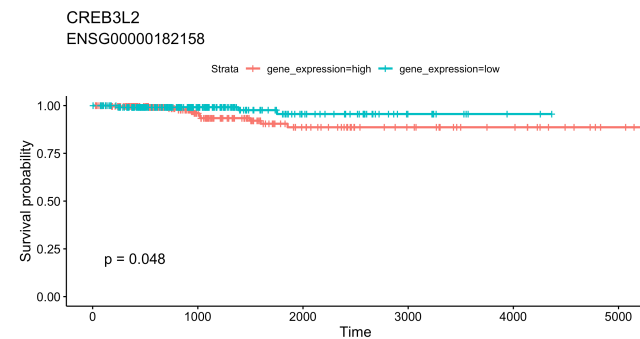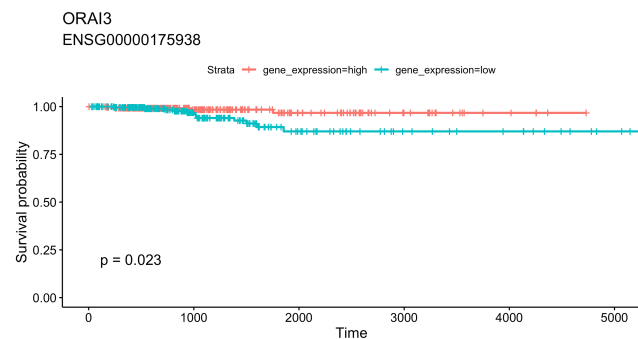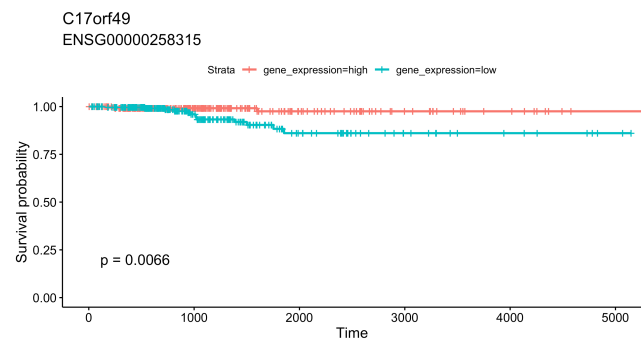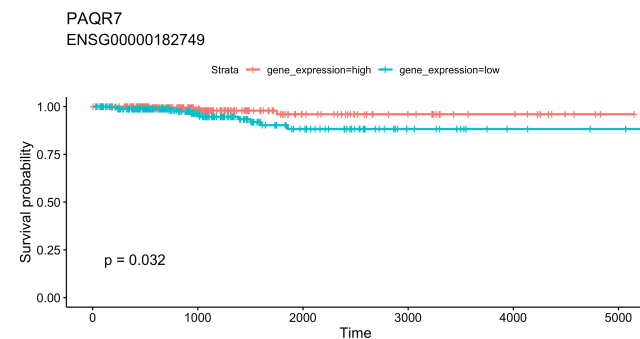

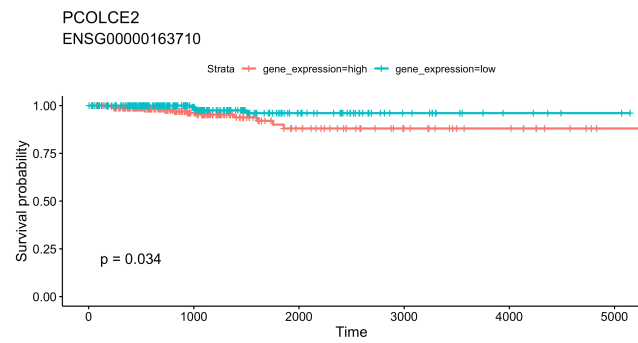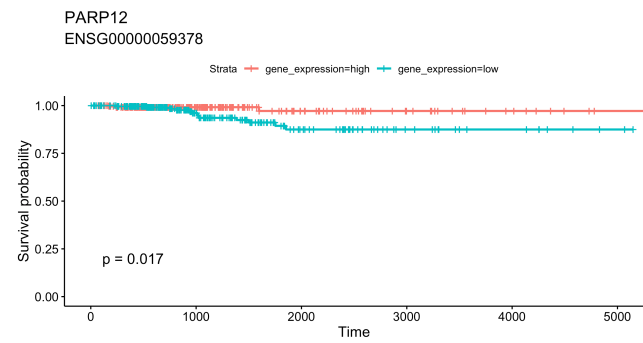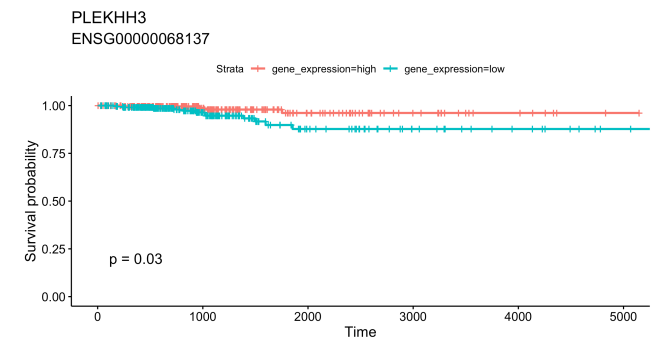

Supplement: Supplementary file 1 [file cancers-12-02183-s001.zip › cancers-855025-SUPPLE-XML/cancers-855025-supple-proof/Suppl_Fig13_thyroid_UPregulated_genes.pdf]

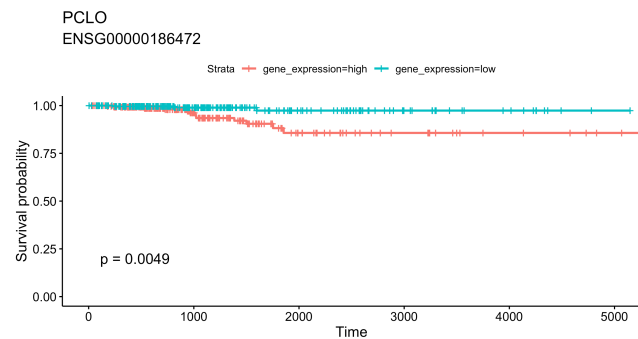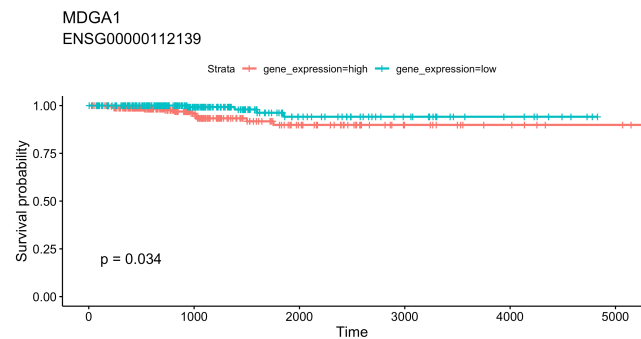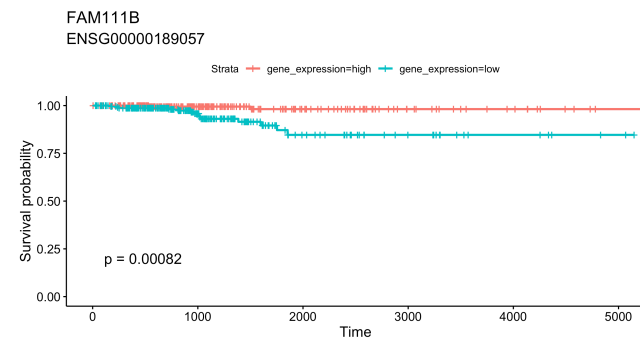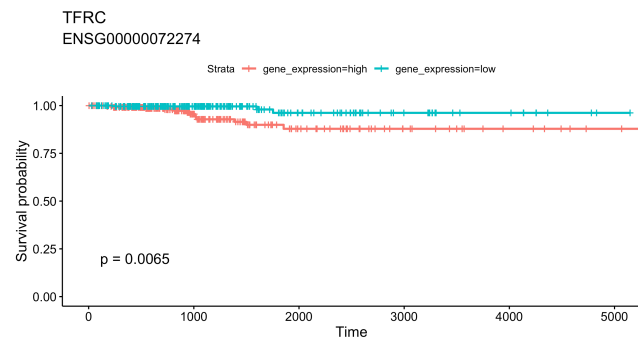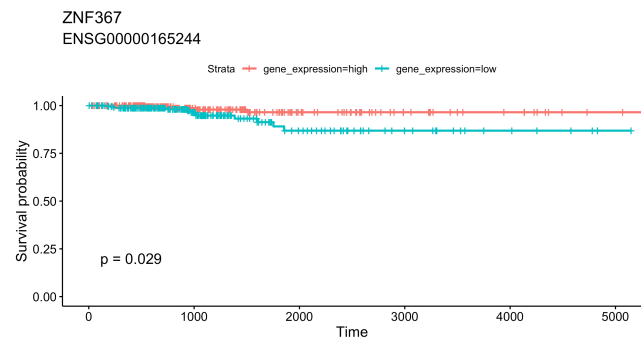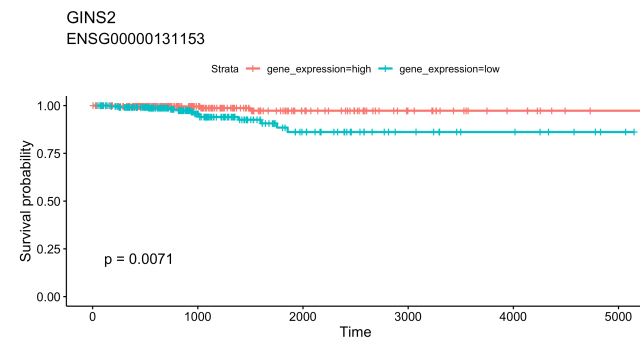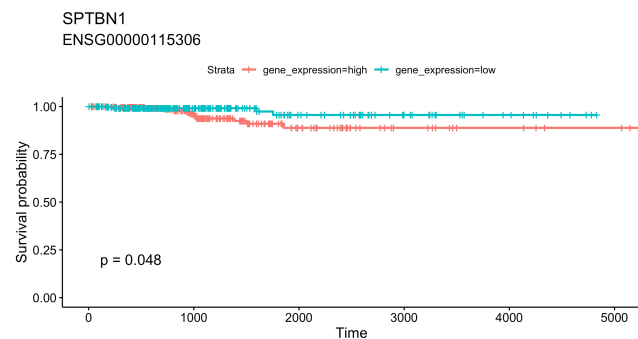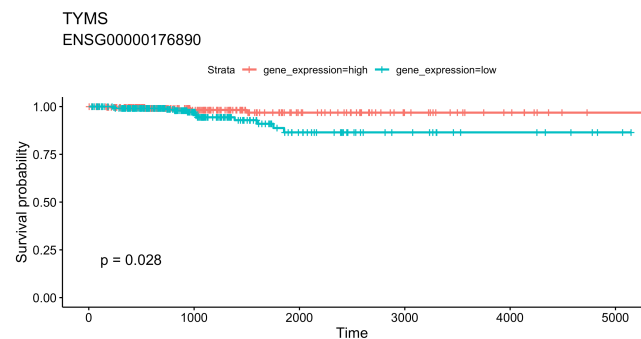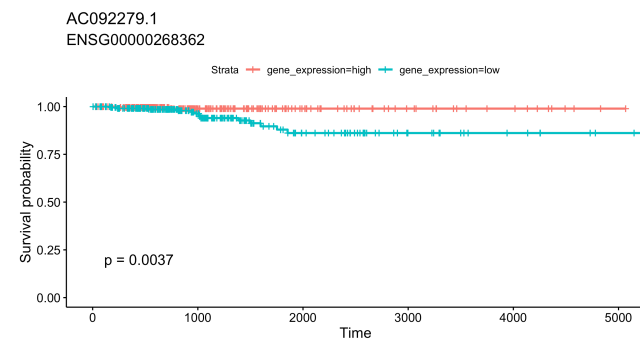

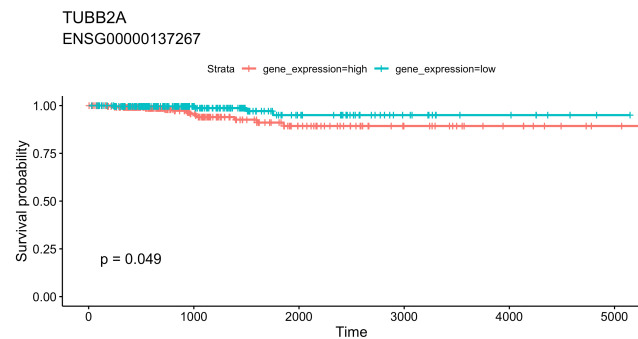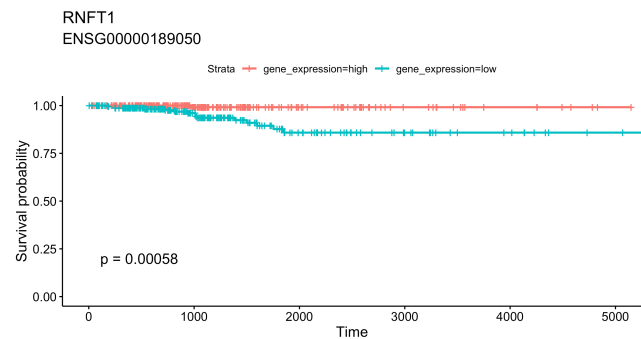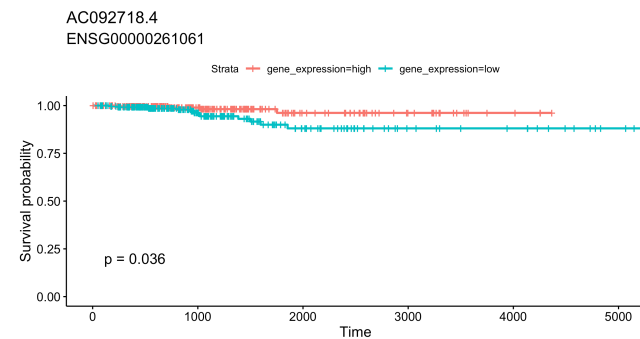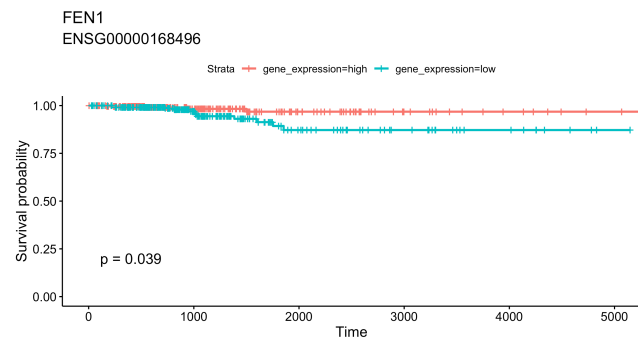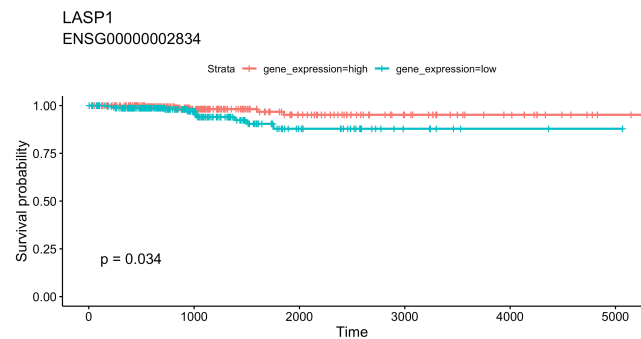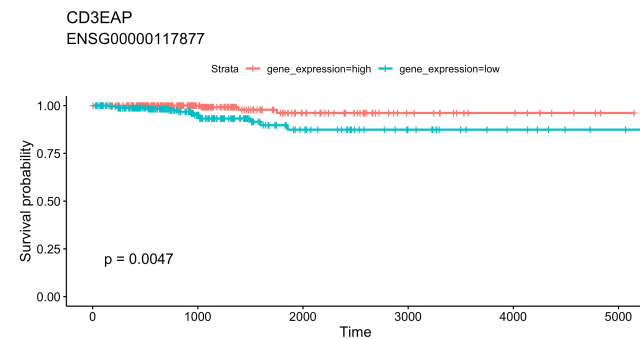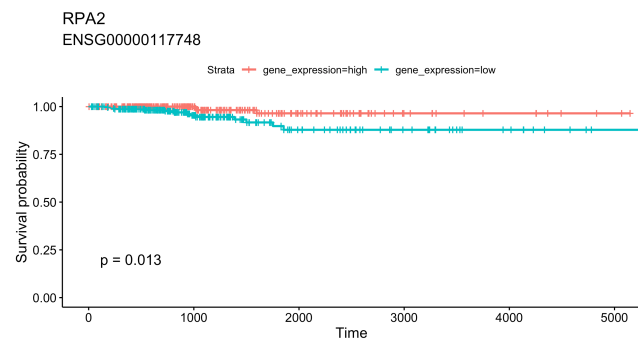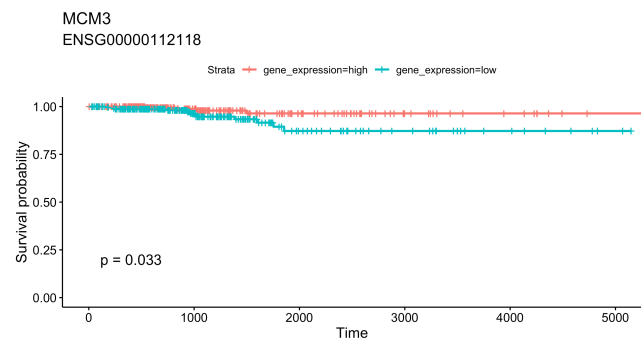

Supplement: Supplementary file 1 [file cancers-12-02183-s001.zip › cancers-855025-SUPPLE-XML/cancers-855025-supple-proof/Suppl_Fig14_thyroid_DOWNregulated_genes.pdf]

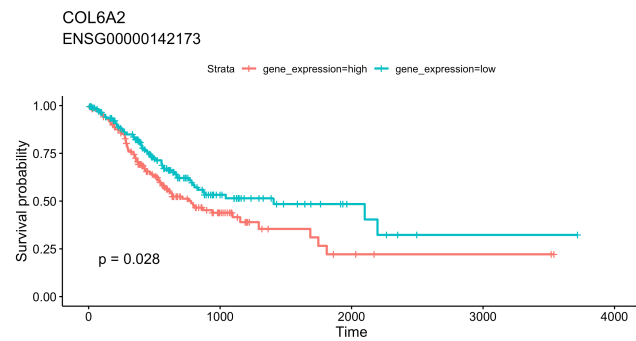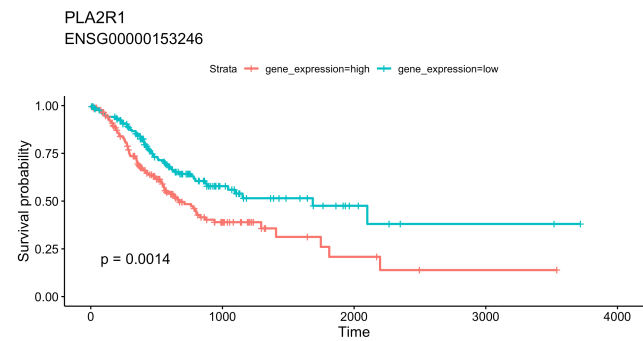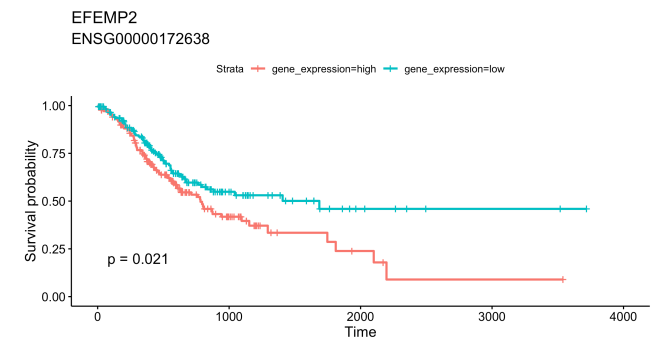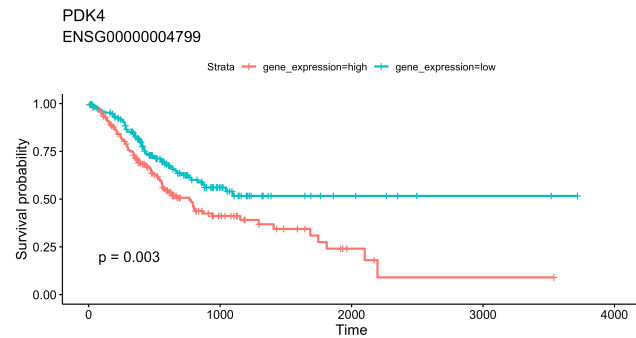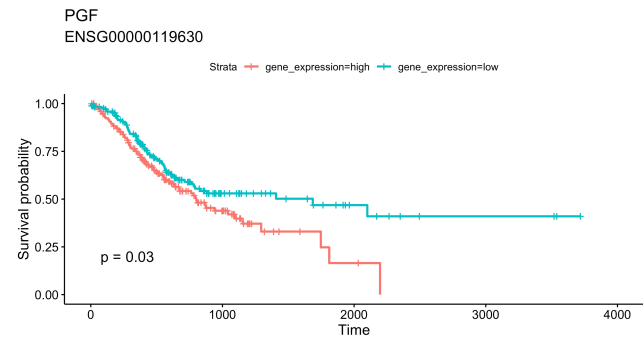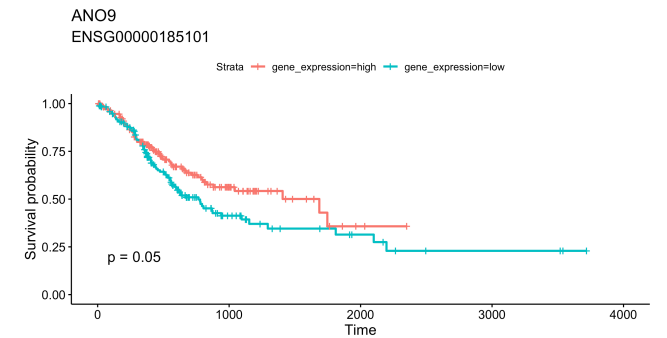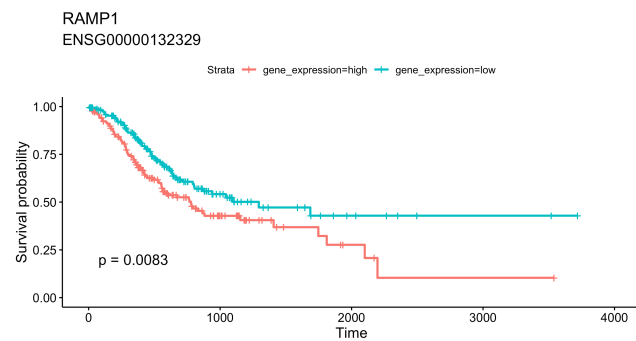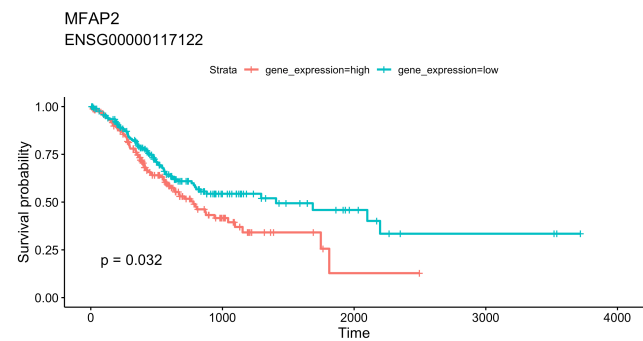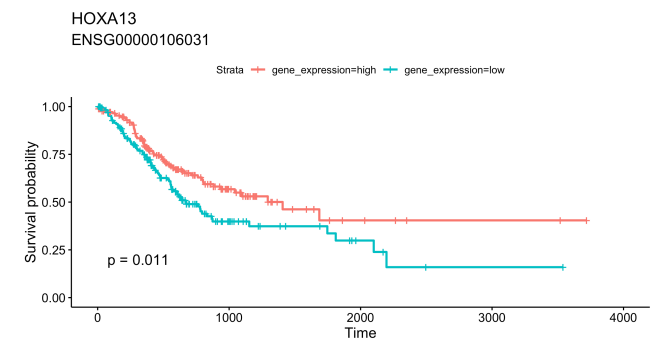

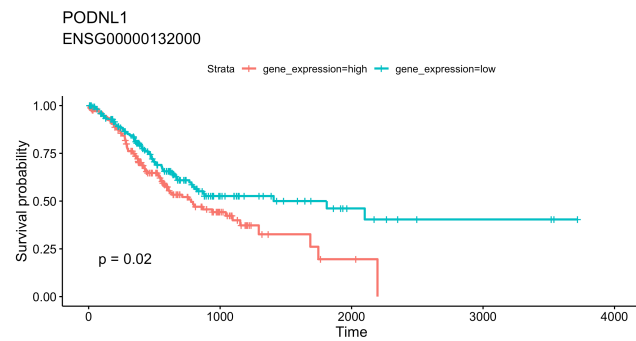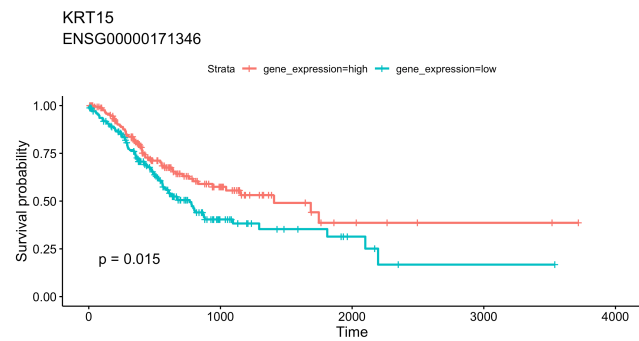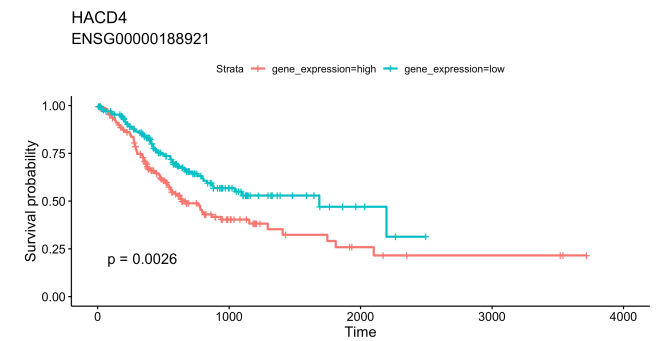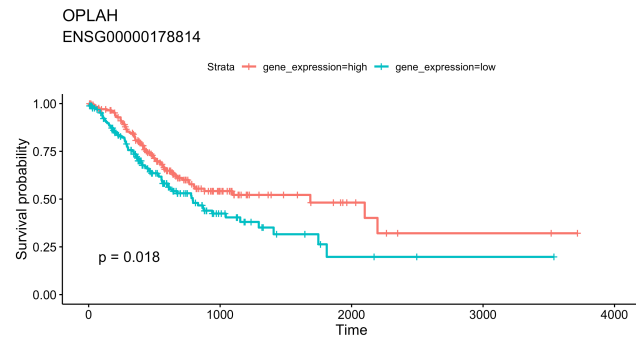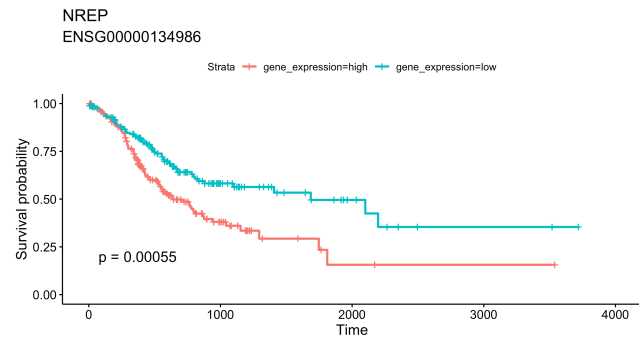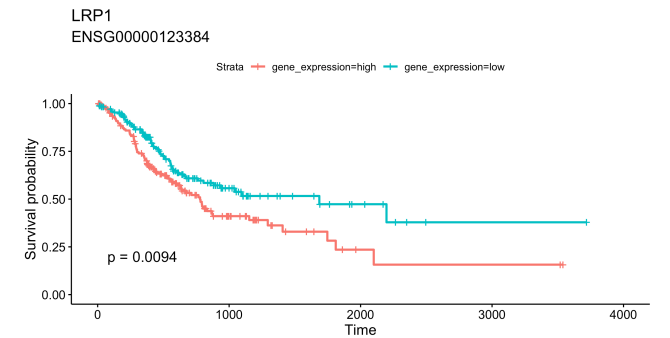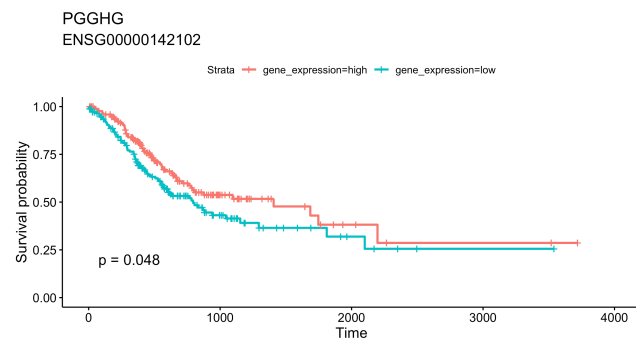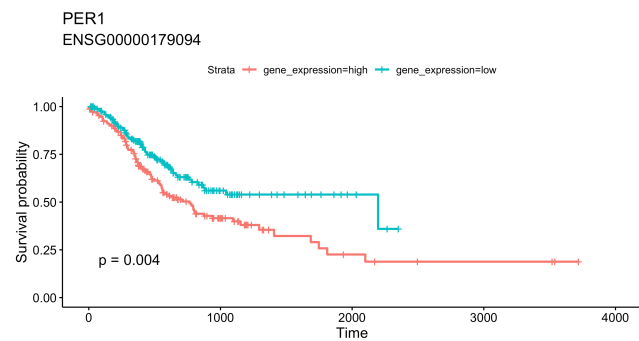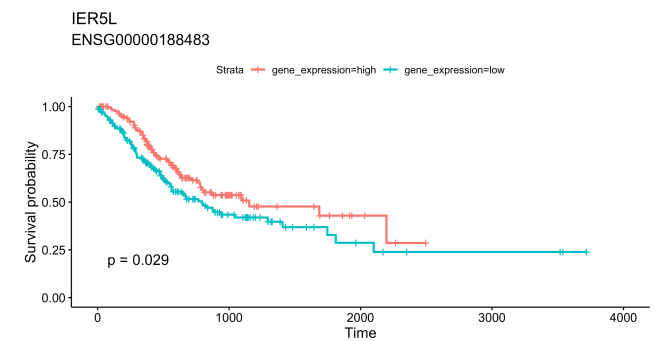

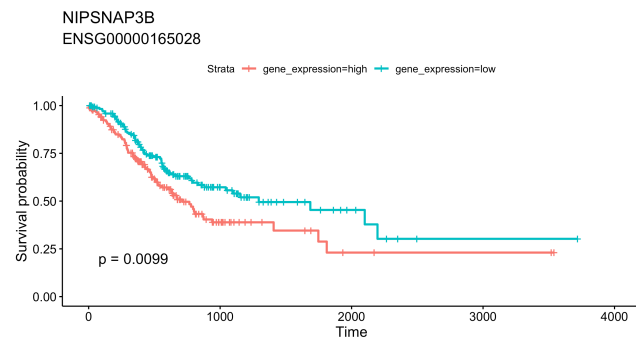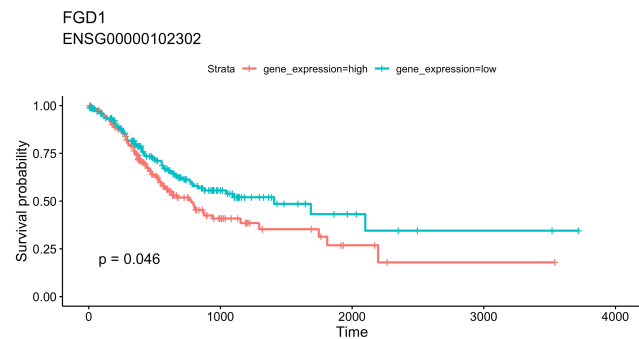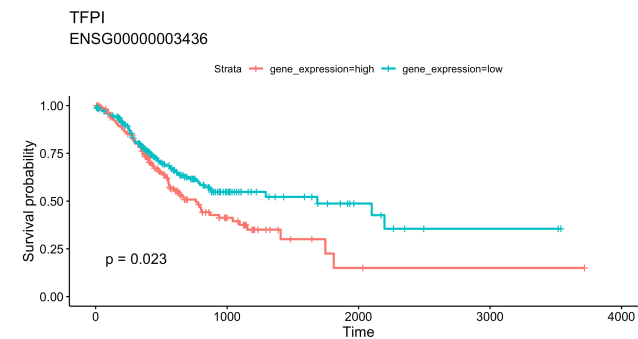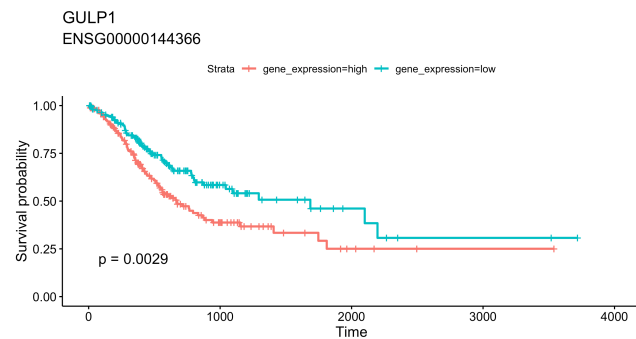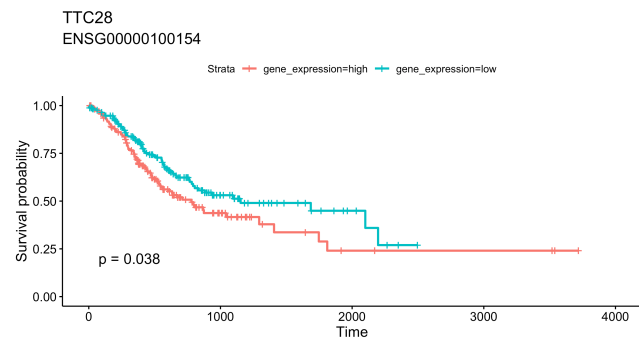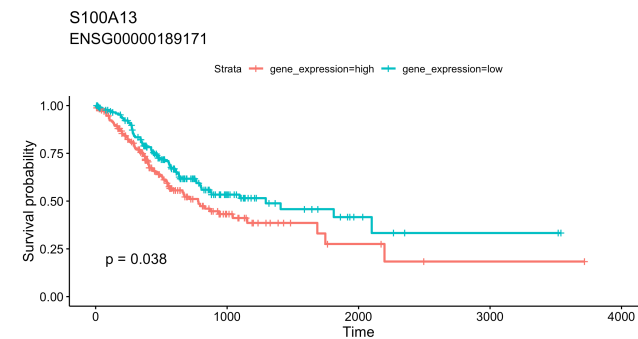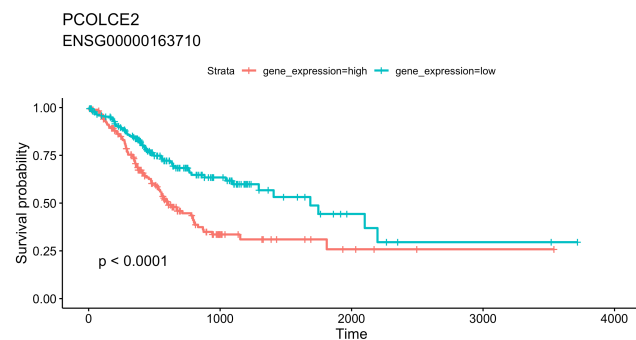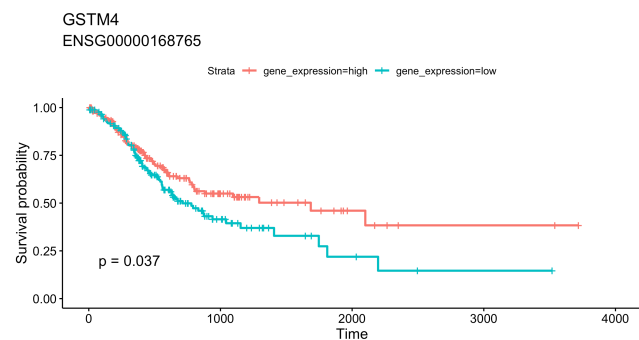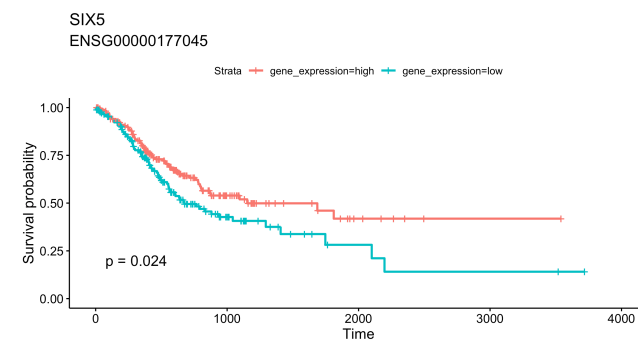

POLRMT  
ENSG00000099821

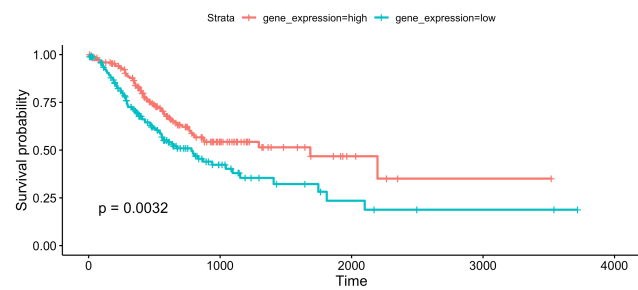

COMMD10  
ENSG00000145781

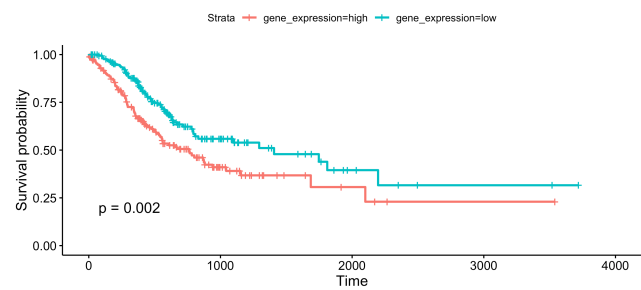

Supplement: Supplementary file 1 [file cancers-12-02183-s001.zip › cancers-855025-SUPPLE-XML/cancers-855025-supple-proof/Suppl_Fig15_stomach_UPregulated_genes.pdf]

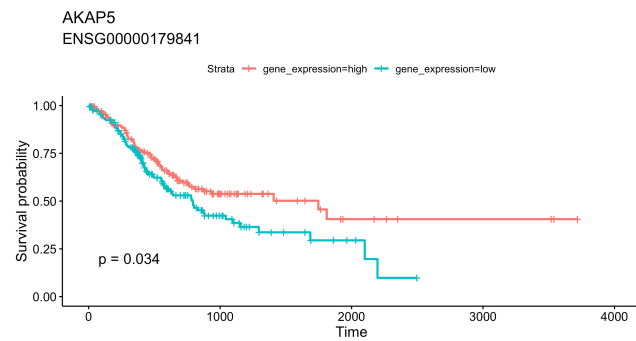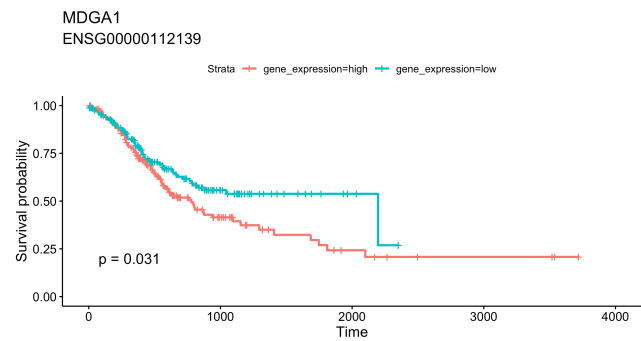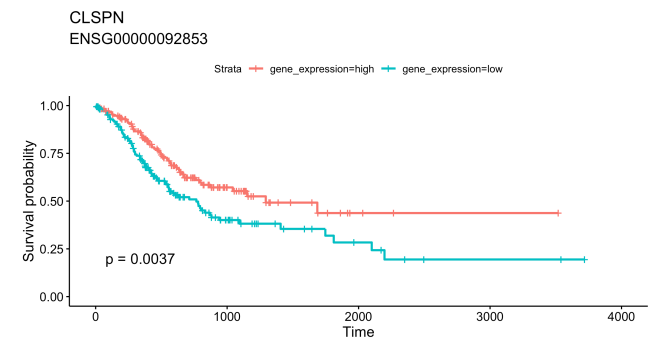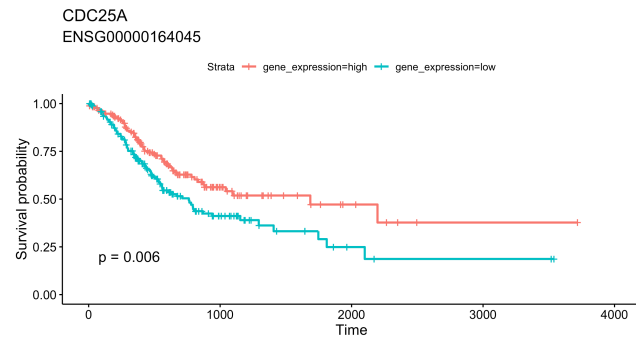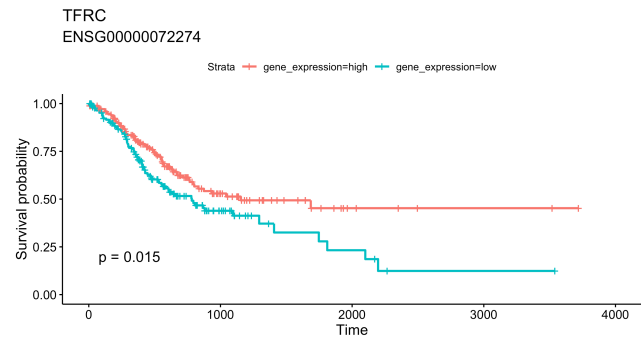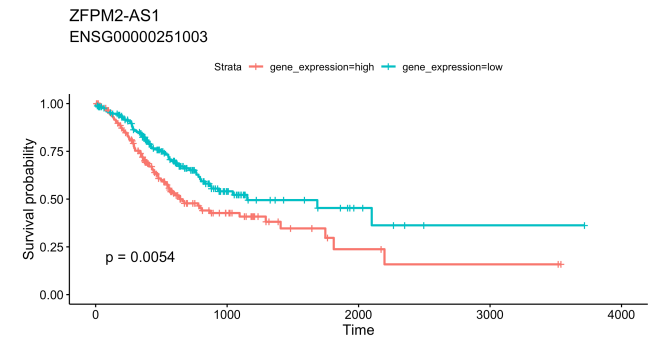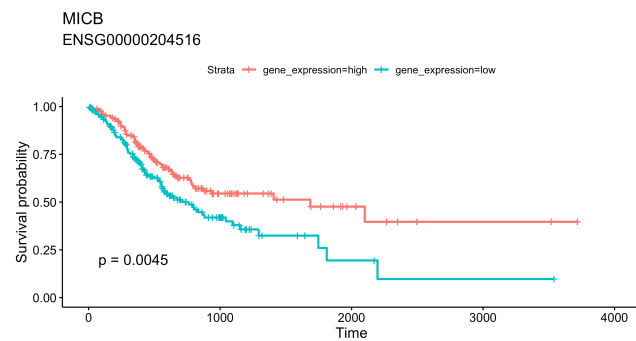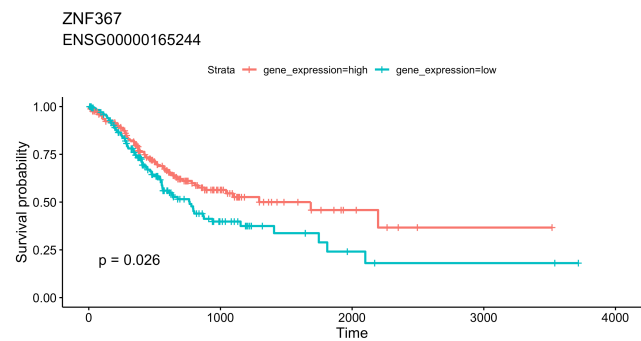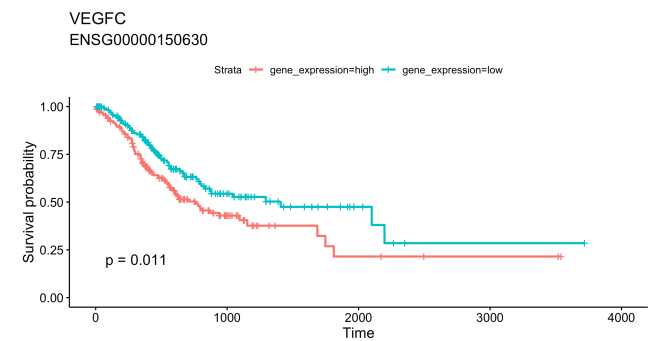

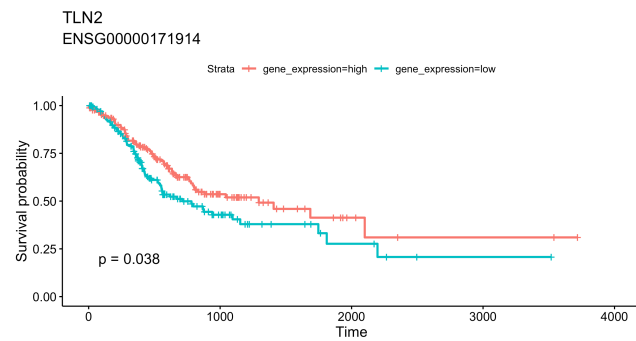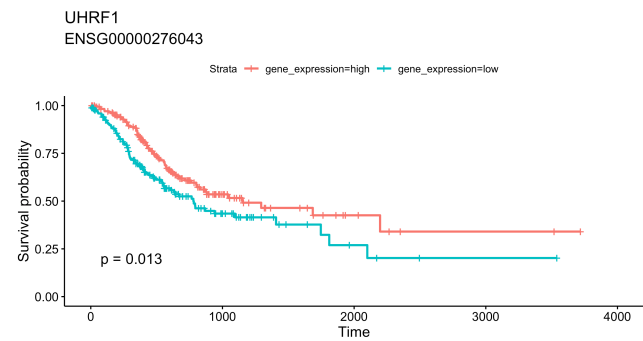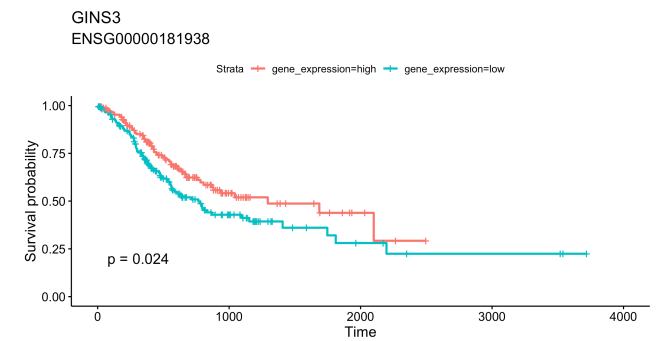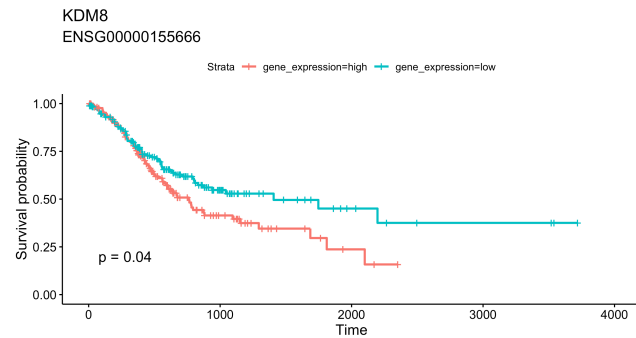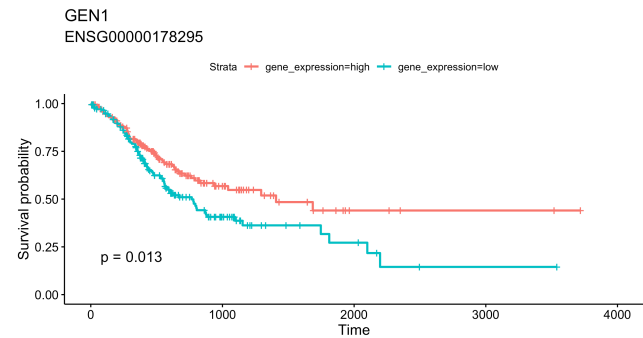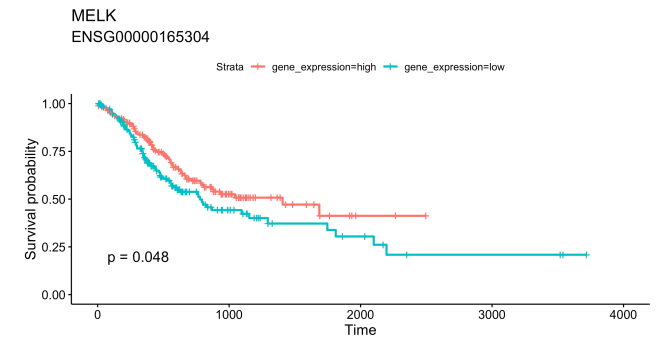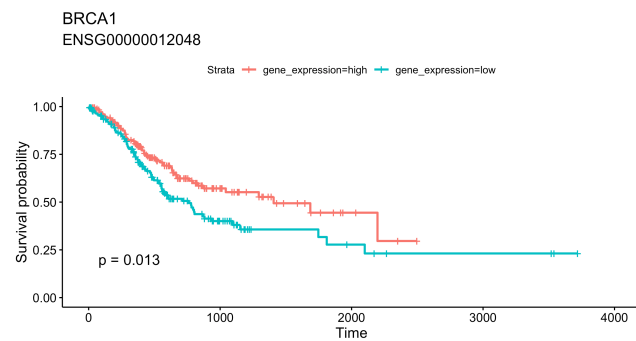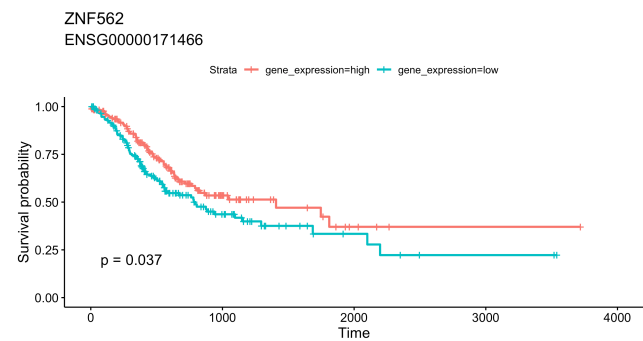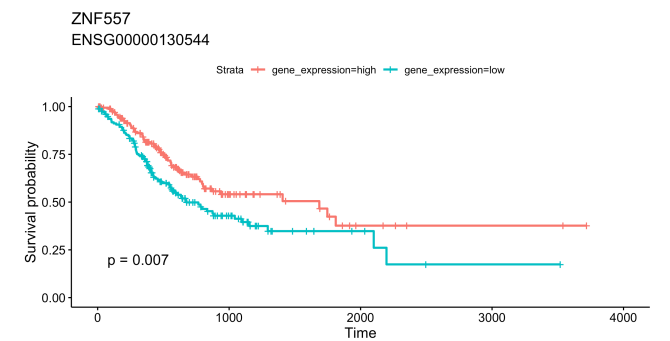

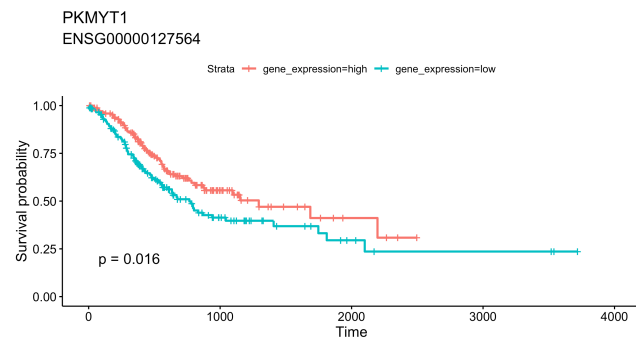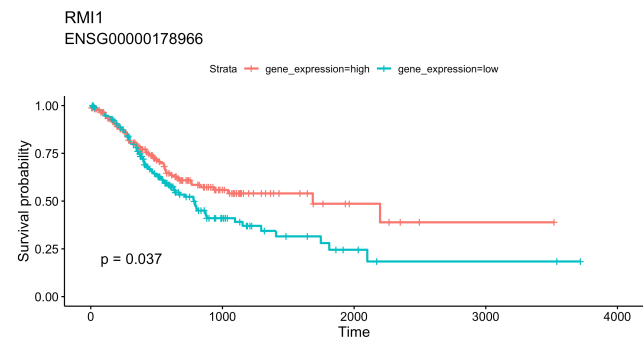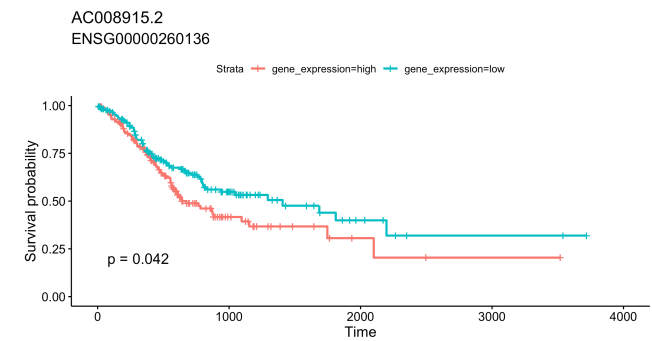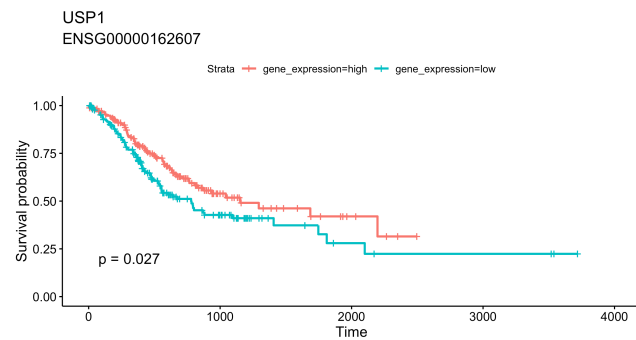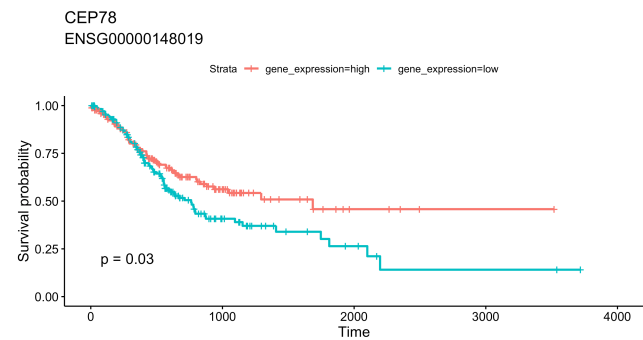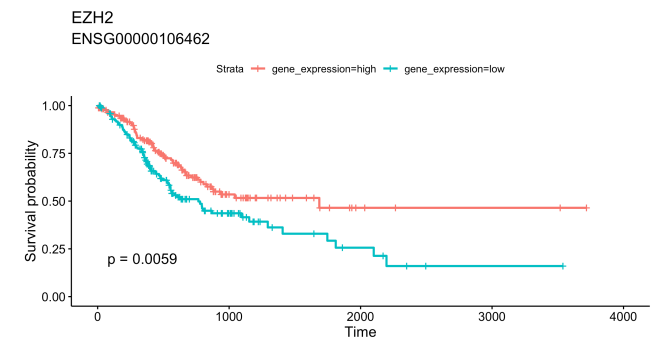

Supplement: Supplementary file 1 [file cancers-12-02183-s001.zip › cancers-855025-SUPPLE-XML/cancers-855025-supple-proof/Suppl_Fig16_stomach_DOWNregulated_genes.pdf]

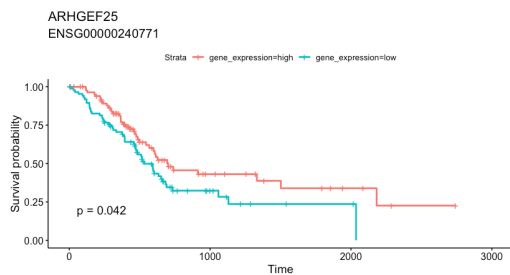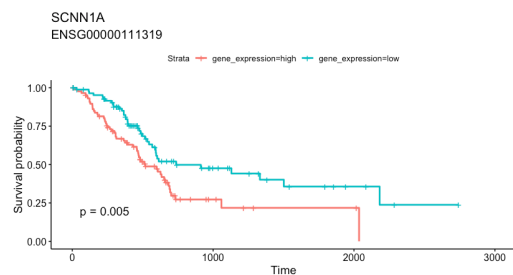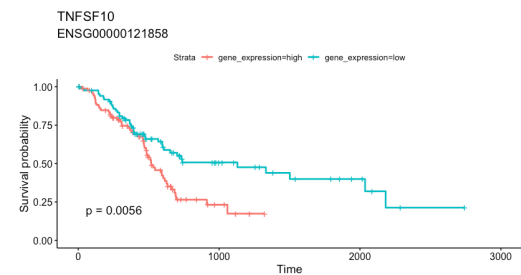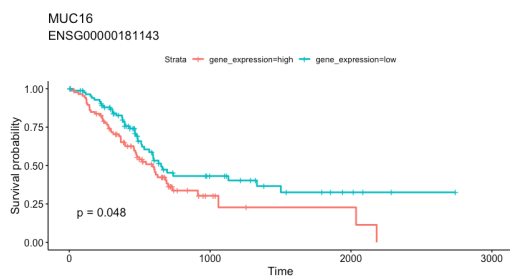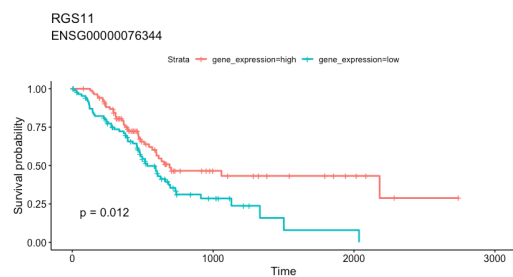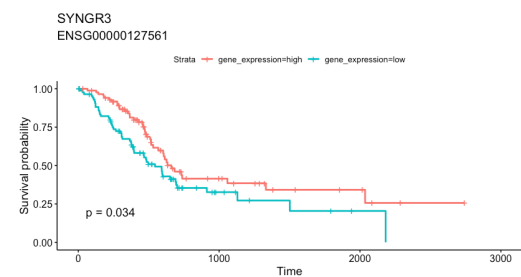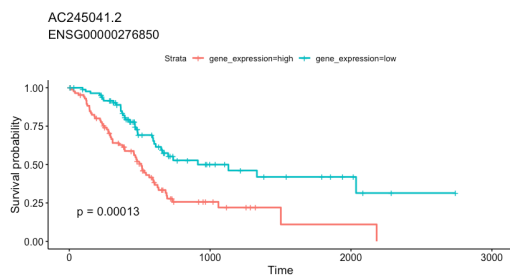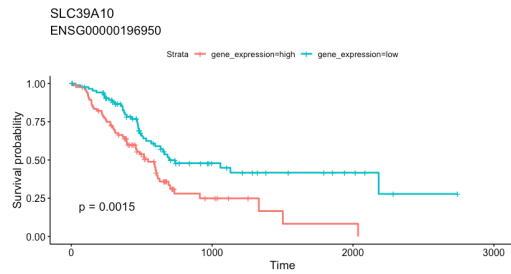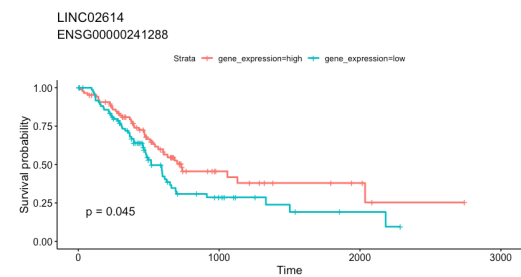

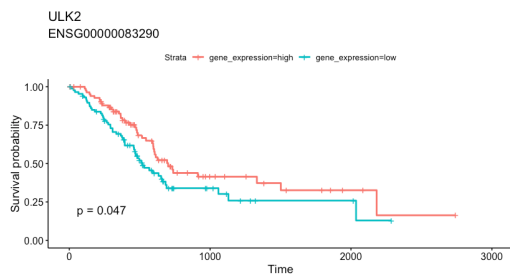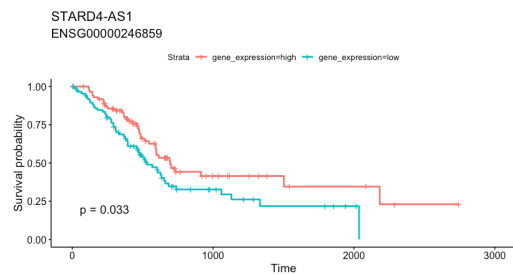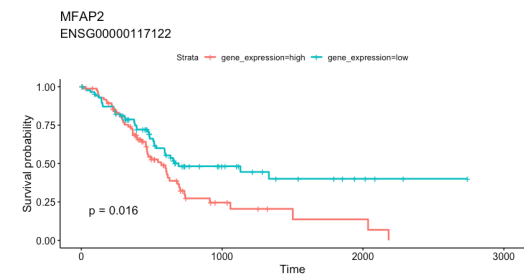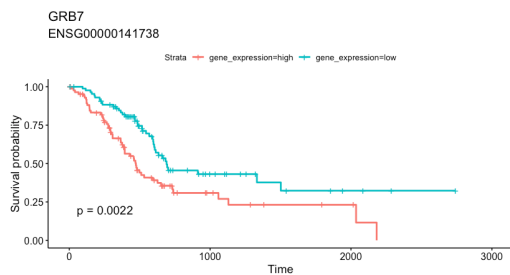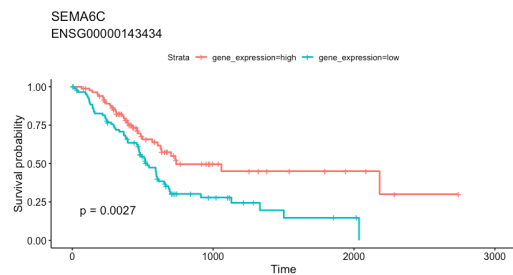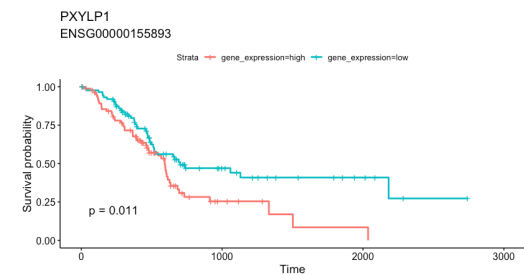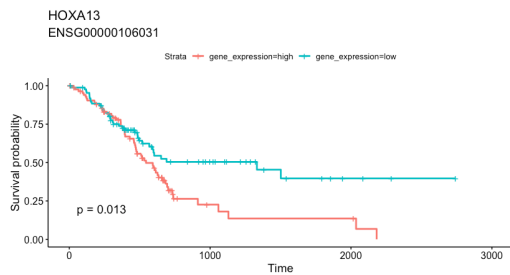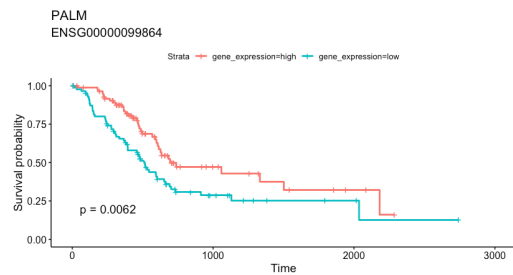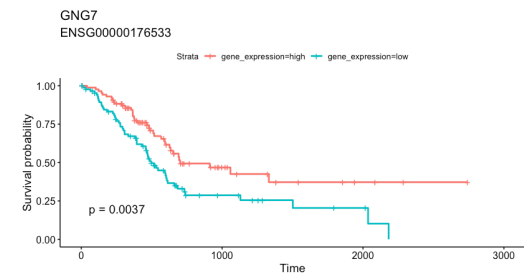

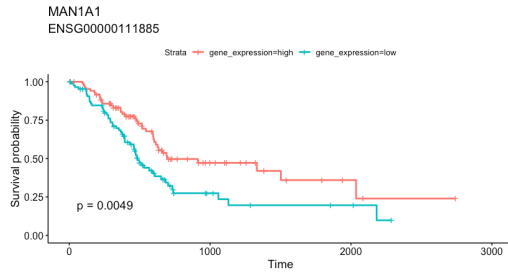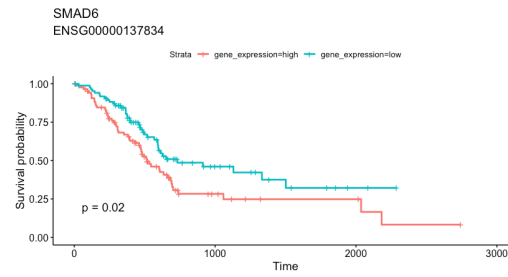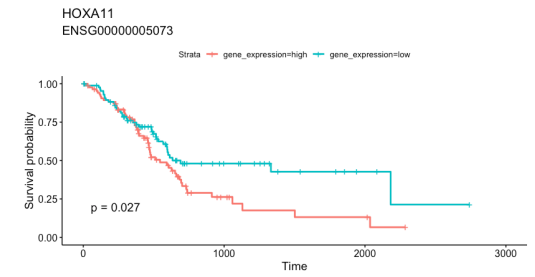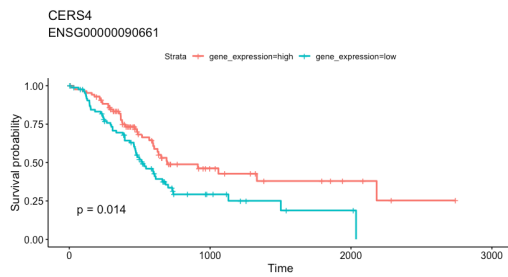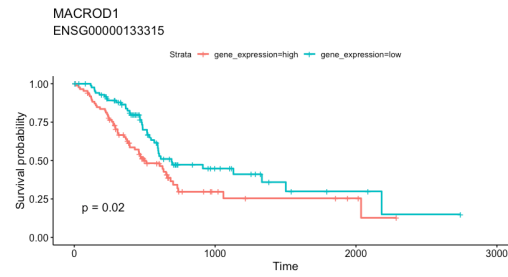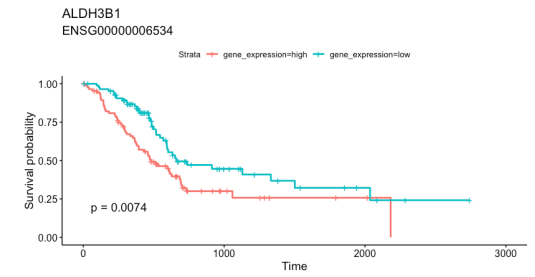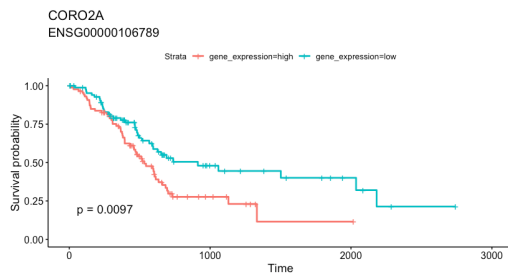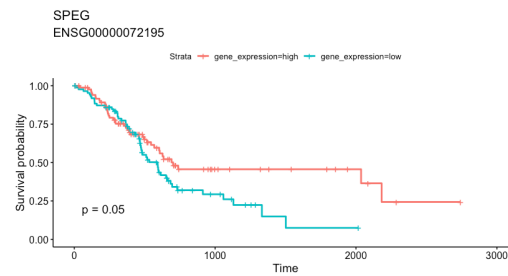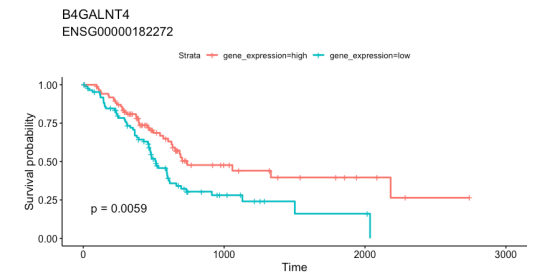

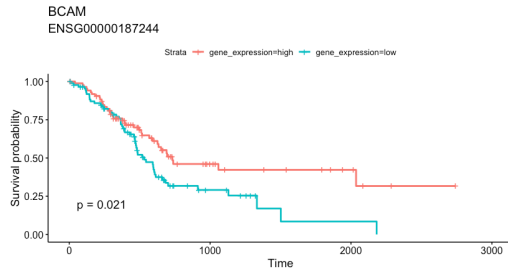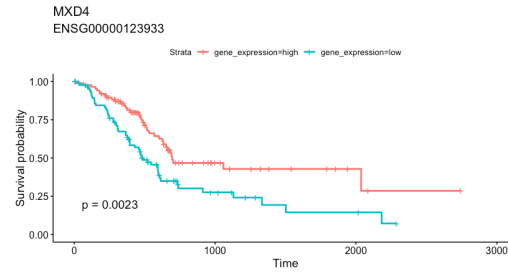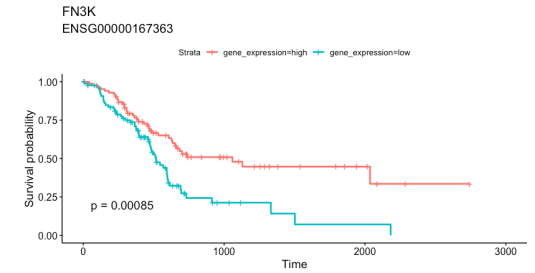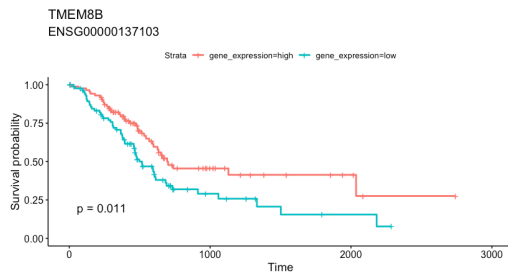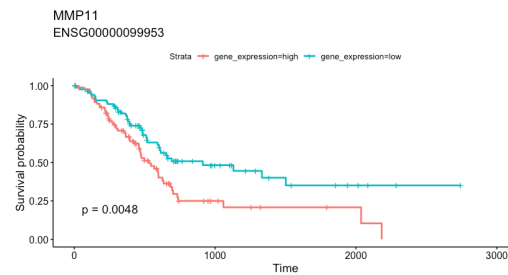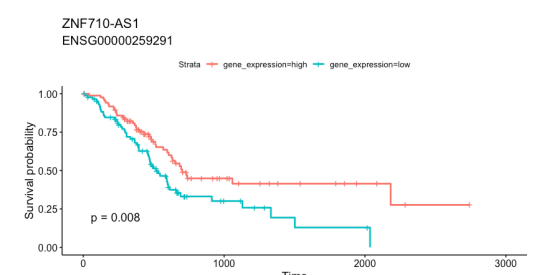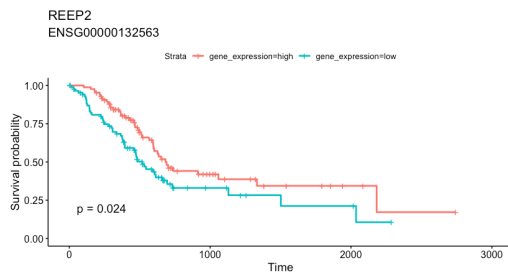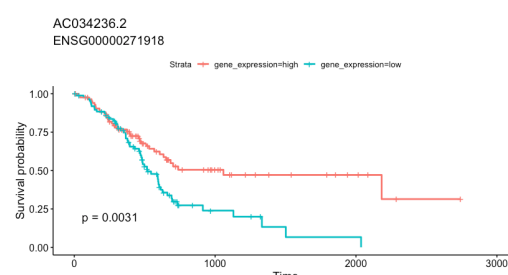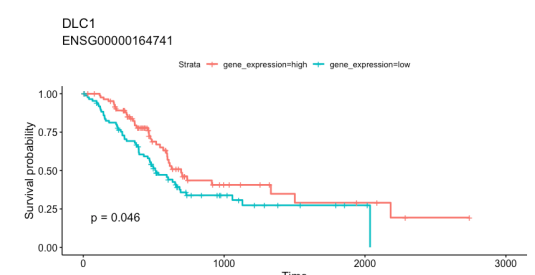

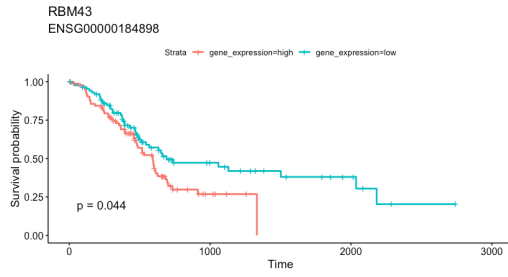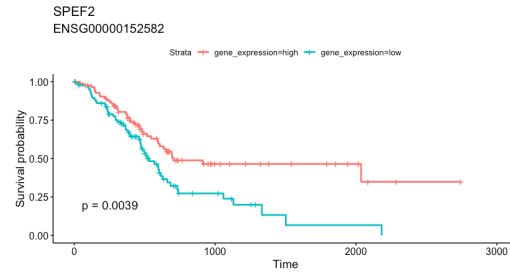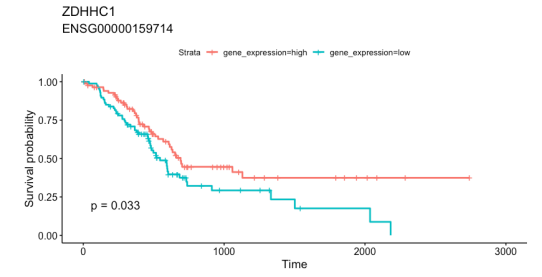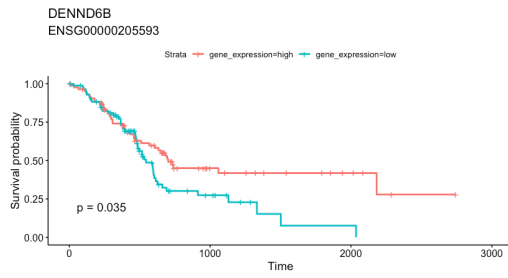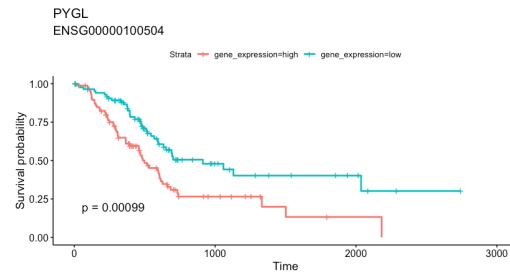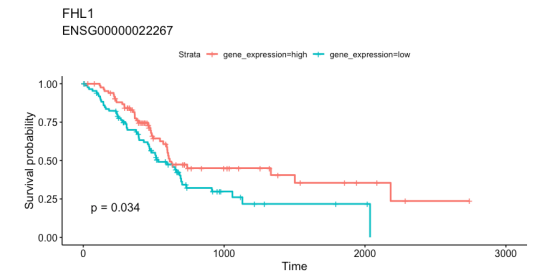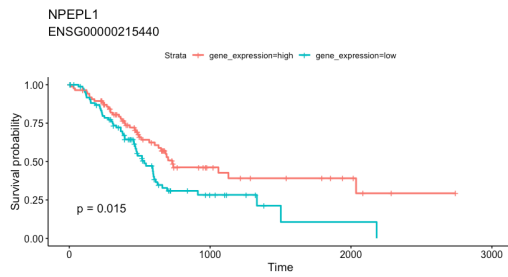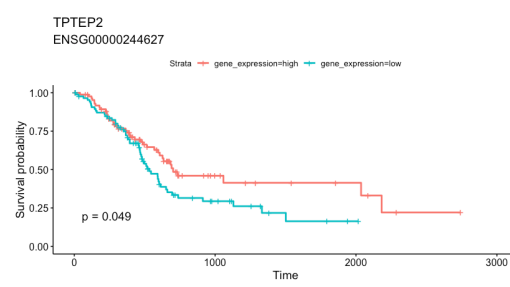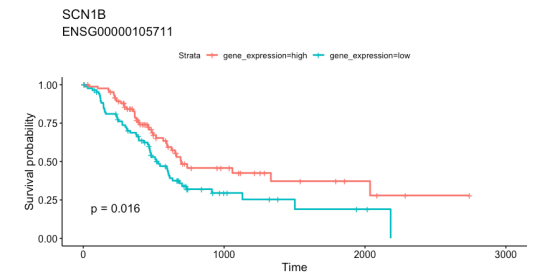

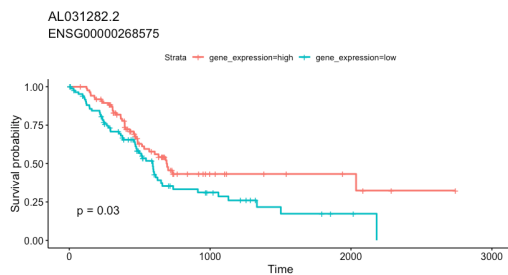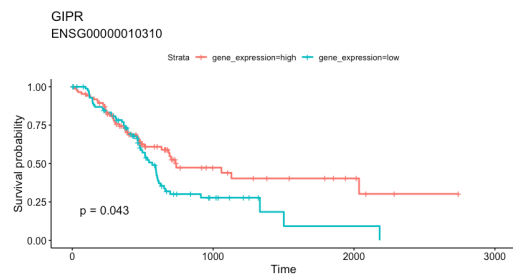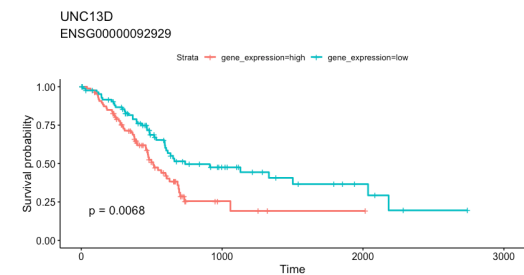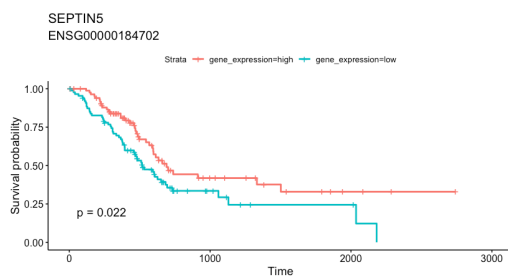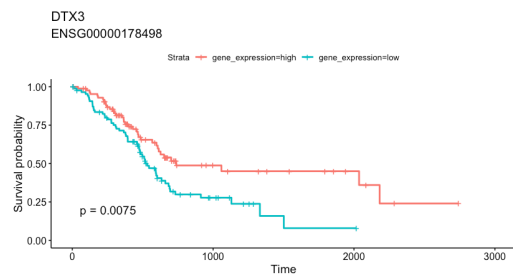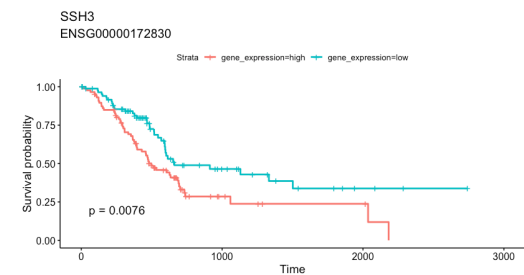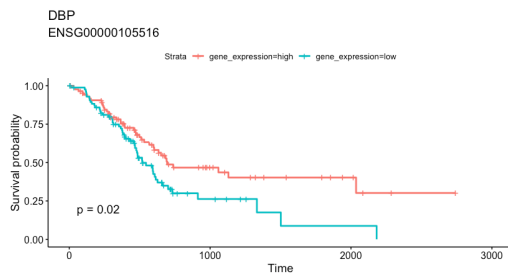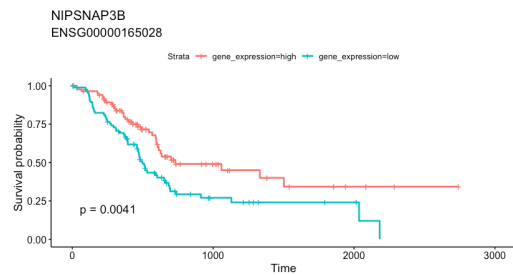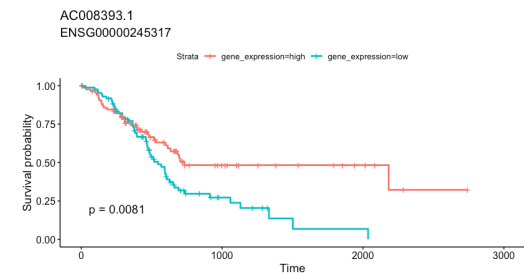

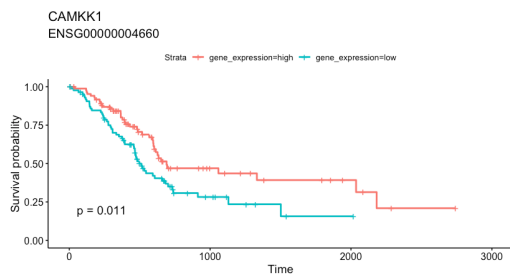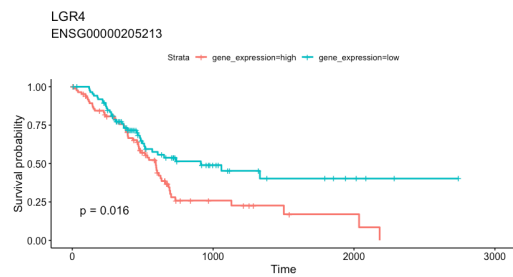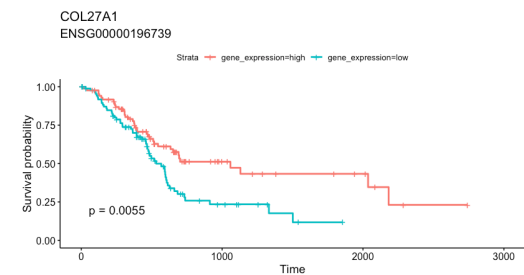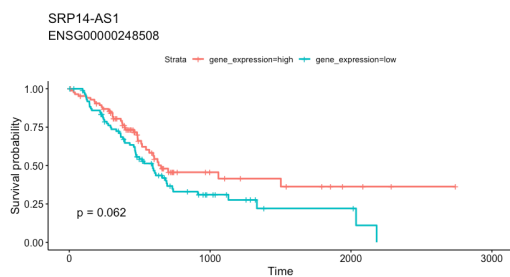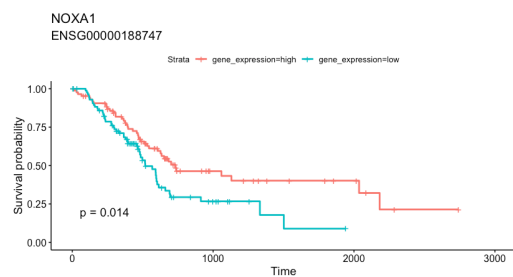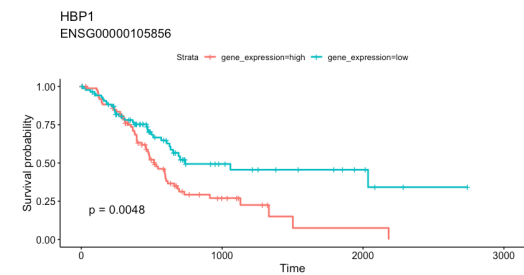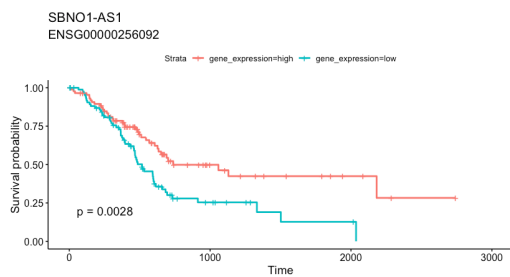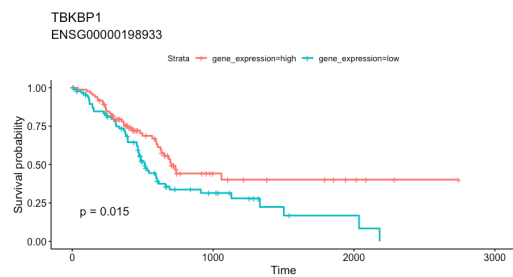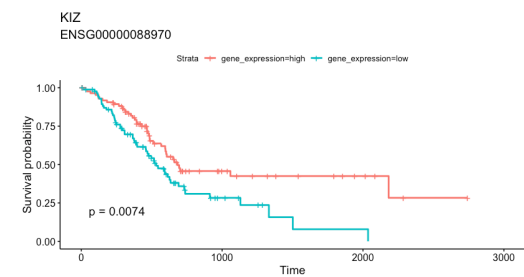

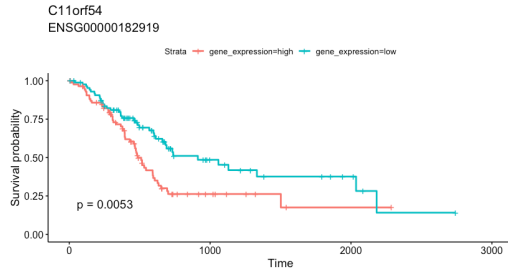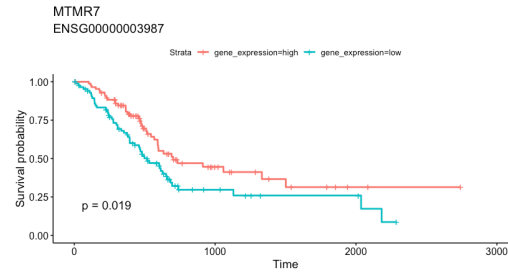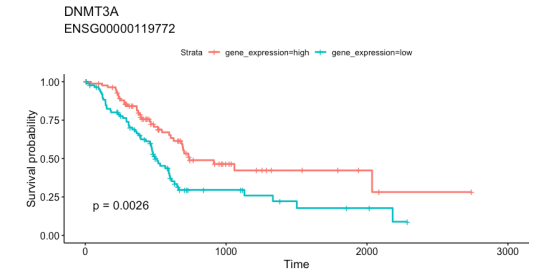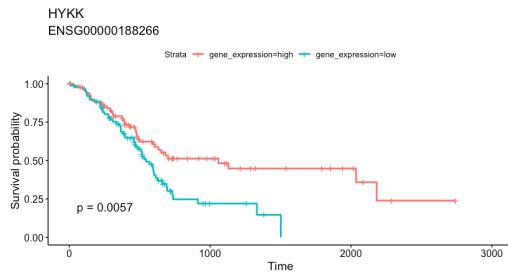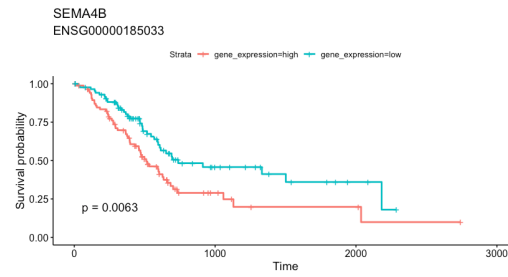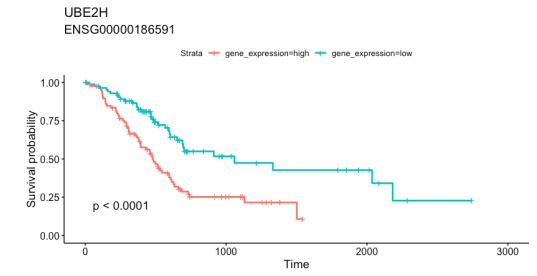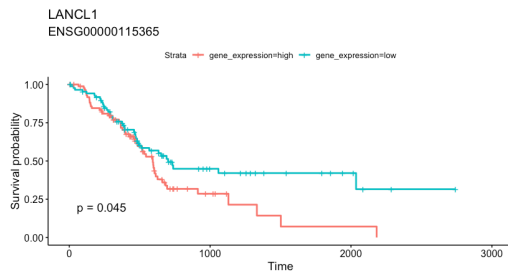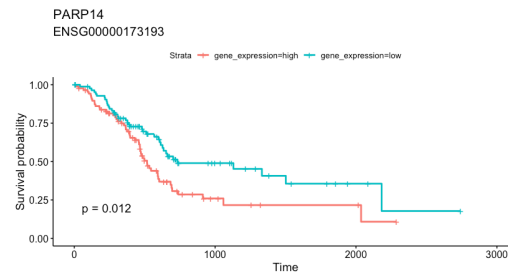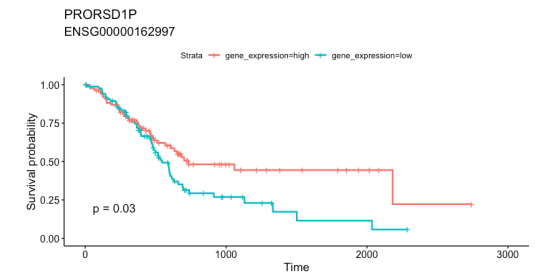

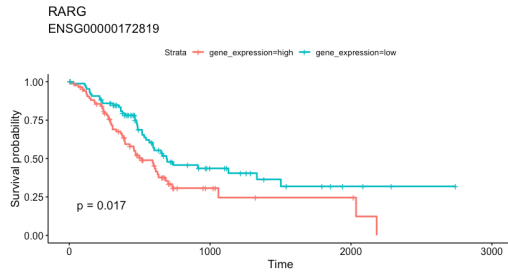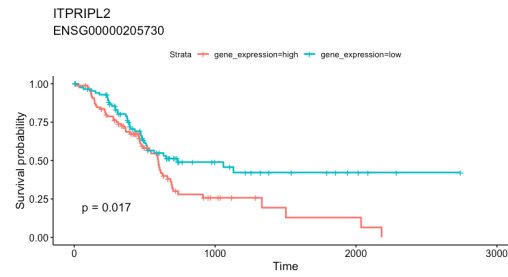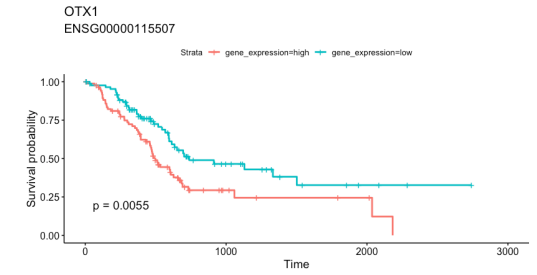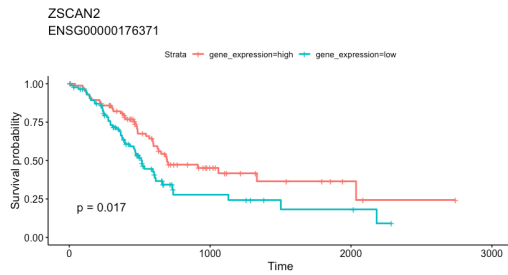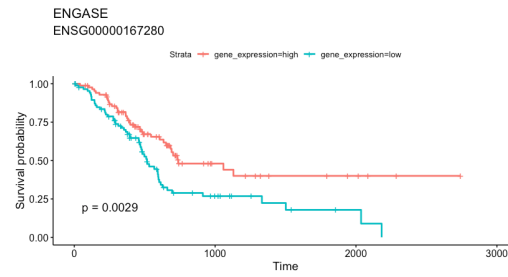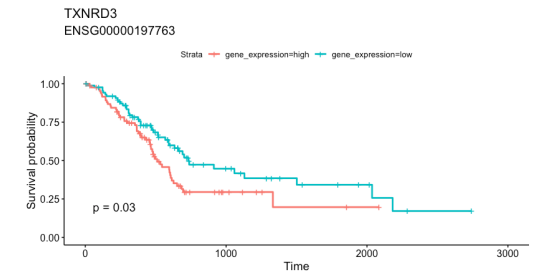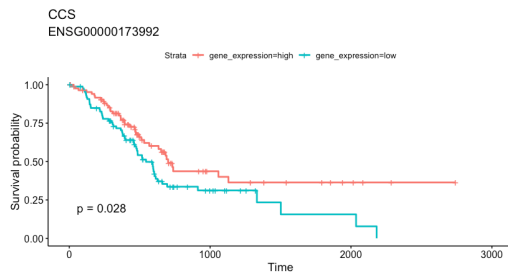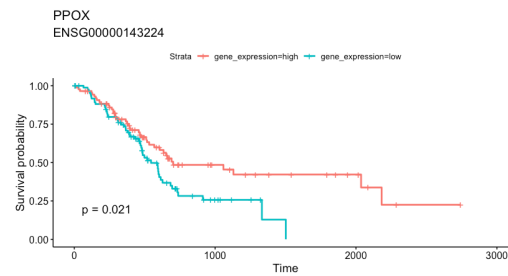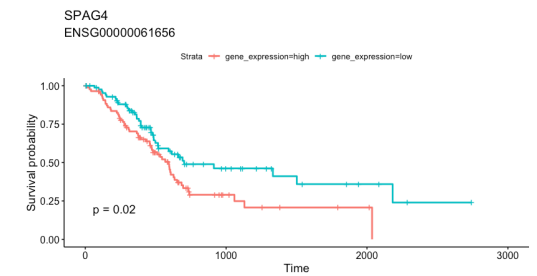

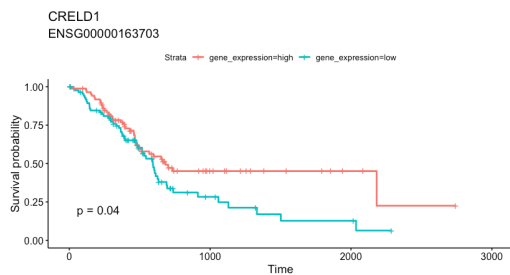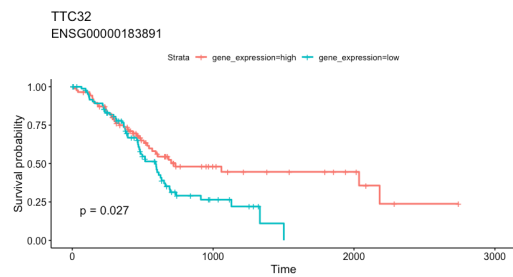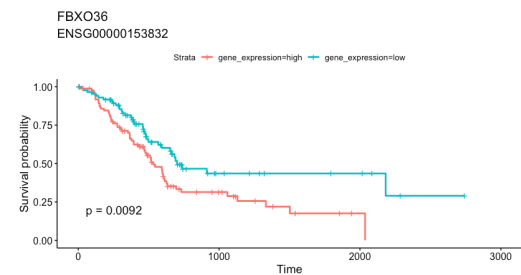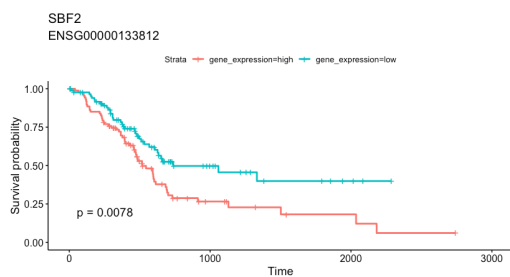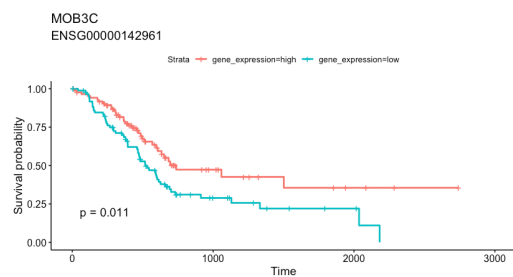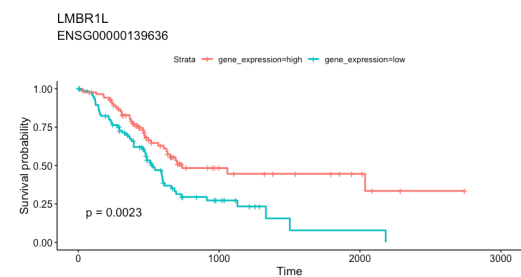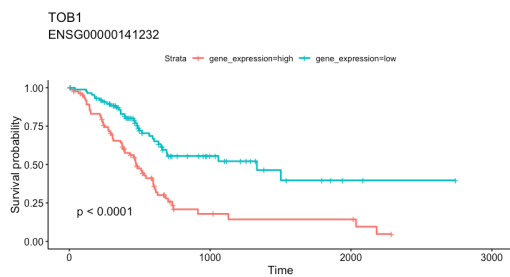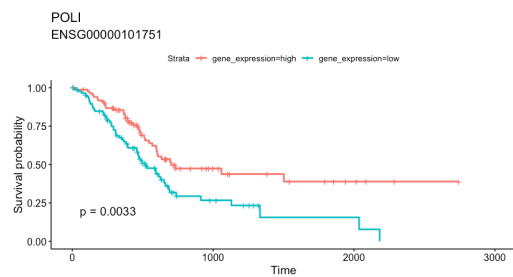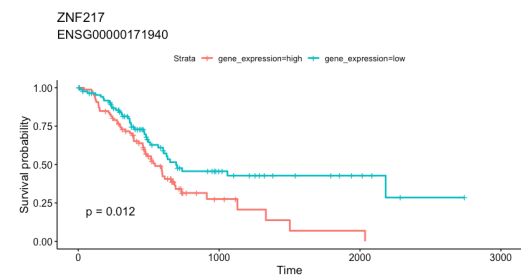

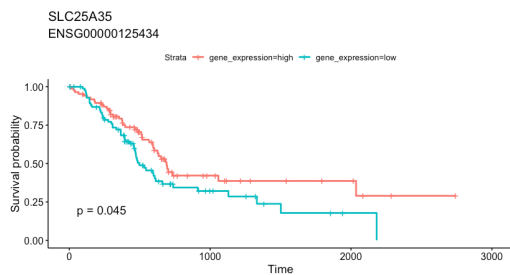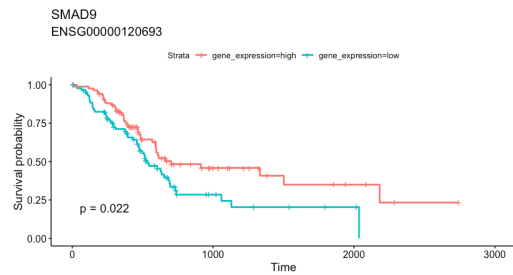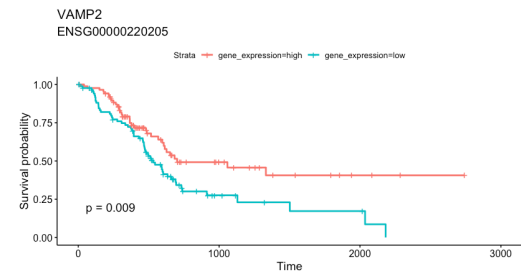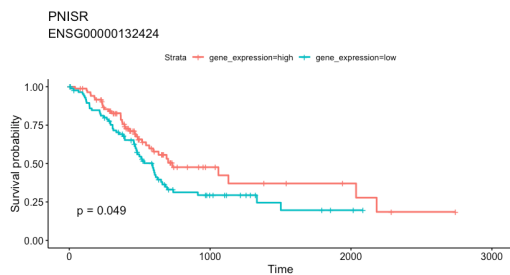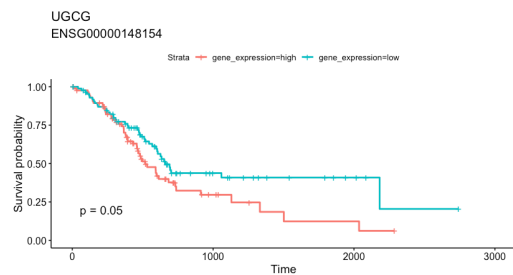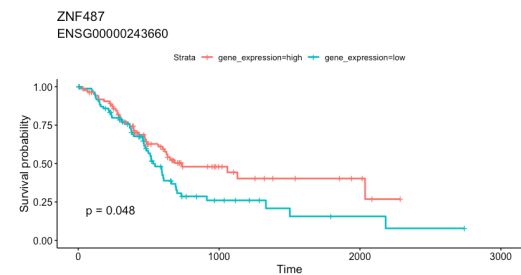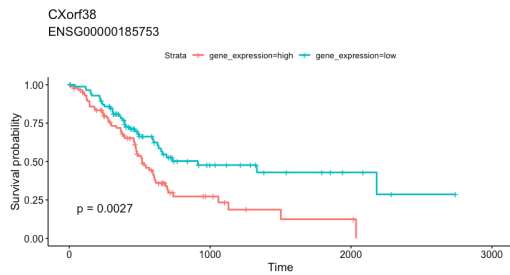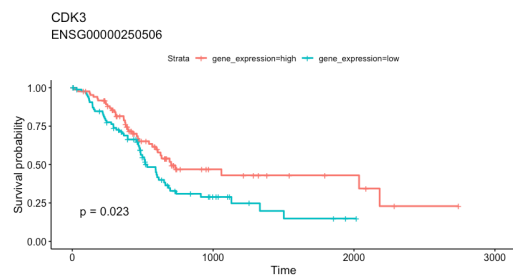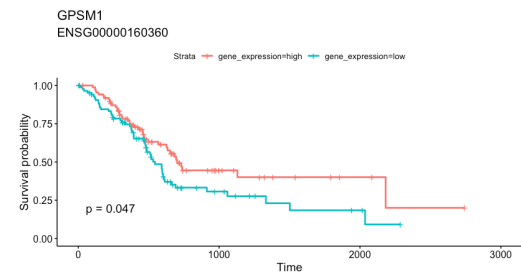

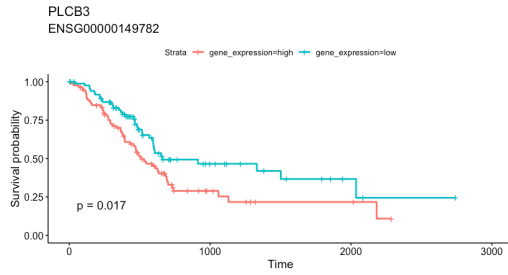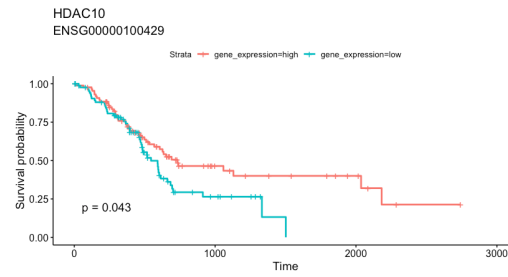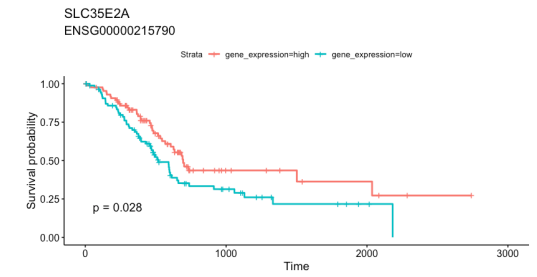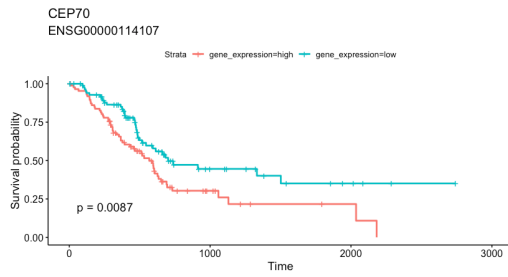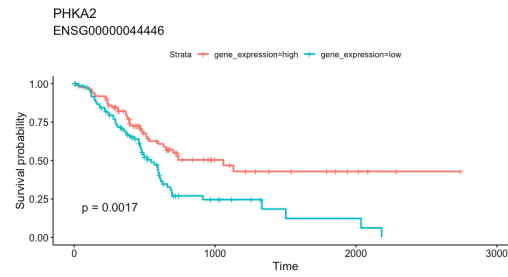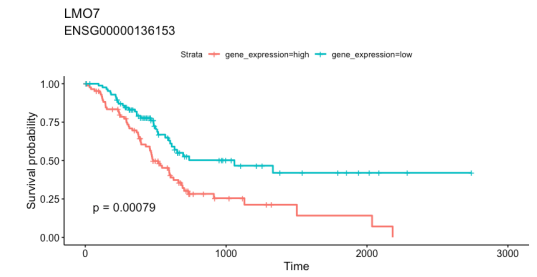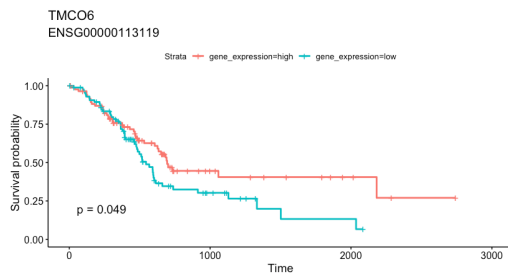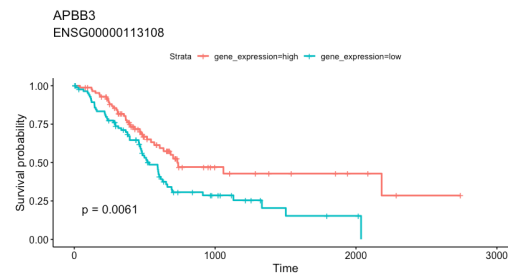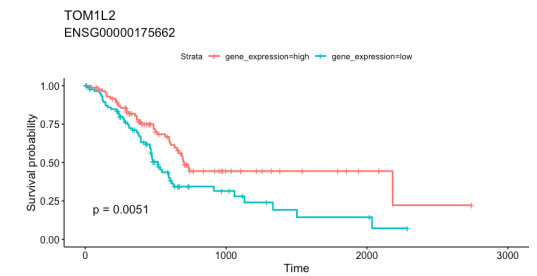

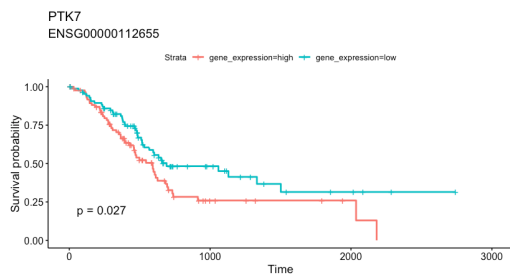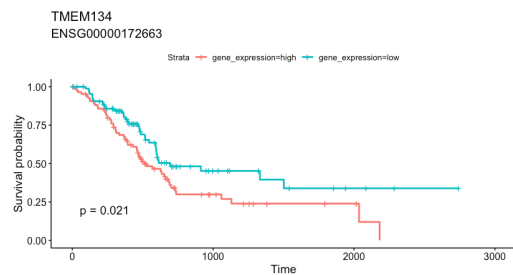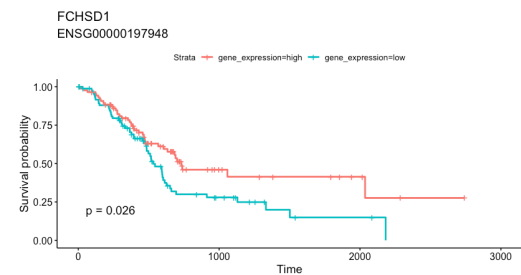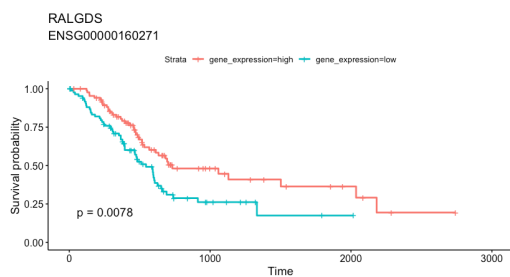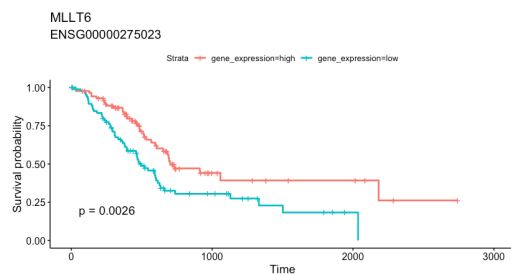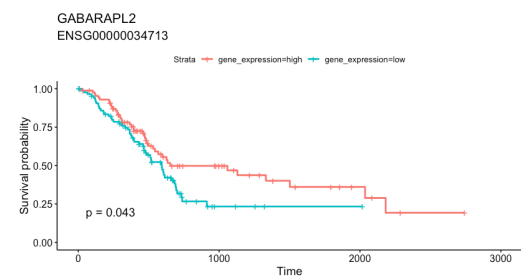

Supplement: Supplementary file 1 [file cancers-12-02183-s001.zip › cancers-855025-SUPPLE-XML/cancers-855025-supple-proof/Suppl_Fig1_pancreas_UPregulated_genes.pdf]

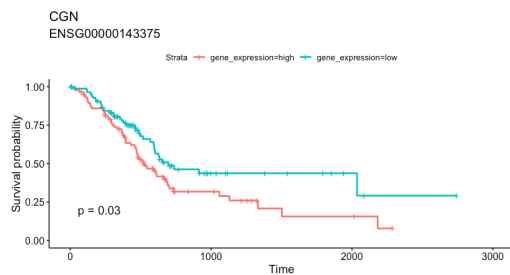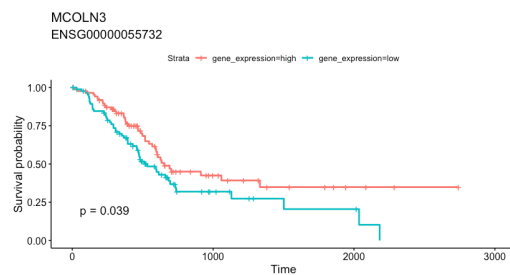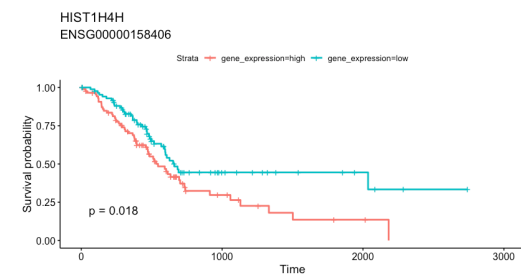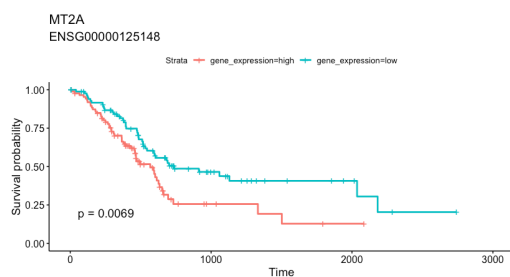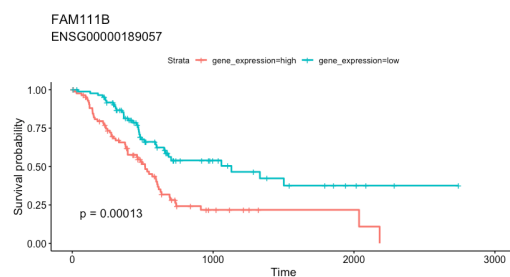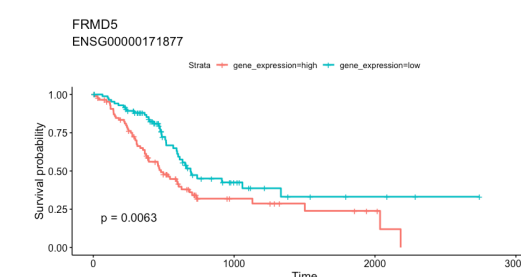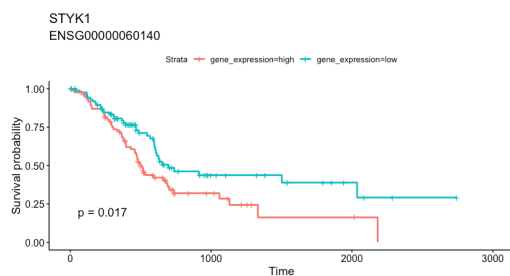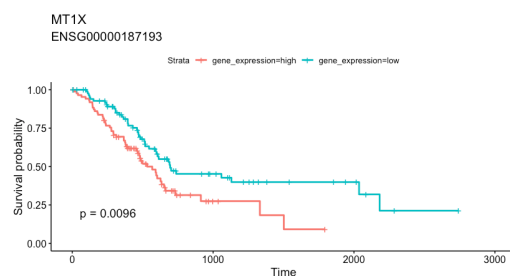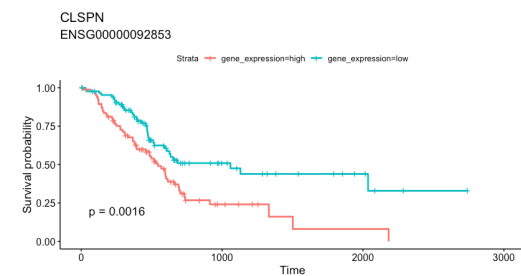

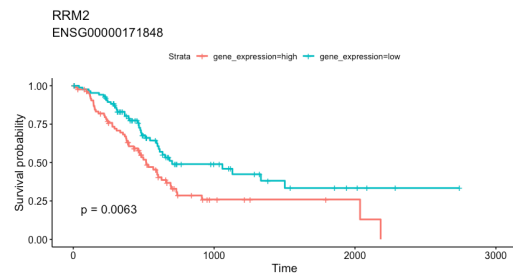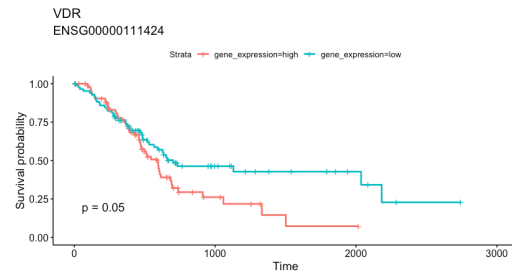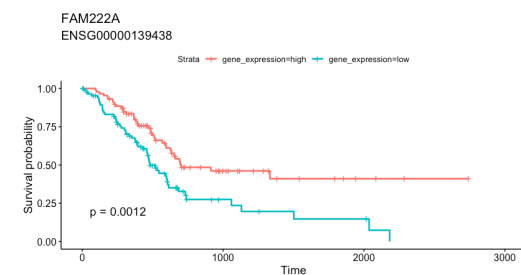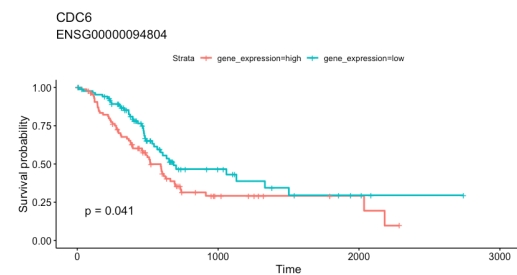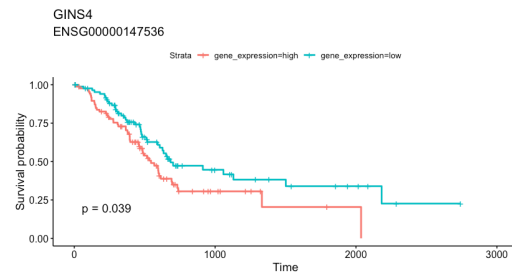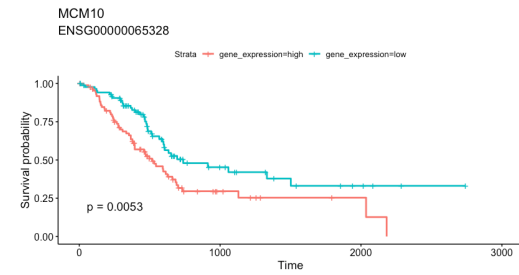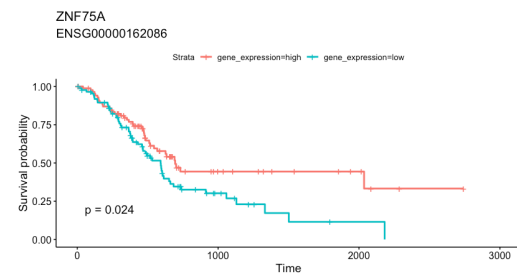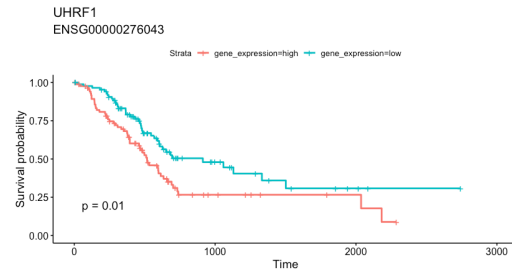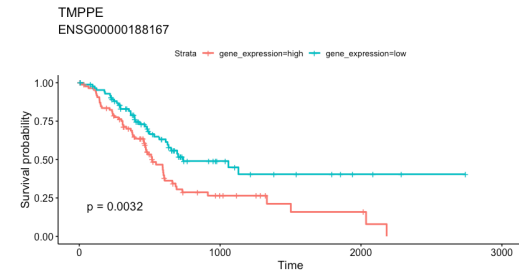

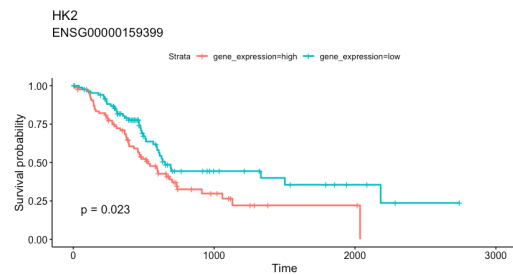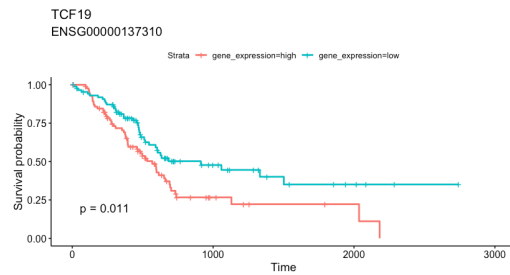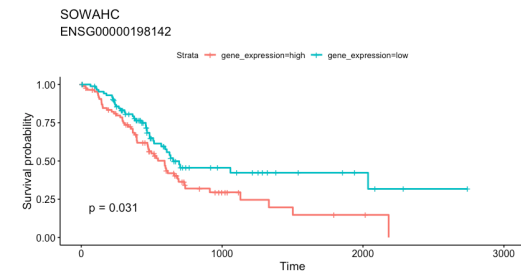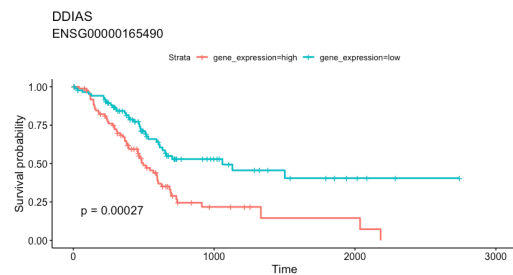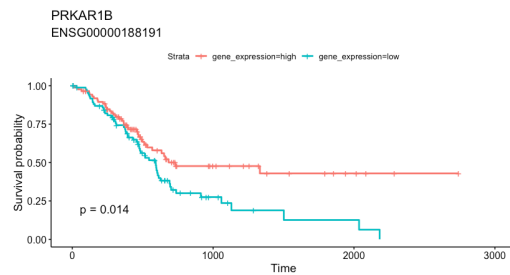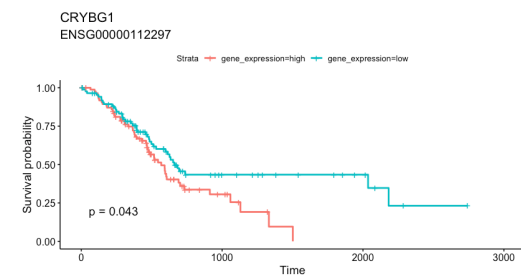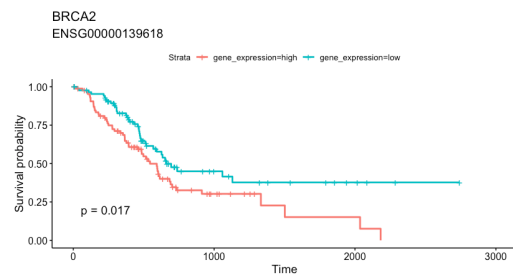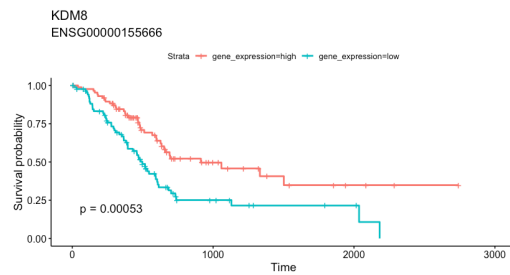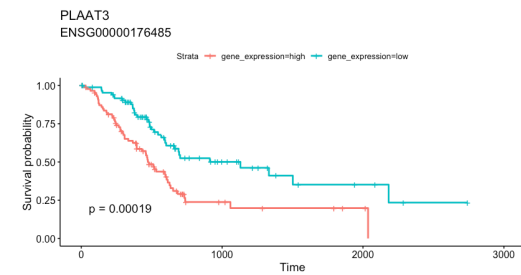

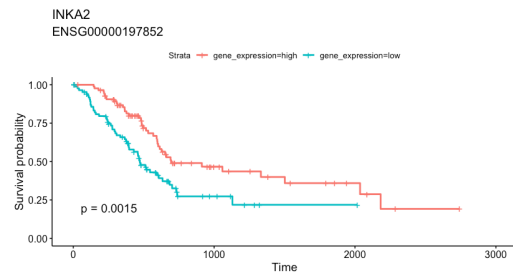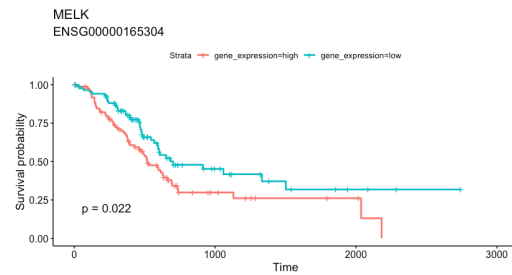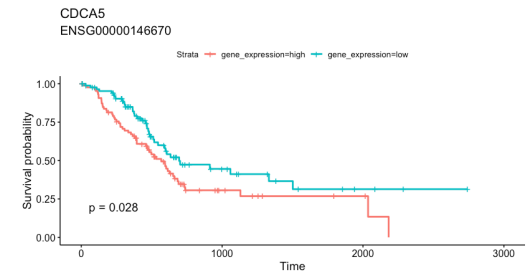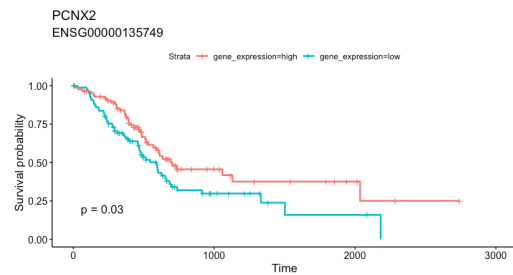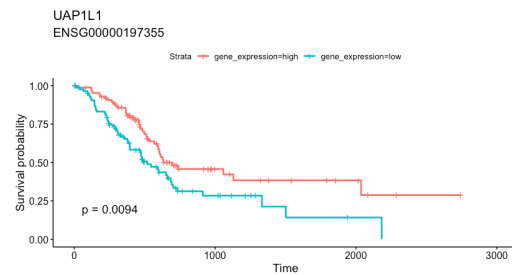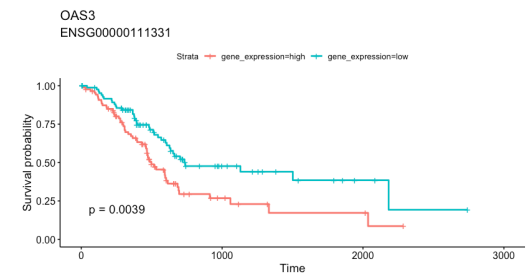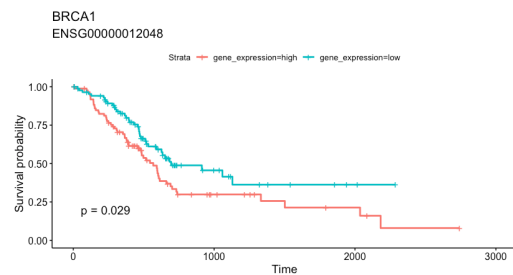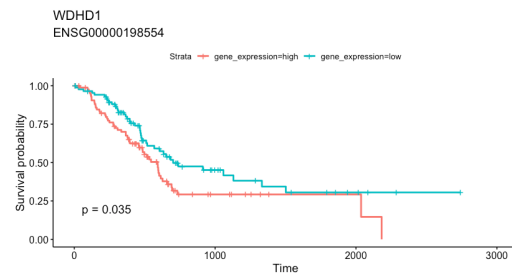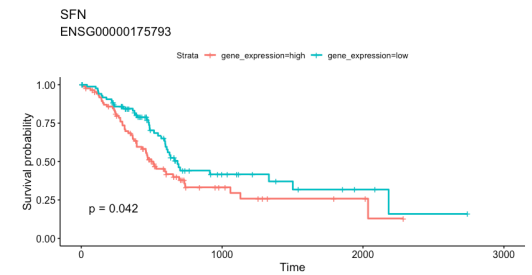

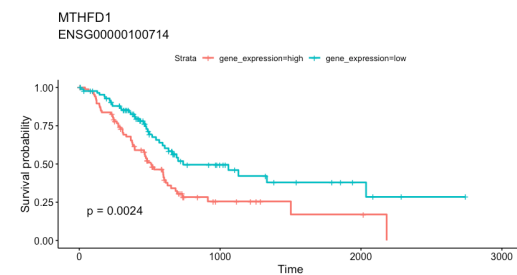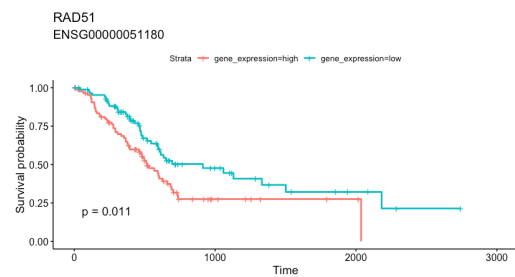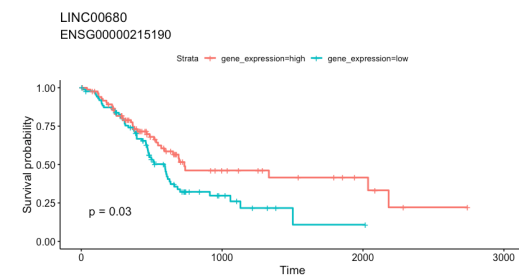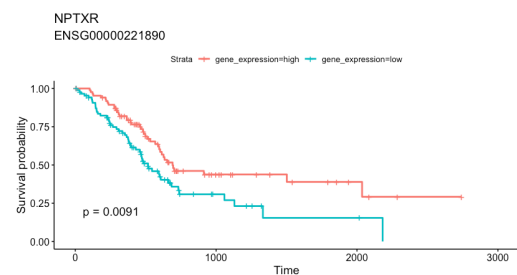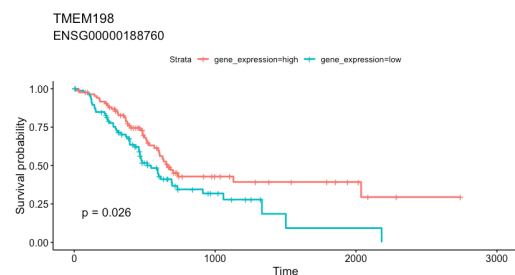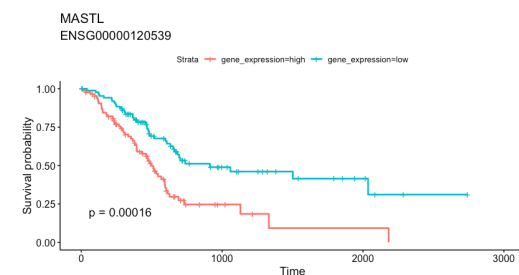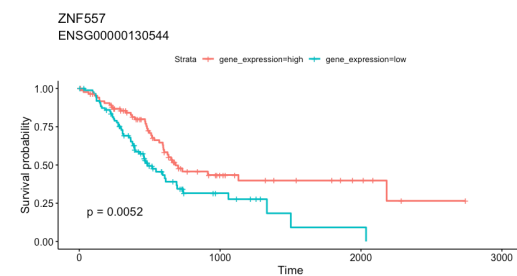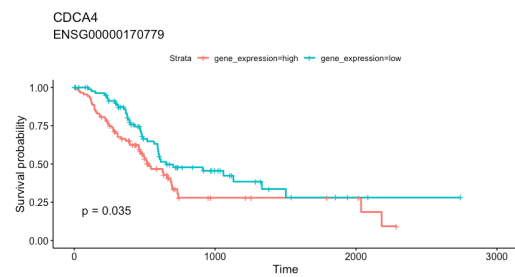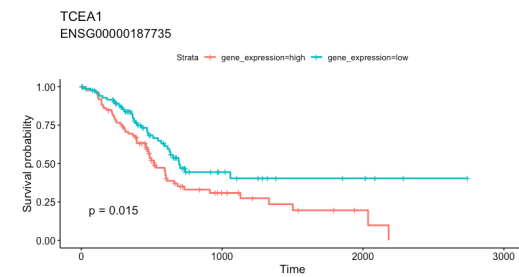

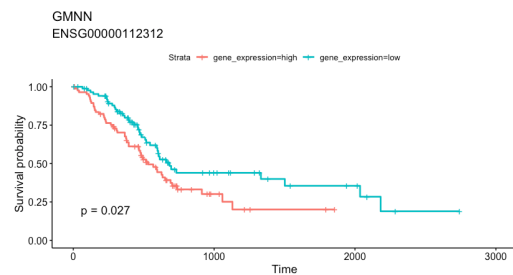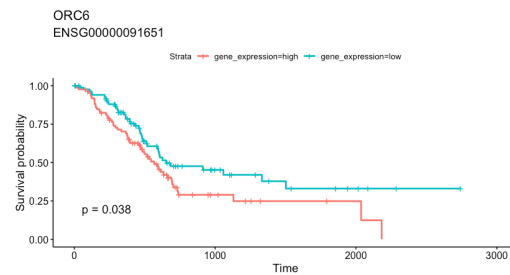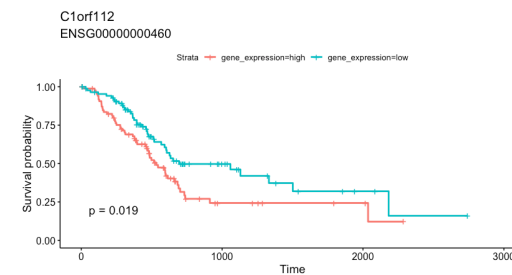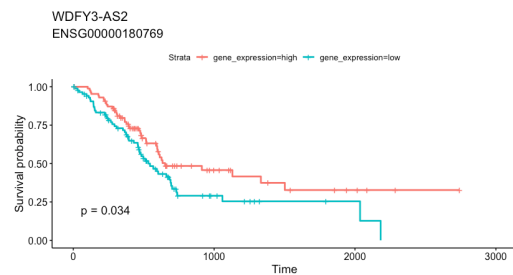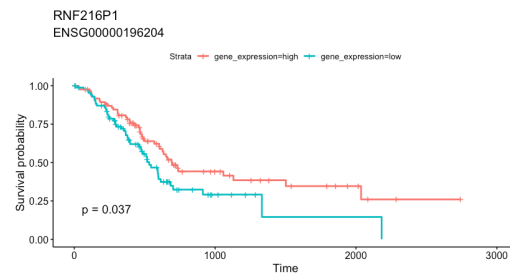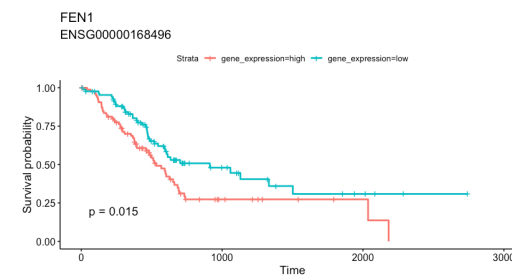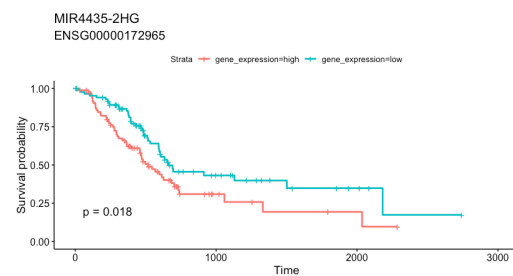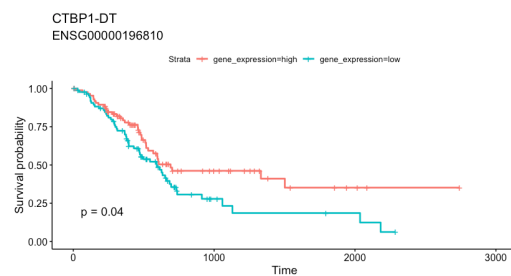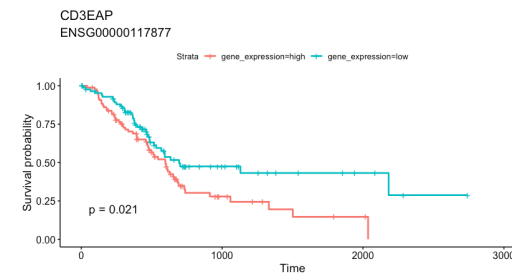

MCM4  
ENSG00000104738

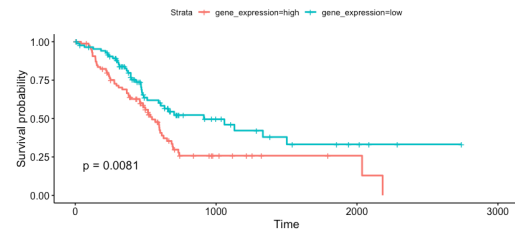

ZNF425  
ENSG00000204947

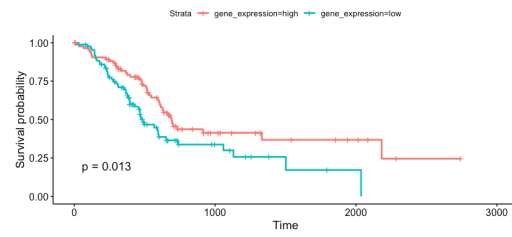

CHMP4A  
ENSG00000254505

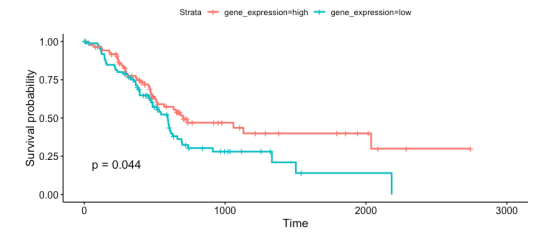

EIF6  
ENSG00000242372

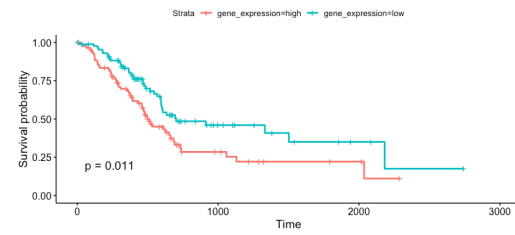

CBX6  
ENSG00000183741

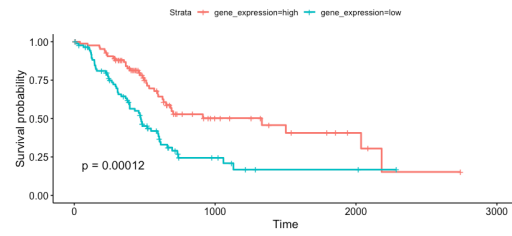

Supplement: Supplementary file 1 [file cancers-12-02183-s001.zip › cancers-855025-SUPPLE-XML/cancers-855025-supple-proof/Suppl_Fig2_pancreas_DOWNregulated_genes.pdf]

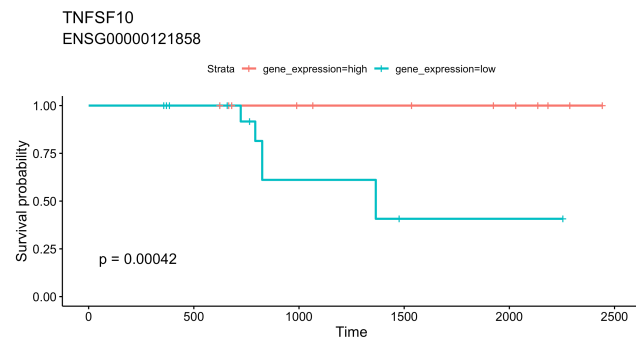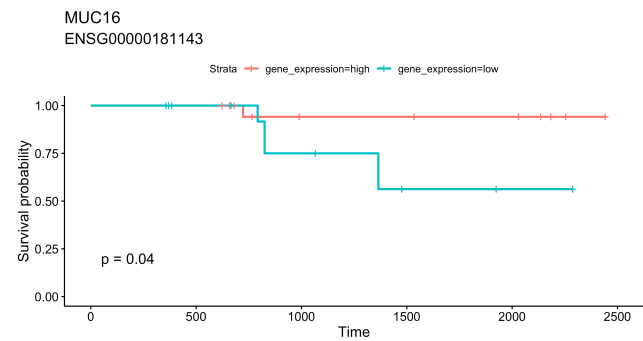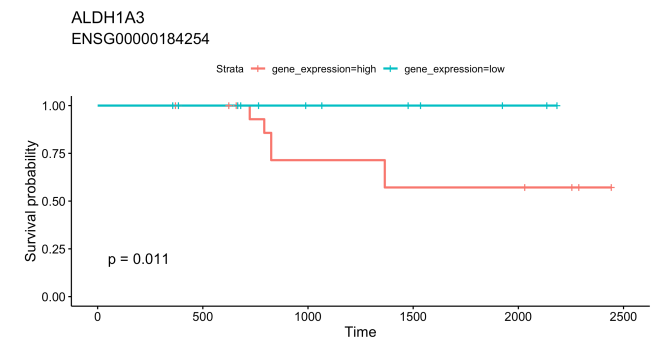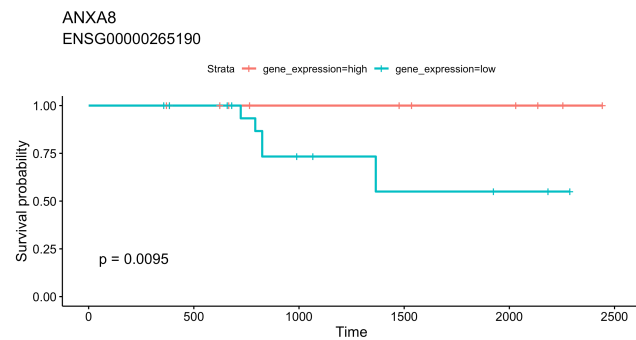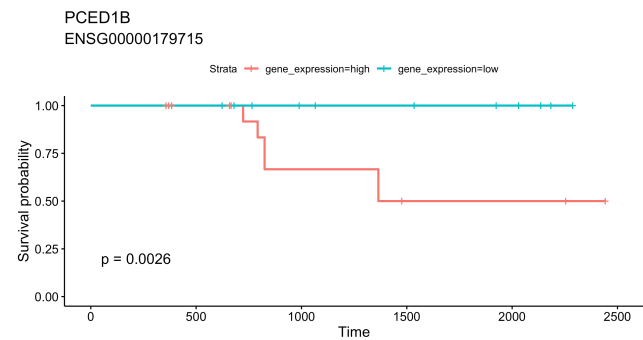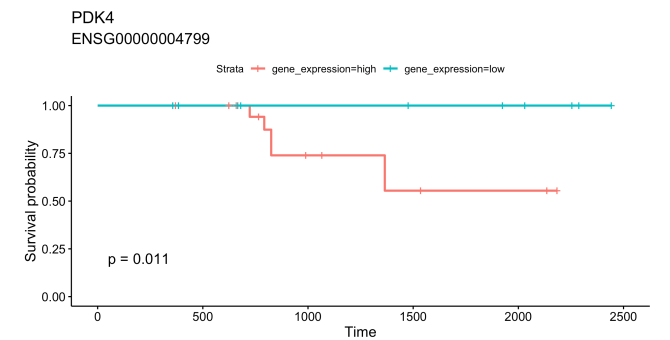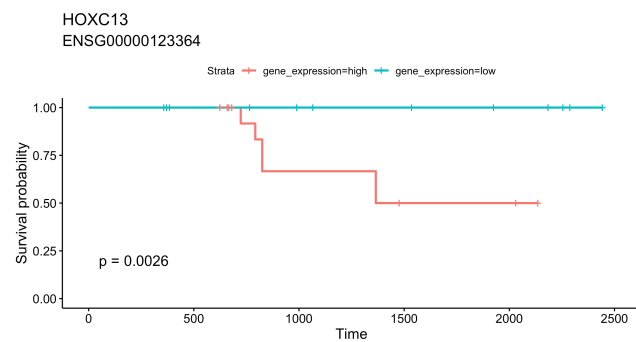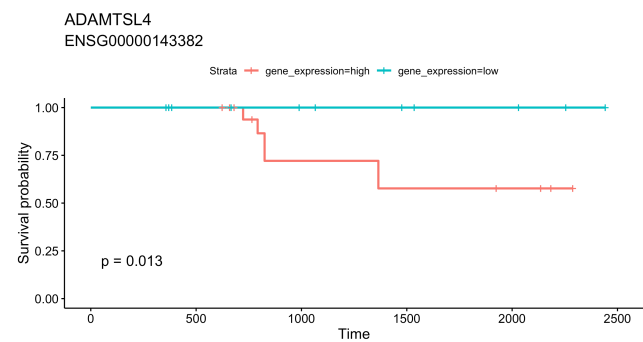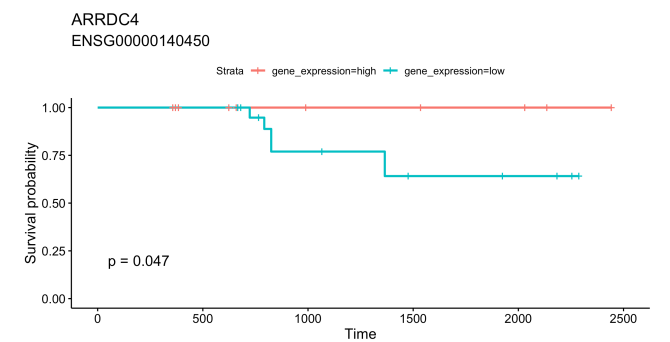

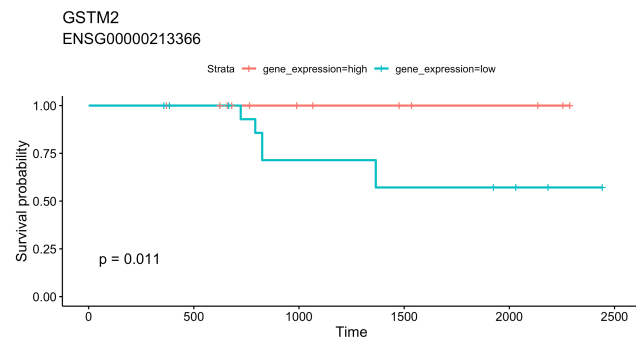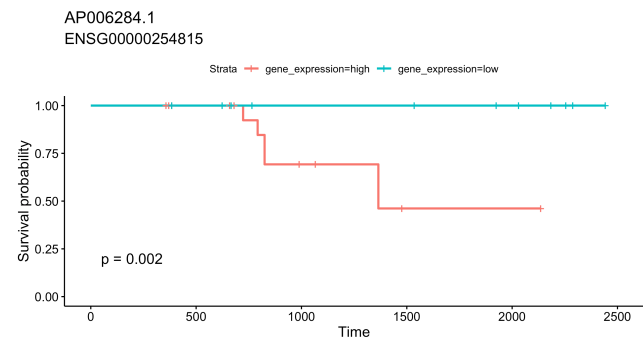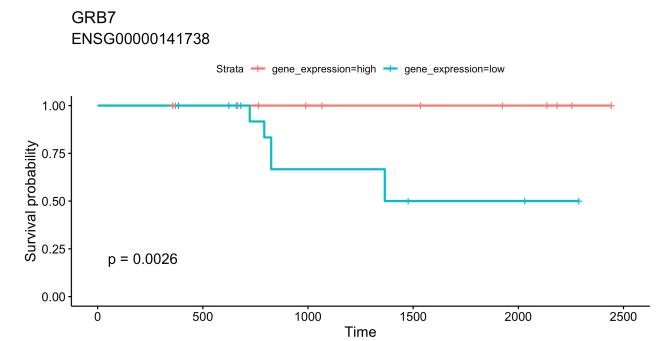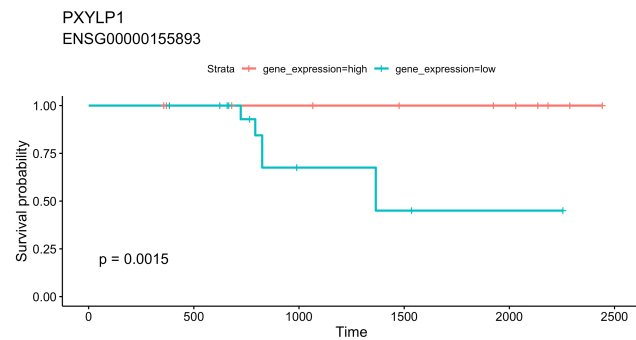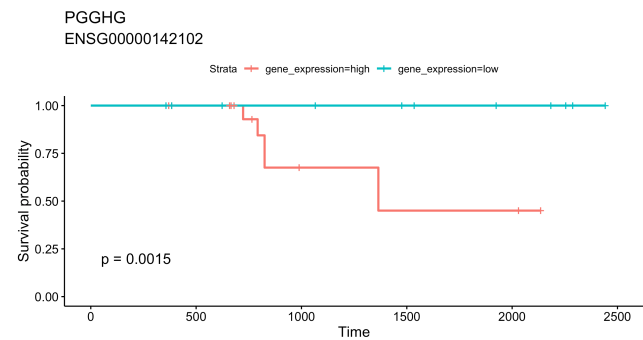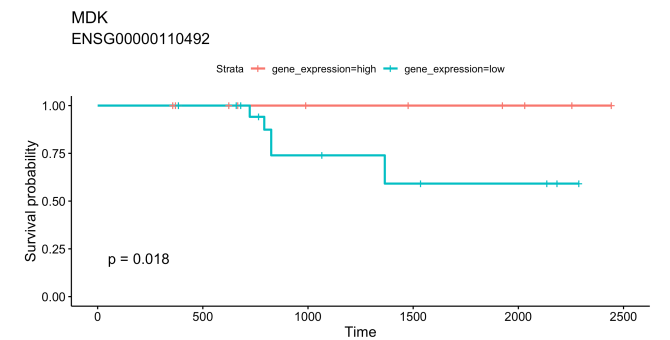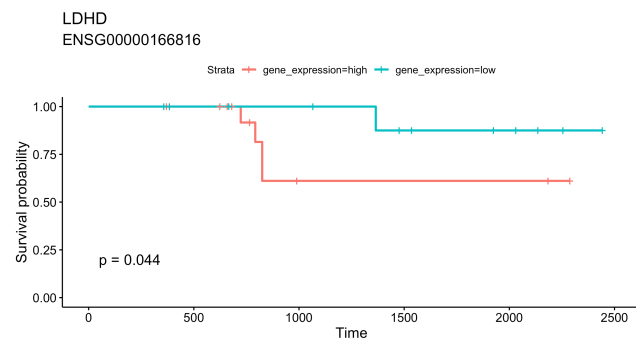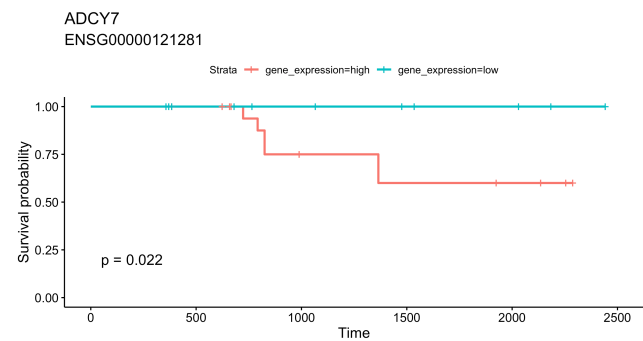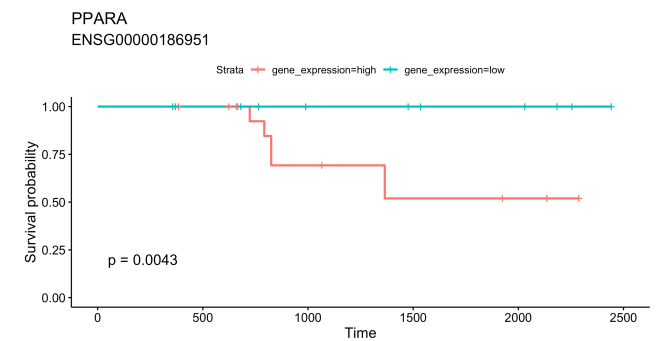

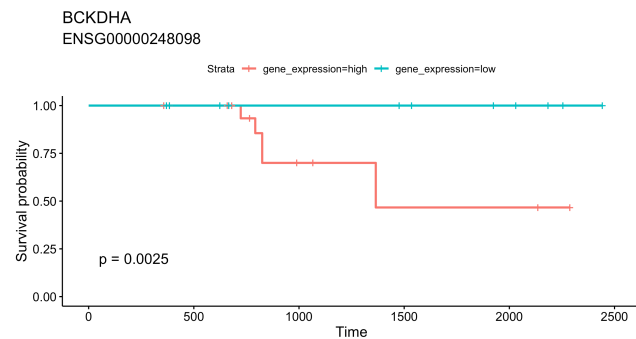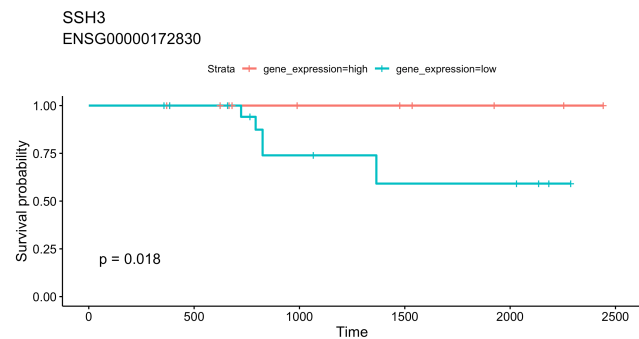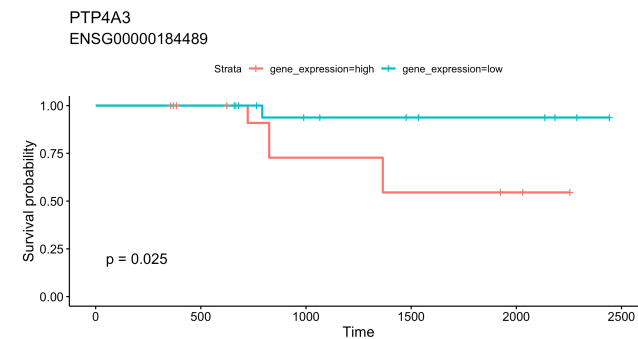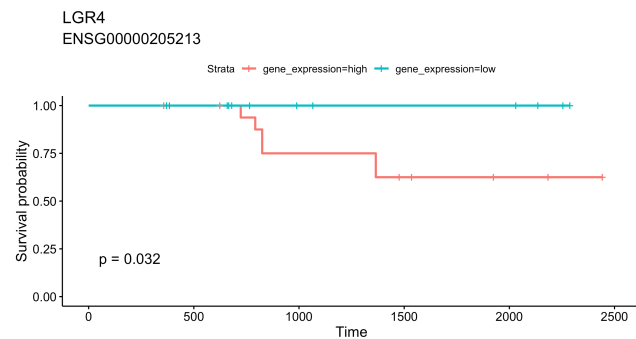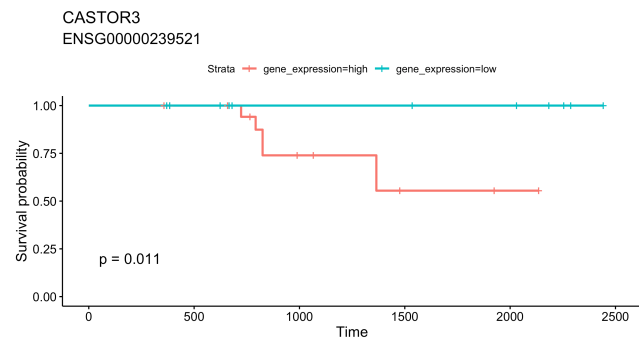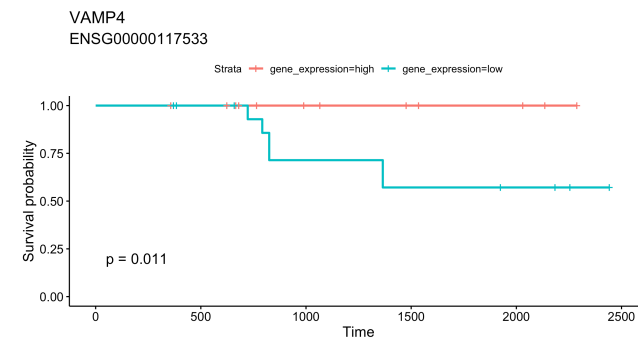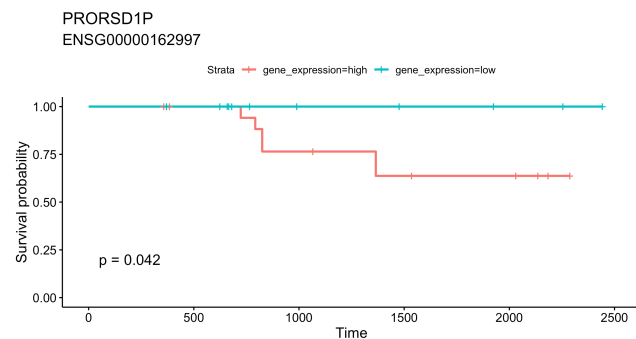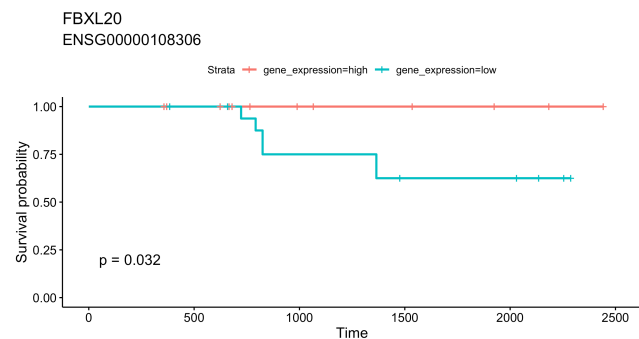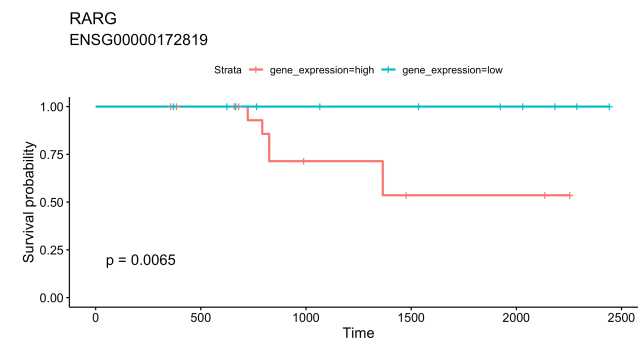

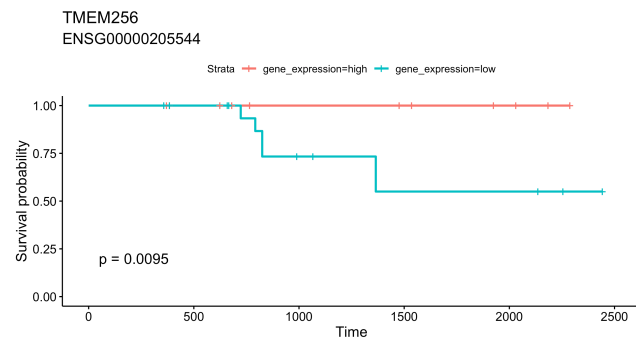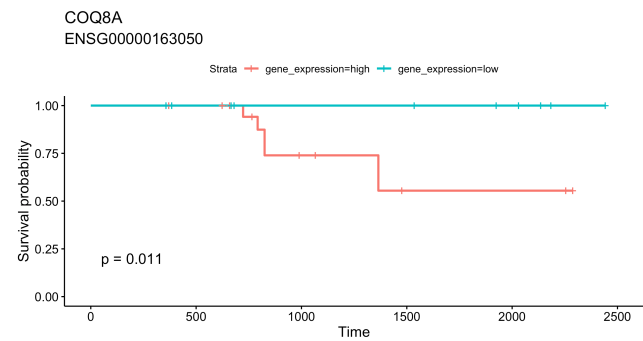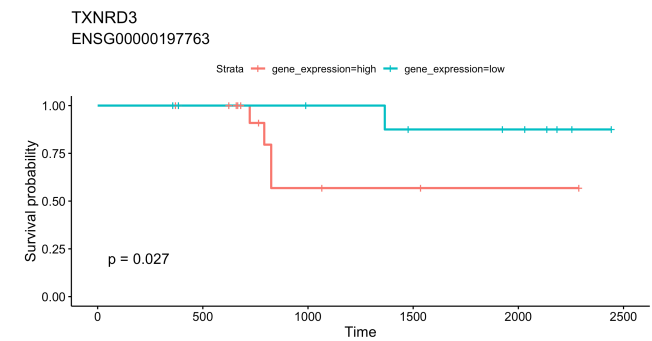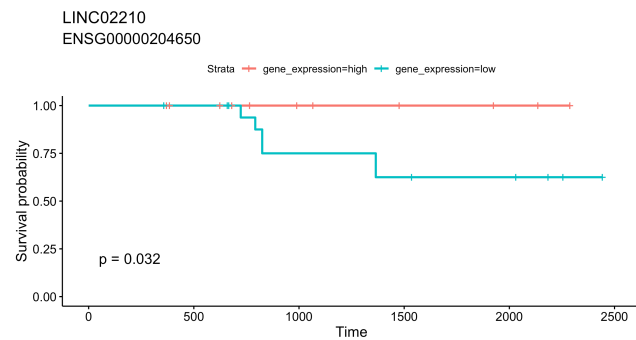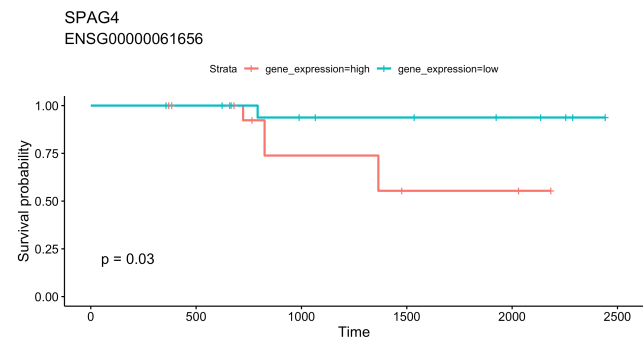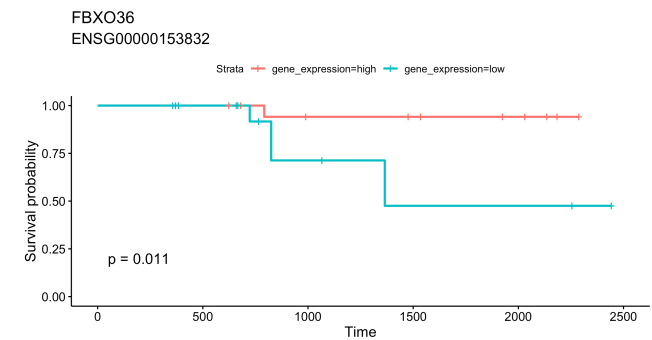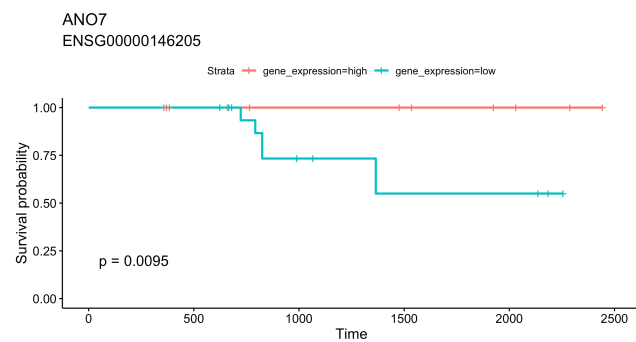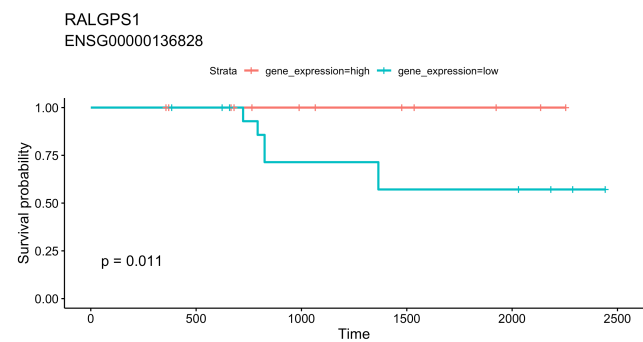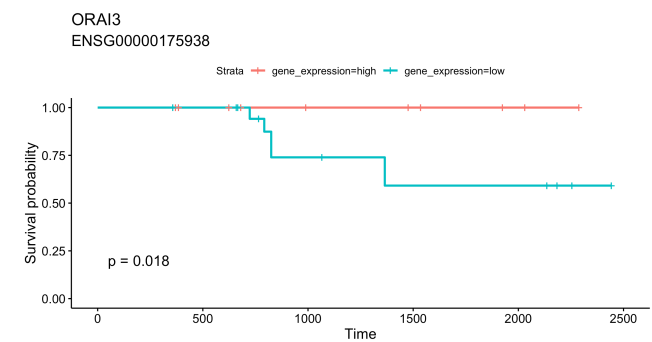

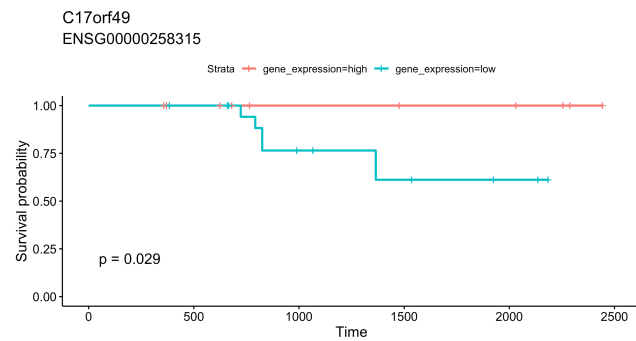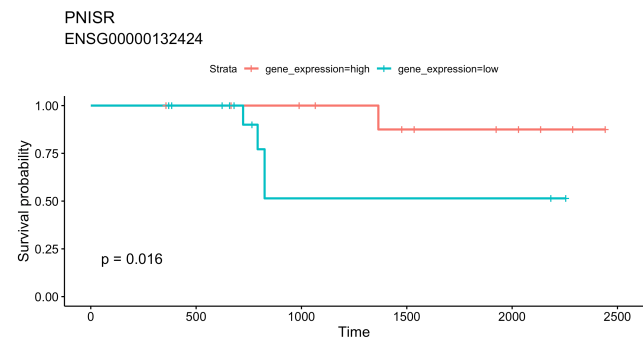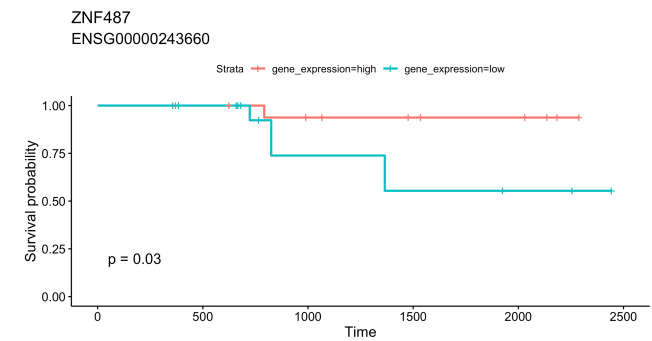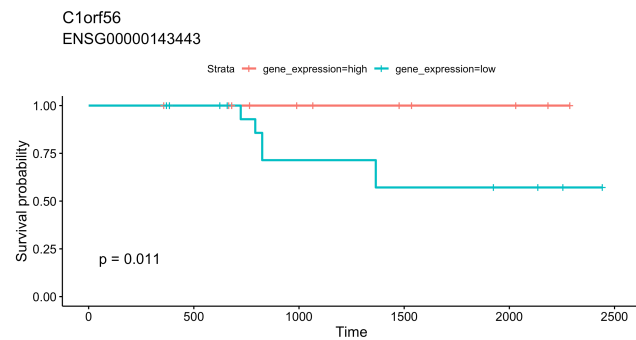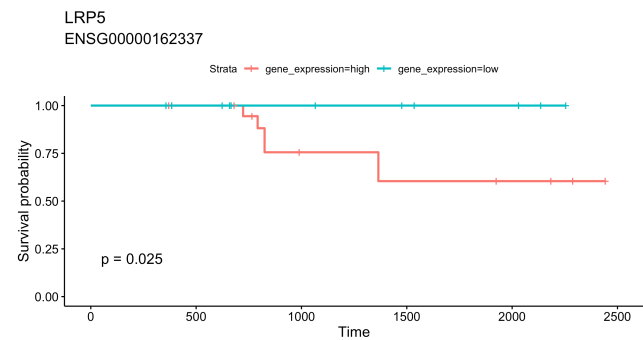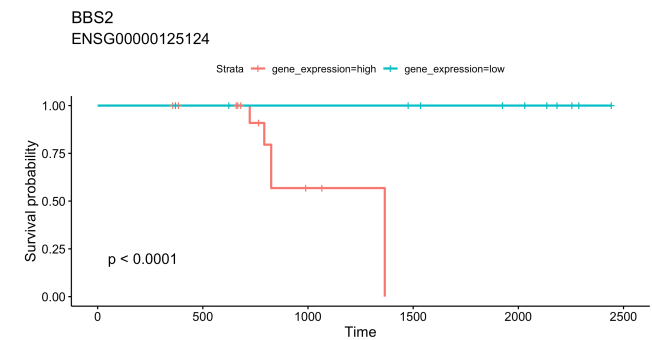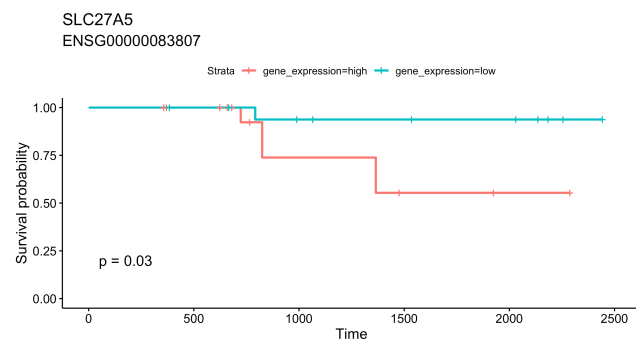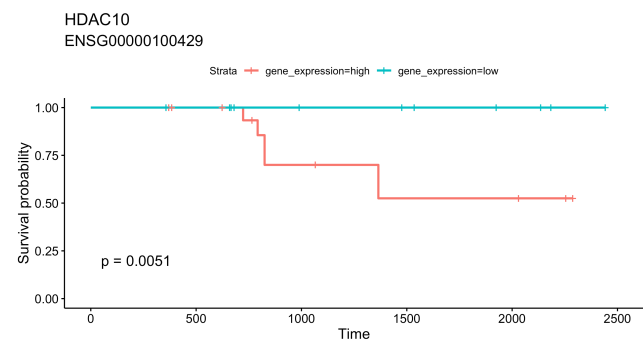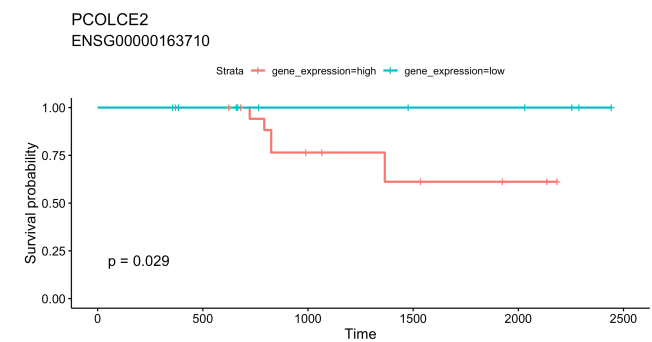

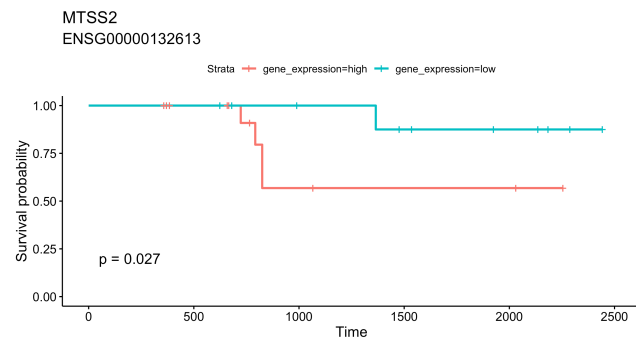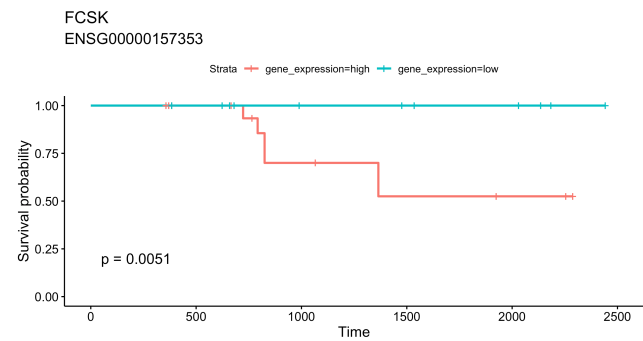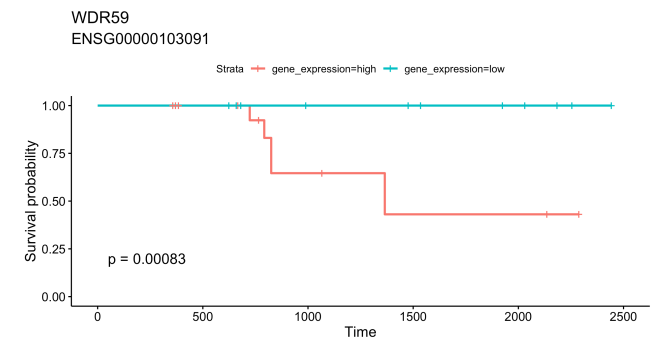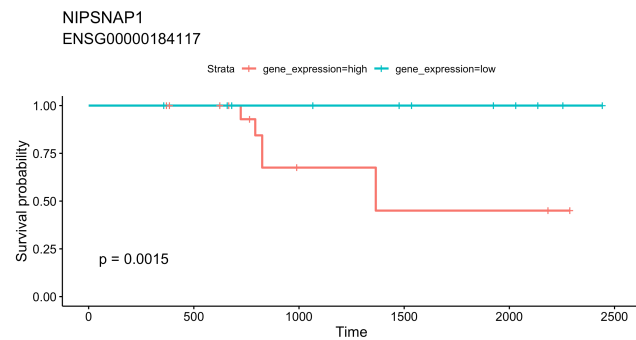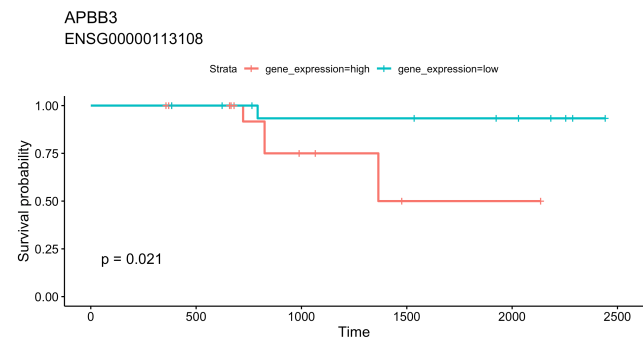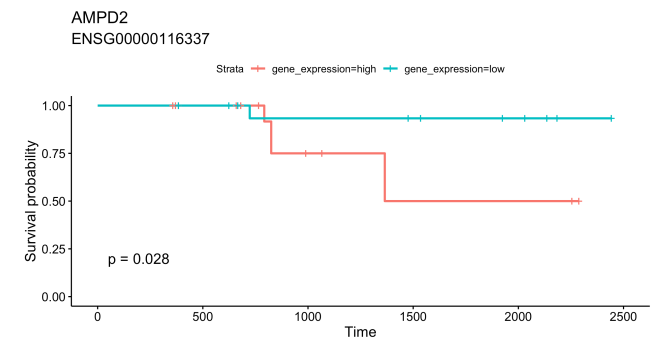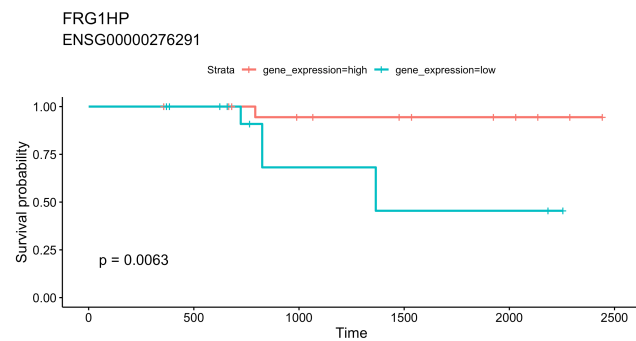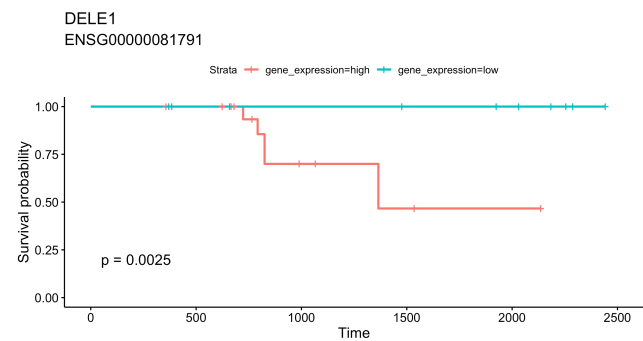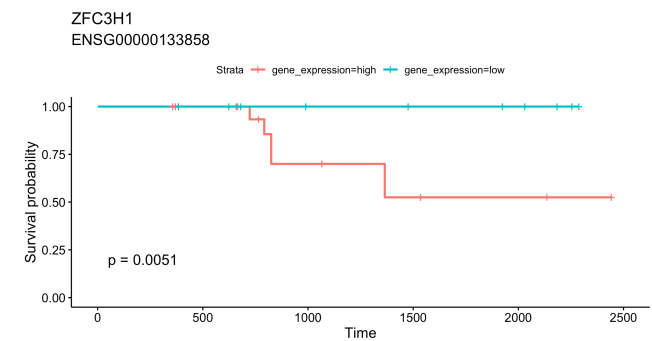

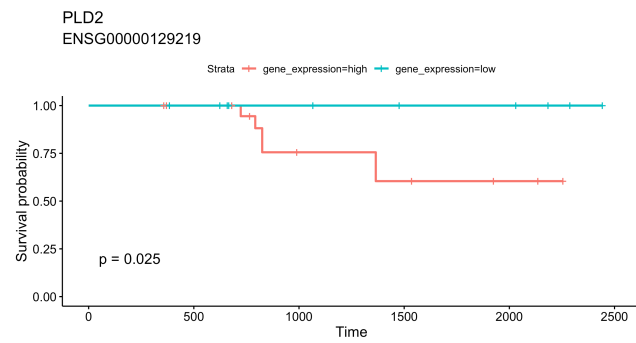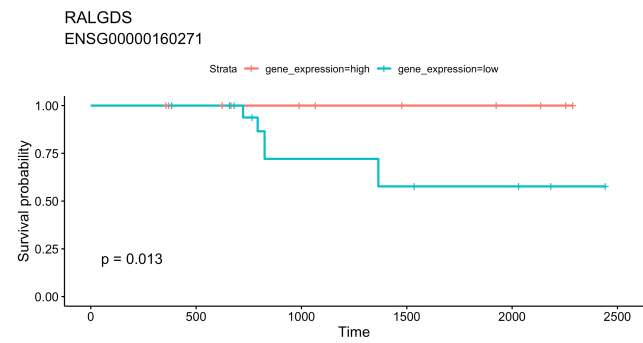

Supplement: Supplementary file 1 [file cancers-12-02183-s001.zip › cancers-855025-SUPPLE-XML/cancers-855025-supple-proof/Suppl_Fig3_breast_LumB_UPregulated_genes.pdf]

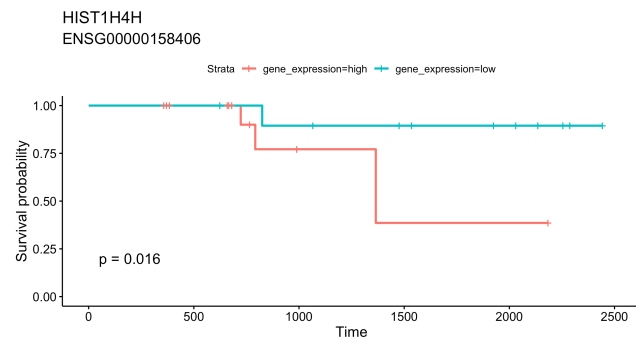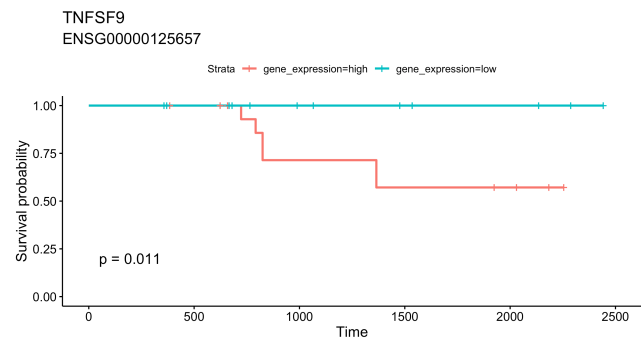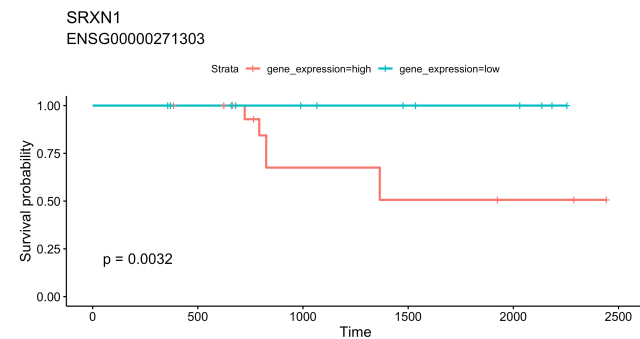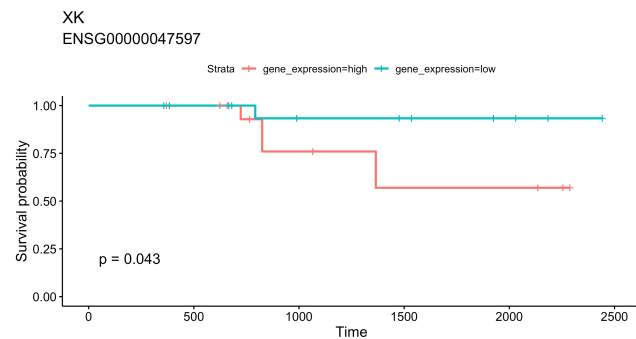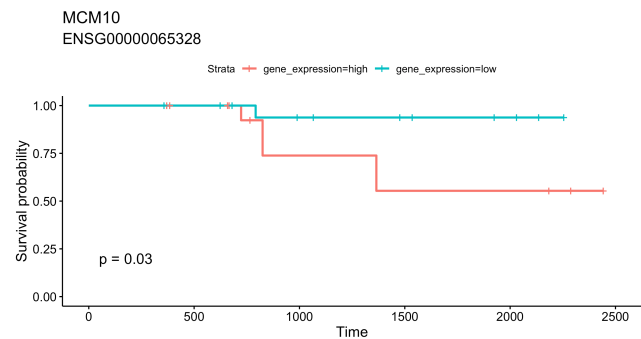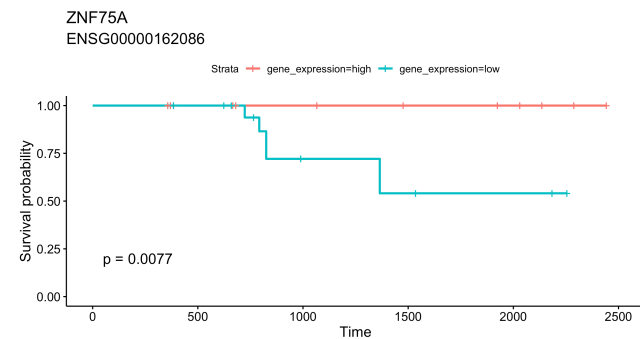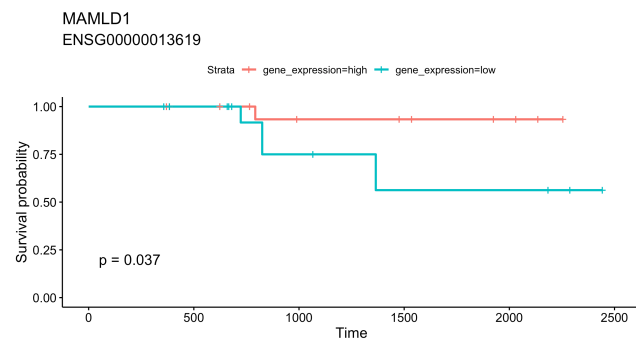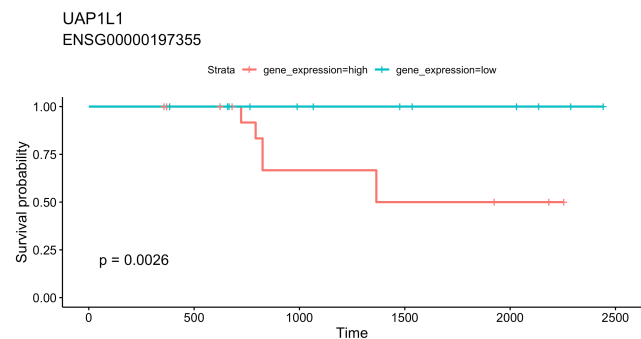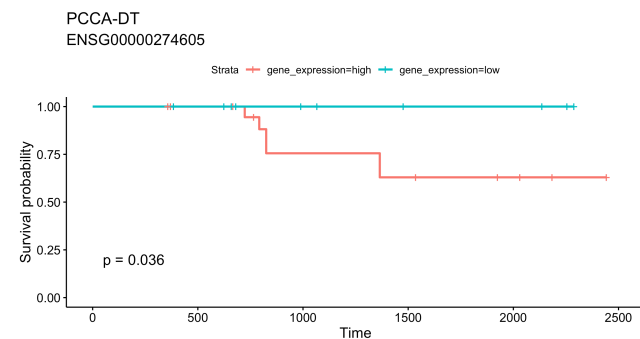

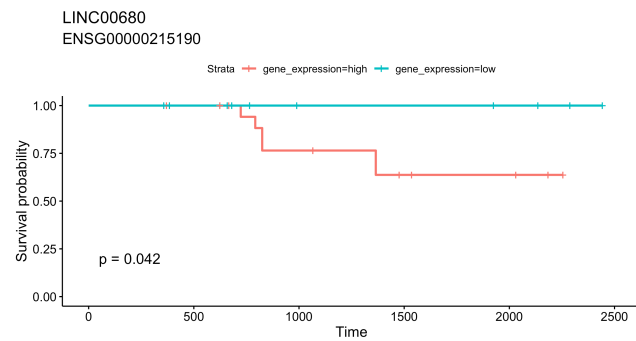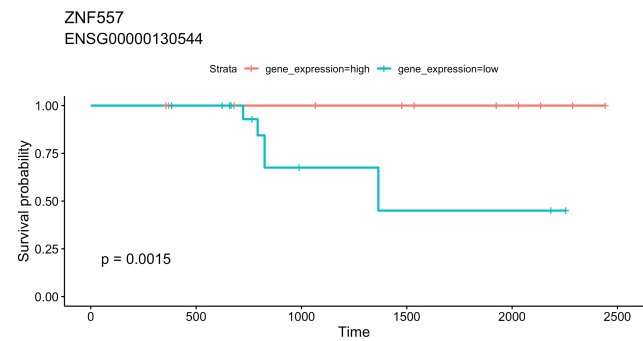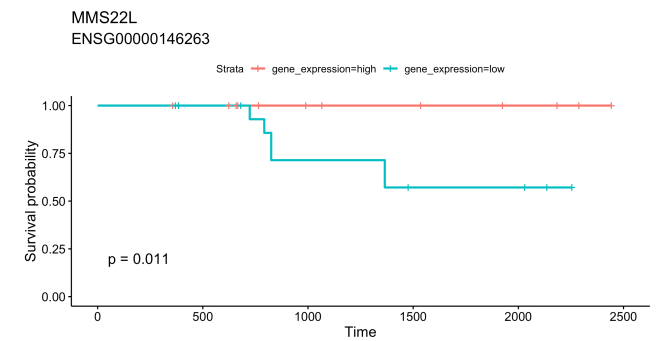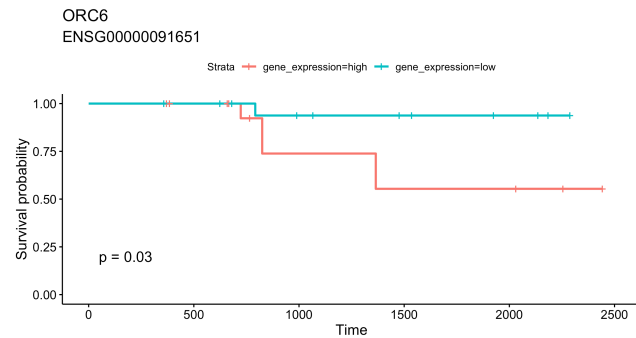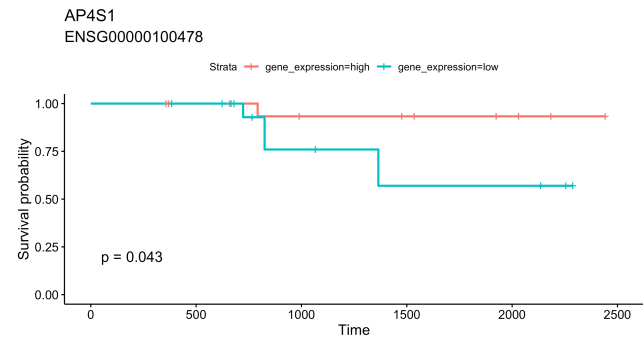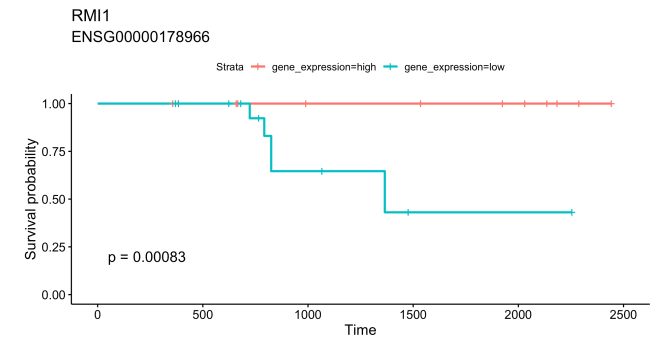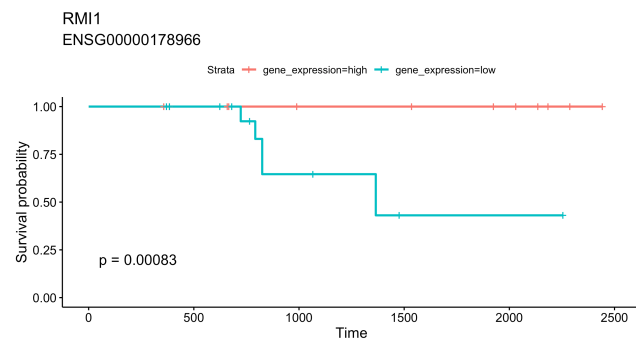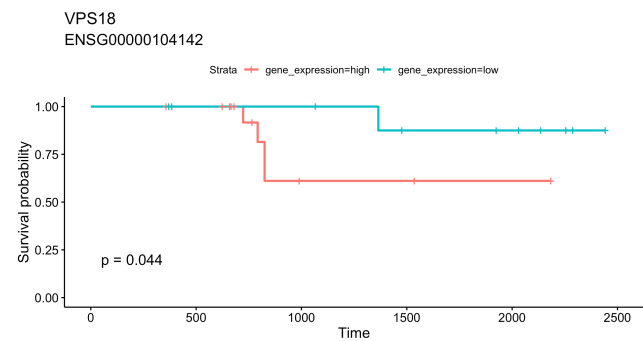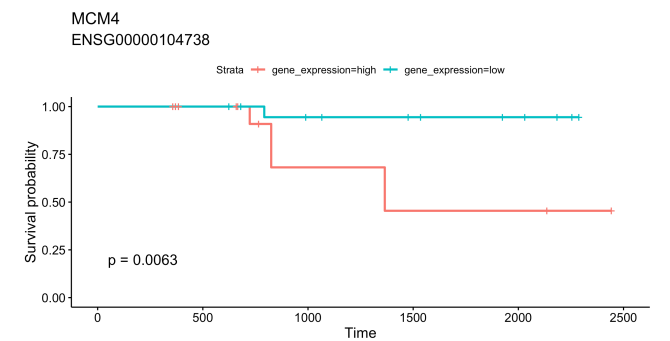

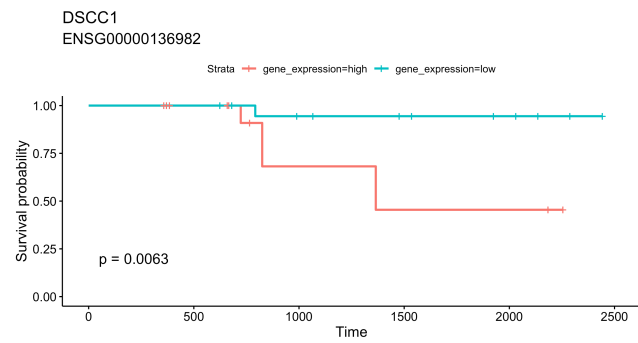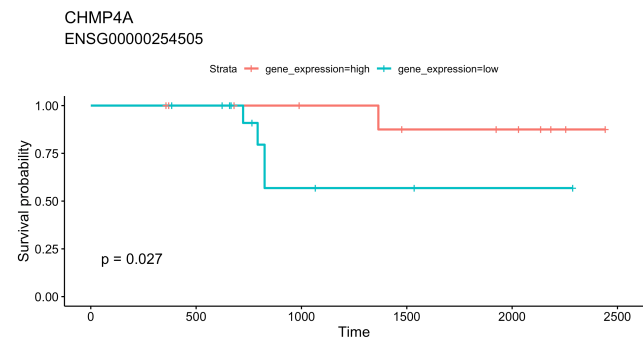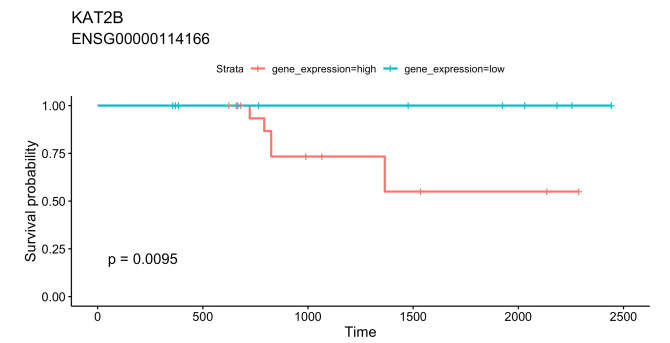

Supplement: Supplementary file 1 [file cancers-12-02183-s001.zip › cancers-855025-SUPPLE-XML/cancers-855025-supple-proof/Suppl_Fig4_breast_LumB_DOWNregulated_genes.pdf]

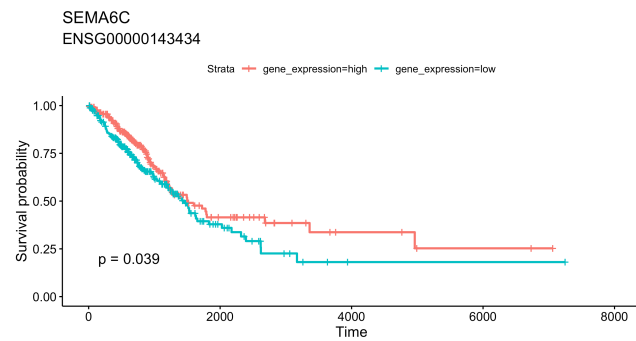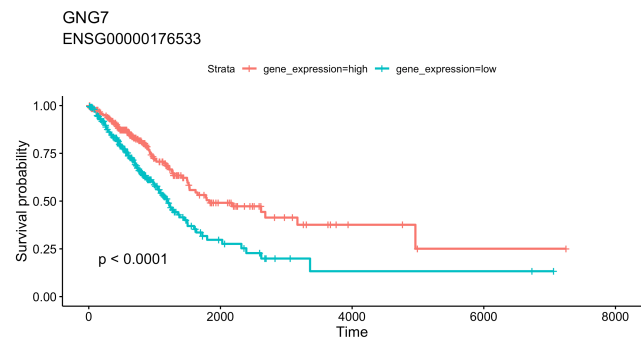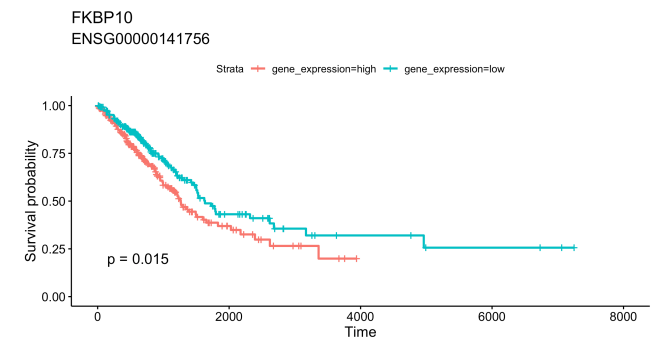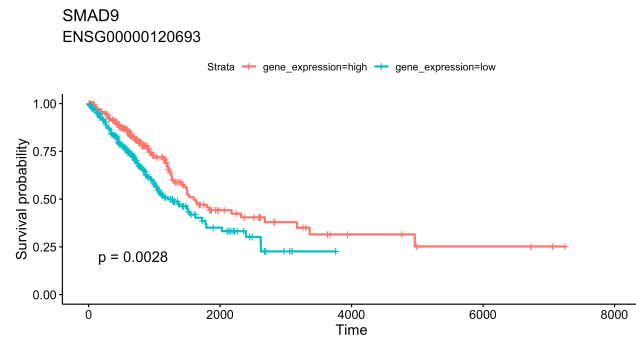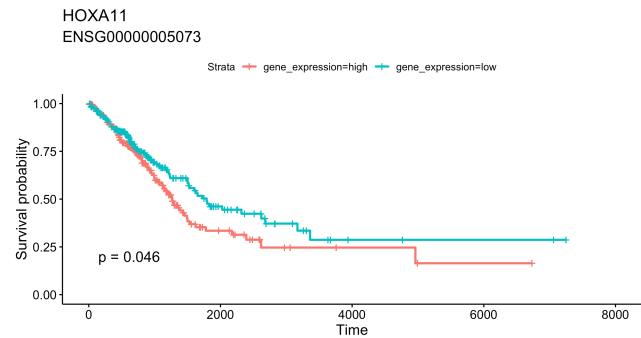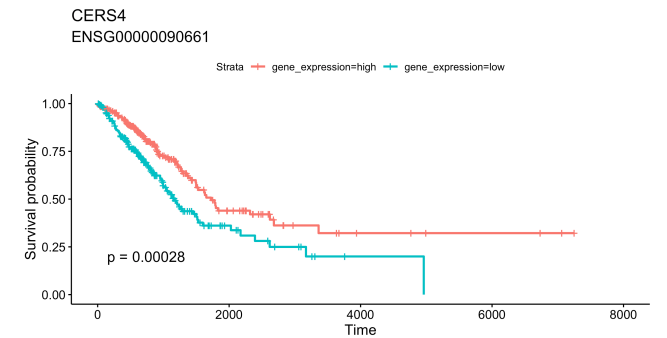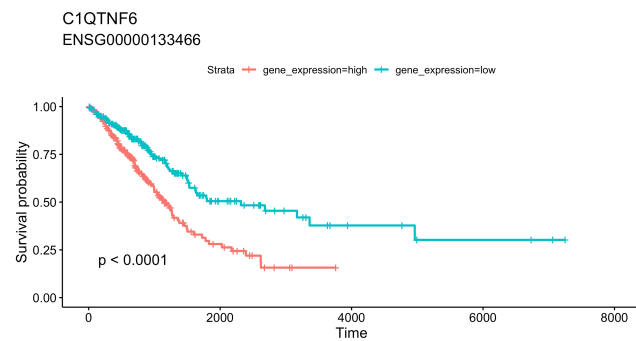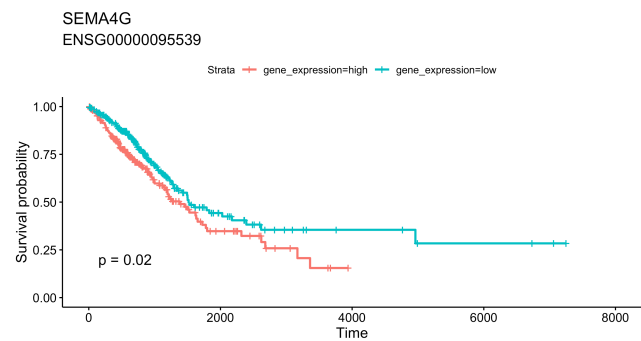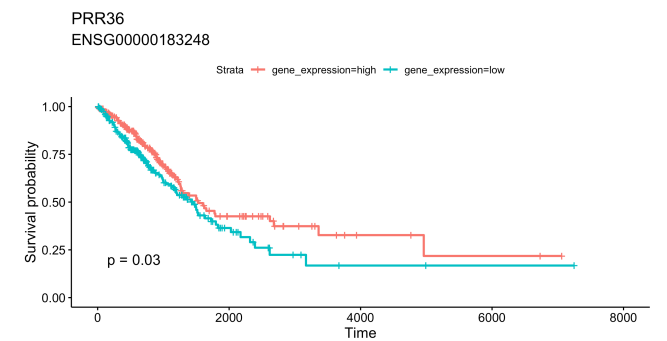

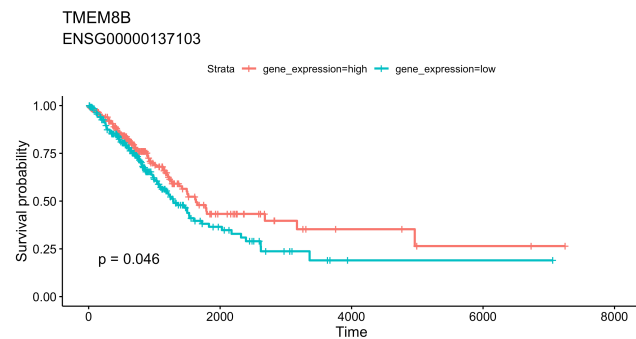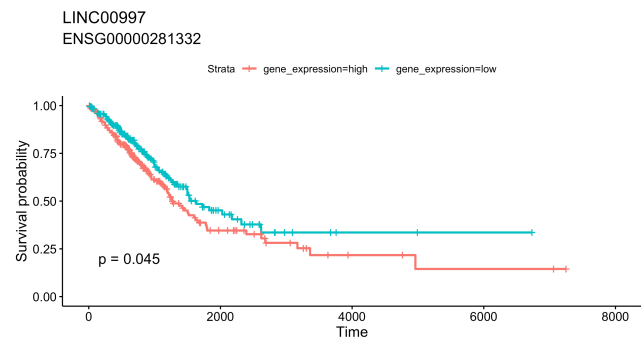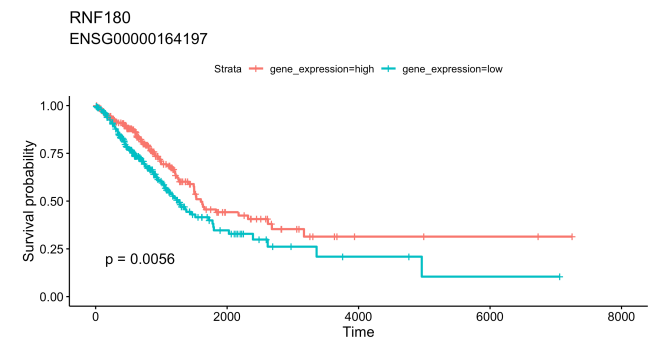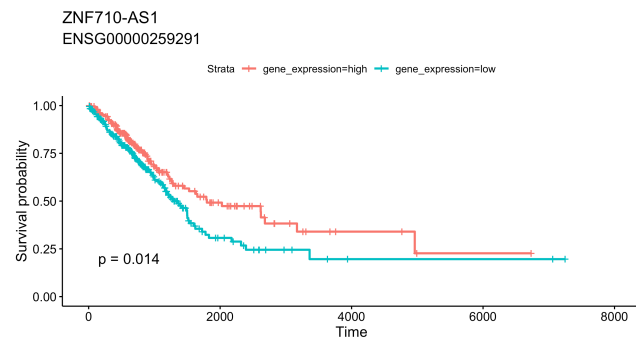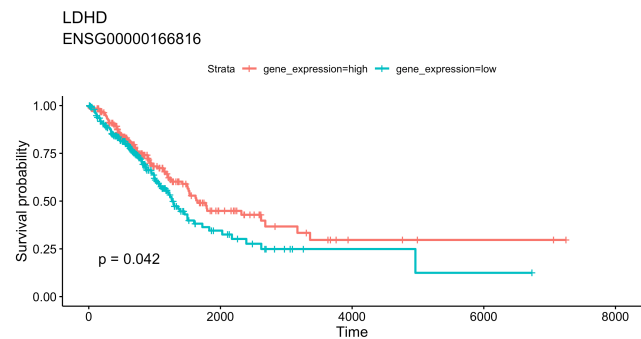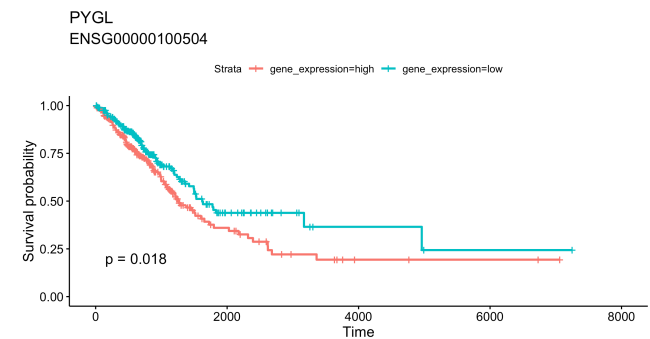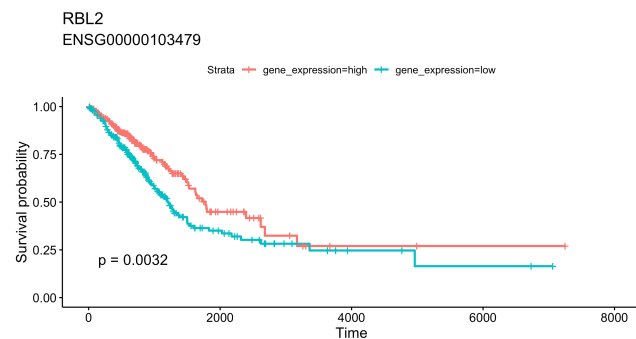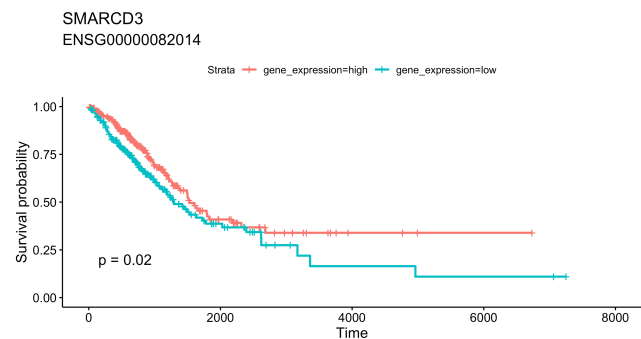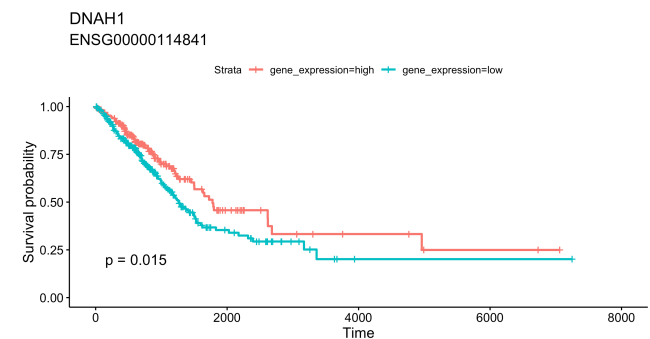

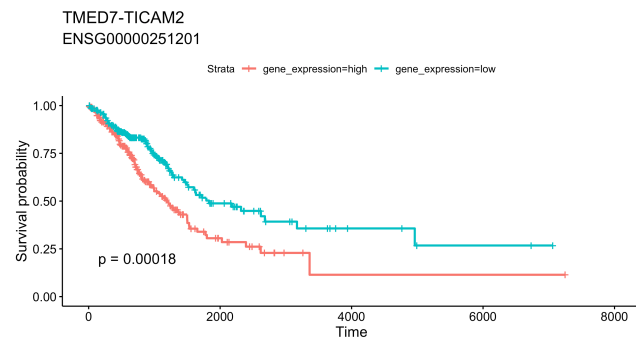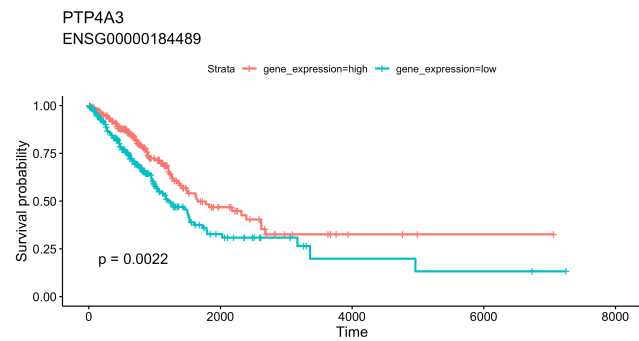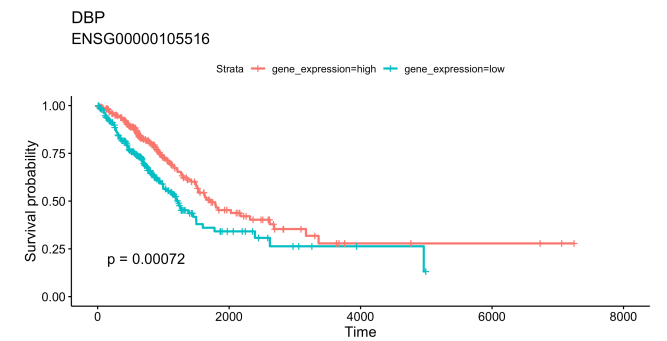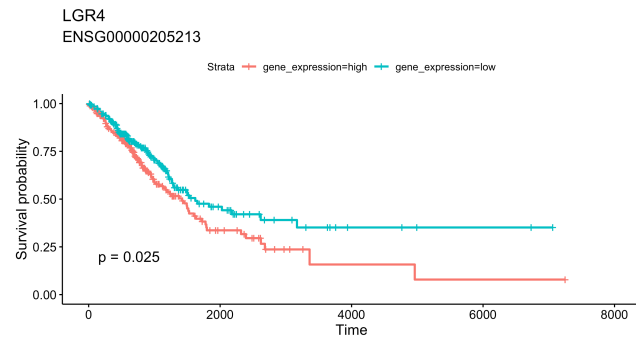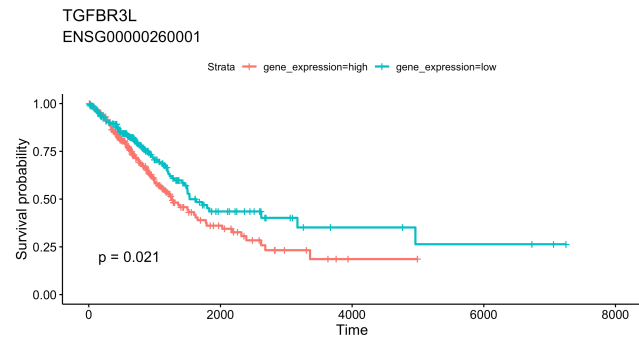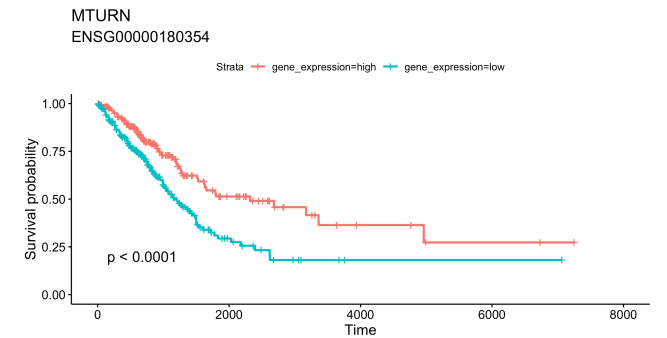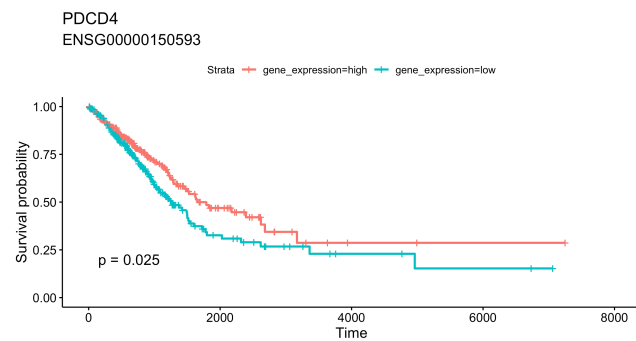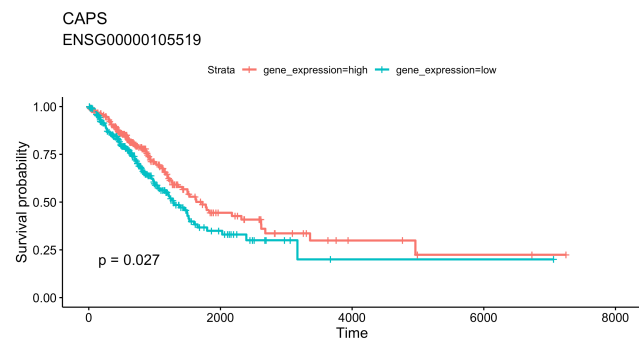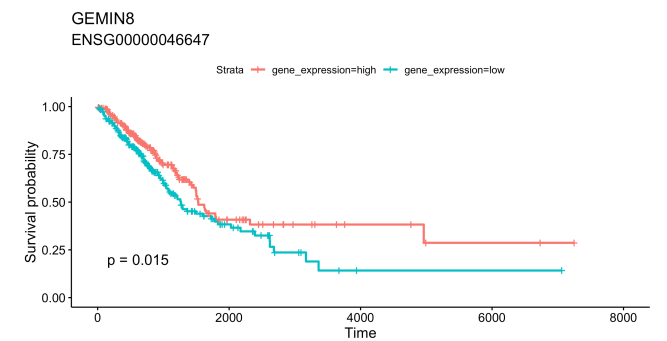

LGALS8  
ENSG00000116977

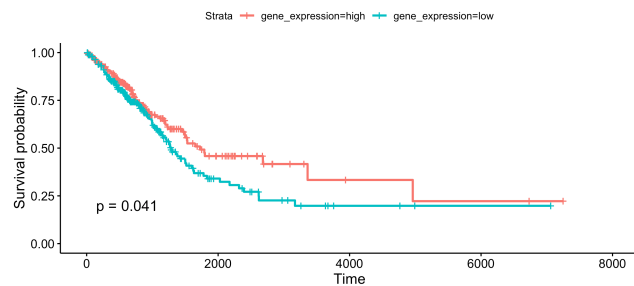

PPOX  
ENSG00000143224

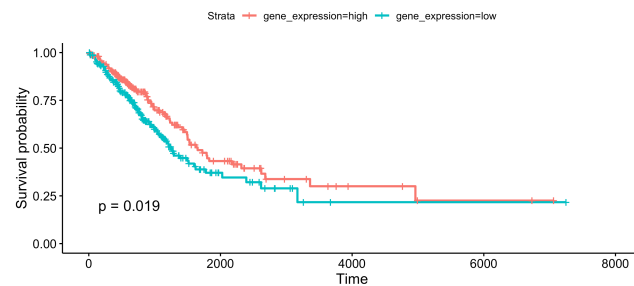

GPR153  
ENSG00000158292

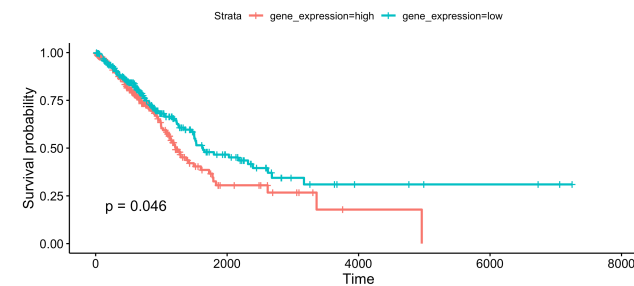

REEP6  
ENSG00000115255

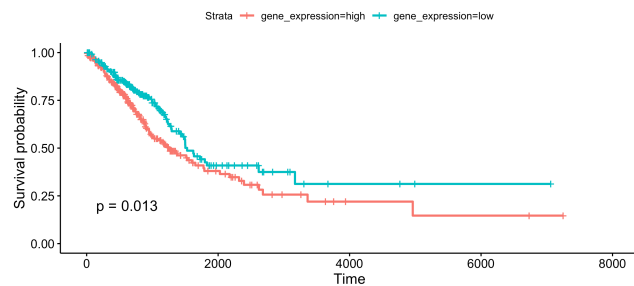

VAMP2  
ENSG00000220205

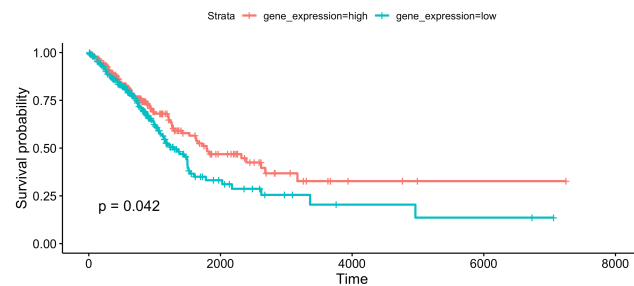

PNISR  
ENSG00000132424

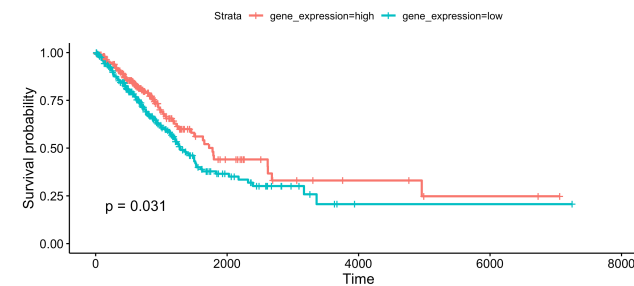

TDRD3  
ENSG00000083544

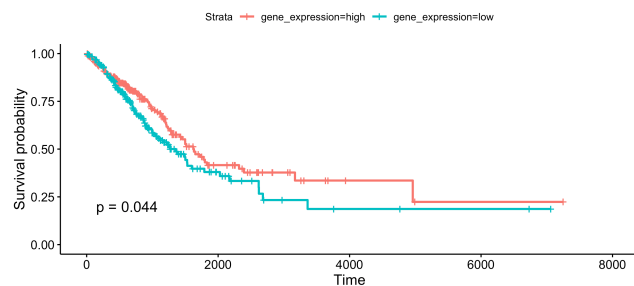

ZNF396  
ENSG00000186496

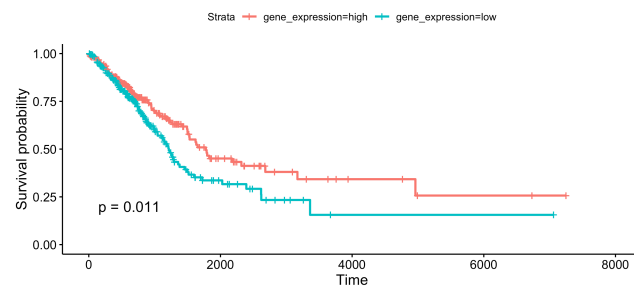

IFT140  
ENSG00000187535

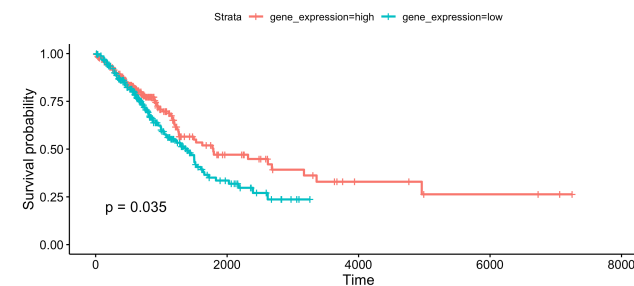

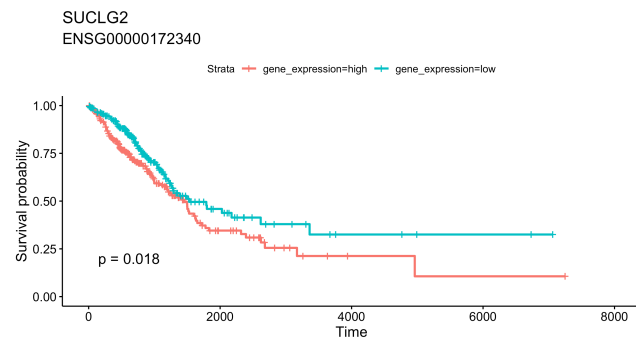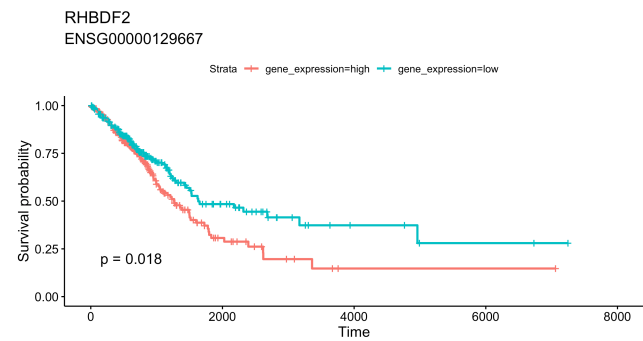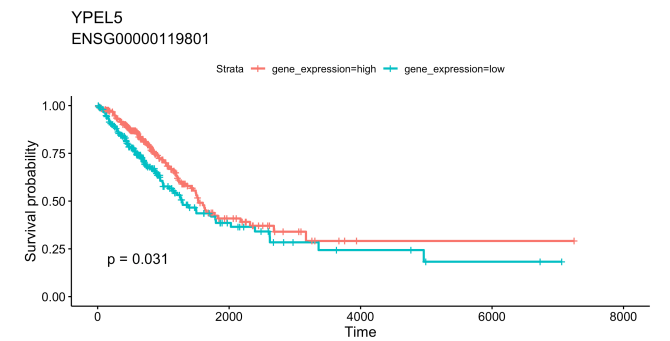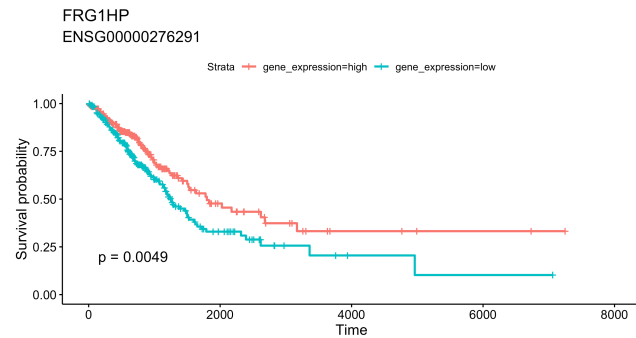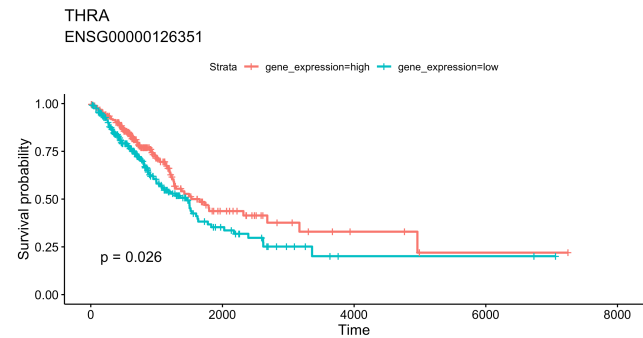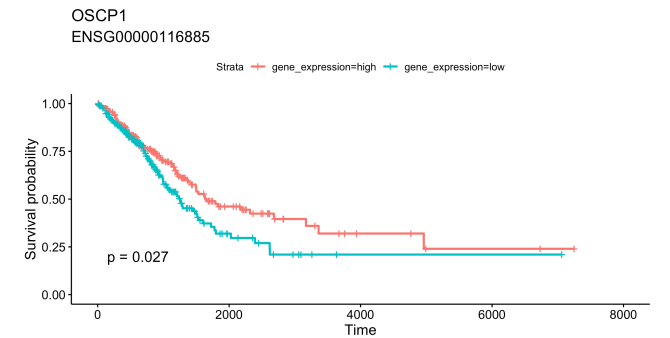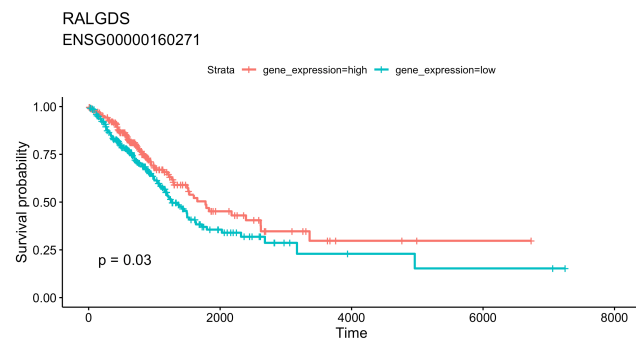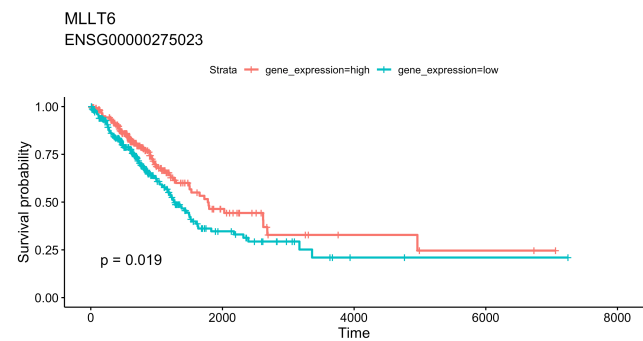

Supplement: Supplementary file 1 [file cancers-12-02183-s001.zip › cancers-855025-SUPPLE-XML/cancers-855025-supple-proof/Suppl_Fig5_lung_adenocarcinoma_UPregulated_genes.pdf]

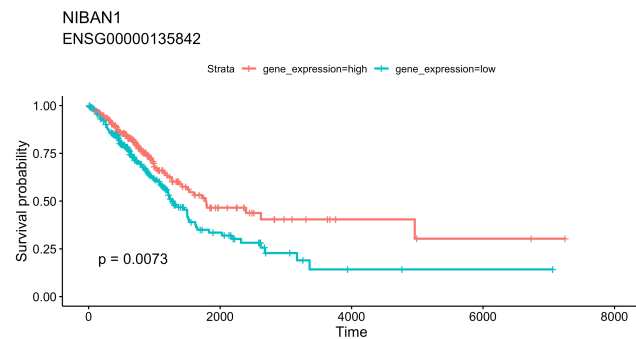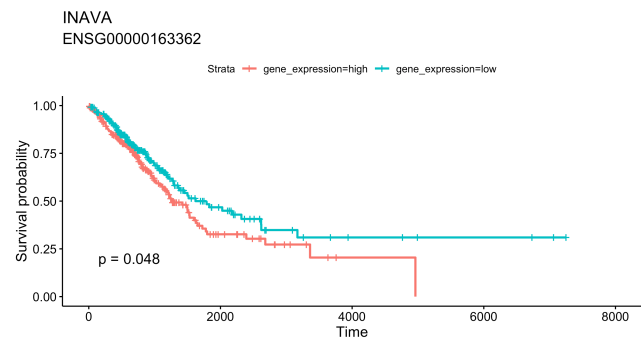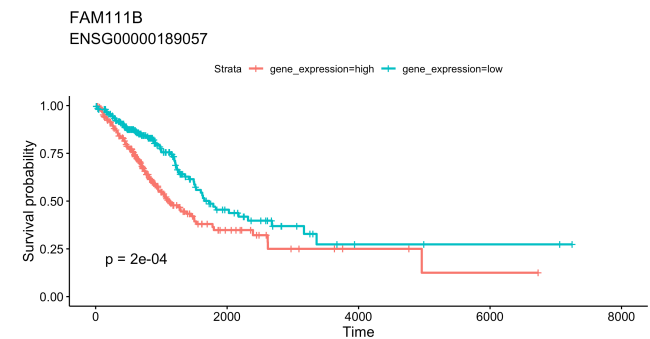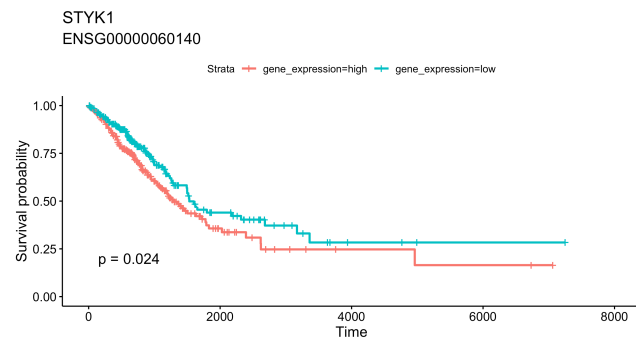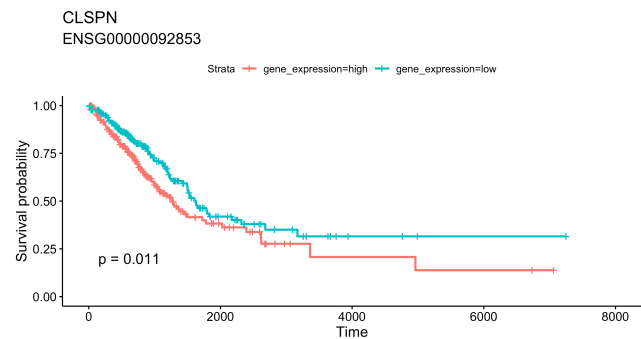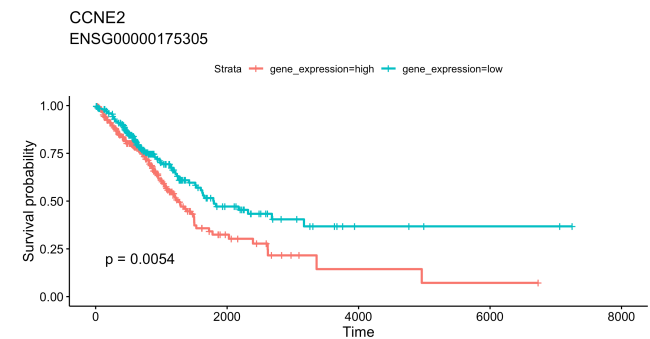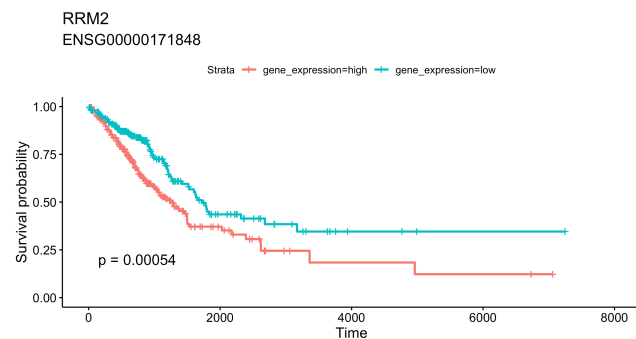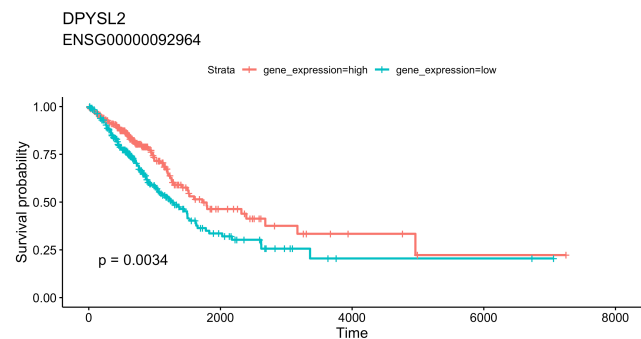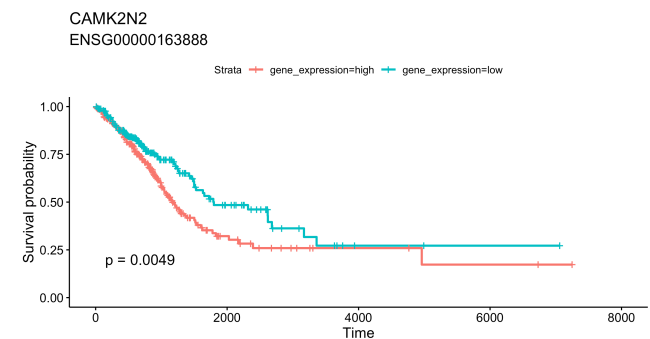

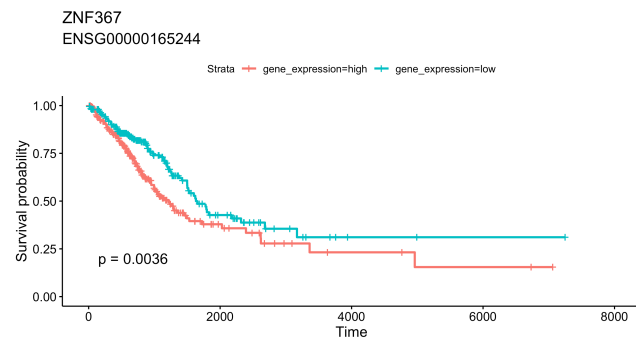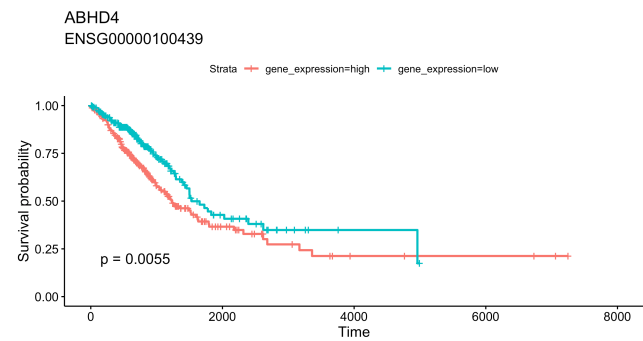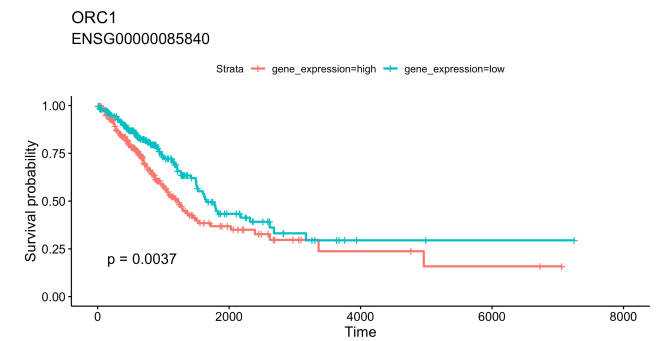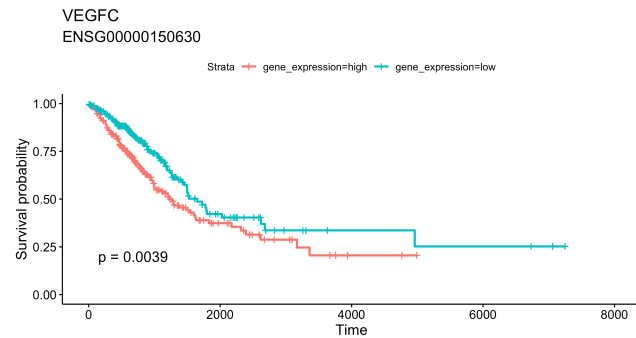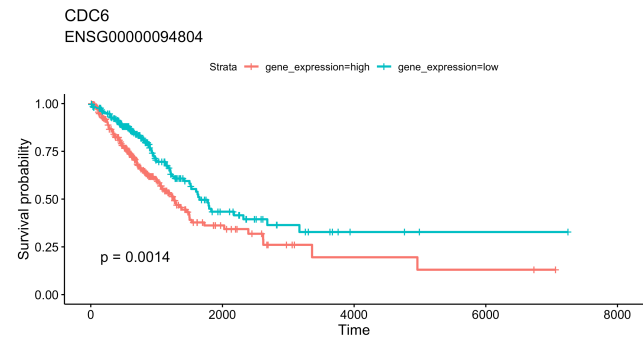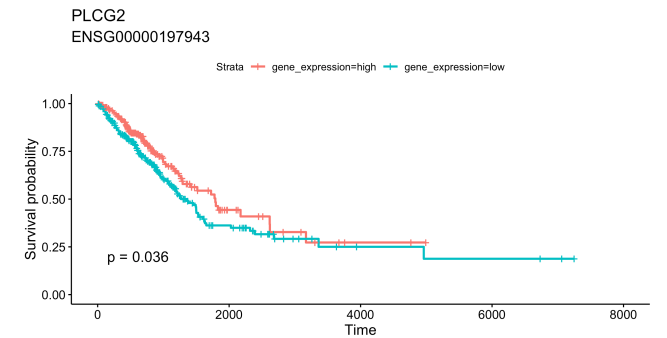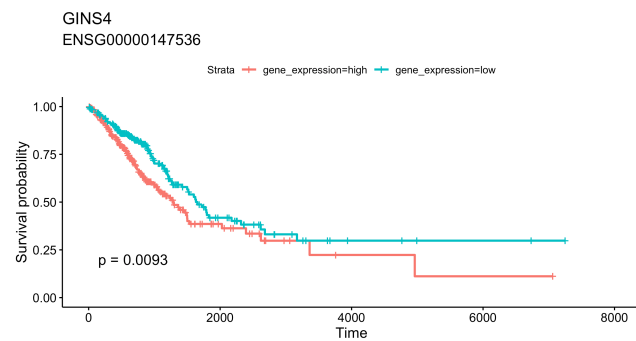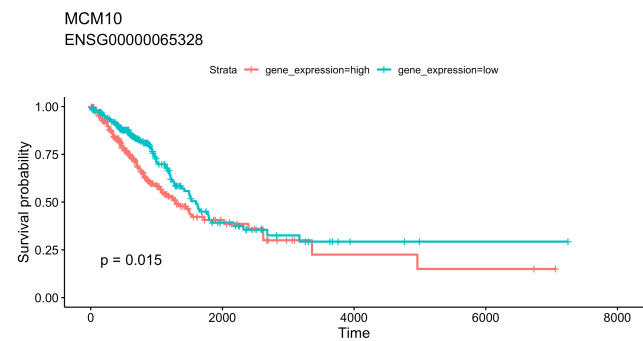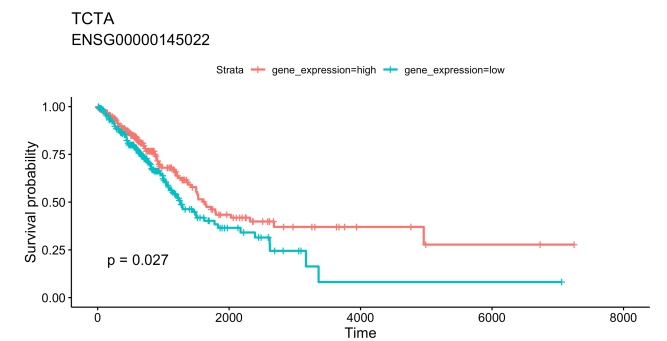

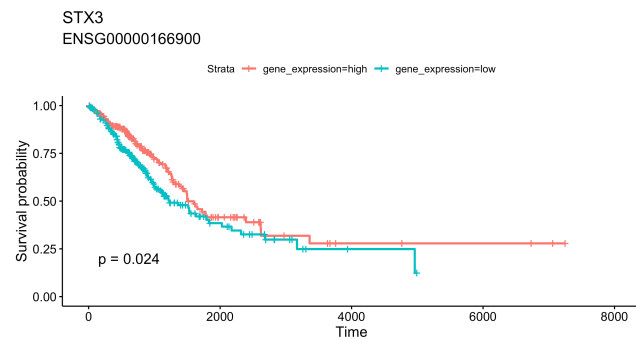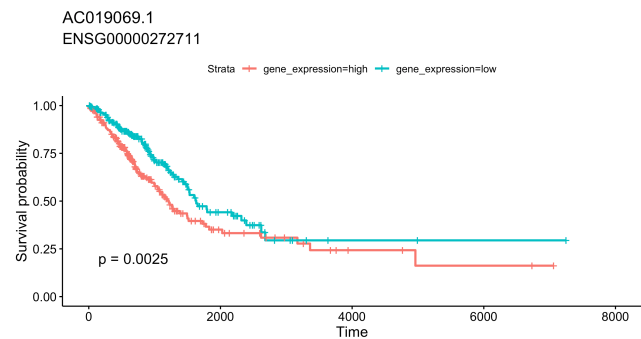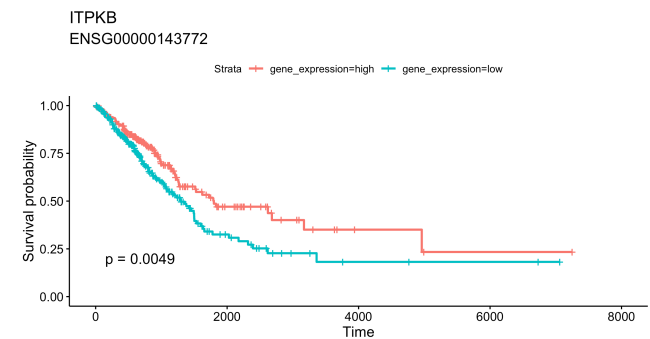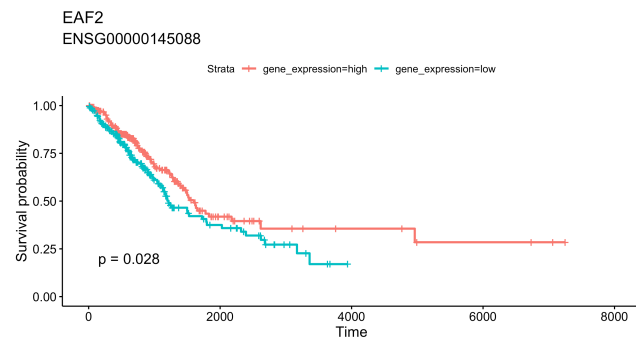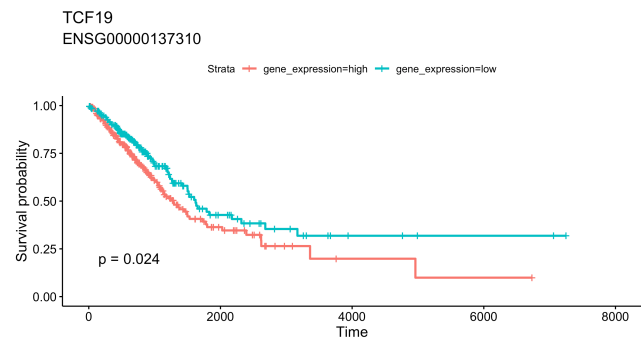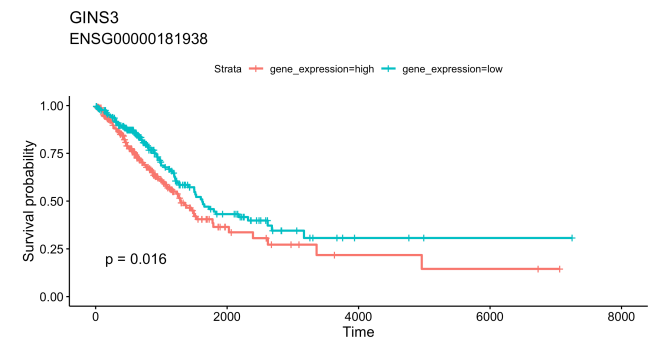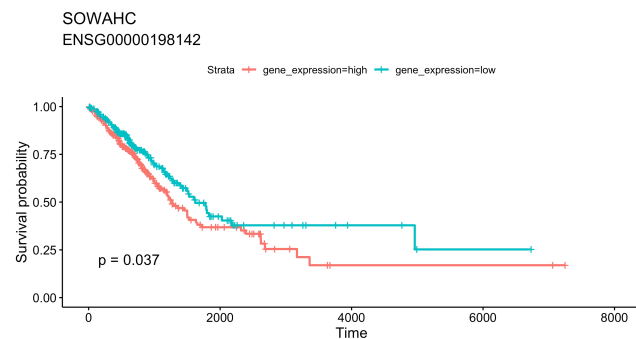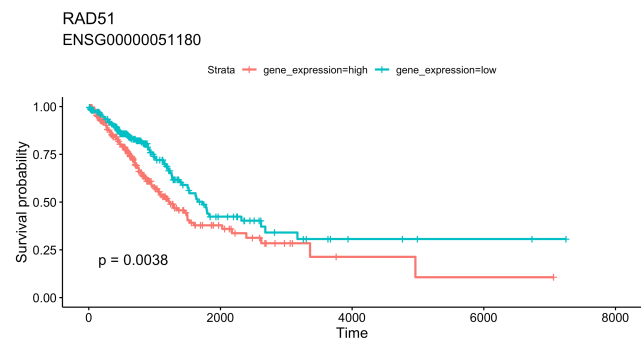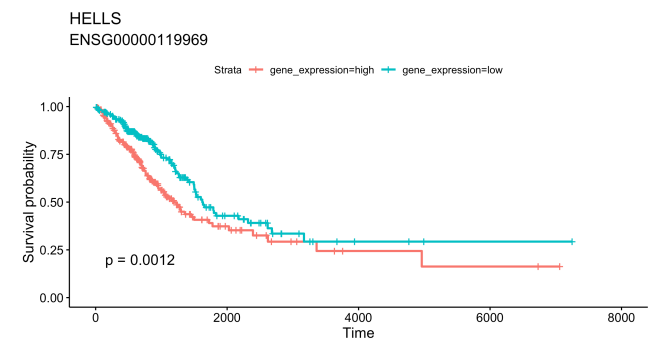

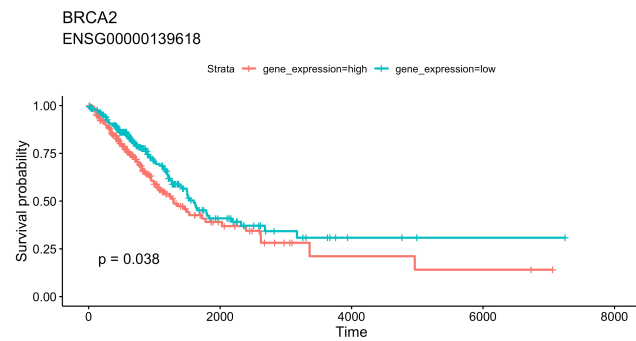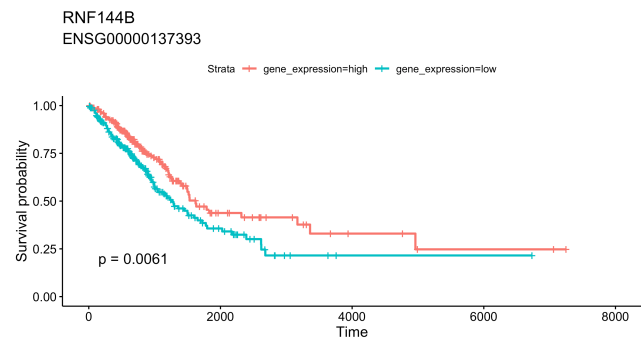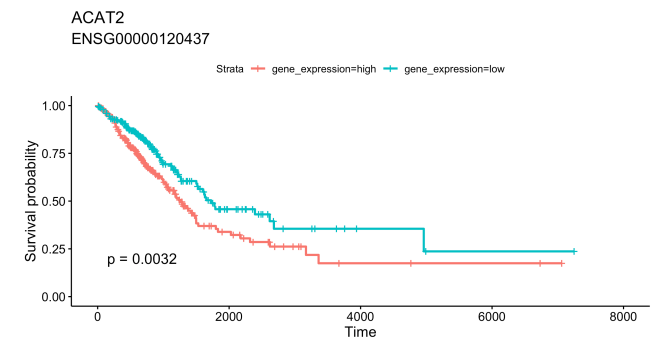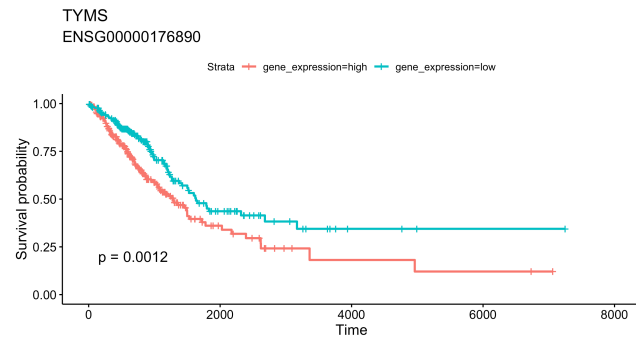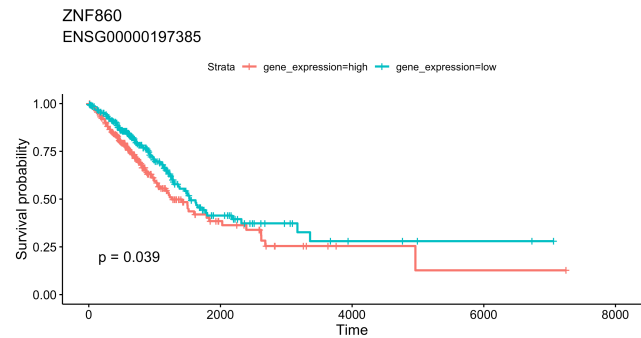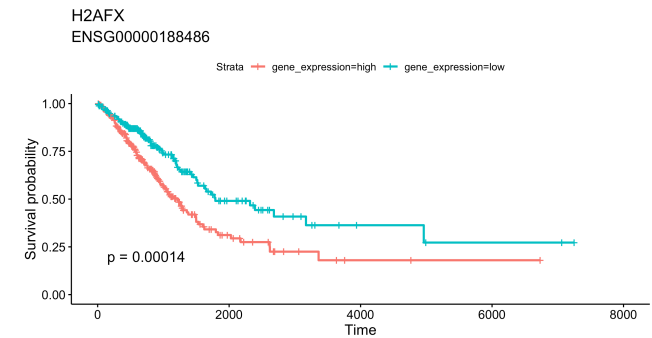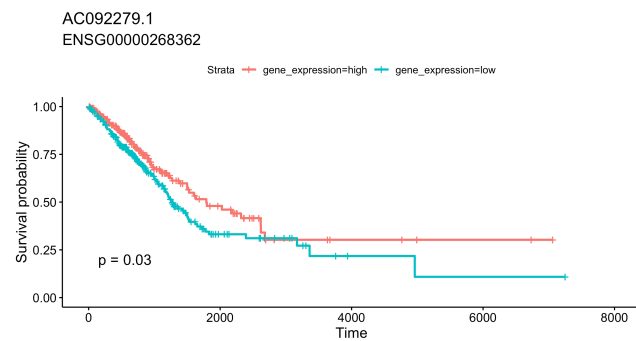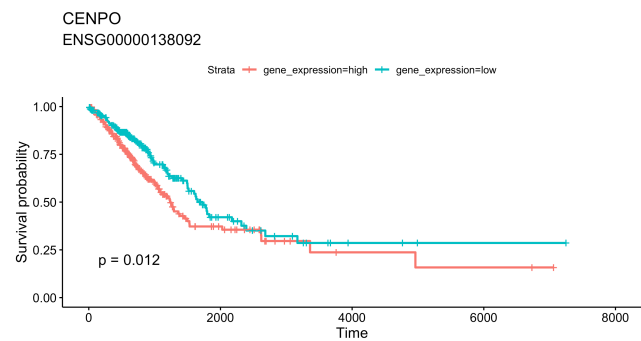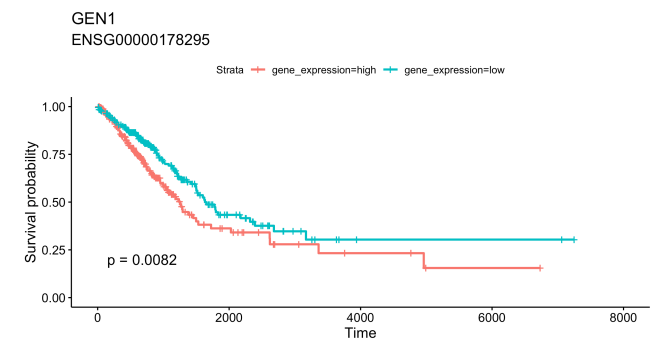

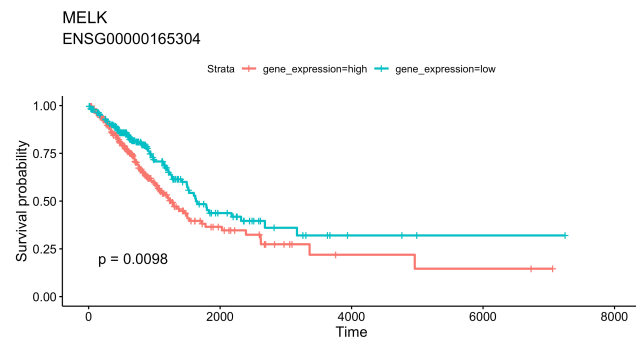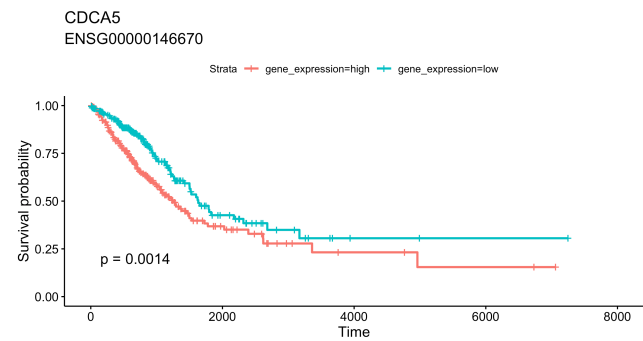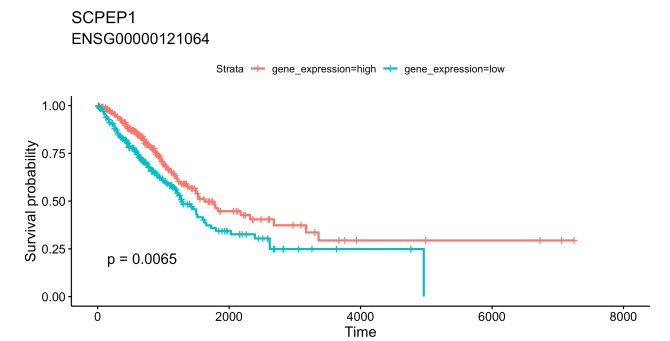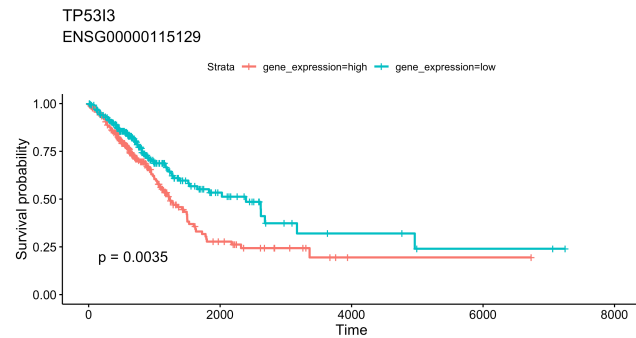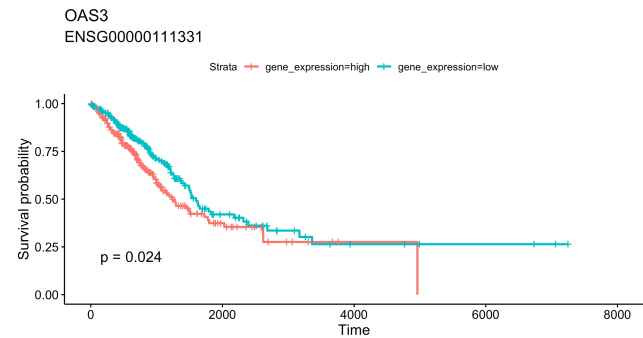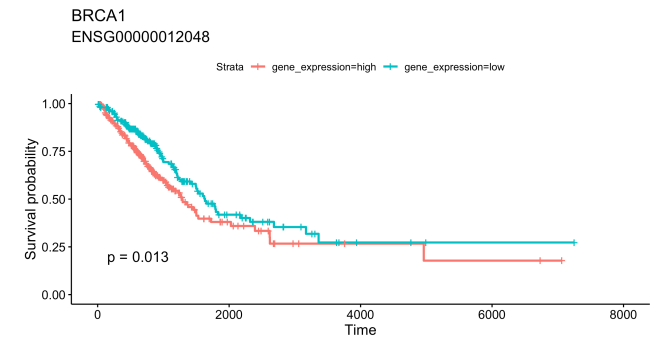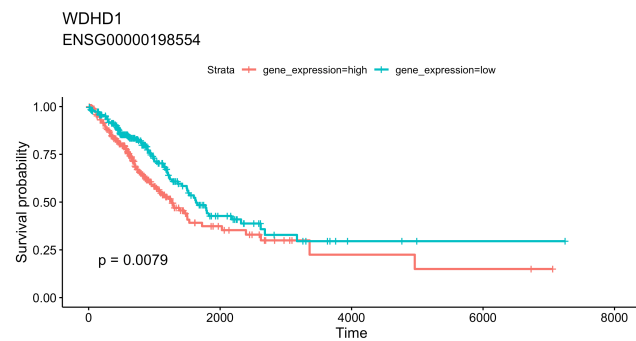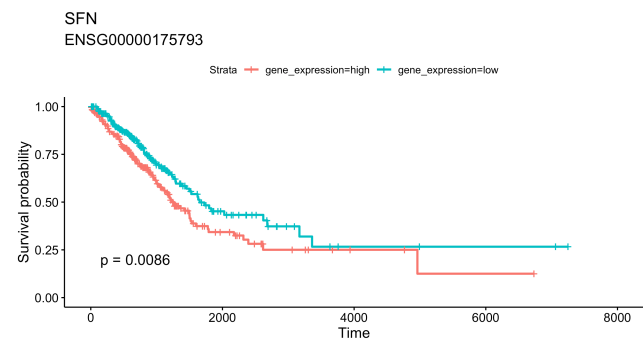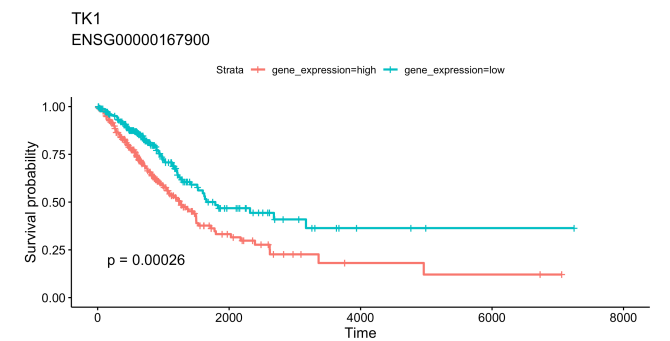

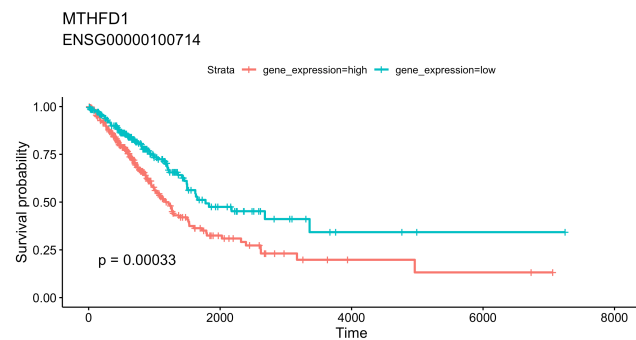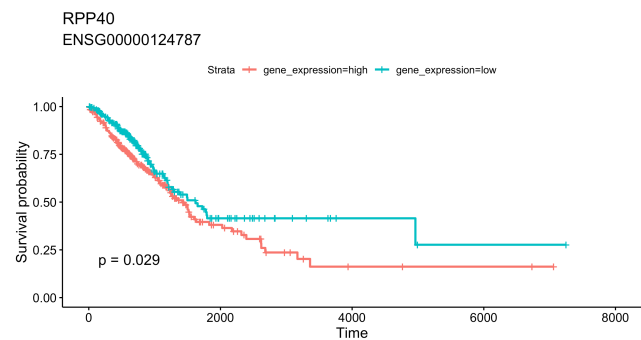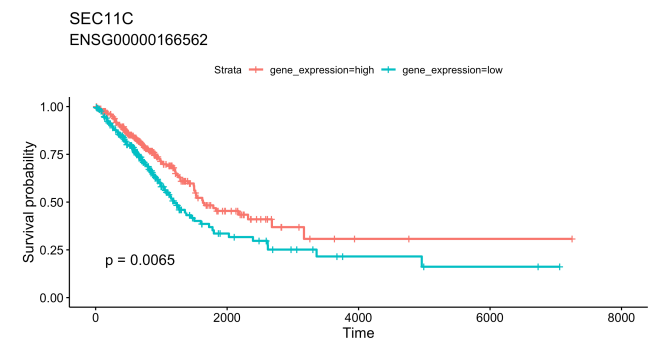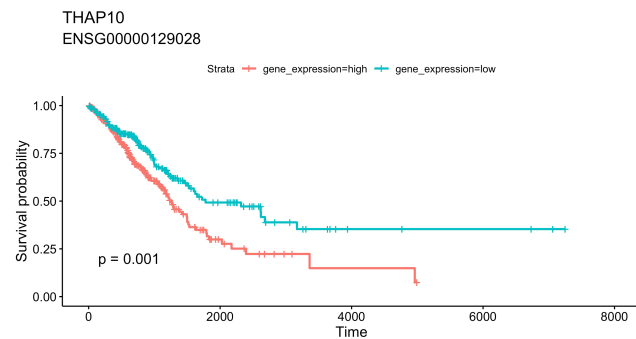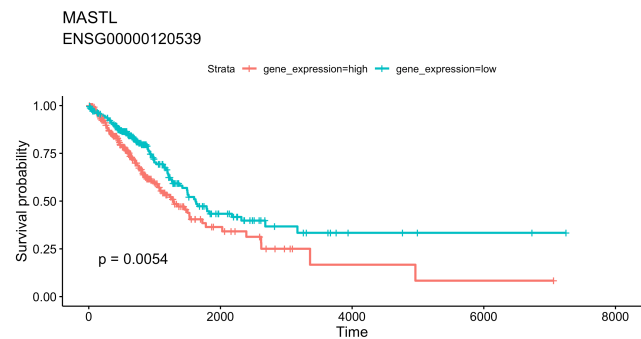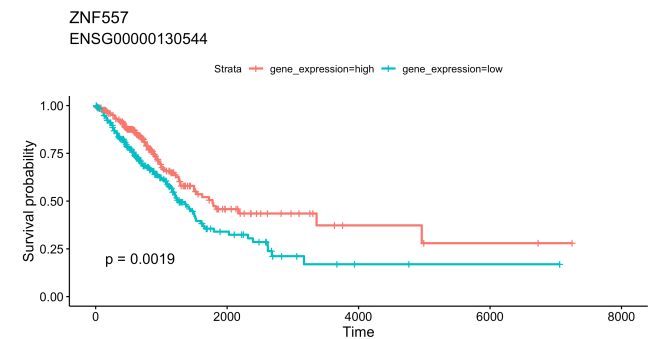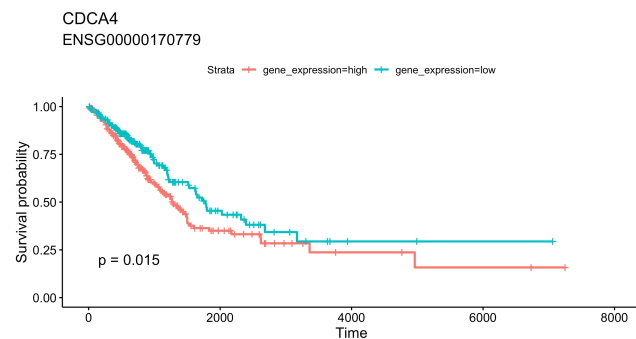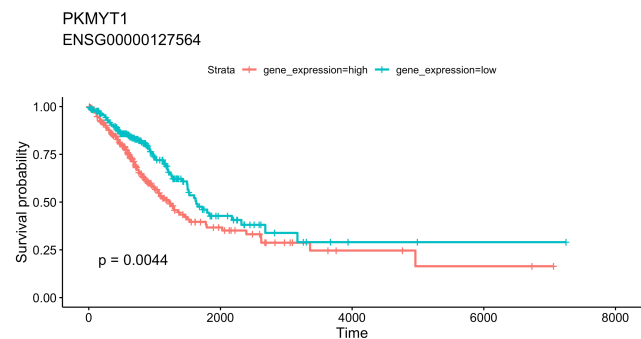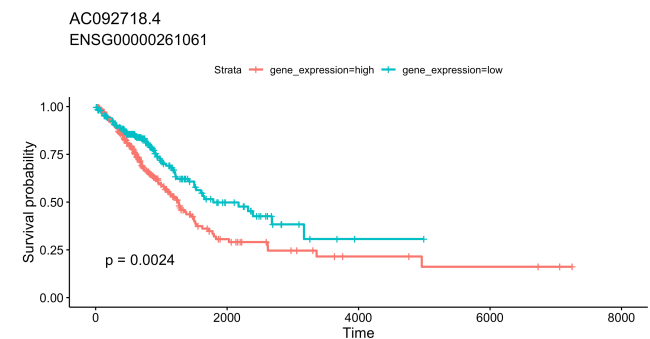

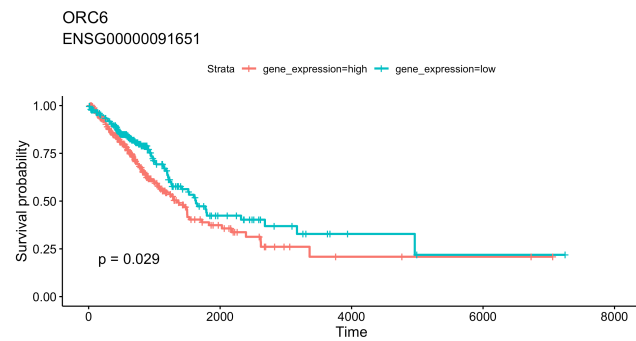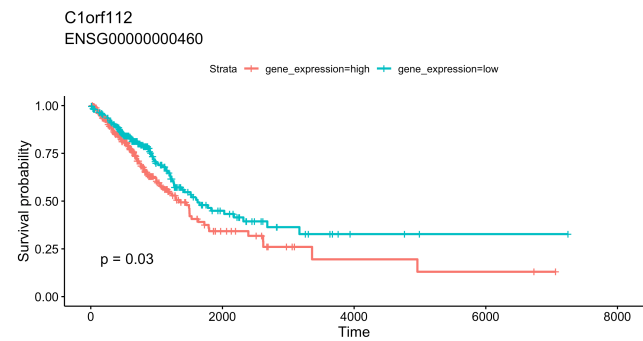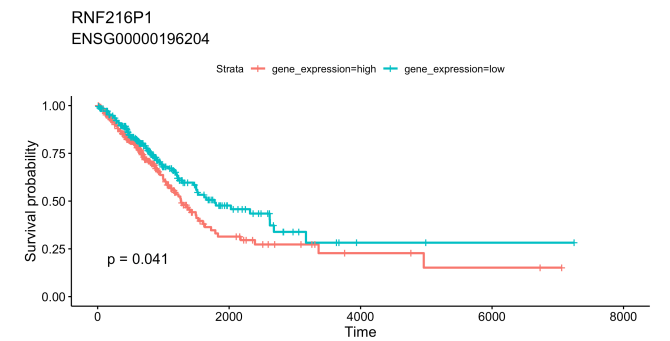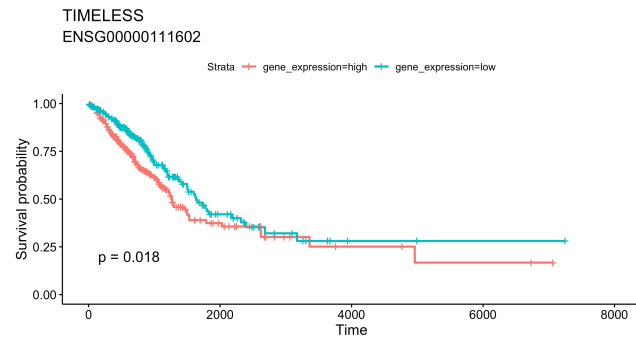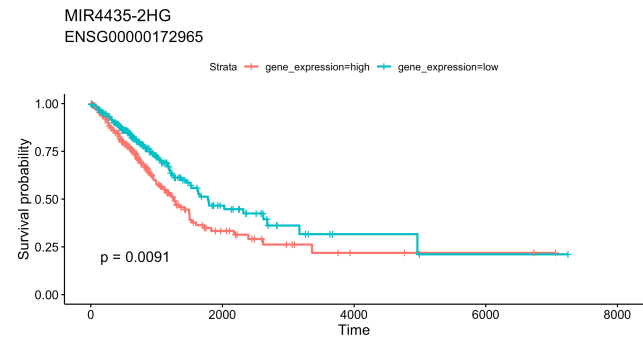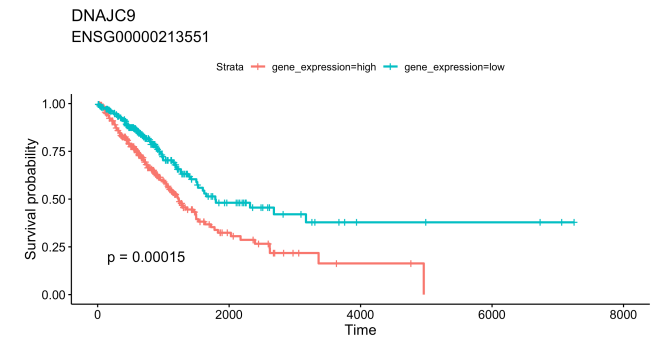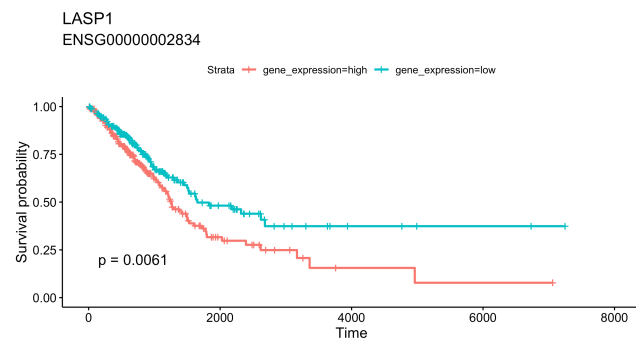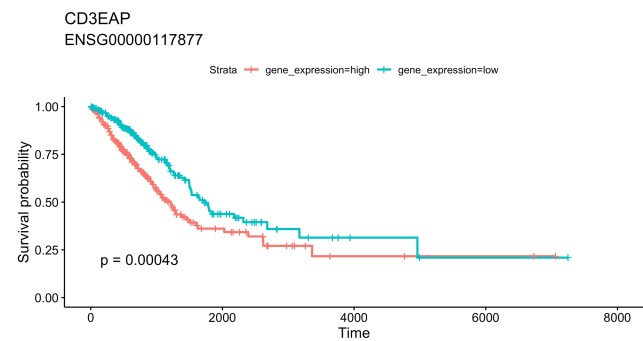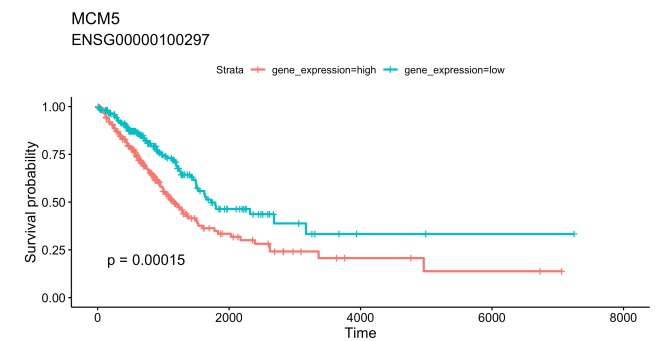

RFC2  
ENSG00000049541

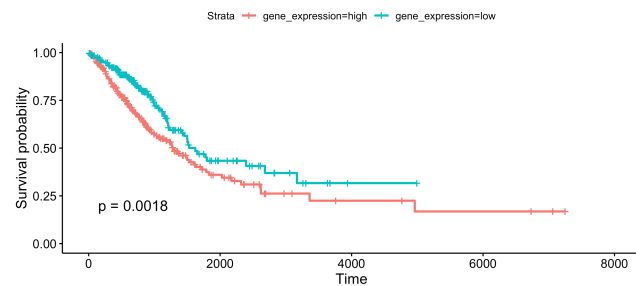

DSCC1  
ENSG00000136982

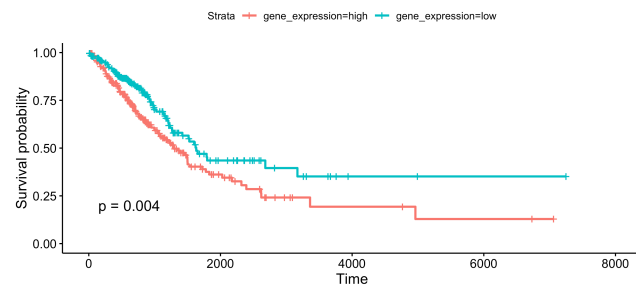

GTF2A1  
ENSG00000165417

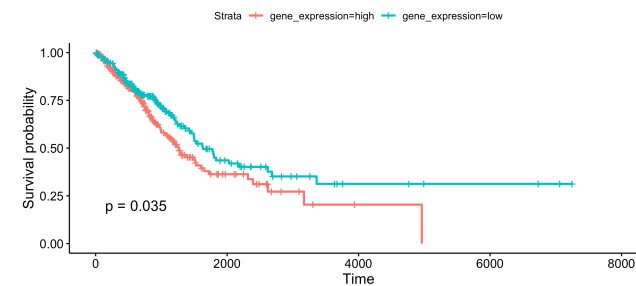

SLC25A33  
ENSG00000171612

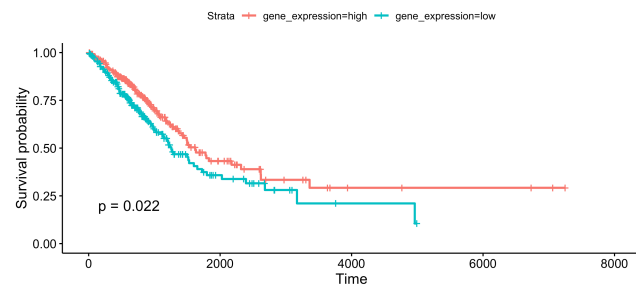

CHMP4A  
ENSG00000254505

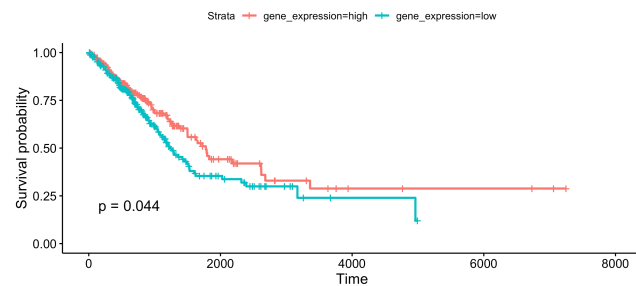

NEU1  
ENSG00000204386

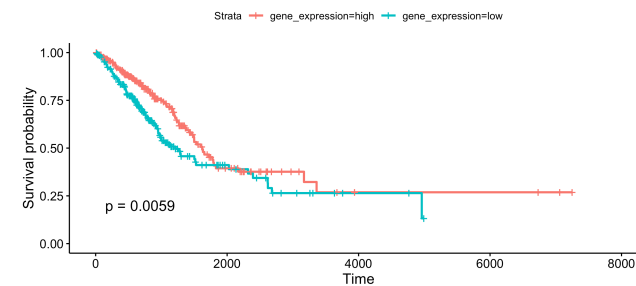

EIF6  
ENSG00000242372

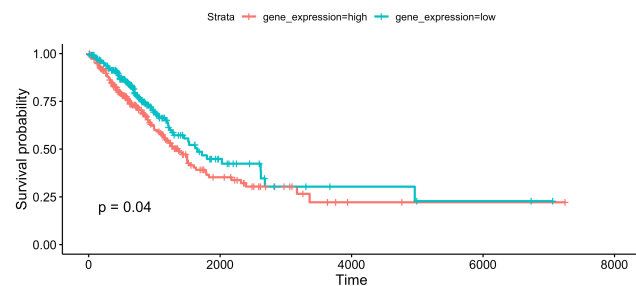

Supplement: Supplementary file 1 [file cancers-12-02183-s001.zip › cancers-855025-SUPPLE-XML/cancers-855025-supple-proof/Suppl_Fig6_lung_adenocarcinoma_DOWNregulated_genes.pdf]

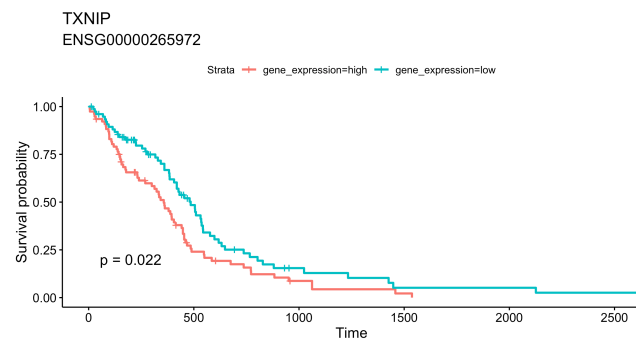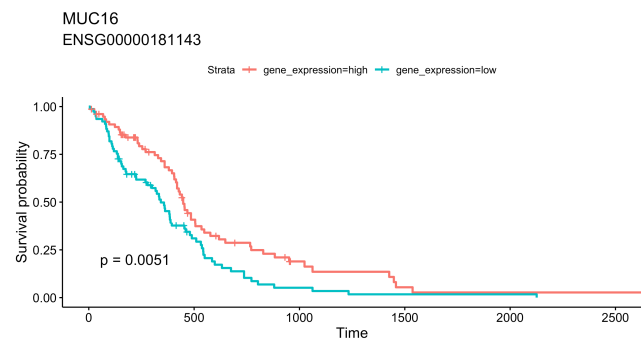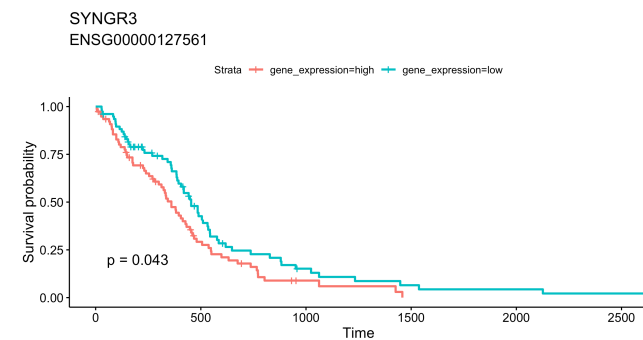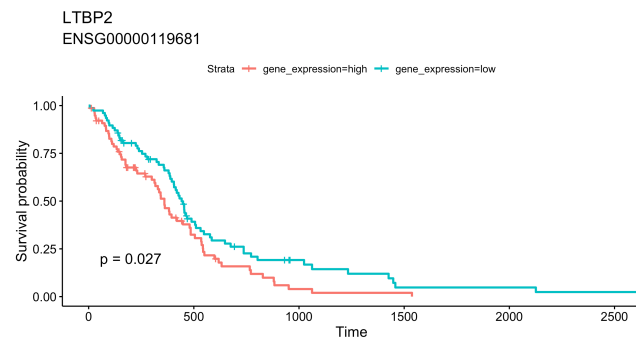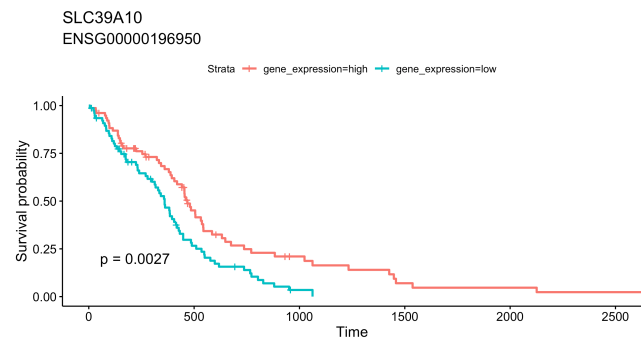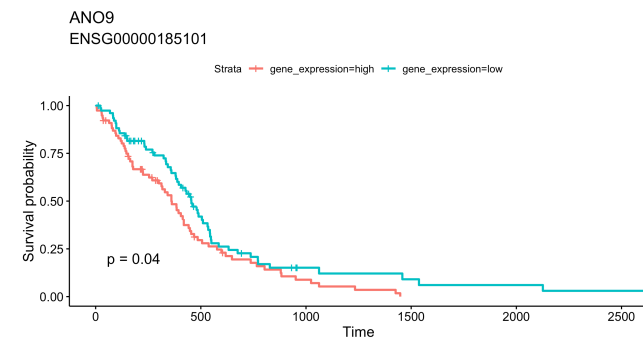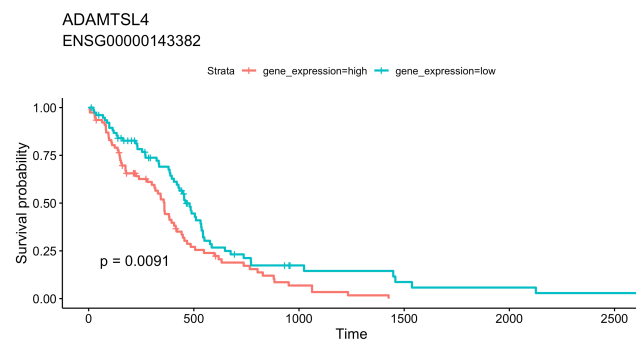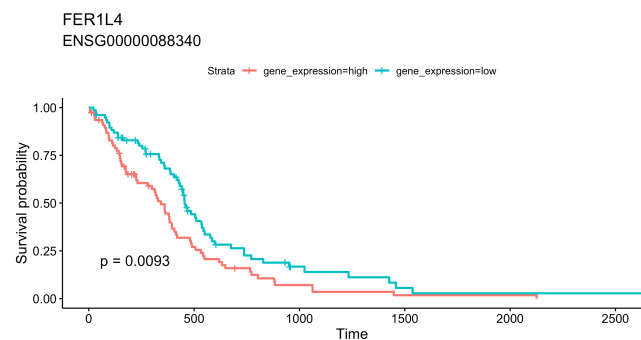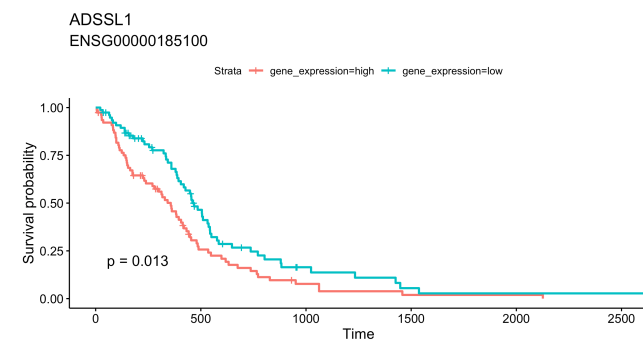

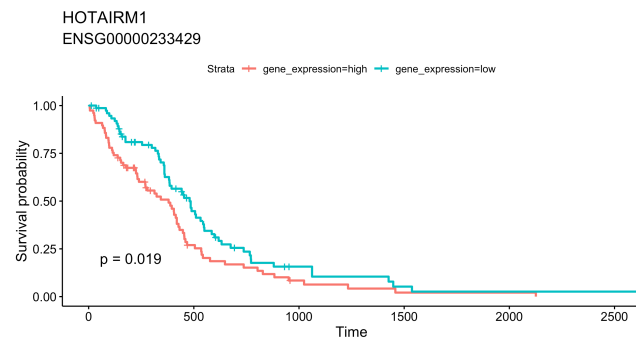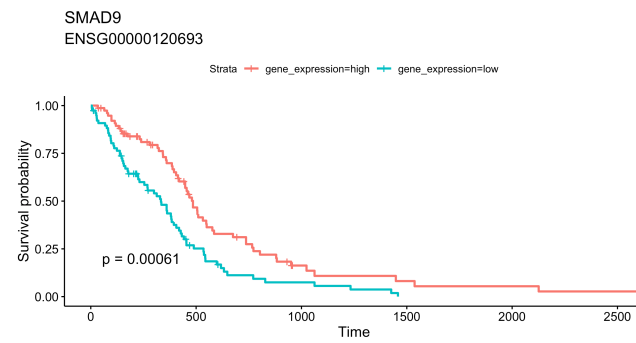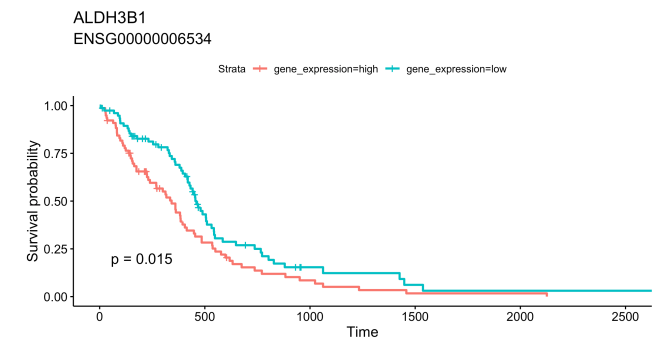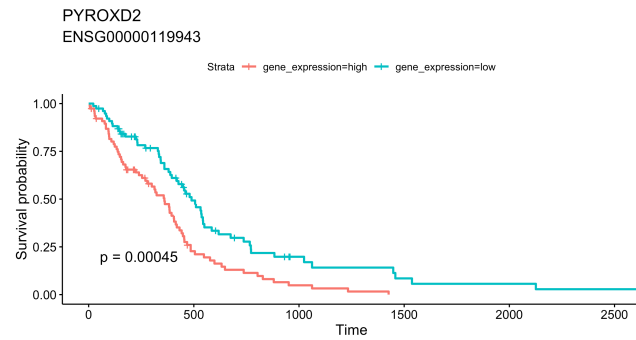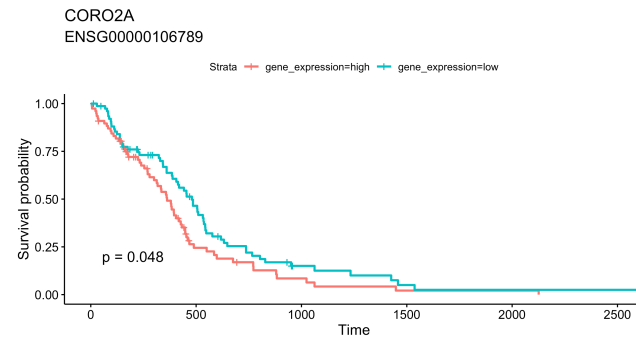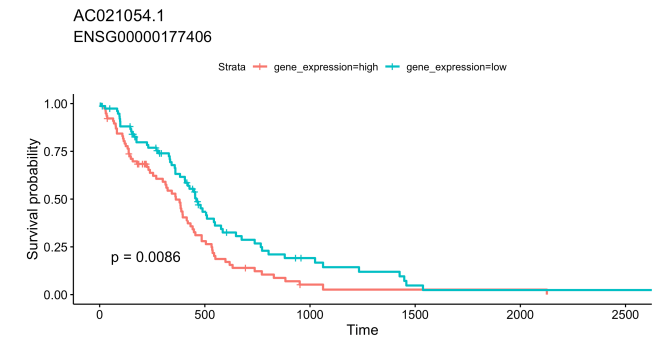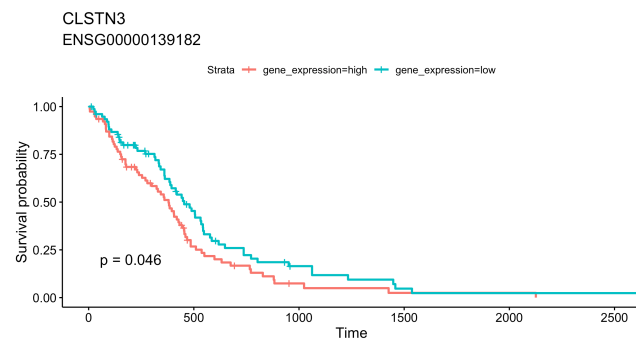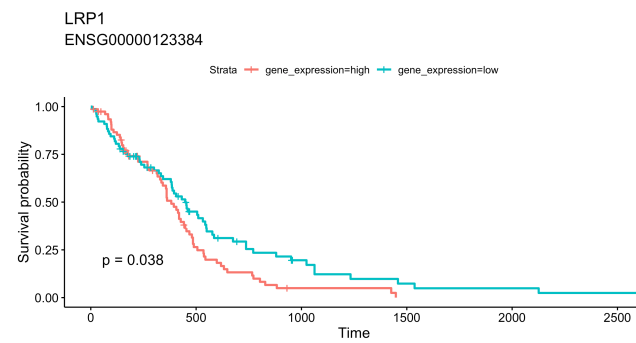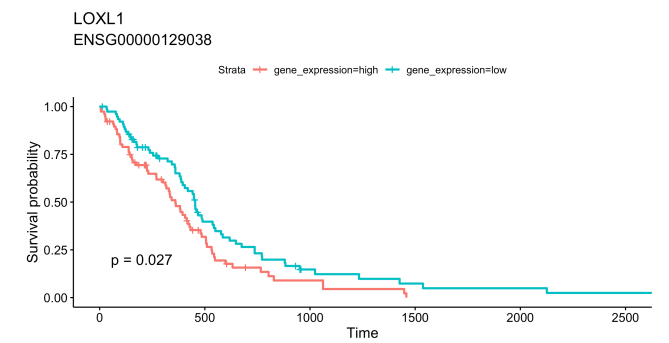

OPN3  
ENSG00000054277

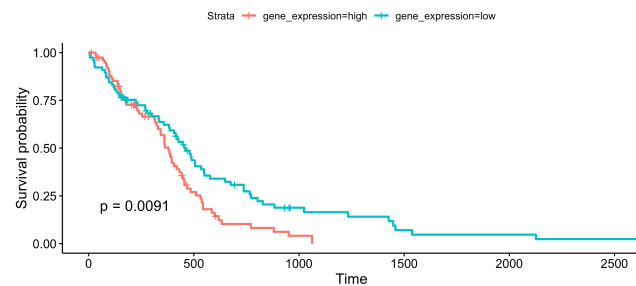

ZDHHC1  
ENSG00000159714

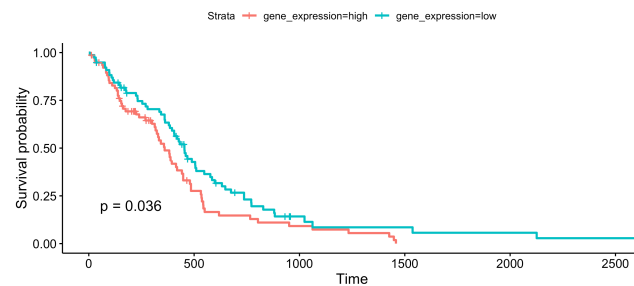

RHOBTB1  
ENSG00000072422

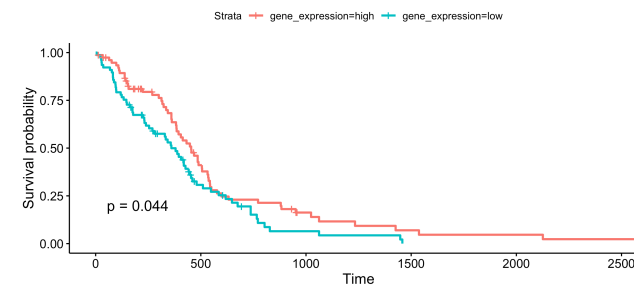

ACADS  
ENSG00000122971

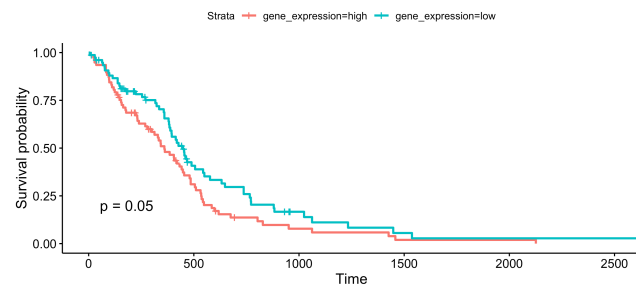

PPARA  
ENSG00000186951

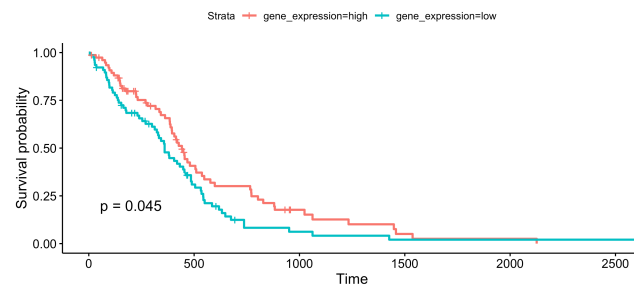

COL27A1  
ENSG00000196739

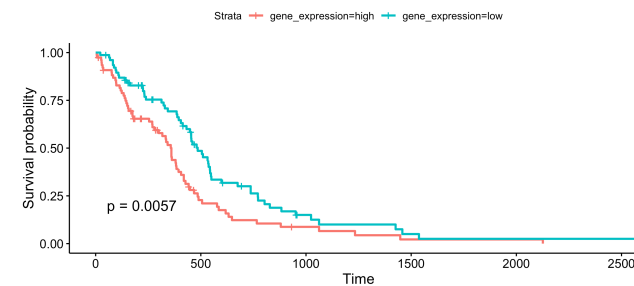

TTC28  
ENSG00000100154

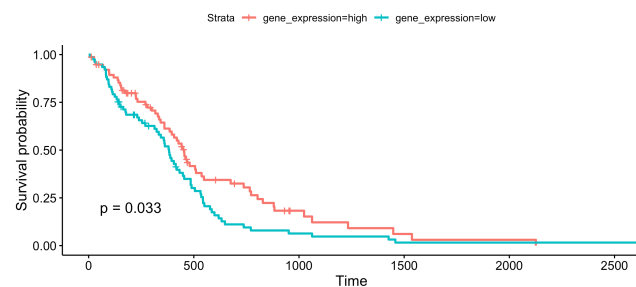

KIZ  
ENSG00000088970

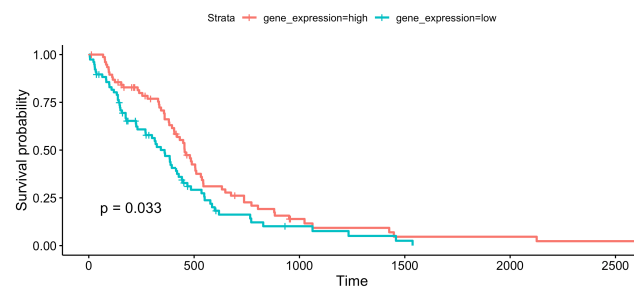

C11orf54  
ENSG00000182919

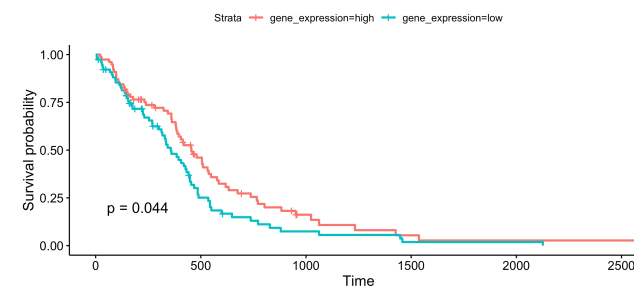

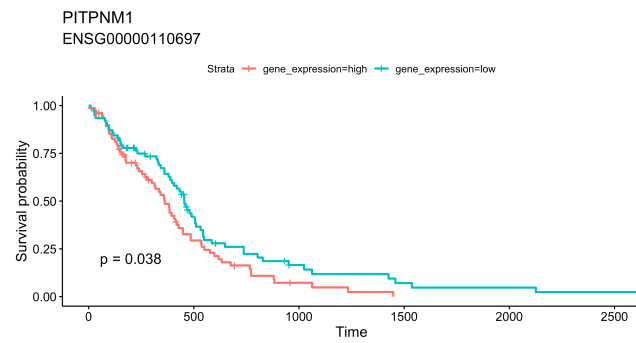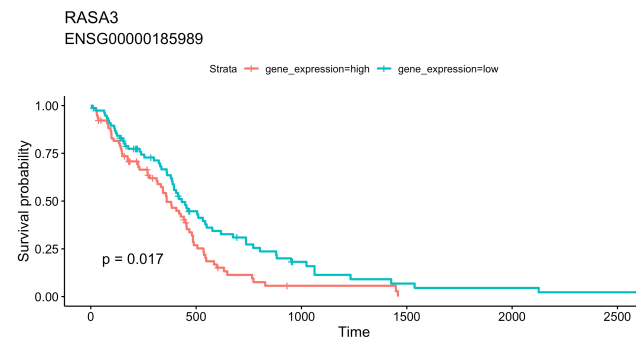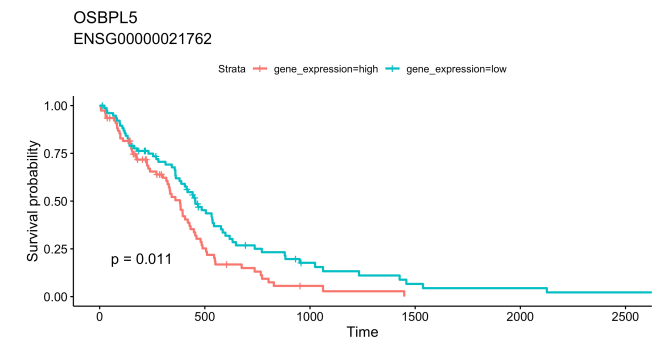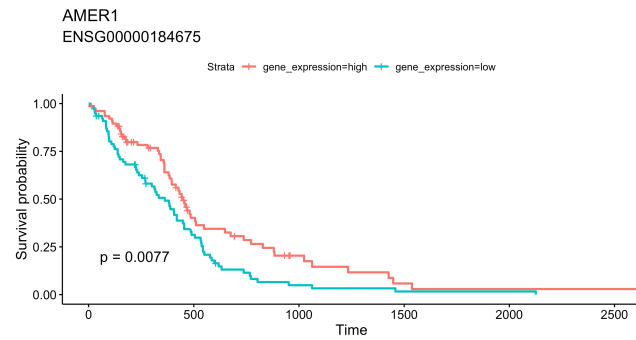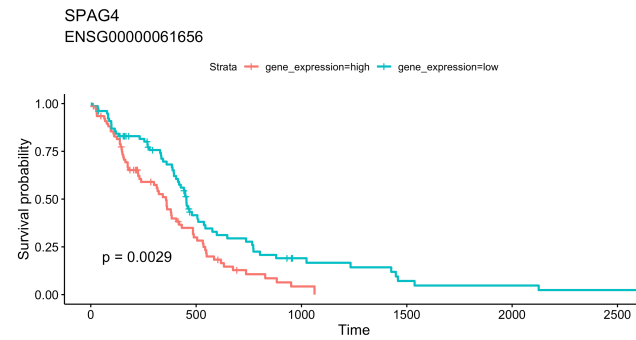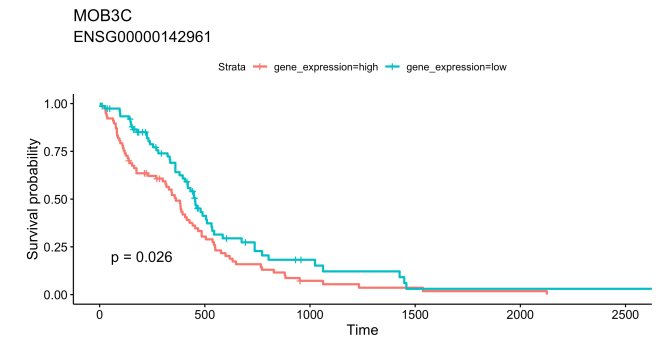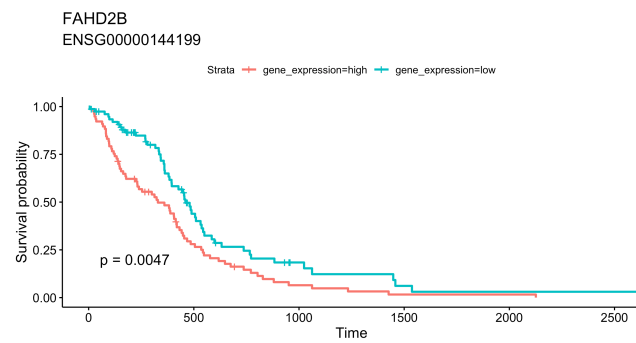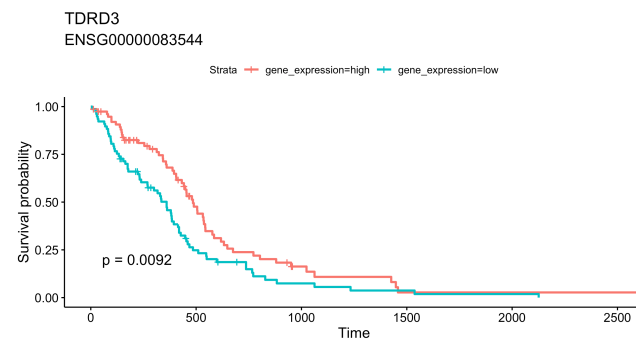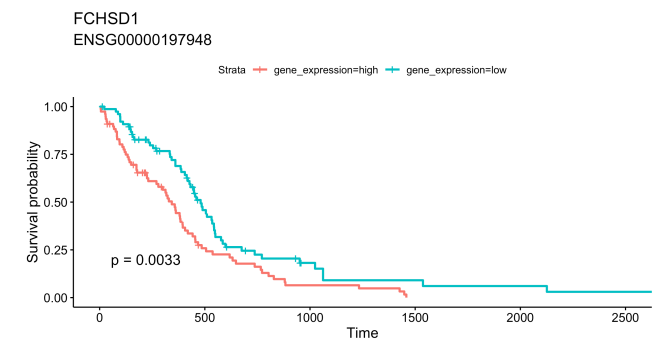

Supplement: Supplementary file 1 [file cancers-12-02183-s001.zip › cancers-855025-SUPPLE-XML/cancers-855025-supple-proof/Suppl_Fig7_glioblastoma_UPregulted_genes.pdf]

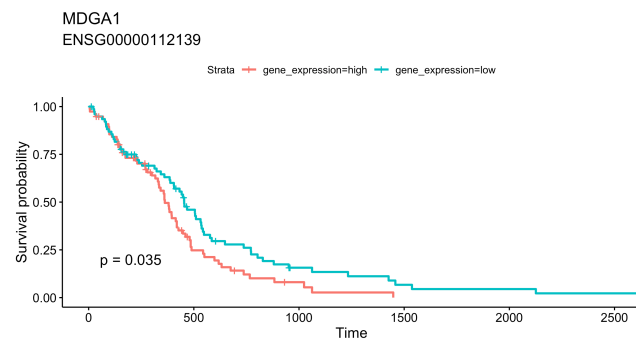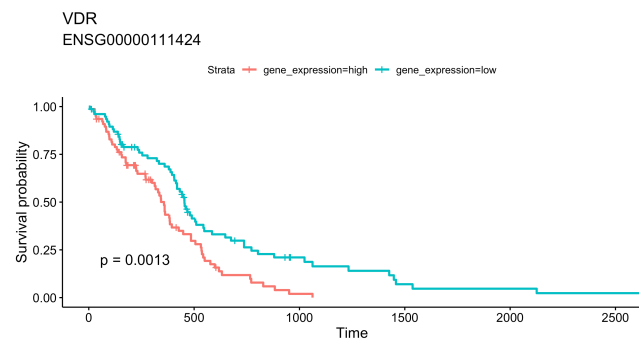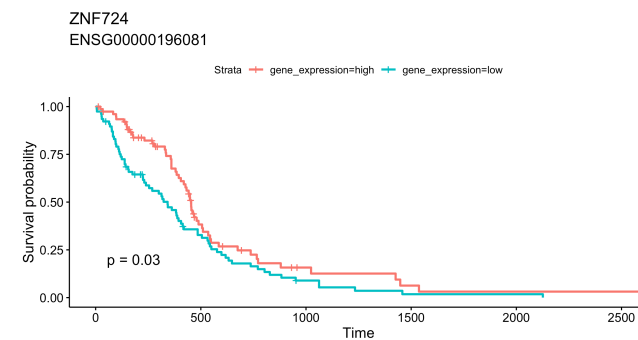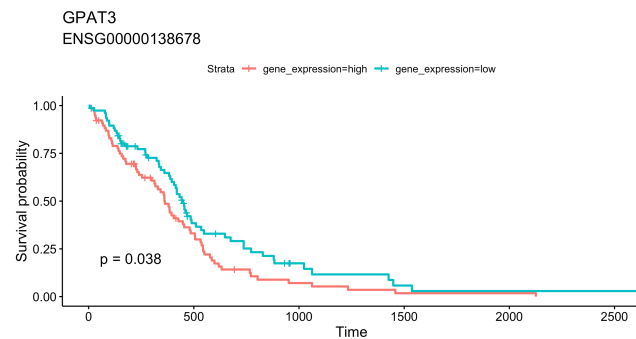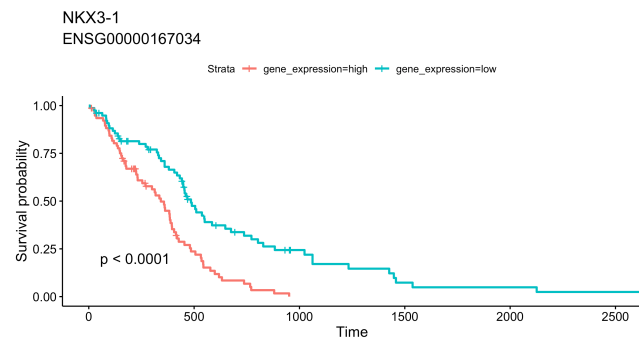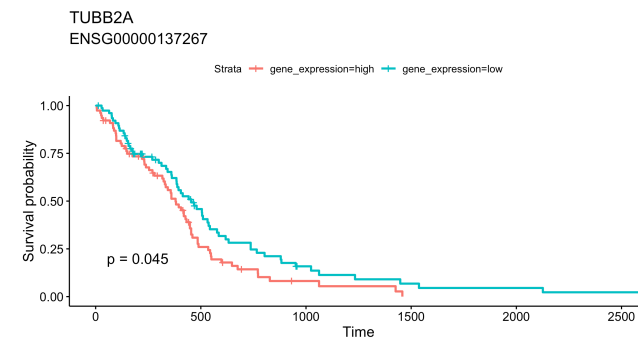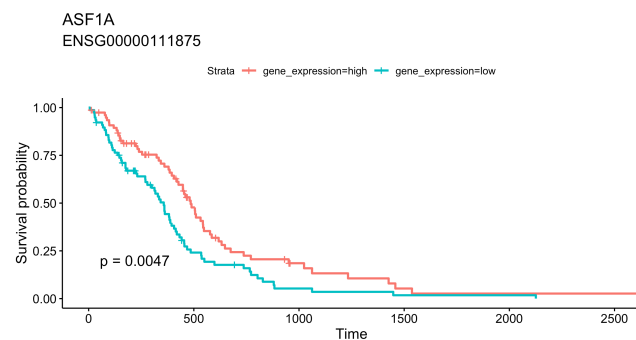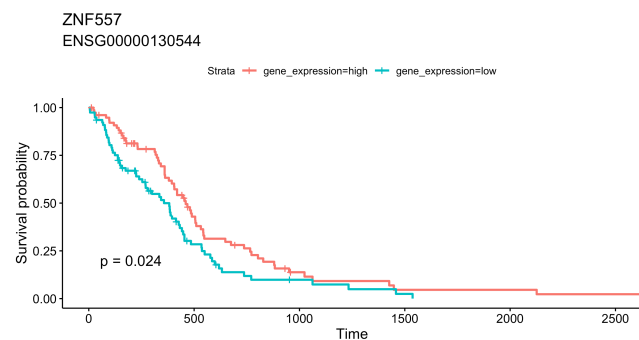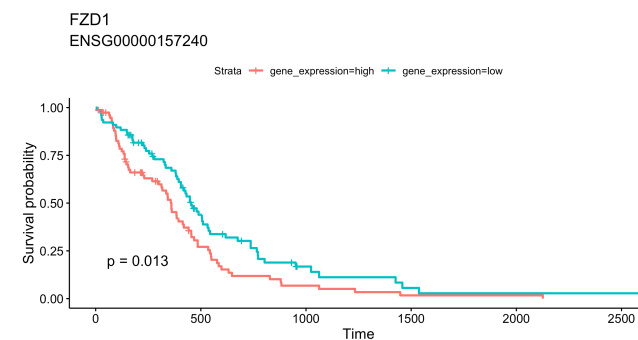

RNF216P1  
ENSG00000196204

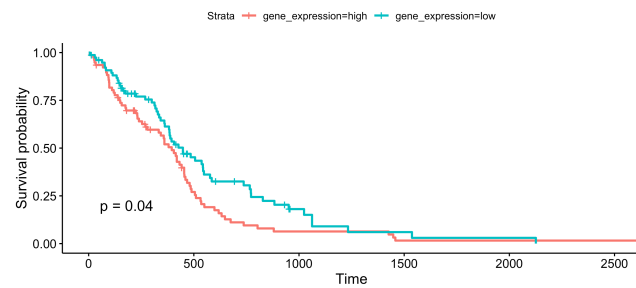

POLR3B  
ENSG00000013503

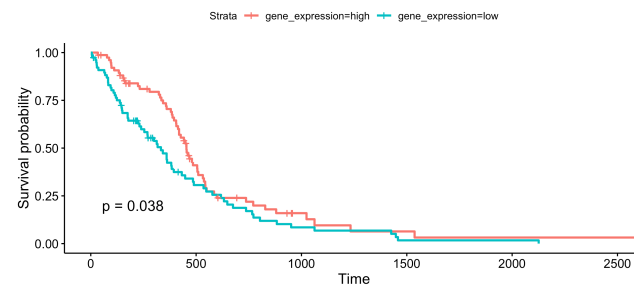

Supplement: Supplementary file 1 [file cancers-12-02183-s001.zip › cancers-855025-SUPPLE-XML/cancers-855025-supple-proof/Suppl_Fig8_glioblastoma_DOWNregulated_genes.pdf]

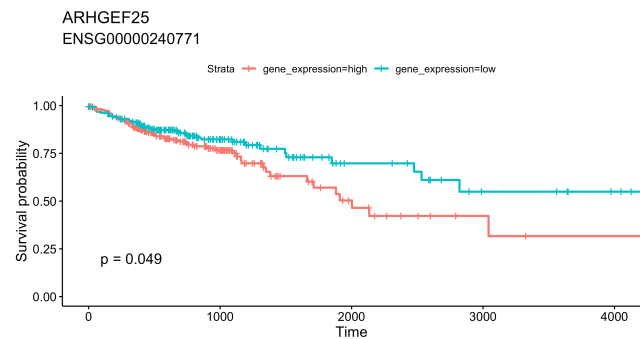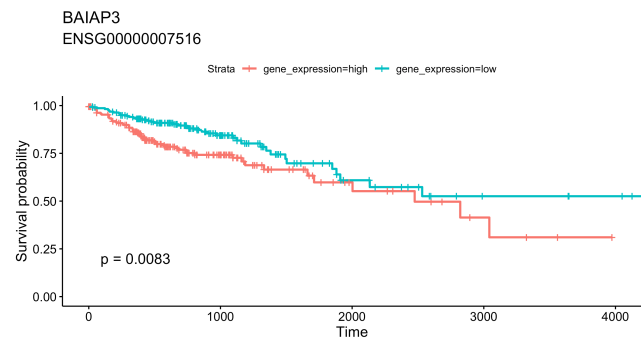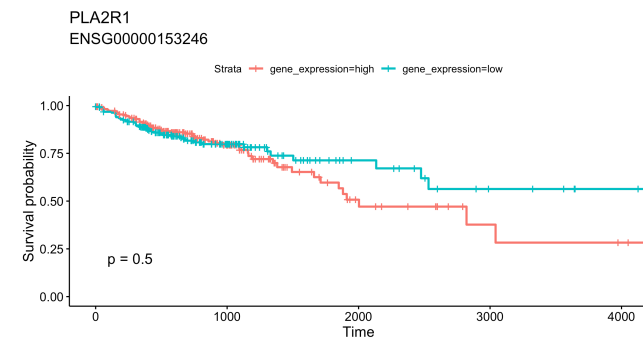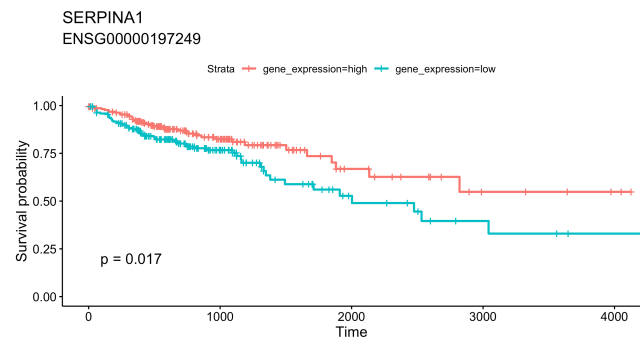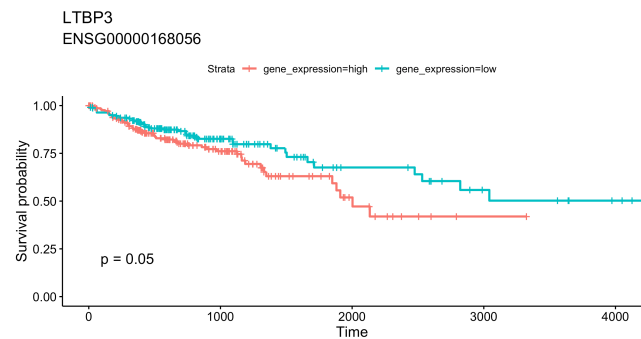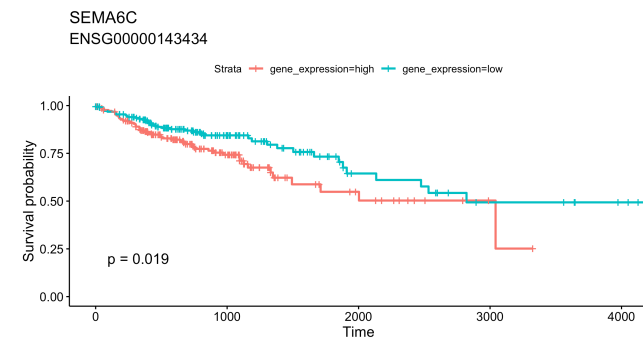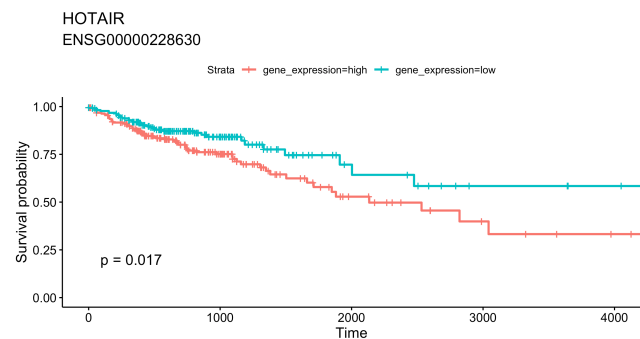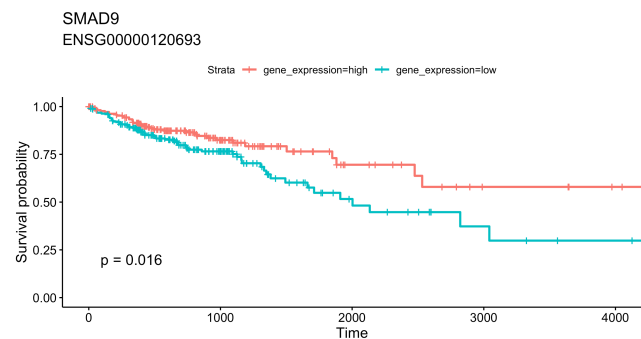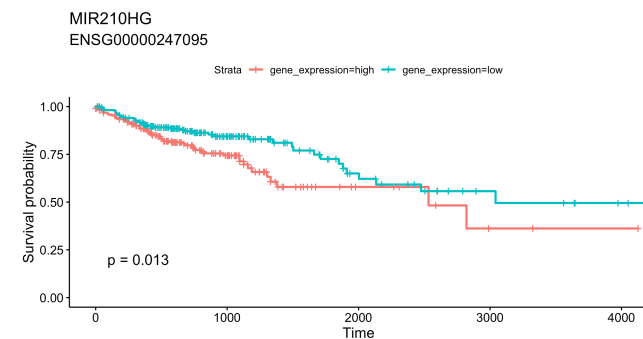

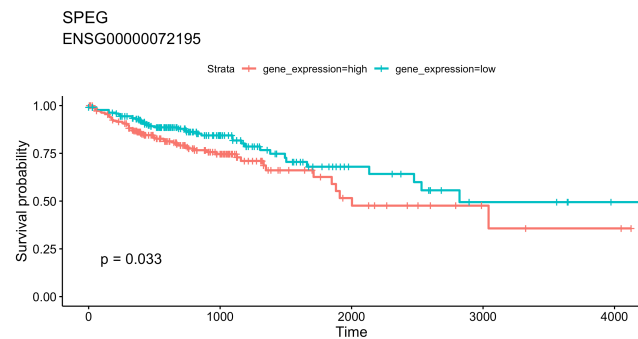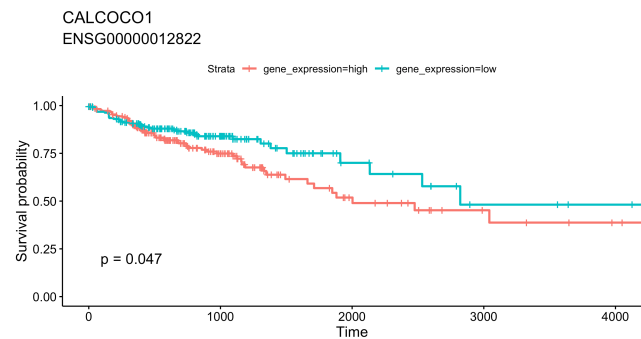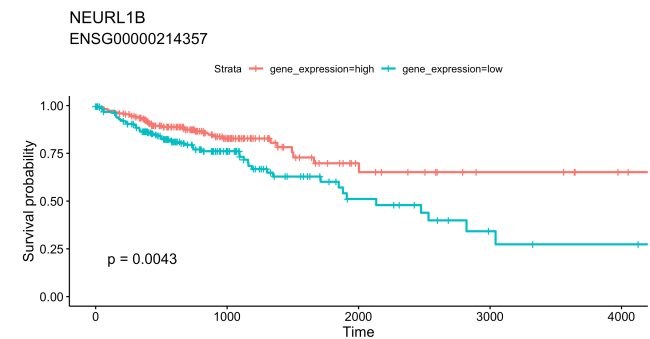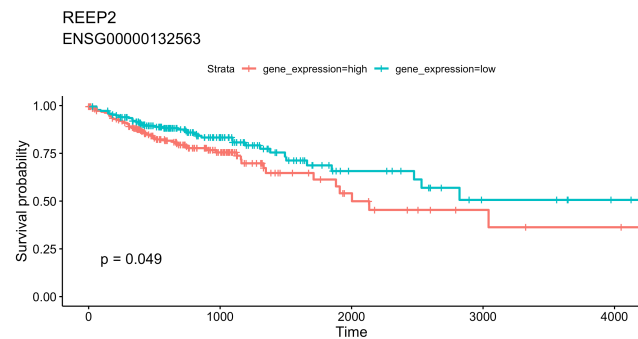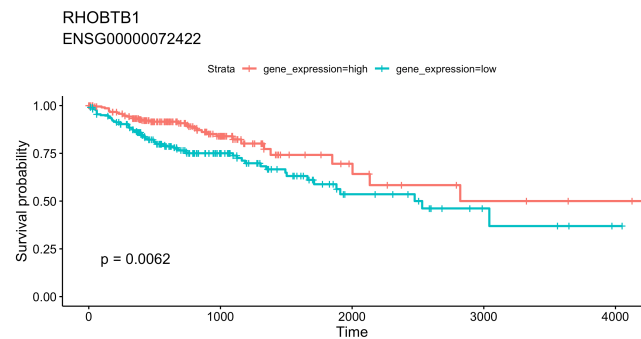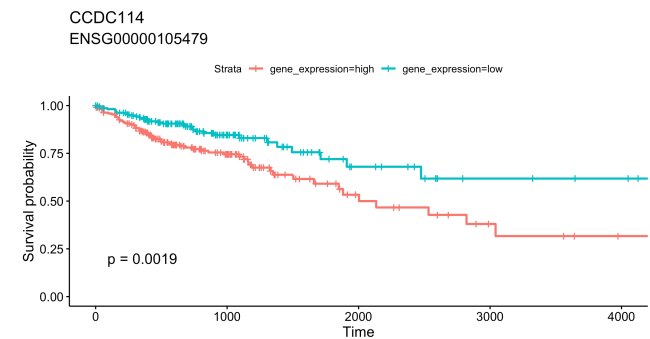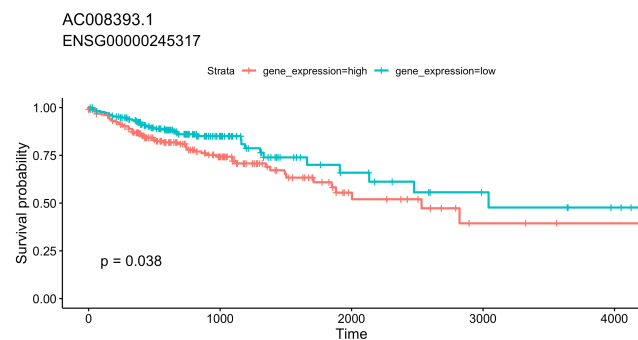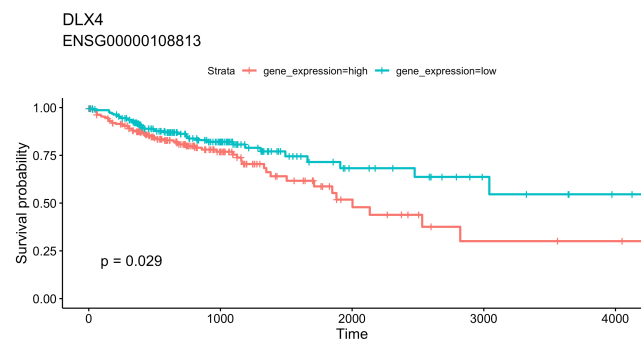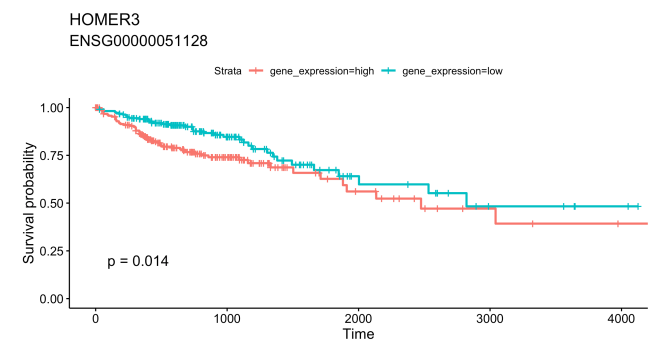

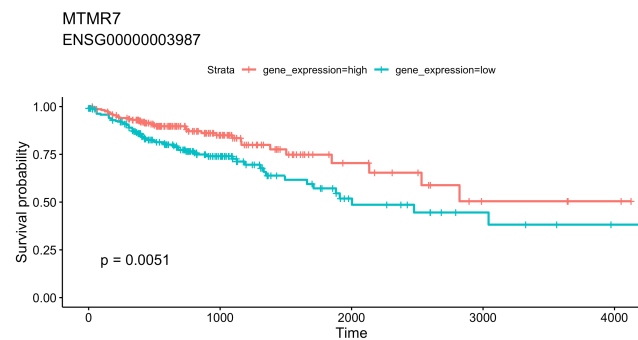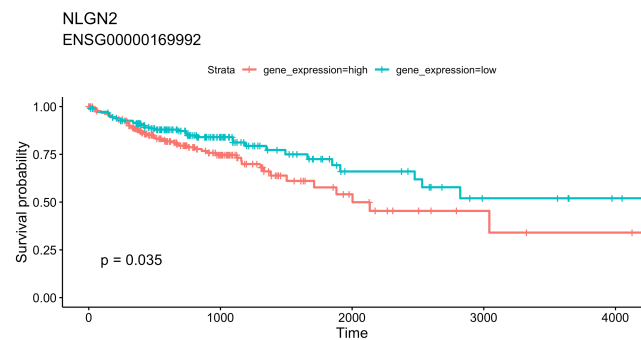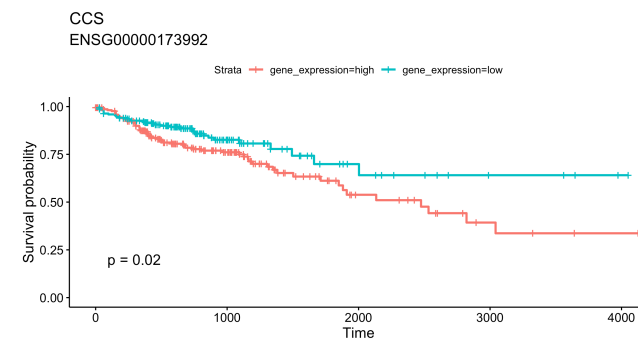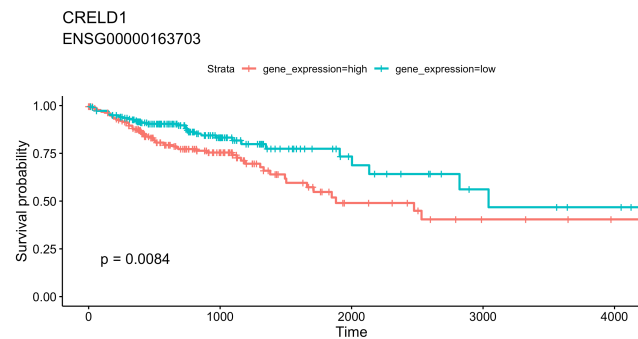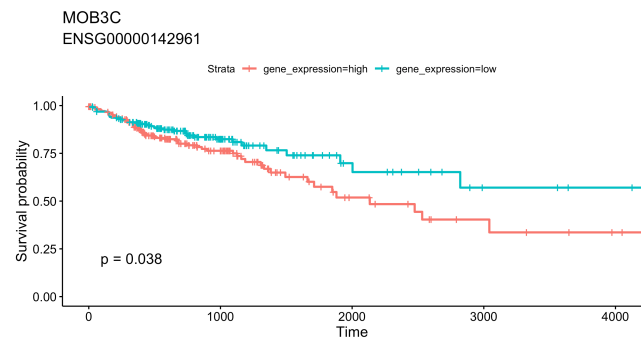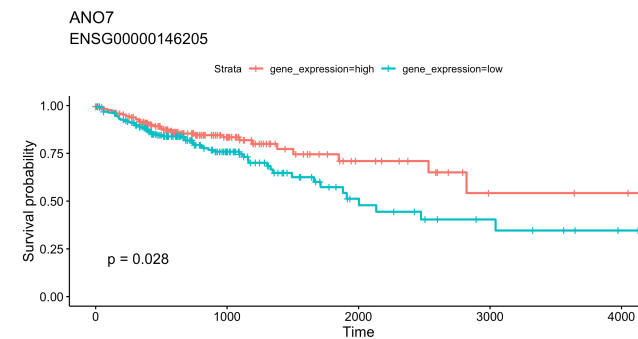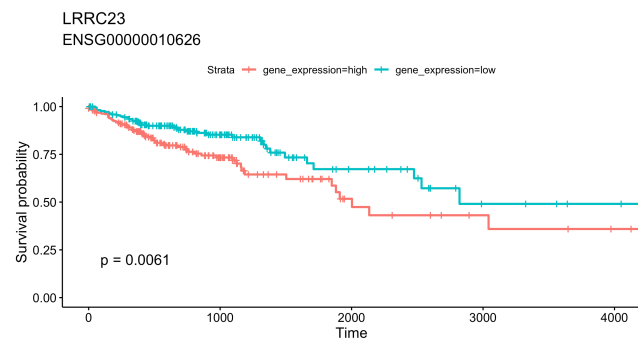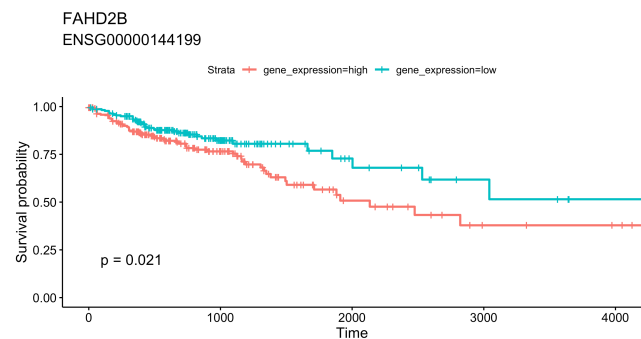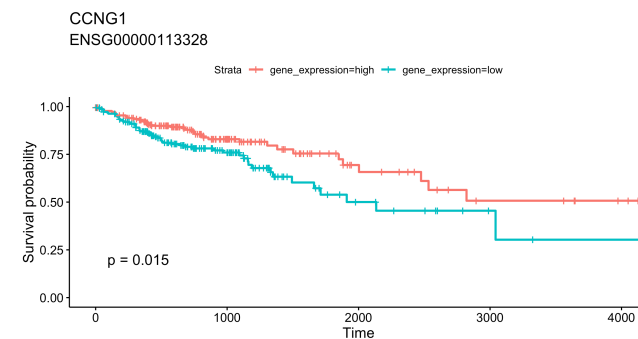

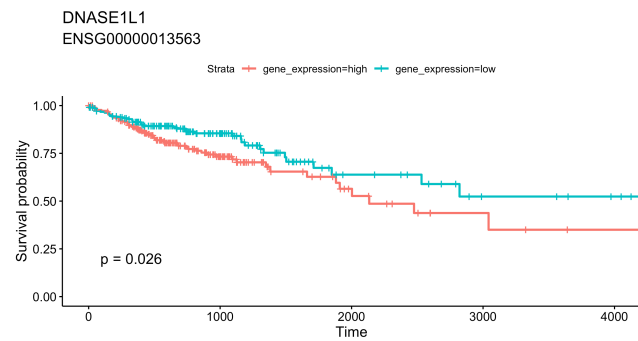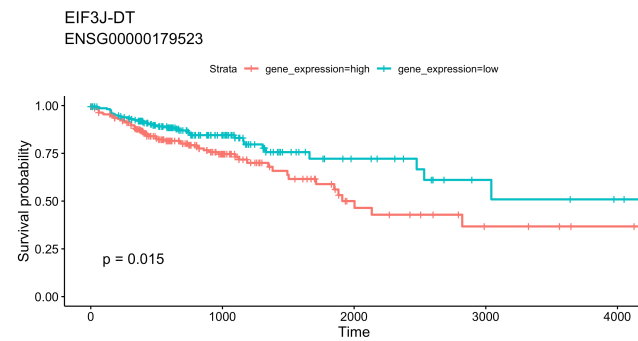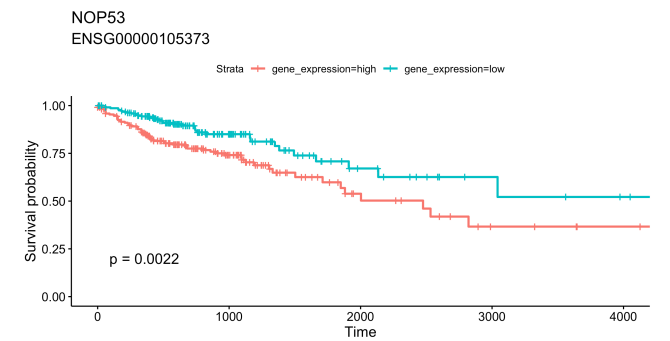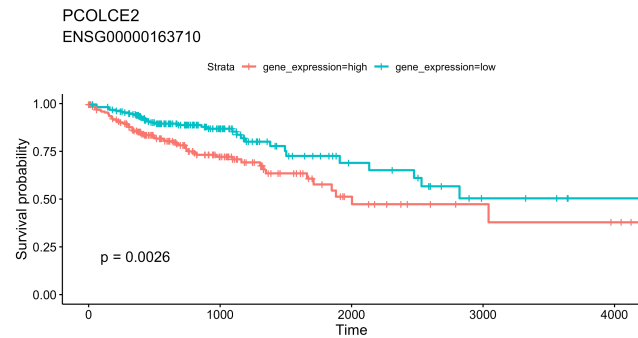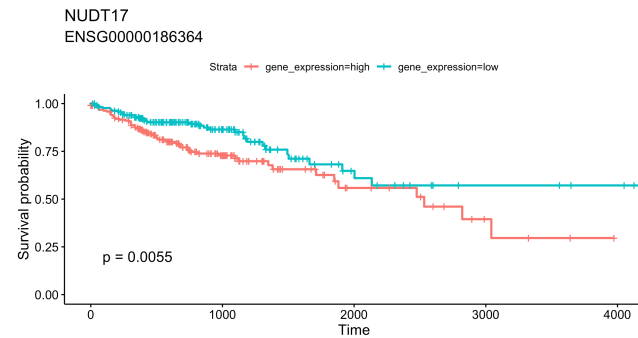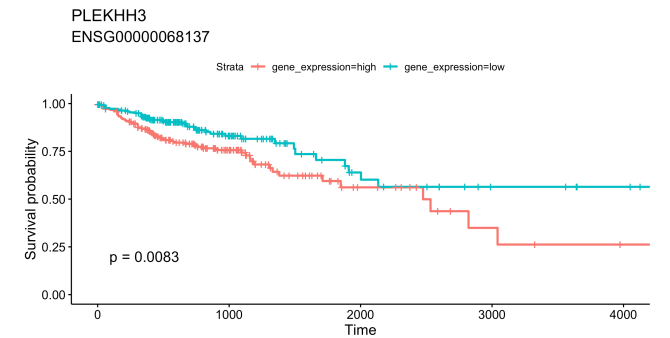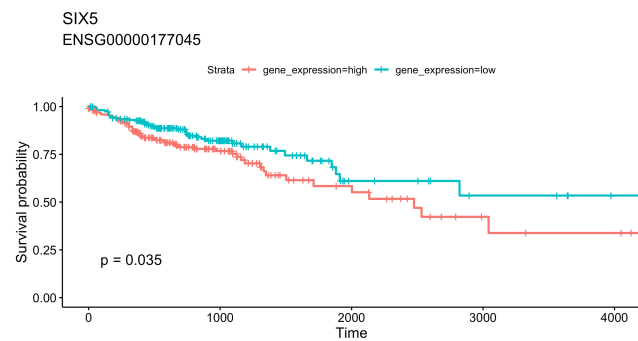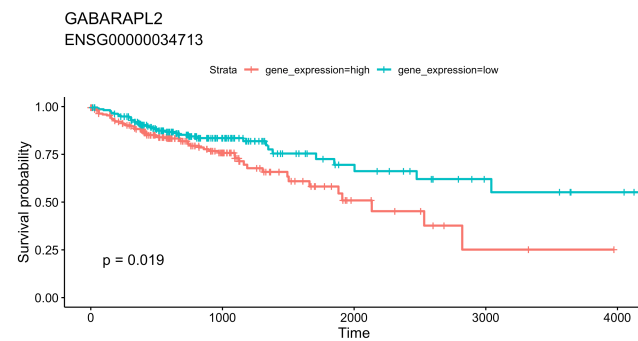

Supplement: Supplementary file 1 [file cancers-12-02183-s001.zip › cancers-855025-SUPPLE-XML/cancers-855025-supple-proof/Suppl_Fig9_colon_UPregulated_genes.pdf]
